# Conjugation of oligonucleotides with activated carbamate reagents prepared by the Ugi reaction for oligonucleotide library synthesis†

**DOI:** 10.1039/d1cb00240f

**Published:** 2022-04-19

**Authors:** Ryosuke Kita, Takashi Osawa, Satoshi Obika

**Affiliations:** Graduate School of Pharmaceutical Sciences, Osaka University 1-6 Yamadaoka Suita Osaka 565-0871 Japan obika@phs.osaka-u.ac.jp; National Institutes of Biomedical Innovation, Health and Nutrition 7-6-8 Saito-Asagi Ibaraki Osaka 567-0085 Japan

## Abstract

The DNA-encoded library (DEL) is a powerful tool for drug discovery. As a result, to obtain diverse DELs, many DNA-compatible chemical reactions have been developed over the past decade. Among the most commonly used reactions in medicinal chemistry, multicomponent reactions (MCRs) can lead to the generation of various compounds in a one-step reaction. In particular, the Ugi reaction can easily provide a peptoid library. Thus, we herein report a solution-phase DEL synthesis based on the Ugi reaction. Using 6-(4-nitrophenoxycarbonylamino)hexanoic acid and *N*-4-nitrophenoxycarbonylglycine as carboxylic acids, peptoids with activated carbamate moieties were obtained as the products of the Ugi reaction. These peptoids were then treated with oligonucleotides bearing a 5′- or 3′-terminal aminohexyl linker to give various oligonucleotide-tagged peptoids in good yields. Moreover, the obtained peptoids could be substituted by a Suzuki cross-coupling reaction and by hydrolysis of the carboxylate ester, followed by condensation with amines. These advances should therefore promote pharmaceutical and medicinal research using DELs.

## Abstract 2

A solution-phase conjugation method based on the Ugi reaction is reported, which enables the synthesis of an oligonucleotide-tagged peptoid library.

## Graphical abstract

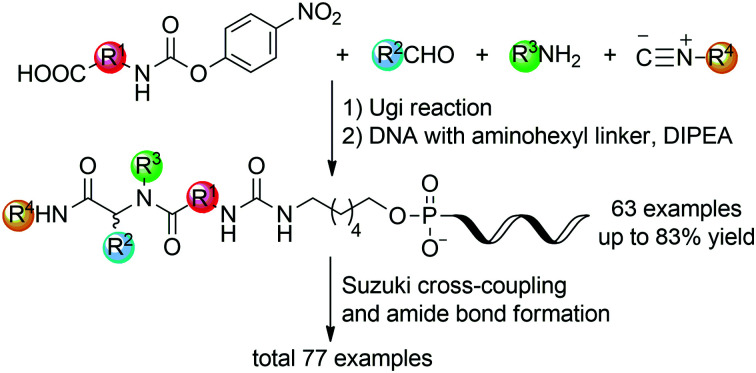

## Introduction

In the early 1990s, the rise of combinatorial chemistry and high-throughput screening (HTS) technologies brought about a major paradigm shift in drug discovery.^1^ HTS technologies enable researchers to quickly perform the millions of genetic, chemical, and pharmacological tests required for drug discovery. Among the various HTS technologies, the concept of DNA-encoded libraries (DELs) was proposed by Brenner and Lerner in 1992,^2^ and this was rapidly applied to the synthesis of oligonucleotide-tagged peptides by Janda and Brenner in 1993.^3^ Since then, significant progress has been made in the field of DEL synthesis,^4,5^ and DELs have become a powerful technology platform for the discovery of ligands for biological targets.^6,7^

From the perspective of medicinal chemistry, multicomponent reactions (MCRs) have a number of advantages over other organic reactions. For example, the majority of MCRs do not require complex transition metal catalysts, and the structures of the reagents can be incorporated into the products, which has attracted particular attention from the viewpoint of green chemistry. Moreover, since MCRs can yield structural diversity and complexity with a minimum number of reaction steps, they are essential to achieving diversity-oriented synthesis, which is of particular importance in the area of drug discovery.^8^ Among the various MCRs developed to date, the Ugi four-component reaction produces a peptoid scaffold from a carboxylic acid, an aldehyde, an amine, and an isocyanide. As a result, this reaction can rapidly provide compound libraries for developing lead structures in medicinal chemistry.^9–12^ As an example, Brunschweiger and coworkers have recently developed an attractive DEL synthesis using isocyanide-based MCRs, such as the Ugi reaction, by employing oligonucleotides bonded to a controlled-pore-glass (CPG) solid support.^13^ They applied their synthetic method to find MDM2 binders and TEAD-YAP interaction inhibitors that perturbed the expression of a gene under the control of these Hippo pathway effectors.^14^

The synthetic routes to DELs developed to date can be divided into two methods, namely solid-phase synthesis and solution-phase synthesis. While solid-phase synthesis can be used for various chemical reactions in which most organic solvents can be employed, the solvents that can be used in the solution-phase route are limited due to the poor solubility of oligonucleotides in organic solvents. In addition, it should be noted that oligonucleotides mounted on solid supports contain impurities that are difficult to remove; this is due to the fact that the solid-supported synthesis of oligonucleotides is carried out as a single continuous operation without any purification.^15^ Furthermore, oligonucleotides are generally synthesized by extending from the 3′-terminal to the 5′-terminal. Therefore, synthesis of the 3′-terminal modified DNA library, which is required for a dual pharmacophore-type DNA library using fragment-based drug discovery (FBDD),^16–20^ is extremely difficult using the solid-phase method, although is relatively facile *via* the solution-phase method. Given this background, we considered that the development of a conjugation method based on the solution-phase Ugi four-component reaction would be valuable in the context of drug discovery based on DELs.

**Scheme 1:**
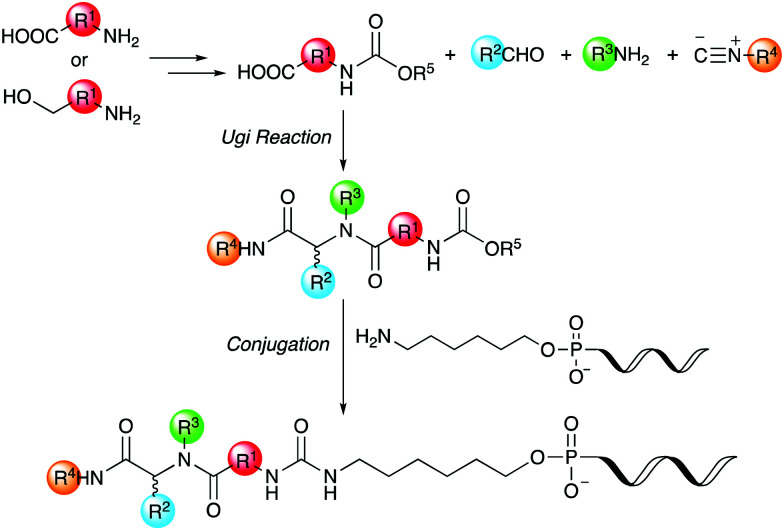
Conjugation strategy with DNA using Ugi reaction products derived from reactive carboxylic acids.

Thus, we herein report a method for the conjugation of oligonucleotides containing an aminohexyl linker with a Ugi reaction product synthesized from a carboxylic acid bearing a carbamate moiety (Scheme 1). Amino acids and amino alcohols, which are the starting materials for obtaining the carboxylic acid reagents, are easily available and have a great structural diversity. In addition, carbamate generally reacts easily with amines to form urea bonds under weakly basic conditions during conjugation reaction, while it hardly react with amines under neutral or weakly acidic conditions during the Ugi reaction. In this study, focusing on the conjugation of DNA with a ligand in the DEL synthesis, the conjugation reaction between oligonucleotides and peptoids prepared by the Ugi reaction and subsequent substitution of the oligonucleotide-tagged peptoid are investigated.

## Results and discussion

### Development of reaction conditions for DEL synthesis

**Scheme 2:**
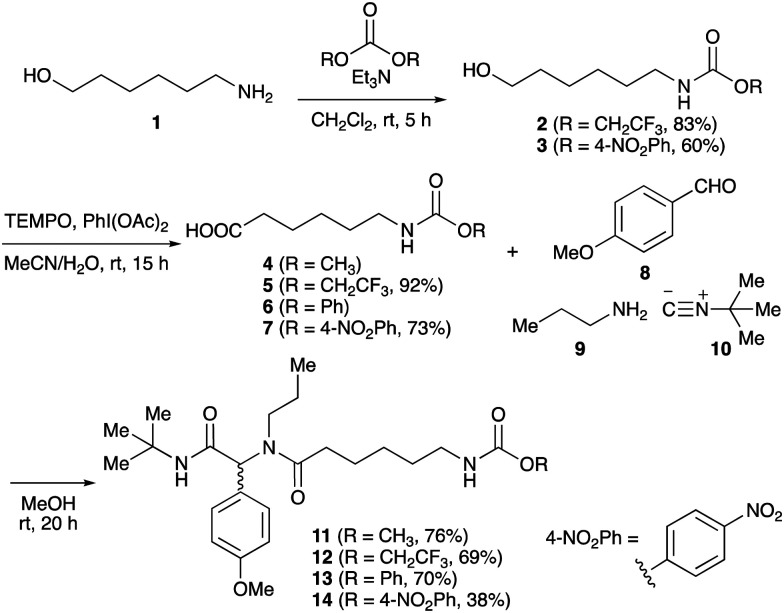
Ugi reactions using *N*-protected amino acids as carboxylic acid reagents.

To initiate our studies, *N*-protected 6-aminohexanoic acids 4–7 were prepared as carboxylic acids for the Ugi reaction (Scheme 2). *O*-Methyl carbamate 4^21^ and *O*-phenyl carbamate 6^22^ were synthesized according to literature procedures. For the syntheses of *O*-2,2,2-trifluoroethyl carbamate 5 and *O*-4-nitrophenyl carbamate 7, 6-amino-1-hexanol 1 was converted into 2 and 3, followed by oxidation of the primary alcohol using TEMPO and PhI(OAc)_2_ to yield 5 and 7, respectively. Anisaldehyde 8, *n*-propylamine 9, *tert*-butyl isocyanide 10, and the obtained four carboxylic acids 4–7 were mixed in MeOH, yielding the desired peptoids 11–14 in yields of 38–76%.

To investigate the reactivities of the synthesized peptoids 11–14, urea bond formation was carried out using a T10mer oligonucleotide conjugated with an aminohexyl group *via* a 5′-terminal phosphodiester bond (DNA 1) as a model substrate, as outlined in Table 1. In addition, the chromatograms obtained by reversed-phase HPLC before and after the reaction are shown in Fig. S2 (ESI†). Methanol, which is commonly used as a solvent for the Ugi reaction, was added to a mixed solvent of water and DMF for the purpose of this reaction. It was found that *O*-methyl carbamate 11 and *O*-2,2,2-trifluoroethyl carbamate 12 did not react with DNA 1, and no product was obtained (Fig. S2B and C, ESI†). In contrast, urea bond formation was observed using 5 equivalents of 5-*O*-phenyl carbamate 13 (Fig. S2D, ESI†); however, product UD-1 was obtained in a low 10% yield, and the majority of starting material (DNA 1) remained (Table 1, entry 3). Furthermore, the treatment of 5 equivalents of *O*-4-nitrophenyl carbamate 14 with DNA 1 yielded the desired product UD-1 (31%) together with recovery of the starting material, DNA 1, in 40% yield (Fig. S2E (ESI†), Table 1, entry 4). From the above results, it was concluded that the 4-nitrophenoxy group was the optimal leaving group relative to the methoxy, 2,2,2-trifluoroethoxy, and phenoxy groups. By increasing the amount of 14 to >50 equivalents, the reaction proceeded completely to give UD-1 as the sole product (Table 1, entries 5–7).

**Table 1:** Urea bond formation between purified peptoids 11–14 and DNA 1^a^

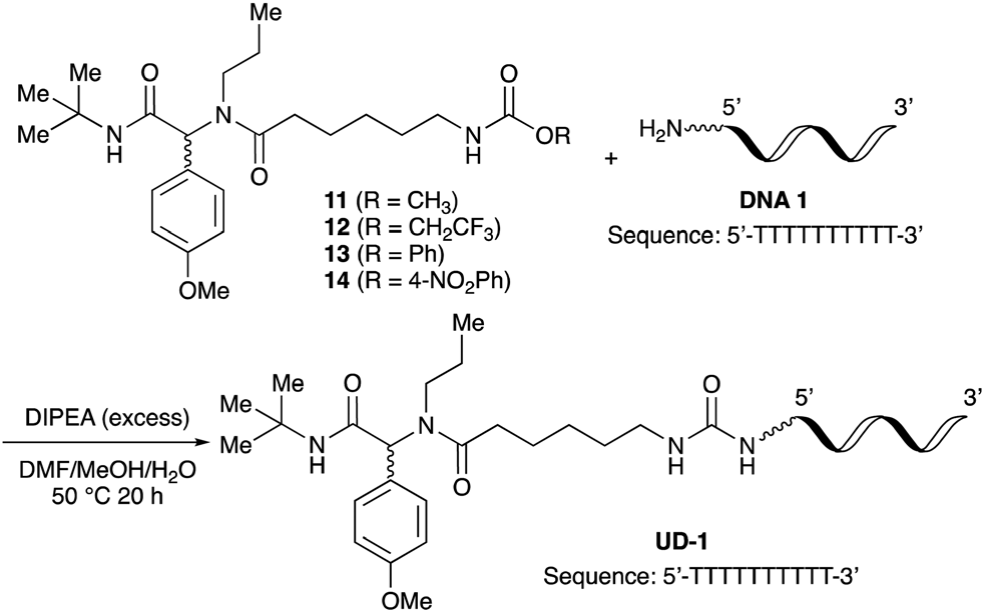				
Entry	DNA 1	Peptoid	UD-1^a^	Recovered DNA 1^a^
1	20 nmol	11 (100 nmol, 5 eq.)	0%	93%
2	20 nmol	12 (100 nmol, 5 eq.)	0%	96%
3	20 nmol	13 (100 nmol, 5 eq.)	10%	74%
4	20 nmol	14 (100 nmol, 5 eq.)	31%	40%
5	20 nmol	14 (200 nmol, 10 eq.)	55%	21%
6	20 nmol	14 (1000 nmol, 50 eq.)	87%	0%
7	20 nmol	14 (2000 nmol, 100 eq.)	85%	0%

a Isolated yield after HPLC-purification.

### Conjugation using the crude peptoids

**Fig. 1:**
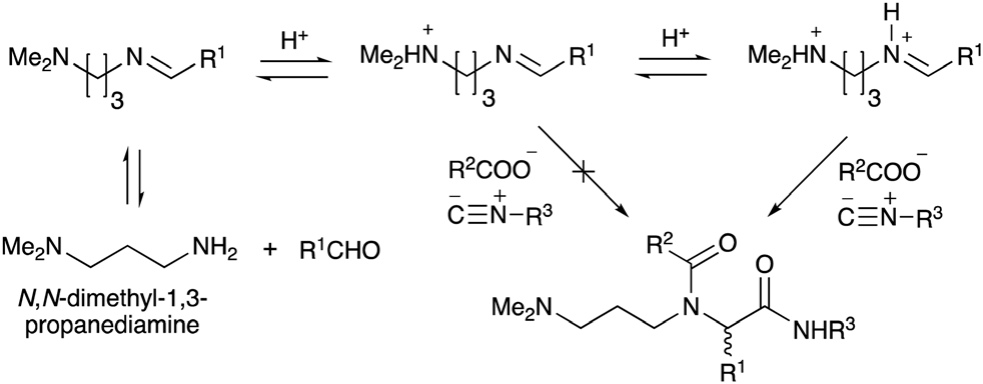
The effect of adding 1 equivalent of HCl (or *p*-TsOH) to the Ugi reaction based on *N,N*-dimethyl-1,3-propanediamine.

To improve the efficiency of DEL synthesis using the activated carbamates, the direct conjugation of DNA with the obtained peptoids was investigated without purification after the Ugi reaction. More specifically, following the Ugi reaction between *O*-4-nitrophenyl carbamate 7, anisaldehyde 8, *n*-propylamine 9, and *tert*-butyl isocyanide 10, the reaction solution was diluted with DMF, and conjugation with DNA 1 was performed using 50 equivalents of the crude peptoid 14 based on the results of the previous experiments (Table 1 and Fig. 1). As a result, the desired product UD-1 was isolated in 26% yield, along with the corresponding carboxylic acid (UD-2, 31% yield), which was obtained by a condensation reaction between residual carbamate 7 and DNA 1 (Table 2, entry 1). Therefore, to improve the yield of the conjugated product UD-1, the amounts of carbamate 7 and isocyanide 10 were reduced to limit the amount of unreacted 7 that was present (Table 2, entries 2–4). It was found that 0.8 equivalents of 7 and 10 gave the desired UD-1 in a good yield (34%, entry 3) compared with the other conditions examined (entries 1, 2, and 4). From these results, the conjugation of crude peptoid 14 with AGCT mix 12mer oligonucleotide (DNA 2) was investigated to establish a foothold in DEL synthesis, which successfully produced UD-3 in 32% yield (Table 2, entry 5).

**Table 2:** Urea bond formation between the crude peptoids and oligonucleotides bearing an aminohexyl linker (DNA 1 and DNA 2)

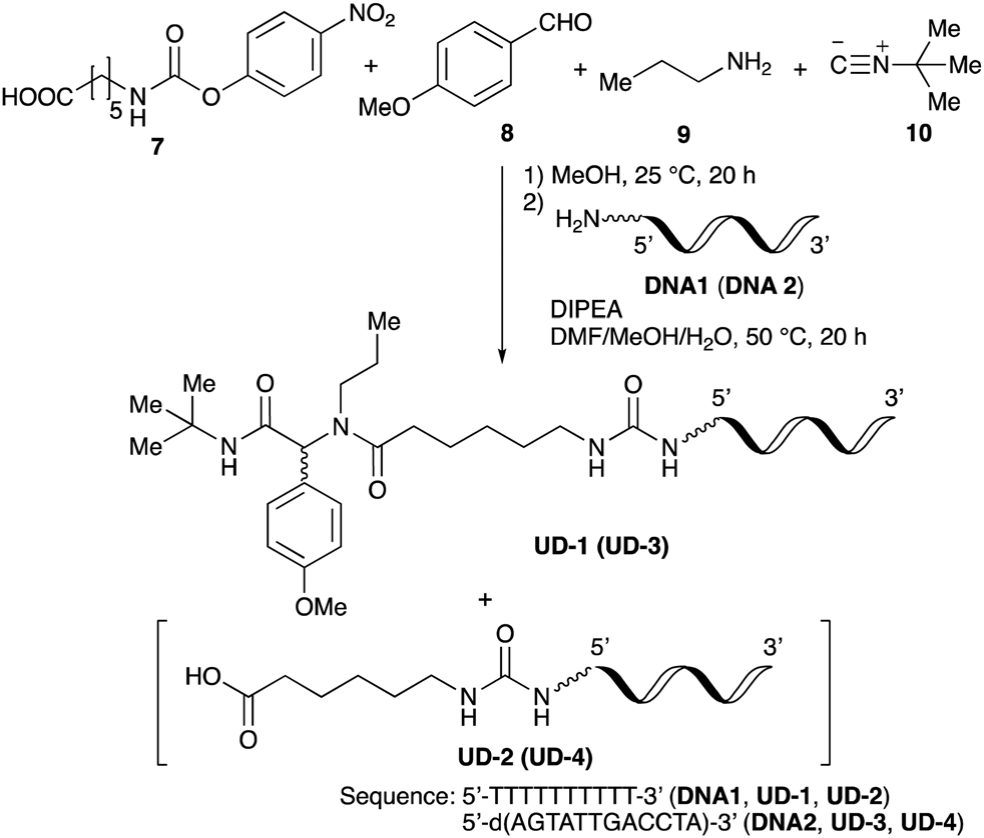					
Entry	DNA 1 (DNA 2)	7 (Crude 14)^c^	Equivalent ratio (7 : 8 : 9 : 10)	UD-1 (UD-3)^d^	UD-2 (UD-4)^d^
1^a^	20 nmol	1000 nmol	1 : 1 : 1 : 1	26%	31%
2^a^	20 nmol	1000 nmol	0.9 : 1 : 1 : 0.9	27%	34%
3^a^	20 nmol	1000 nmol	0.8 : 1 : 1 : 0.8	34%	22%
4^a^	20 nmol	1000 nmol	0.7 : 1 : 1 : 0.7	31%	28%
5^b^	20 nmol	1000 nmol	0.8 : 1 : 1 : 0.8	32%	37%

a The starting material was DNA 1.

b The starting material was DNA 2.

c The amount of crude 14 was calculated assuming that carboxylic acid 7 was completely converted.

d Isolated yield after HPLC purification.

### Substrate scope and limitations

With the optimized conditions in hand, we then carried out DNA-library synthesis utilizing a range of readily available and structurally diverse reagents. Various aldehydes, amines, and isocyanides were employed to investigate the substrate scope and limitations of the reaction, and the results are summarized in Table 3. The reagents include various functional groups, such as aromatic, aliphatic, methoxycarbonyl, amino, and hydroxy groups, which produced moderate to high yields of the desired products. The optimized reaction conditions, as outlined in Table 2, were found to be tolerant to many different functional groups. However, in some cases, the conjugation reaction did not take place to any great extent. For example, the combination of *p*-anisaldehyde and methyl isocyanoacetate did not yield the desired conjugated products (UD-25 and 26), regardless of the type of amine. In addition, using *N*,*N*-dimethyl-1,3-propanediamine as an amine reagent led to the desired conjugated products (UD-10, 17, 24, 33, 40, 47, and 54) forming in a low yield, likely due to the tertiary amine moiety inhibiting formation of the iminium ion, which is the key step in the Ugi reaction (Fig. 1). Based on this speculation, 1 eq. of hydrochloric acid (or *p*-TsOH) was added during the Ugi reaction, which dramatically improved the yield of the conjugation reaction (up to 63%).

**Table 3:** Substrate scope and limitations of urea bond formation using peptoids prepared by combination of the Ugi reaction products and DNA 2

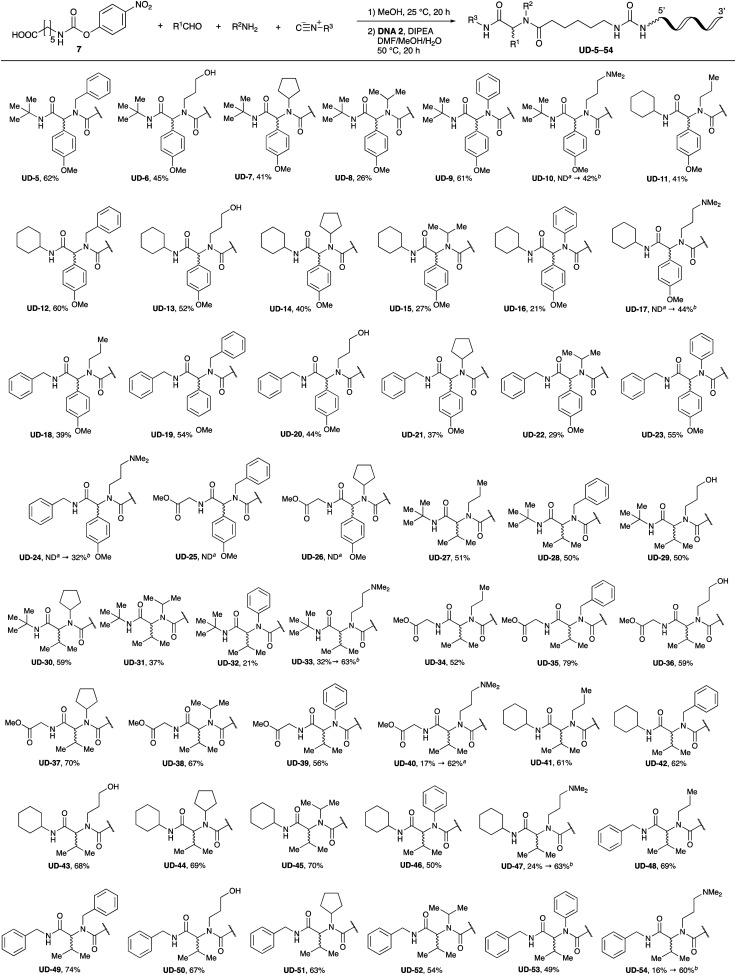

a Not detected.

b 1 equivalent of HCl was added in Ugi reaction.

From the above results, it was apparent that various aldehydes, amines, and isocyanides can be applied to this reaction. Thus, to find an alternative carboxylic acid reagent, we attempted to find a carboxylic acid reagent that can be used in the Ugi reaction. In our method, the carboxylic acid must contain an amino group. Therefore, a derivative of the amino acid glycine (16)^23,24^ was prepared by the condensation of glycine *tert*-butyl ester 15 and bis(4-nitrophenyl)carbonate followed by acidic deprotection of the *tert*-butyl group. Subsequently, 16 was converted to its corresponding peptoid through the Ugi reaction, and conjugation with DNA 2 was carried out to obtain UD-55–61 in a high yield (Table 4, 41–83%). It is worth pointing out that both natural and unnatural amino acids, which are readily available, can be employed in our Ugi-based conjugation strategy.

**Table 4:** Urea bond formation between DNA 2 and the crude peptoid prepared using 4-nitrophenoxycarbonyl glycine 16

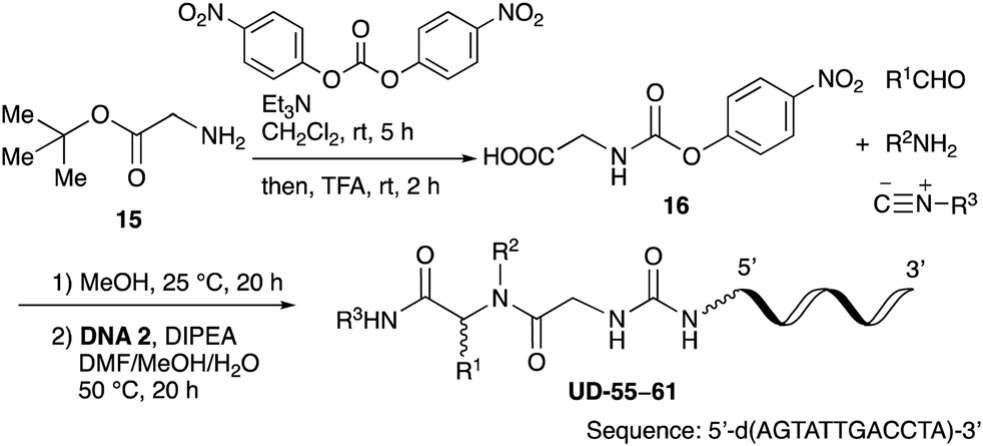					
Entry	R^1^CHO	R^2^NH_2_	R^3^–N^+^≡C^−^	Product	Yield^a^ (%)
1	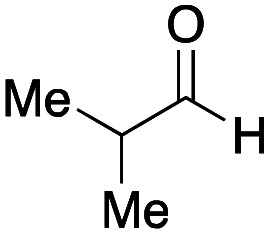	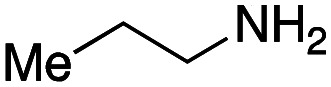	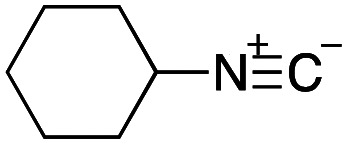	UD-55	46
2	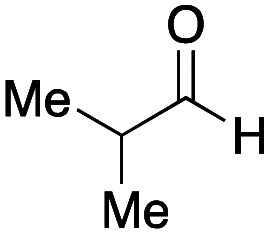	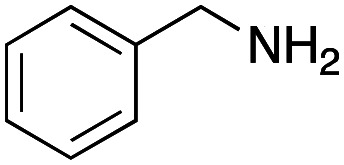	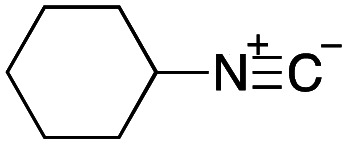	UD-56	83
3	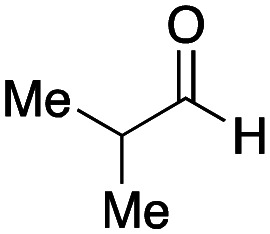	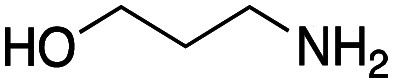	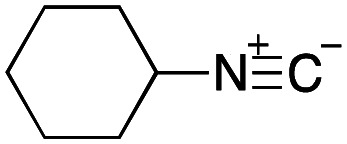	UD-57	41
4	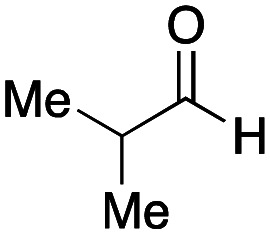	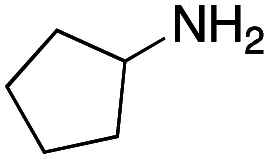	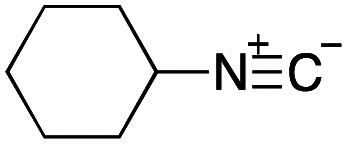	UD-58	64
5	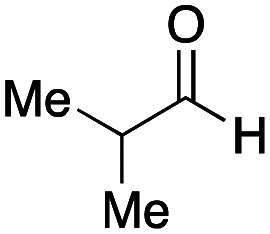	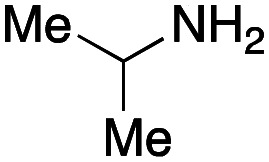	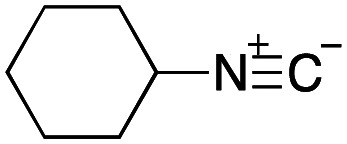	UD-59	63
6	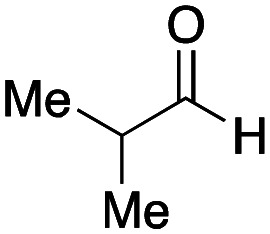	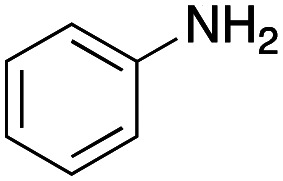	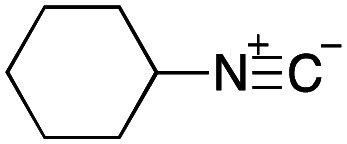	UD-60	45
7^b^	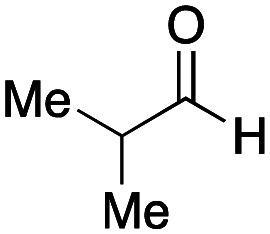	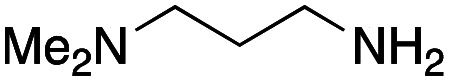	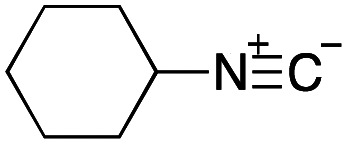	UD-61	45

a Isolated yield after HPLC purification.

b 1 equivalent of HCl was added in Ugi reaction.

**Scheme 3:**
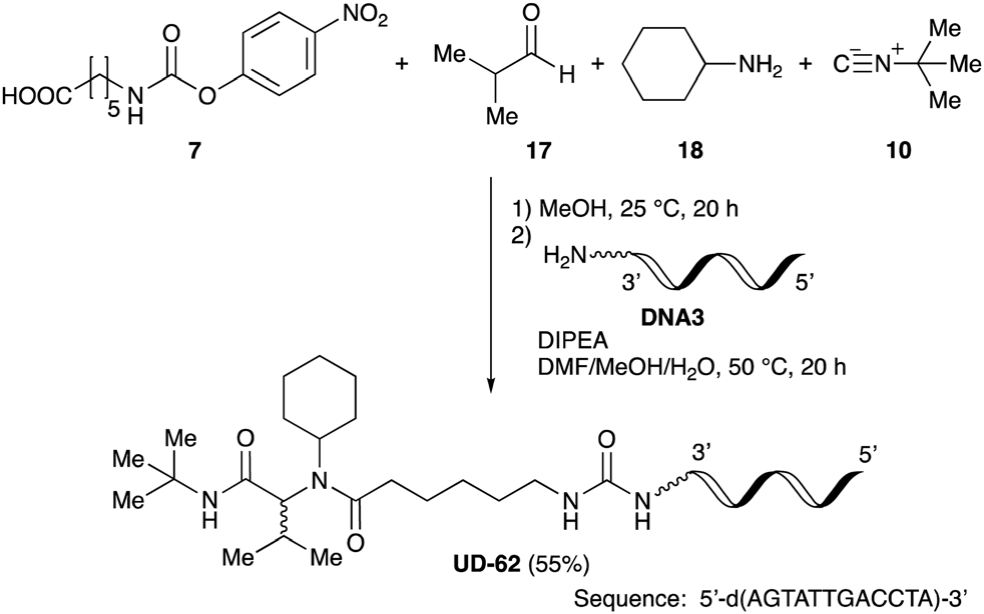
Urea bond formation using DNA 3 which contains an aminohexyl linker introduced *via* a 3′-terminal phosphodiester bond.

As mentioned above, the synthesis of 3′-terminal modified DNA is generally difficult using the solid-phase method, since DNA is prepared by extending from the 3′-terminal to the 5′-terminal. Thus, to apply our method to the synthesis of a 3′-terminal modified DNA library, a conjugation reaction using an oligonucleotide attached to an aminohexyl group *via* a 3′-terminal phosphodiester bond (DNA3) was carried out. More specifically, DNA 3 was treated with a carbamate reagent, prepared from isobutyraldehyde 17, cyclohexylamine 18, *tert*-butyl isocyanide 10, and carboxylic acid 7 to give UD-62 in 55% yield (Scheme 3).

**Scheme 4:**
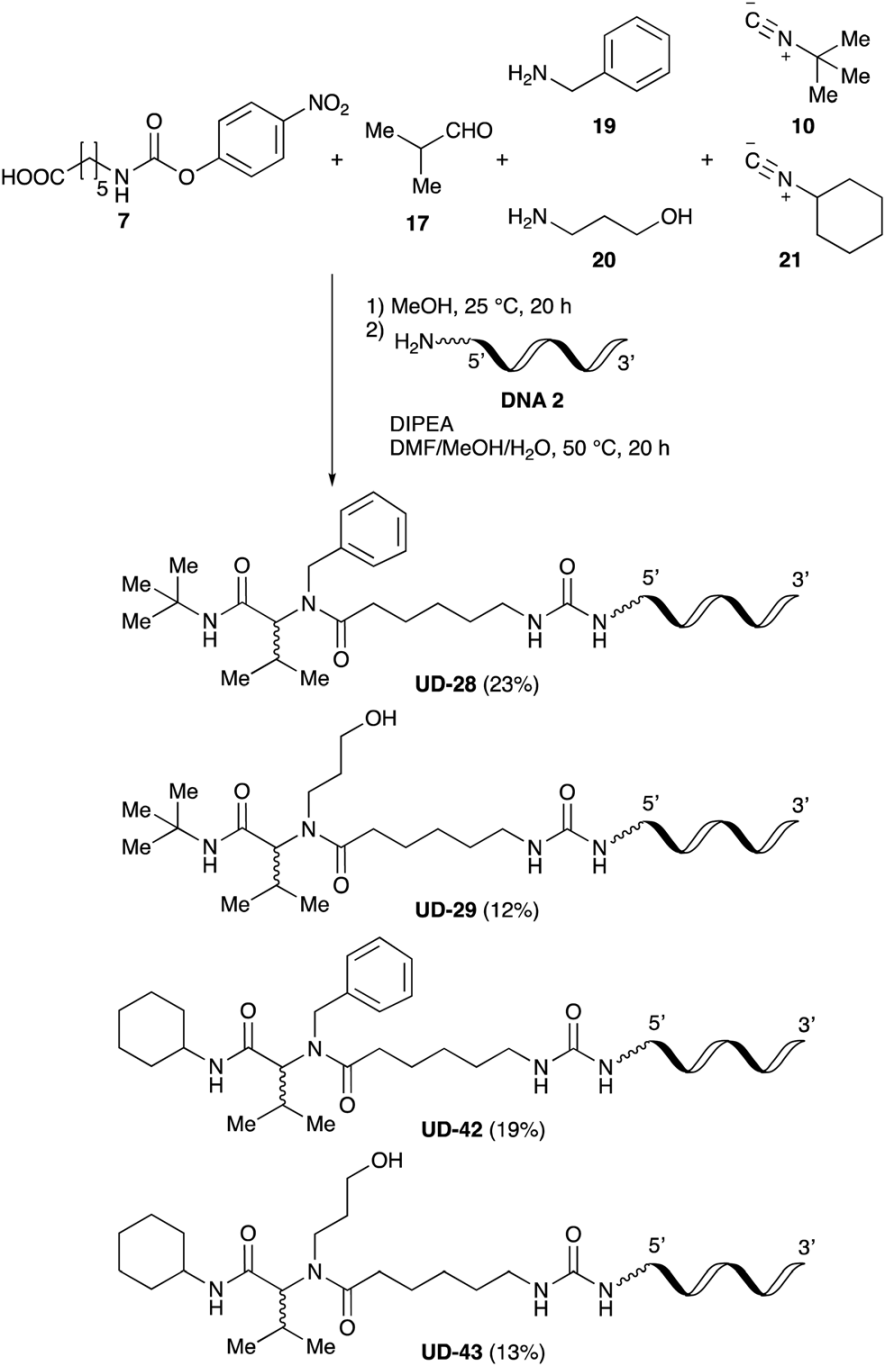
Urea bond formation using DNA 2 and the crude peptoid prepared using a carboxylic acid 7, an aldehyde 17, amines 19, 20, and isocyanides 10, 21.

Finally, we examined the Ugi reaction and conjugation, using a reaction mixture of a carboxylic acid 7, an aldehyde 17, two amines 19, 20, and two isocyanides 10, 21, as a model experiment to easily synthesize various DELs (Scheme 4). This reaction could successfully give four desired products UD-28 (23%), UD-29 (12%), UD-42 (19%), UD-43 (13%).

### Substitution of the peptoids

**Scheme 5:**
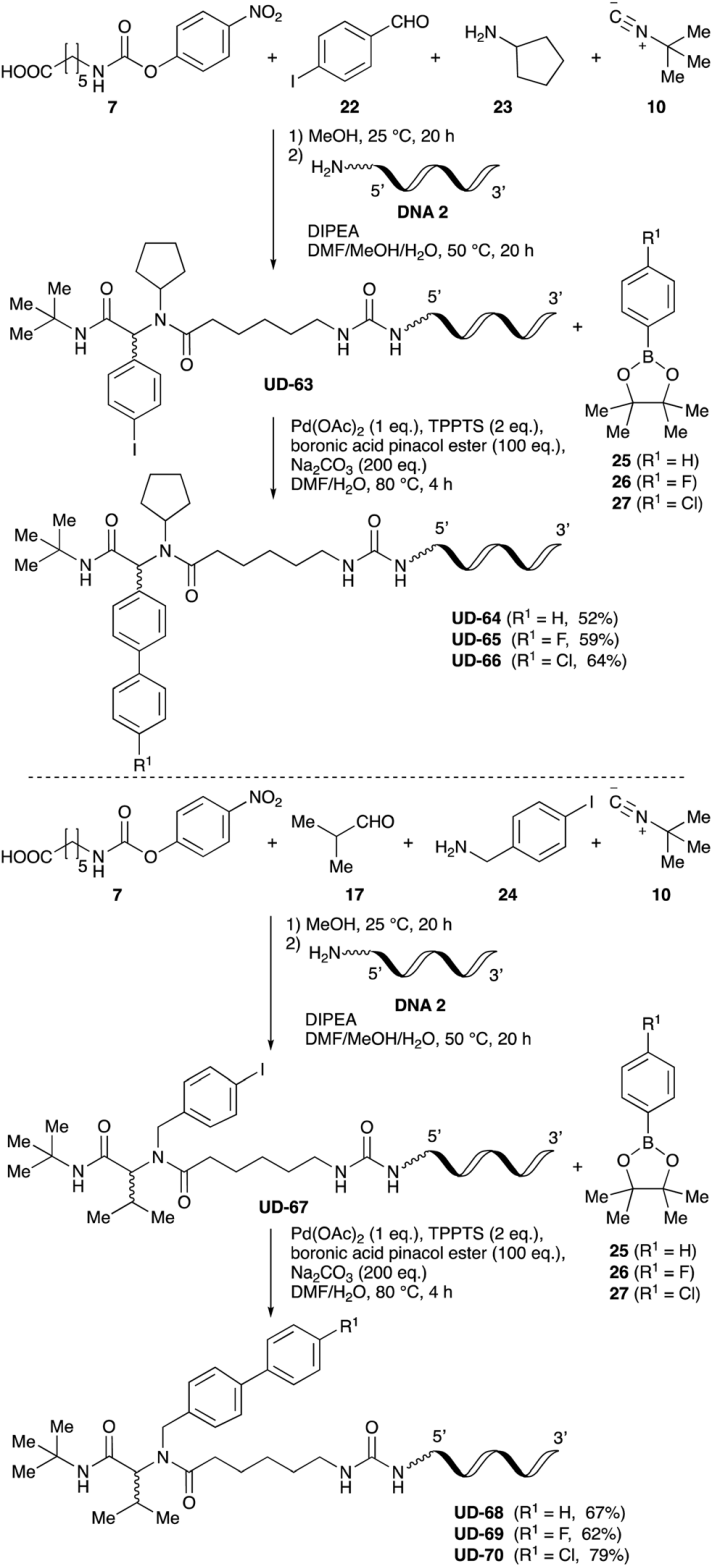
Chemical substitution of UD-63 and UD-67 by Suzuki cross-coupling.

To expand the application range of our method, the derivatization of peptoids was investigated. As the method of derivatization, we chose the Suzuki cross-coupling reaction and the condensation reaction of a carboxylic acid with an amine, since great progress has been made on these reactions in the context of DEL synthesis and in the synthesis of chemically modified oligonucleotides.^25–34^ For modification by the Suzuki cross-coupling reaction, the Ugi reaction and subsequent conjugation process were initially carried out using 4-iodobenzaldehyde 22 or 4-iodobenzylamine 24 to give the substrate for the coupling reaction (*i.e.*, UD-63, 50% yield; or UD-67, 56% yield). The obtained UD-63 and UD-67 were treated with boronic acid pinacol esters 25–27 in the presence of sodium carbonate and the water-soluble Pd(OAc)_2_-TPPTS complex^35,36^ to give the corresponding biphenyl UD-64–66 and UD-68–70 in yields of 52–79% (Scheme 5).

For amide bond formation, 4-(4,6-dimethoxy-1,3,5-triazin-2-yl)-4-methylmorpholinium chloride (DMTMM) was used as a condensing agent according to a previously described DEL synthesis method.^37^ Initially, peptoid UD-35, which was prepared using methyl isocyanoacetate for the Ugi reaction, was hydrolyzed using aqueous NaOH as a base. The crude carboxylic acid was then successfully converted to triamides UD-71–76 in moderate yields by treatment with various amines in the presence of DMTMM (Table 5). In the substitution reactions, amines with various structures, such as a linear amine (*n*-propylamine, entry 1), a branched amine (cyclohexylamine, entry 3), an aromatic amine (aniline, entry 5), and a secondary amine (piperidine, entry 6), were introduced into the peptoids.

**Table 5:** Chemical substitution of UD-35 by amide bond formation

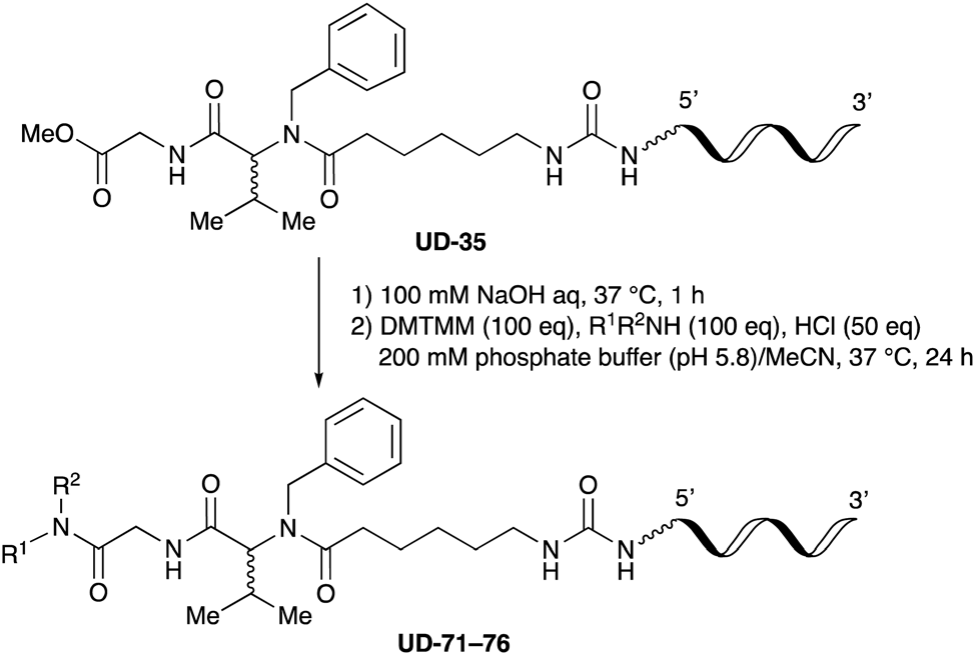			
Entry	Amine (R^1^R^2^NH_2_)	Product	Yield^a^ (%)
1	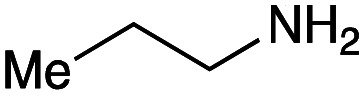	UD-71	62
2	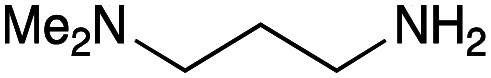	UD-72	41
3	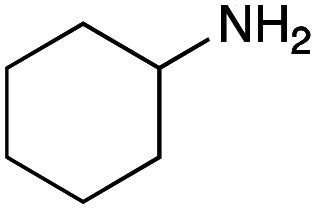	UD-73	29
4	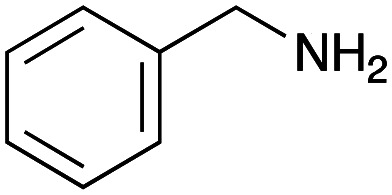	UD-74	53
5	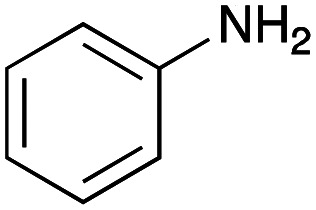	UD-75	82
6	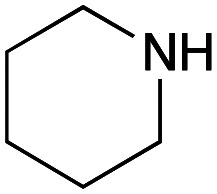	UD-76	41

a Isolated yield after HPLC-purification.

**Scheme 6:**
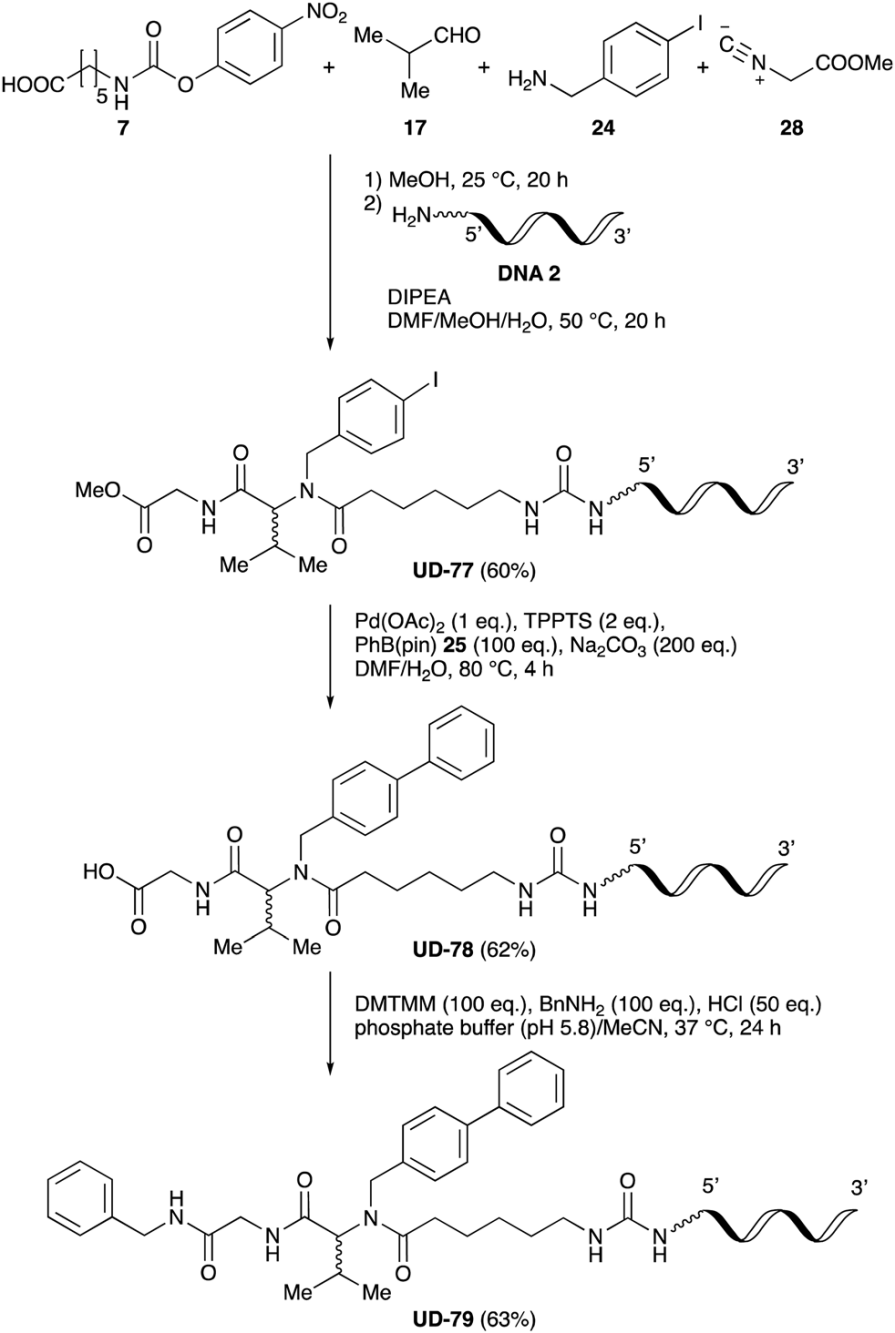
Chemical substitution of peptoid UD-77 by sequential Suzuki cross-coupling and amide bond formation.

Finally, UD-77, which is a substrate for both the Suzuki cross-coupling reaction and amide bond formation, was prepared using carboxylic acid 7, isobutylaldehyde 17, 4-iodobenzylamine 24, methyl isocyanoacetate 28, and DNA 2. The obtained peptoid UD-77 was then converted to biphenyl UD-78 in 62% yield *via* a Suzuki cross-coupling. In this reaction, the methyl ester moiety of UD-77 was hydrolyzed by basic aqueous sodium carbonate, and the condensation of the resulting biphenyl UD-78 with benzylamine was subsequently performed to give triamide UD-79, which was isolated in 63% yield after purification (Scheme 6). As a result, we were able to establish a six-component conjugation method based on the Ugi reaction that is applicable to solution-phase synthesis. In addition, a validation experiment using a DNA without an aminohexyl linker (DNA 2′), which has the same sequence as DNA 2. Natural DNA 2′ did not react under the reaction conditions (Fig. S3–S5, ESI†), which shows that our method based on the Ugi reaction is DNA compatible. The present study has not yet resulted in the production of DEL, the ligation and PCR process for DEL synthesis need to be investigated. However, since the nucleobases necessary for the formation of Watson–Crick base pairs were not damaged during the reactions, our approach has the potential to be applied to drug discovery using DEL.

## Conclusions

In summary, we successfully developed a solution-phase conjugation method for the synthesis of an oligonucleotide-tagged peptoid library, namely a DNA-encoded library. This was achieved using activated carbamate reagents prepared by the Ugi reaction. Importantly, our conjugation method was found to be tolerant to many different functional groups. In our approach, although it is necessary to synthesize a carboxylic acid containing a carbamate moiety, various commercially available amino acids can be used. In addition, we demonstrated that the peptoid constructed by the Ugi reaction could be derivatized by a Suzuki cross-coupling reaction or through a condensation reaction between the amine and a carboxylic acid. In general, ligation of DNA barcode using T4 DNA ligase is required after derivatization of the ligands for DEL synthesis.^3^ In our case, the four components of the Ugi reaction can be encoded by the barcode DNAs corresponding to each of carboxylic acid, aldehyde, amine, and isocyanide. Although it may be necessary to examine the DEL synthesis including a ligation process, we believe that our method is fully applicable to DEL synthesis. Our approach based on the Ugi reaction, which is applicable to solution-phase synthesis, will be expected to promote drug discovery using DNA-encoded libraries.

## Experimental

### General

All moisture-sensitive reactions were conducted in well-dried glassware under a N_2_ atmosphere. All chemicals were purchased from vendors and used as purchased without further purification. Anhydrous CH_2_Cl_2_ and MeOH were used as purchased. NMR experiments were performed on JEOL JNM-ECS300, JNM-ECS400, and JNM-ECA500 spectrometers. ^1^H NMR spectra were recorded at 300 MHz, 400 MHz, and 500 MHz. ^13^C NMR spectra were recorded at 75 MHz, 100 MHz, and 125 MHz. Chemical shift values are reported in parts per million (ppm) relative to internal tetramethylsilane (*δ* = 0.00 ppm) for ^1^H NMR. For ^13^C NMR, the chemical shift values are reported in ppm relative to methanol-d_4_ (*δ* = 49.0 ppm) or chloroform-d_1_ (*δ* = 77.0 ppm). IR spectra were recorded on a JASCO FT/IR-4200 spectrometer. The MALDI-TOF mass spectra of all new compounds were recorded on a JEOL SpiralTOF JMS-S3000. The MALDI-TOF mass of all oligonucleotides was recorded on a Bruker Daltonics Autoflex maX TOF/TOF mass spectrometer. For column chromatography, Fuji Silysia PSQ-100B silica gel was used. The reaction progress was monitored by analytical thin-layer chromatography (TLC) on pre-coated glass sheets (Silica gel 60 F_254_, Merck). Compounds were visualized under 254 nm wavelength UV light and stained with *p*-anisaldehyde and/or ninhydrin. For preparative high performance liquid chromatography (HPLC), SHIMADZU CBM-20A, DGU-20A_5R_, LC-20AD, CTO-20A, SPD-20A, and FRC-10A were used, and for analytical HPLC, SHIMADZU CBM-20A, DGU-20A_3R_, LC-20AD, CTO-20A, SPD-20A, and SIL-20A were employed. The yields of the oligonucleotide-tagged peptoids were calculated by measuring their absorbances at 260 nm on a NanoDrop instrument (DeNovix DS-11). The oligonucleotides conjugated with an aminohexyl group *via* a 5′-terminal phosphodiester bond (DNA 1 and DNA 2) or a 3′-terminal phosphodiester bond (DNA 3) were purchased from GeneDesign, Inc.

### Synthetic procedures

#### 6-*N*-(2,2,2-Trifluoroethoxycarbonyl)amino-1-hexanol (2)

Under a N_2_ atmosphere, bis(2,2,2-trifluoroethyl) carbonate (1.5 mL, 10 mmol) and triethylamine (1.4 mL, 10 mmol) were added to an ice-cold solution of 6-amino-1-hexanol (1.17 g, 10 mmol) in anhydrous CH_2_Cl_2_ (50 mL). After stirring at room temperature for 5 h, the reaction mixture was concentrated to remove the solvent *in vacuo*. The residue (4.80 g) was purified by column chromatography (silica gel 80 g, *n*-hexane : EtOAc = 1 : 3) to yield compound 2 (2.02 g, 83%) as a white solid. IR *ν*_max_/cm^−1^ (KBr): 1219, 1251, 1267, 1292, 1342, 1364, 1391, 1420, 1466, 1478, 1537, 1598, 1666, 1696, 2778, 2860, 2903, 2937, 2971, 3058 and 3338. ^1^H NMR (300 MHz, CDCl_3_) *δ*: 1.22–1.62 (9H, m), 3.62 (2H, q, *J* = 7.0 Hz), 3.64 (2H, q, *J* = 6.0 Hz), 4,45 (2H, q, *J* = 8.5 Hz) and 4.99 (1H, s). ^13^C NMR (75 MHz, CDCl_3_) *δ*: 25.3, 26.3, 29.7, 32.5, 41.1, 60.3 (q, *J* = 36 Hz), 62.7, 123.1 (q, *J* = 275 Hz) and 154.5. HRMS (MALDI) calcd for C_9_H_16_F_3_NNaO_3_ [M + Na]^+^: 266.0980, found: 266.0976.

#### 6-*N*-(4-Nitrophenoxycarbonyl)amino-1-hexanol (3)

Under a N_2_ atmosphere, bis(4-nitrophenyl)carbonate (2.04 g, 10 mmol) and triethylamine (1.4 mL, 10 mmol) were added to an ice-cold solution of 6-amino-1-hexanol (1.17 g, 10 mmol) in anhydrous CH_2_Cl_2_ (50 mL). After stirring at room temperature for 5 h, the reaction mixture was concentrated to remove the solvent *in vacuo*. The residue (4.33 g) was purified by column chromatography (silica gel 80 g, *n*-hexane : EtOAc = 1 : 2) to yield compound 3 (1.69 g, 60%) as a white solid. IR *ν*_max_/cm^−1^ (KBr): 1218, 1251, 1285, 1352, 1488, 1531, 1595, 1620, 1670, 1700, 2758, 2861, 2935, 3024, 3057, 3089, 3114 and 3338. ^1^H NMR (300 MHz, CDCl_3_) *δ*: 1.29 (1H, t, *J* = 5.2 Hz), 1.37–1.49 (4H, m), 1.54–1.67 (6H, m), 3.30 (2H, q, *J* = 6.9 Hz), 3.66 (2H, q, *J* = 6.3 Hz), 5.13 (1H, s), 7.28–7.35 (2H, m) and 8.20–8.28 (2H, m). ^13^C NMR (100 MHz, CDCl_3_) *δ*: 25.4, 26.5, 29.8, 32.6, 41.3, 62.8, 122.1, 125.2, 144.8, 153.3 and 156.1. HRMS (MALDI) calcd for C_13_H_18_N_2_NaO_5_ [M + Na]^+^: 305.1109, found: 305.1108.

#### 6-*N*-(2,2,2-Trifluoroethoxycarbonyl)amino-1-hexanoic acid (5)

TEMPO (230 mg, 1.5 mmol) and PhI(OAc)_2_ (5.24 g, 16 mmol) were added to an ice-cold solution of compound 2 (1.80 g, 7.4 mmol) in CH_3_CN/H_2_O (1 : 1, 40 mL). After stirring at room temperature for 15 h, the reaction mixture was concentrated to remove the solvent *in vacuo*. The residue (4.88 g) was purified by column chromatography (silica gel 80 g, *n*-hexane : EtOAc = 1 : 2 to 1 : 4) to yield compound 5 (1.75 g, 92%) as a white solid. IR *ν*_max_/cm^−1^ (KBr): 1200, 1264, 1298, 1323, 1363, 1414, 1461, 1554, 1699, 2679, 2873, 2952, 3081 and 3332. ^1^H NMR (300 MHz, CDCl_3_) *δ*: 1.33–1.71 (6H, m), 2.37 (2H, t, *J* = 7.5 Hz), 3.20 (2H, q, *J* = 7.0 Hz), 4.45 (2H, q, *J* = 8.5 Hz), 5.03 (1H, s) and 10.7 (1H, s). ^13^C NMR (75 MHz, CDCl_3_) *δ*: 24.1, 26.0, 19.3, 33.7, 41.0, 60.8 (q, *J* = 37 Hz), 123.1 (q, *J* = 275 Hz), 154.5 and 179.5. HRMS (MALDI) calcd for C_9_H_14_F_3_NNaO_4_ [M + Na]^+^: 280.0773, found: 280.0764.

#### 6-*N*-(4-Nitrophenoxycarbonyl)amino-1-hexanoic acid (7)

TEMPO (110 mg, 0.71 mmol) and PhI(OAc)_2_ (2.51 g, 7.8 mmol) were added to an ice-cold solution of compound 3 (1.0 g, 3.5 mmol) in CH_3_CN/H_2_O (1 : 1, 20 mL). After stirring at room temperature for 15 h, the reaction mixture was concentrated to remove the solvent *in vacuo*. The residue (3.88 g) was purified by column chromatography (silica gel 80 g, *n*-hexane : EtOAc = 1 : 1 to 1 : 4) to yield compound 7 (766 mg, 73%) as a white solid. IR *ν*_max_/cm^−1^ (KBr): 1217, 1254, 1276, 1308, 1350, 1412, 1440, 1465, 1490, 1524, 1543, 1598, 1617, 1699, 2687, 2868, 2943, 3082 and 3334. ^1^H NMR (400 MHz, CDCl_3_) *δ*: 1.40–1.49 (2H, m), 1.59–1.74 (5H, m), 2.40 (2H, q, *J* = 7.3 Hz), 3.31 (2H, q, *J* = 6.8 Hz), 5,17 (1H, s), 7.29–7.35 (2H, m) and 8.22–8.28 (2H, m). ^13^C NMR (100 MHz, CD_3_OD) *δ*: 25.7, 27.3, 30.3, 34.8, 41.9, 123.4, 126.0, 146.1, 155.6, 157.8 and 177.5. HRMS (MALDI) calcd for C_13_H_16_N_2_NaO_6_ [M + Na]^+^: 319.0900, found: 319.0901.

#### Methyl [6-((2-*tert*-butylamino)-1-(4-methoxyphenyl)-2-oxoethyl)propylamino]-6-oxohexyl]carbamate (11)

Under a N_2_ atmosphere, anisaldehyde 8 (0.61 mL, 5.0 mmol) was added to a solution of *n*-propylamine 9 (0.41 mL, 5.0 mmol) in anhydrous MeOH (10 mL) at room temperature. After stirring at room temperature for 2 h, compound 4^21^ (946 mg, 5.0 mmol) and *tert*-butyl isocyanide 10 (0.56 mL, 5.0 mmol) were added to this reaction solution at room temperature. After stirring at room temperature for a further 20 h, the reaction mixture was concentrated to remove the solvent *in vacuo*. The residue (2.89 g) was purified by column chromatography (silica gel 50 g, *n*-hexane : EtOAc = 2 : 1 to 1 : 3) to yield compound 11 (1.71 g, 76%) as a colorless oil. IR *ν*_max_/cm^−1^ (KBr): 1262, 1365, 1392, 1455, 1520, 1542, 1625, 1652, 1687, 1721, 2629, 2839, 2872, 2970, 3064, 3215 and 3321. ^1^H NMR (300 MHz, CDCl_3_) *δ*: 0.66 (3H, t, *J* = 7.5 Hz), 0.86–1.01 (1H, m), 1.32–1.76 (16H, m), 2.28–2.48 (2H, m), 3.16–3.24 (4H, m), 3.66 (3H, s), 3.82 (3H, s), 4.87 (1H, s), 5.67 (1H, s), 5.74 (1H, s), 6.88 (2H, d, *J* = 8.0 Hz) and 7.31 (2H, d, *J* = 8.0 Hz). ^13^C NMR (75 MHz, CDCl_3_) *δ*: 11.1, 23.1, 24.3, 24.8, 26.2, 28.4, 29.5, 33.1, 33.7, 40.6, 48.1, 51.2, 51.8, 55.1, 61.8, 113.9, 127.6, 130.6, 159.3, 169.5, 173.7 and 176.3. HRMS (MALDI) calcd for C_24_H_39_N_3_NaO_5_ [M + Na]^+^: 472.2787, found: 472.2788.

#### 2,2,2-Trifluoroethyl [6-((2-*tert*-butylamino)-1-(4-methoxyphenyl)-2-oxoethyl)propylamino]-6-oxohexyl]carbamate (12)

Under a N_2_ atmosphere, anisaldehyde 8 (0.61 mL, 5.0 mmol) was added to a solution of *n*-propylamine 9 (0.41 mL, 5.0 mmol) in anhydrous MeOH (10 mL) at room temperature. After stirring at room temperature for 2 h, compound 5 (1.28 g, 5.0 mmol) and *tert*-butyl isocyanide 10 (0.56 mL, 5.0 mmol) were added to this reaction solution at room temperature. After stirring at room temperature for a further 20 h, the reaction mixture was concentrated to remove the solvent *in vacuo*. The residue (2.50 g) was purified by column chromatography (silica gel 50 g, *n*-hexane : EtOAc = 2 : 1 to 2 : 3) to yield compound 12 (1.78 g, 69%) as a colorless oil. IR *ν*_max_/cm^−1^ (KBr): 1210, 1268, 1291, 1363, 1417, 1480, 1529, 1548, 1644, 1684, 1795, 2876, 2965, 3076 and 3322. ^1^H NMR (300 MHz, CDCl_3_) *δ*: 0.65 (3H, t, *J* = 7.5 Hz), 0.88–1.00 (1H, m), 1.32–1.74 (16H, m), 2.39–2.49 (2H, m), 3.18–3.27 (4H, m), 3.82 (3H, s), 4.45 (2H, q, *J* = 8.5 Hz), 5.27 (1H, s), 5.61 (1H, s), 5.72 (1H, s), 6.88 (2H, d, *J* = 8.0 Hz) and 7.31 (2H, d, *J* = 8.0 Hz). ^13^C NMR (175 MHz, CDCl_3_) *δ*: 11.1, 23.1, 24.7, 26.1, 28.5, 29.3, 33.0, 40.9, 48.2, 48.3, 51.3, 55.1, 60.6 (q, *J* = 36 Hz), 113.9, 123.1 (q, *J* = 275 Hz), 127.7, 130.7, 154.5, 159.4, 169.5 and 173.6. HRMS (MALDI) calcd for C_25_H_38_F_3_N_3_NaO_5_ [M + Na]^+^: 540.2661, found: 540.2661.

#### Phenyl[6-((2-*tert*-butylamino)-1-(4-methoxyphenyl)-2-oxoethyl)propylamino]-6-oxohexyl]carbamate (13)

Under a N_2_ atmosphere, anisaldehyde 8 (0.61 mL, 5.0 mmol) was added to a solution of *n*-propylamine 9 (0.41 mL, 5.0 mmol) in anhydrous MeOH (10 mL) at room temperature. After stirring at room temperature for 2 h, compound 6^22^ (1.25 g, 5.0 mmol) and *tert*-butyl isocyanide 10 (0.56 mL, 5.0 mmol) were added to this reaction solution at room temperature. After stirring at room temperature for a further 20 h, the reaction mixture was concentrated to remove the solvent *in vacuo*. The residue (2.56 g) was purified by column chromatography (silica gel 50 g, *n*-hexane : EtOAc = 2 : 1 to 1 : 2) to yield compound 13 (1.78 g, 70%) as a colorless oil. IR *ν*_max_/cm^−1^ (KBr): 1250, 1365, 1392, 1454, 1494, 1512, 1538, 1634, 1668, 1743, 2872, 2970, 3072 and 3337. ^1^H NMR (300 MHz, CDCl_3_) *δ*: 0.66 (3H, t, *J* = 7.5 Hz), 0.86–1.00 (1H, m), 1.32–1.77 (16H, m), 2.36–2.46 (2H, m), 3.20–3.33 (4H, m), 3.81 (3H, s), 5.34 (1H, s), 5.66 (1H, s), 5.74 (1H, s), 6.88 (2H, d, *J* = 8.0 Hz) and 7.11–7.37 (7H, m). ^13^C NMR (75 MHz, CDCl_3_) *δ*: 11.2, 23.2, 24.8, 26.3, 28.5, 29.4, 33.1, 40.9, 48.3, 51.4, 55.2, 62.1, 114.0, 121.6, 125.1, 127.7, 129.1, 130.7, 151.1, 154.7, 159.4, 169.5 and 173.7. HRMS (MALDI) calcd for C_29_H_41_N_3_NaO_5_ [M + Na]^+^: 534.2944, found: 534.2939.

#### 4-Nitrophenyl [6-((2-*tert*-butylamino)-1-(4-methoxyphenyl)-2-oxoethyl)propylamino]-6-oxohexyl]carbamate (14)

Under a N_2_ atmosphere, anisaldehyde 8 (0.61 mL, 5.0 mmol) was added to a solution of *n*-propylamine 9 (0.41 mL, 5.0 mmol) in anhydrous MeOH (10 mL) at room temperature. After stirring at room temperature for 2 h, compound 7 (1.48 g, 5.0 mmol) and *tert*-butyl isocyanide 10 (0.56 mL, 5.0 mmol) were added to this reaction solution at room temperature. After stirring at room temperature for a further 20 h, the reaction mixture was concentrated to remove the solvent *in vacuo*. The residue (2.95 g) was purified by column chromatography (silica gel 50 g, *n*-hexane : EtOAc = 3 : 1 to 2 : 3) to yield compound 14 (1.06 g, 38%) as a colorless oil. IR *ν*_max_/cm^−1^ (KBr): 1214, 1251, 1346, 1392, 1422, 1455, 1487, 1512, 1522, 1614, 1681, 1748, 2834, 2871, 2967, 3081, 3116 and 3326. ^1^H NMR (300 MHz, CDCl_3_) *δ*: 0.66 (3H, t, *J* = 7.5 Hz), 0.94–0.97 (1H, m), 1.31–1.78 (16H, m), 2.34–2.50 (2H, m), 3.19–3.35 (4H, m), 3.82 (3H, s), 5.59–5.71 (3H, m), 6.88 (2H, d, *J* = 8.0 Hz), 7.29–7.34 (4H, m) and 8.21–8.26 (2H, m). ^13^C NMR (75 MHz, CDCl_3_) *δ*: 11.2, 23.2, 24.6, 26.1, 28.6, 29.1, 33.0, 41.0, 51.5, 55.3, 62.4, 114.1, 122.0, 125.1, 127.7, 130.1, 144.6, 153.2, 156.1, 159.6, 169.5 and 173.6. HRMS (MALDI) calcd for C_29_H_40_N_4_NaO_7_ [M + Na]^+^: 579.2795, found: 579.2791.

### General procedure of urea bond formation using crude peptoids prepared by the Ugi reaction and conjugation with oligonucleotides

The aldehyde (50 μmol) and amine (50 μmol) were added to anhydrous MeOH (100 μL) in a PCR tube at room temperature. The reaction mixture was then shaken at 25 °C for 2 h in a block bath shaker (1000 rpm). After this time, carboxylic acid 7 or 16^23,24^ (40 μmol) and isocyanide (40 μmol) were added to the solution at room temperature and the reaction mixture was shaken at 25 °C for 20 h in a block bath shaker. Subsequently, DMF (60 μL) was added to the reaction solution to obtain a final crude peptoid concentration of 250 mM. An aliquot of the solution of 250 mM crude peptoids in MeOH/DMF (8.0 μL, 1000 nmol) and DIPEA (0.5 μL) were then added to a 10 mM aqueous DNA solution (DNA 1–3, 2.0 μL, 20 nmol) in a PCR tube, and the reaction mixture was shaken at 50 °C for 20 h in a block bath shaker (1000 rpm). To precipitate the oligonucleotide, 3 M sodium acetate solution (2 μL) and ethanol (50 μL) were added to the solution. After centrifugation, the supernatant was removed, and the resulting pellet was dissolved in 0.1 M aqueous triethylammonium acetate (TEAA) buffer (pH = 7.0, 200 μL). The crude solution was purified by reversed-phase HPLC (Waters XBridge® Oligonucleotide BEH C18 OBD™ Prep Column, 130 Å, 2.5 μm, 10 mm × 50 mm) using 0.1 M TEAA buffer (pH = 7.0) as eluent A, and MeCN as eluent B. A linear gradient from 7 to 40% MeCN (over 30 min) was used at 50 °C at a flow rate of 4 mL min^−1^ and the process was monitored by UV visualization at 260 nm. The compositions of oligonucleotide-tagged peptoids were confirmed by MALDI-TOF MS analysis, and the yields were calculated from the peak values recorded at 260 nm on a NanoDrop instrument (DeNovix DS-11).

### Urea bond formation using crude peptoids prepared by the Ugi reaction using two amines and two isocyanides

Isobutyraldehyde 17 (50 μmol) and amines 19 and 20 (each 25 μmol) were added to anhydrous MeOH (100 μL) in a PCR tube at room temperature. The reaction mixture was then shaken at 25 °C for 2 h in a block bath shaker (1000 rpm). After this time, carboxylic acid 7 (40 μmol) and isocyanides 10 and 21 (each 20 μmol) were added to the solution at room temperature and the reaction mixture was shaken at 25 °C for 20 h in a block bath shaker. Subsequently, DMF (60 μL) was added to the reaction solution to obtain a final crude peptoid concentration of 250 mM. An aliquot of the solution of 250 mM crude peptoids in MeOH/DMF (16 μL, 2000 nmol) and DIPEA (1.0 μL) were then added to a 10 mM aqueous DNA solution (DNA 2, 4.0 μL, 40 nmol) in a PCR tube, and the reaction mixture was shaken at 50 °C for 20 h in a block bath shaker (1000 rpm). To precipitate the oligonucleotide, 3 M sodium acetate solution (2 μL) and ethanol (50 μL) were added to the solution. After centrifugation, the supernatant was removed, and the resulting pellet was dissolved in 0.1 M aqueous triethylammonium acetate (TEAA) buffer (pH = 7.0, 200 μL). The crude solution was purified by reversed-phase HPLC (Waters XBridge® Oligonucleotide BEH C18 OBD™ Prep Column, 130 Å, 2.5 μm, 10 mm × 50 mm) using 0.1 M TEAA buffer (pH = 7.0) as eluent A, and MeCN as eluent B. A linear gradient from 7 to 40% MeCN (over 30 min) was used at 50 °C at a flow rate of 4 mL min^−1^ and the process was monitored by UV visualization at 260 nm. The compositions of oligonucleotide-tagged peptoids were confirmed by MALDI-TOF MS analysis, and the yields were calculated from the peak values recorded at 260 nm on a NanoDrop instrument (DeNovix DS-11).

### General procedure of peptoid substitution *via* the Suzuki cross-coupling reaction

Pd(OAc)_2_ (4.4 mg, 20 μmol) and TPPTS (11 mg, 40 μmol) were dissolved in H_2_O (10 mL) to prepare a solution containing the 2 mM Pd(OAc)_2_–TPPTS complex. Subsequently, 200 mM boronic acid pinacol ester in DMF (5.0 μL, 1000 nmol), 200 mM Na_2_CO_3_ in H_2_O (10 μL, 2000 nmol), and the 2 mM Pd(OAc)_2_-TPPTS complex in H_2_O (5.0 μL, 10 nmol) were added to the lyophilized DNA (10 nmol) in a PCR tube. The reaction mixture was then shaken at 80 °C for 4 h in a block bath shaker (1000 rpm), then diluted with 0.1 M TEAA (pH 7.0, 100 μL) prior to purification using an NAP-5 column (Cytiva). The obtained crude solution was purified by reversed-phase HPLC (Waters XBridge® Oligonucleotide BEH C18 OBD™ Prep Column, 130 Å, 2.5 μm, 10 mm × 50 mm) using 0.1 M TEAA buffer (pH = 7.0) as eluent A, and MeCN as eluent B. A linear gradient from 7 to 40% MeCN (over 30 min) was used at 50 °C at a flow rate of 4 mL min^−1^ and the process was monitored by UV visualization at 260 nm. The product composition was confirmed by MALDI-TOF MS analysis, and the yields were calculated from the peak values recorded at 260 nm on a NanoDrop instrument (DeNovix DS-11).

### General procedure for methyl ester hydrolysis

A 100 mM solution of NaOH in H_2_O (50 μL) was added to the lyophilized DNA (10 nmol) in a PCR tube, and the reaction mixture was shaken at 37 °C for 4 h in a block bath shaker (1000 rpm). After this time, the reaction mixture was diluted with 2 M TEAA (pH 7.0, 100 μL), and the resulting mixture was purified using a NAP-5 column (Cytiva). The obtained carboxylic acid was used for the subsequent condensation reaction without any purification.

### General procedure for peptoid substitution *via* amide bond formation

200 mM sodium phosphate buffer in H_2_O (10 μL), 250 mM HCl in H_2_O (2.0 μL, 500 nmol), 200 mM amine in MeCN (5.0 μL, 1000 nmol), and 200 mM DMTMM in H_2_O (5.0 μL, 1000 nmol) were added to the lyophilized DNA (10 nmol) in a PCR tube, and the reaction mixture was shaken at 37 °C for 24 h in a block bath shaker (1000 rpm). After this time, 3 M sodium acetate solution (2 μL) and ethanol (50 μL) were added to the solution to precipitate the oligonucleotide. After subsequent centrifugation, the supernatant was removed, and the obtained pellet was dissolved in 0.1 M aqueous triethylammonium acetate (TEAA) buffer (pH = 7.0, 200 μL). The obtained crude solution was purified by reversed-phase HPLC (Waters XBridge® Oligonucleotide BEH C18 OBD™ Prep Column, 130 Å, 2.5 μm, 10 mm × 50 mm) using 0.1 M TEAA buffer (pH = 7.0) as eluent A, and MeCN as eluent B. A linear gradient from 7 to 40% MeCN (over 30 min) was used at 50 °C at a flow rate of 4 mL/min and the process was monitored by UV visualization at 260 nm. The product composition was confirmed by MALDI-TOF MS analysis, and the yields were calculated from the peak values recorded at 260 nm on a NanoDrop instrument (DeNovix DS-11).

## Author contributions

T. O. and S. O. designed the study. R. K. and T. O. performed the DEL synthesis, HPLC analysis, and purification, and wrote the draft version of the manuscript.

## Conflicts of interest

There are no conflicts to declare.

## Supplementary Material

CB-003-D1CB00240F-s001
