# Supplementary material for: Conjugation of oligonucleotides with activated carbamate reagents prepared by the Ugi reaction for oligonucleotide library synthesis
Source: RSC Chem Biol. 2022 Apr 19;3(6):728–38. doi: 10.1039/d1cb00240f (PMC9175101; doi:10.1039/d1cb00240f)

Electronic Supplementary Material (ESI) for RSC Chemical Biology.
This journal is © The Royal Society of Chemistry 2022

Supporting Information

Conjugation of oligonucleotides with activated carbamate reagents prepared by the Ugi reaction for oligonucleotide library synthesis

Ryosuke Kita,[a] Takashi Osawa[a] and Satoshi Obika *[a,b]

[a] *Graduate School of Pharmaceutical Sciences, Osaka University, 1-6 Yamadaoka, Suita, Osaka 565-0871, Japan.*

[b] *National Institutes of Biomedical Innovation, Health and Nutrition, 7-6-8 Saito-Asagi, Ibaraki, Osaka 567-0085, Japan.*

E-mail: obika@phs.osaka-u.ac.jp

Contents:

- **Figure S1.**
  ^{1}H NMR, ^{13}C NMR spectra of each compound .... S2−S9
- **Figure S2.**
  HPLC chromatograms after the conjugation reaction between peptoids **11–14** and **DNA 1** ... S10
- **Figure S3.**
  HPLC chromatograms after the conjugation reaction using DNA with or without an aminohexyl linker and the crude peptoid prepared by Ugi reaction. S10
- **Figure S4.**
  HPLC chromatograms after Suzuki-coupling S11
- **Figure S5.**
  HPLC chromatograms after the amide bond formation reaction. S11
- **Figure S6.**
  HPLC chart and MALDI-TOF mass spectrometry data of each conjugate compound .... S12−S88

Figure S1. ^{1}H NMR, ^{13}C NMR spectra of each compound

(a) ^{1}H NMR ($CDCl_3$) of compound **2**

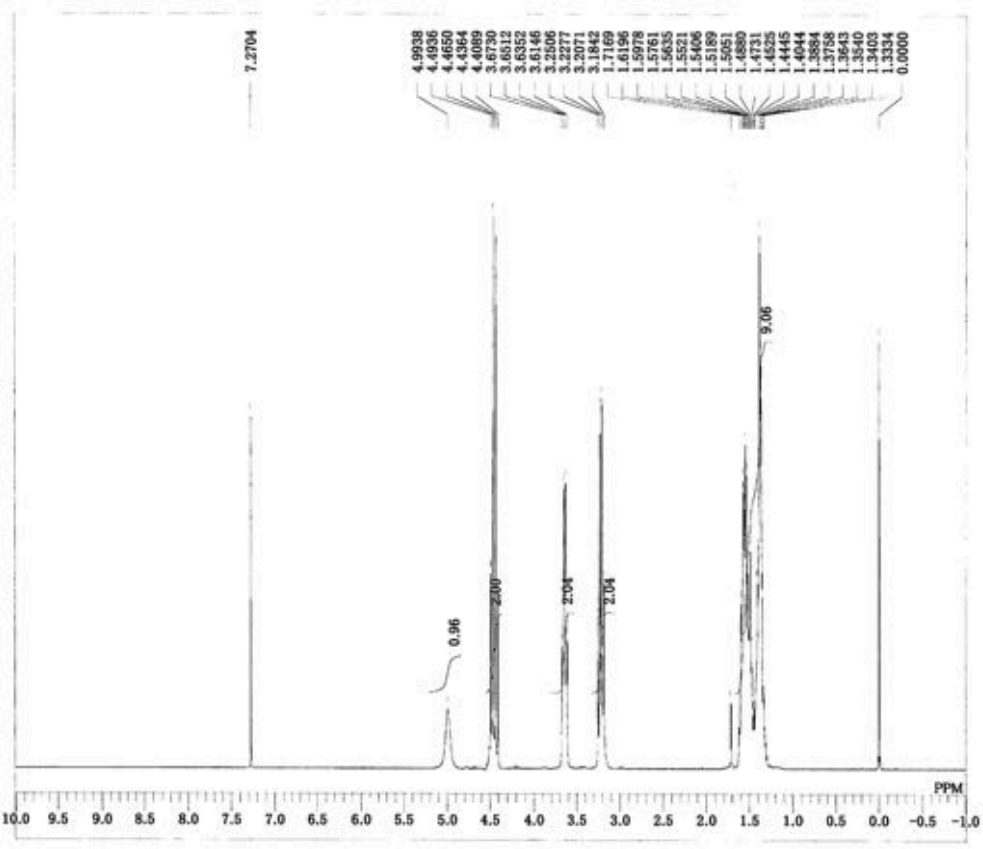

^{13}C NMR ($CDCl_3$) of compound **2**

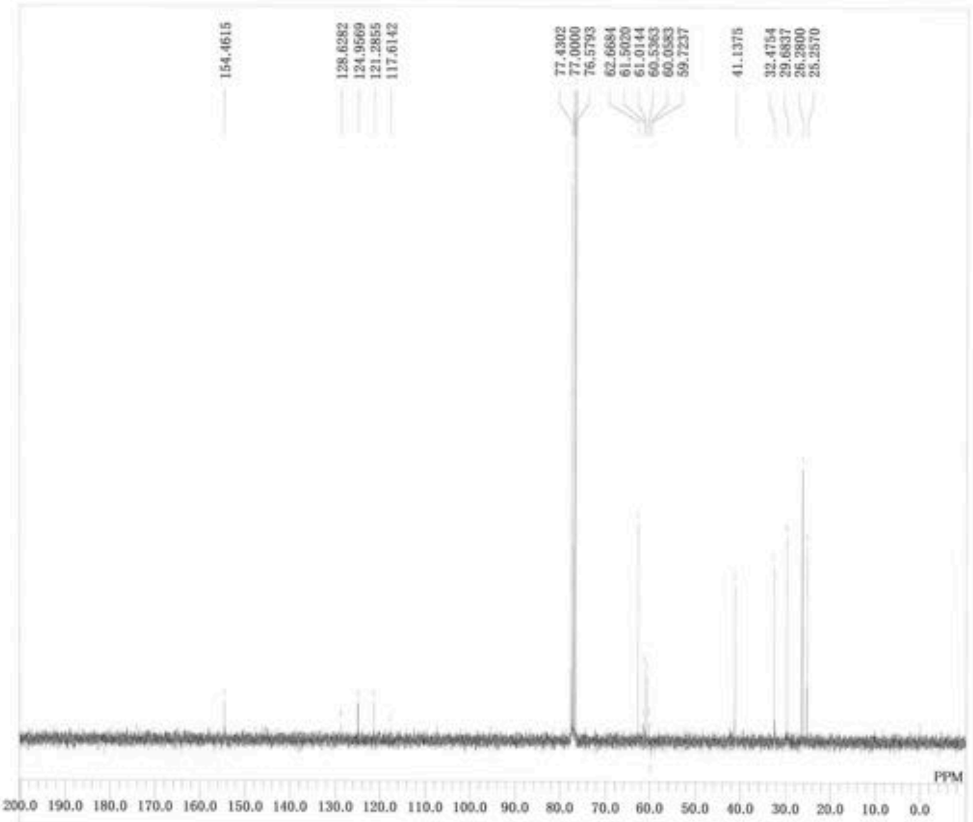

(b) ¹H NMR ($CDCl_3$) of compound **3**

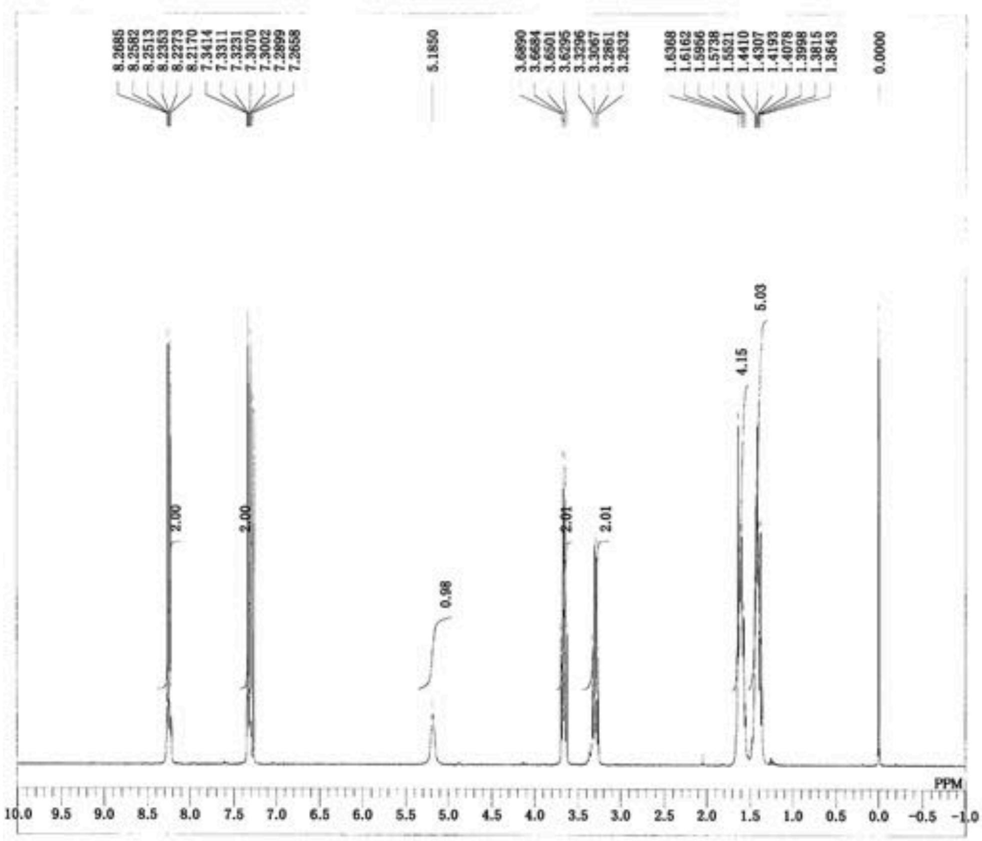

^{13}C NMR ($CDCl_3$) of compound **3**

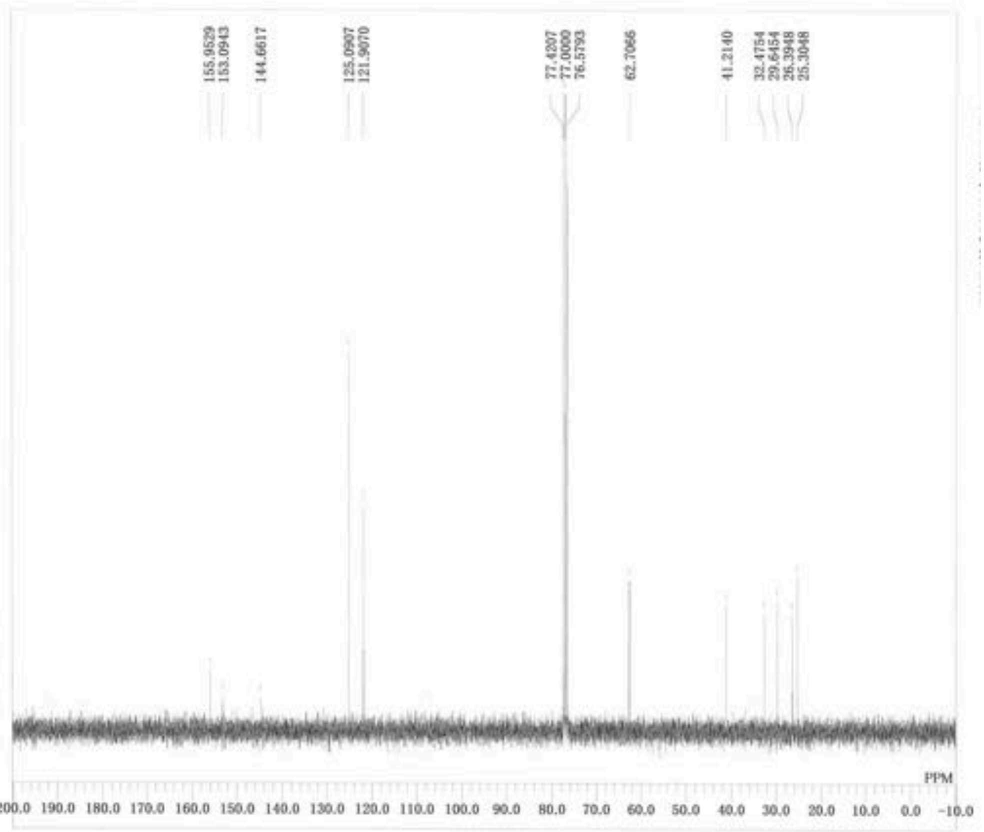

(c) ^{1}H NMR ($CDCl_3$) of compound **5**

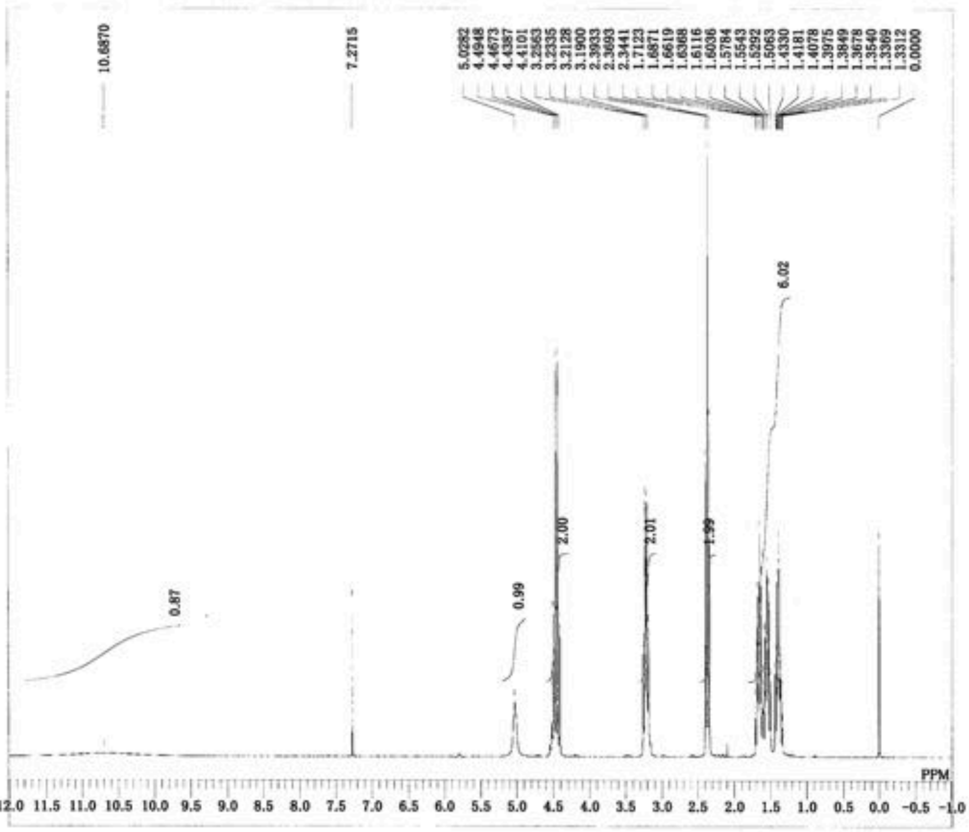

^{13}C NMR ($CDCl_3$) of compound **5**

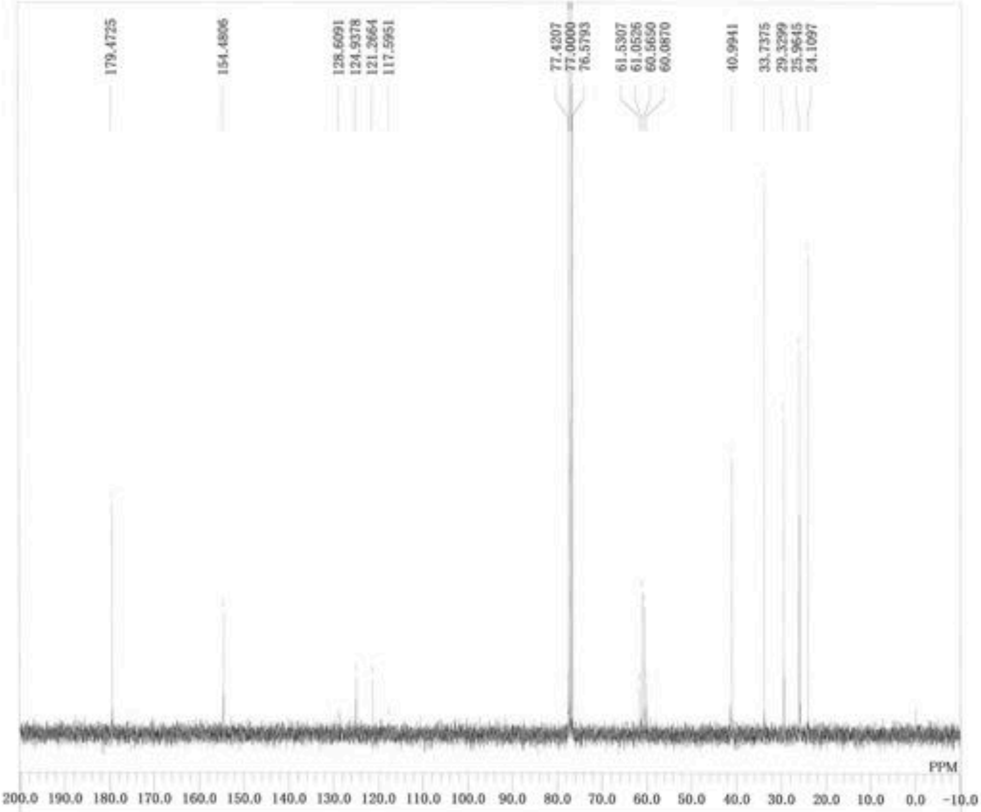

(d) ^{1}H NMR ($CDCl_3$) of compound **7**

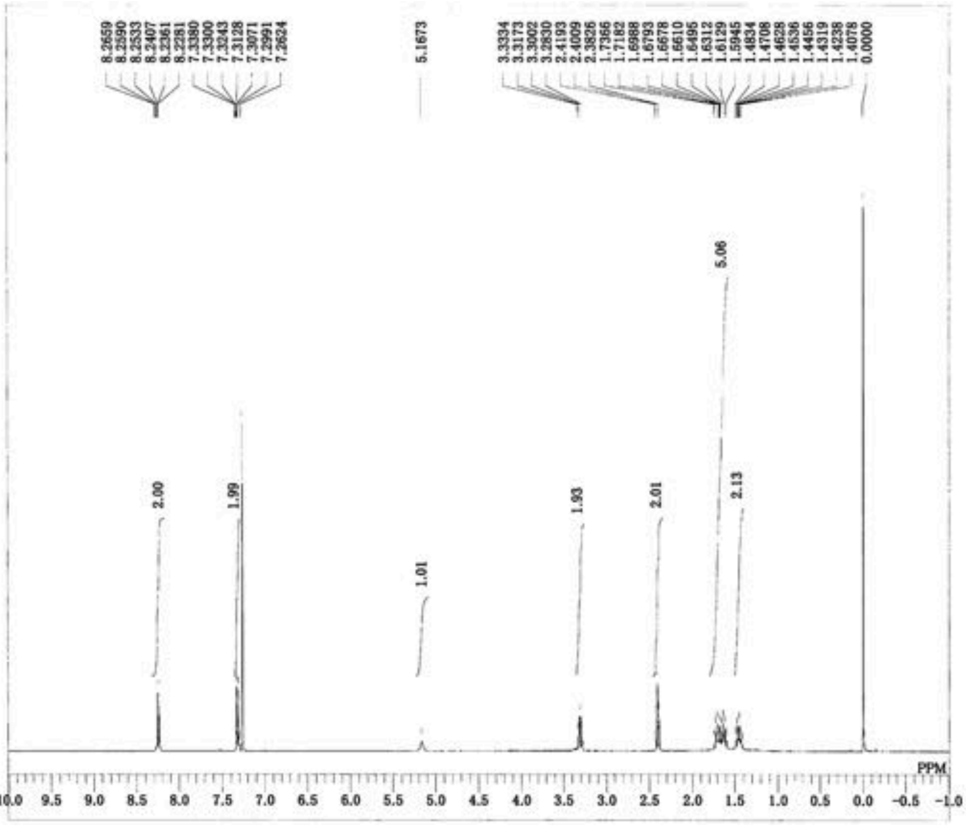

^{13}C NMR (CD_3OD) of compound **7**

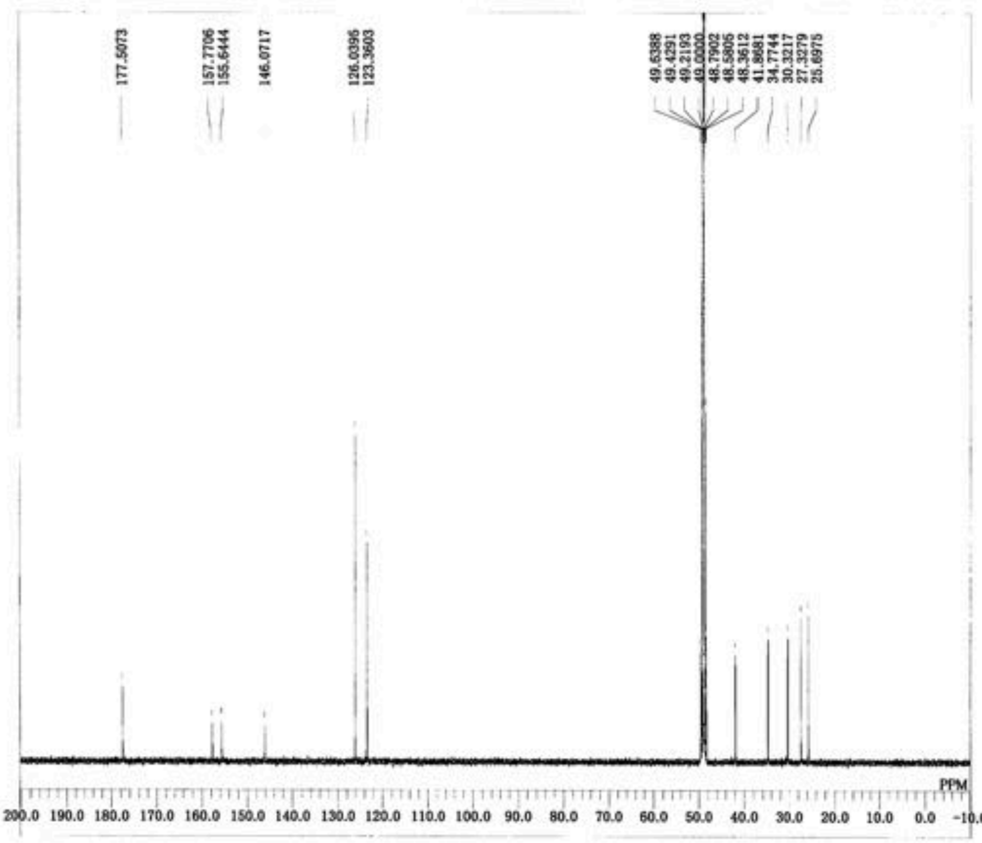

(e) ^{1}H NMR ($CDCl_3$) of compound **11**

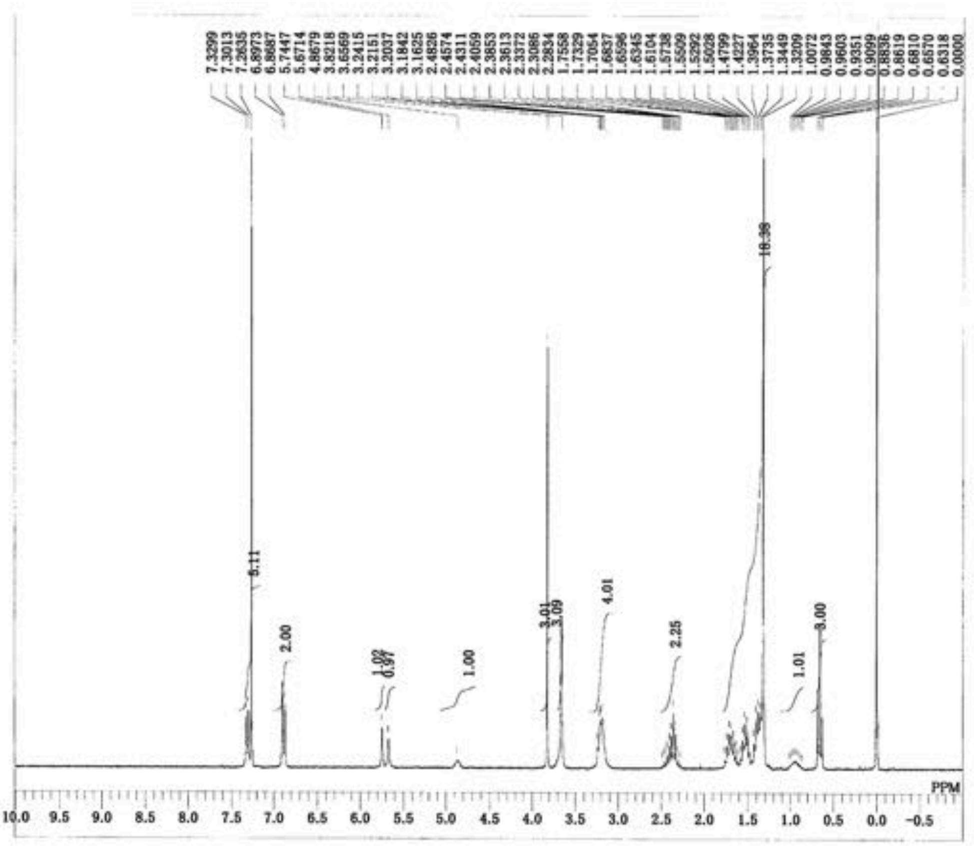

^{13}C NMR ($CDCl_3$) of compound **11**

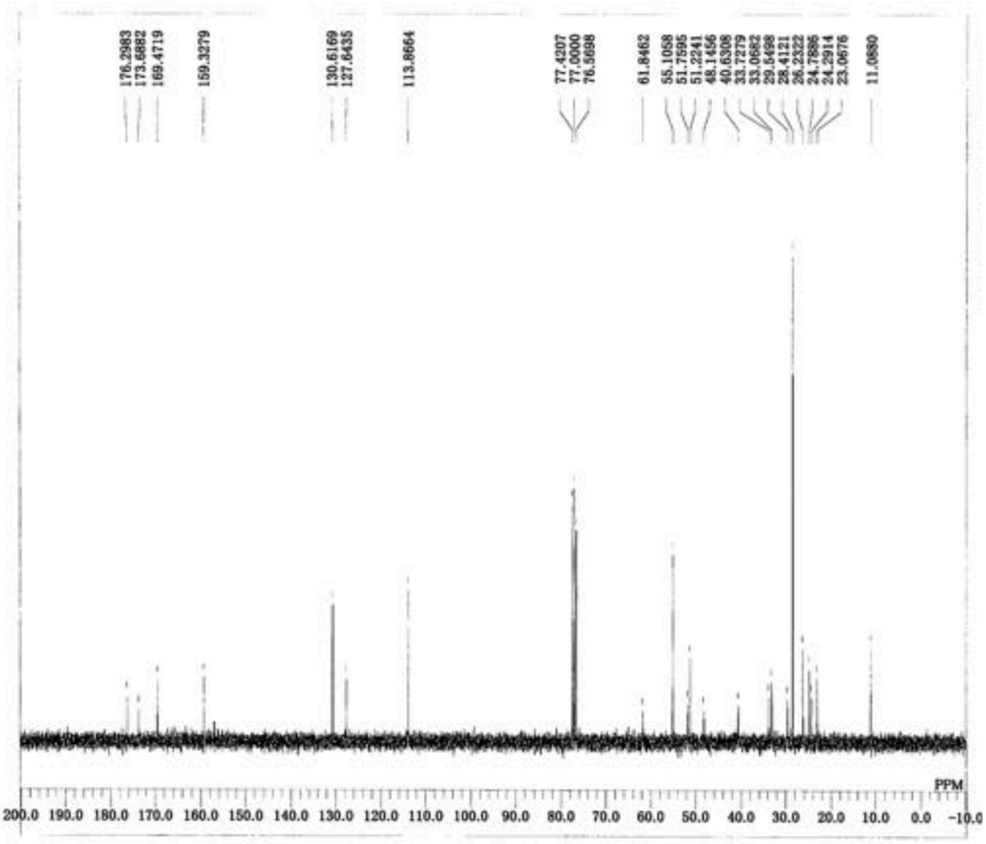

(f) ^{1}H NMR ($CDCl_3$) of compound **12**

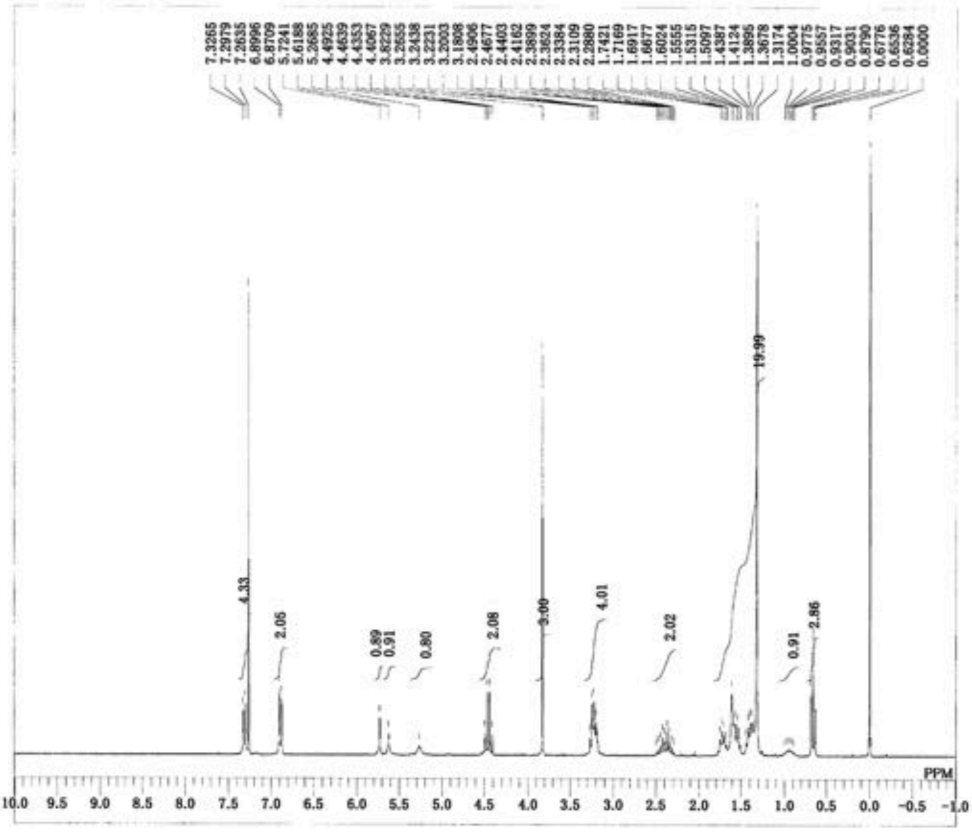

^{13}C NMR ($CDCl_3$) of compound **12**

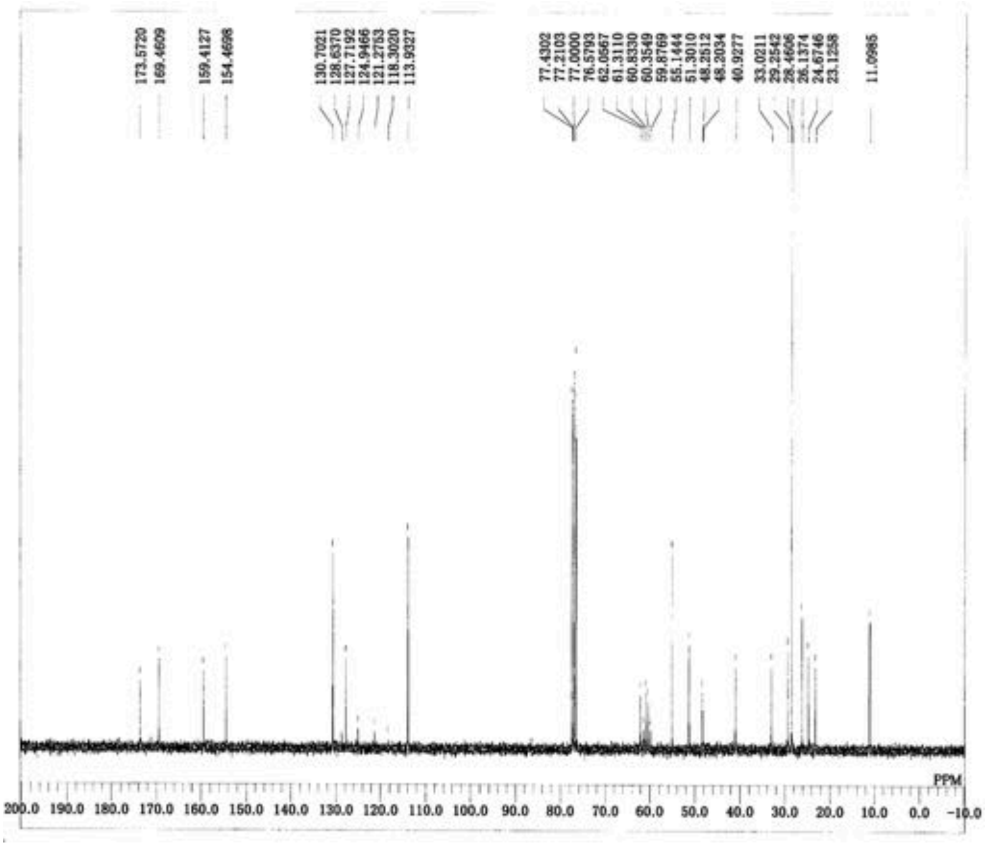

(g) ^{1}H NMR ($CDCl_3$) of compound **13**

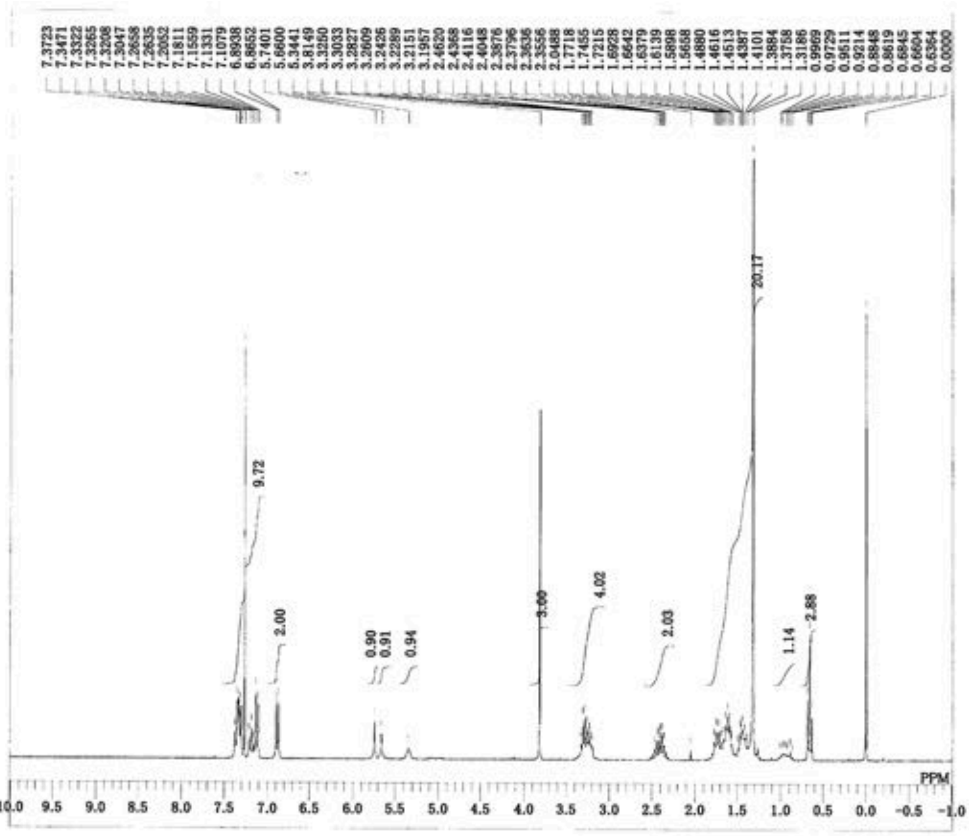

^{13}C NMR ($CDCl_3$) of compound **13**

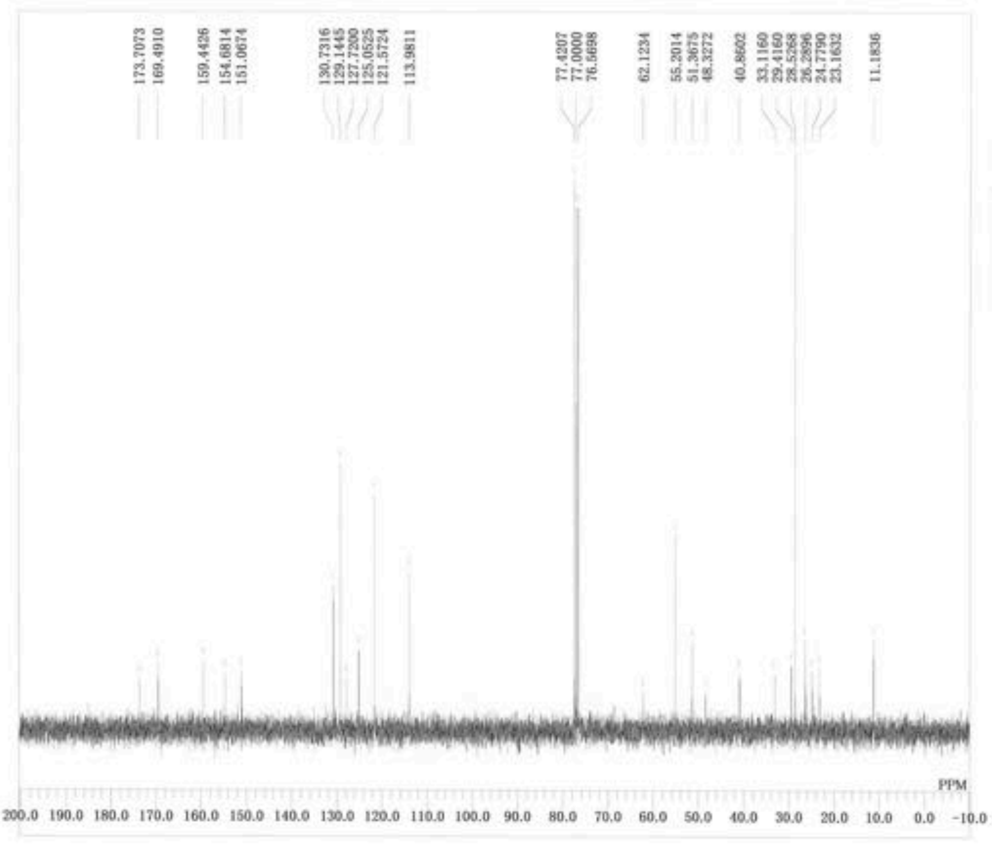

(h) ^{1}H NMR ($CDCl_3$) of compound **14**

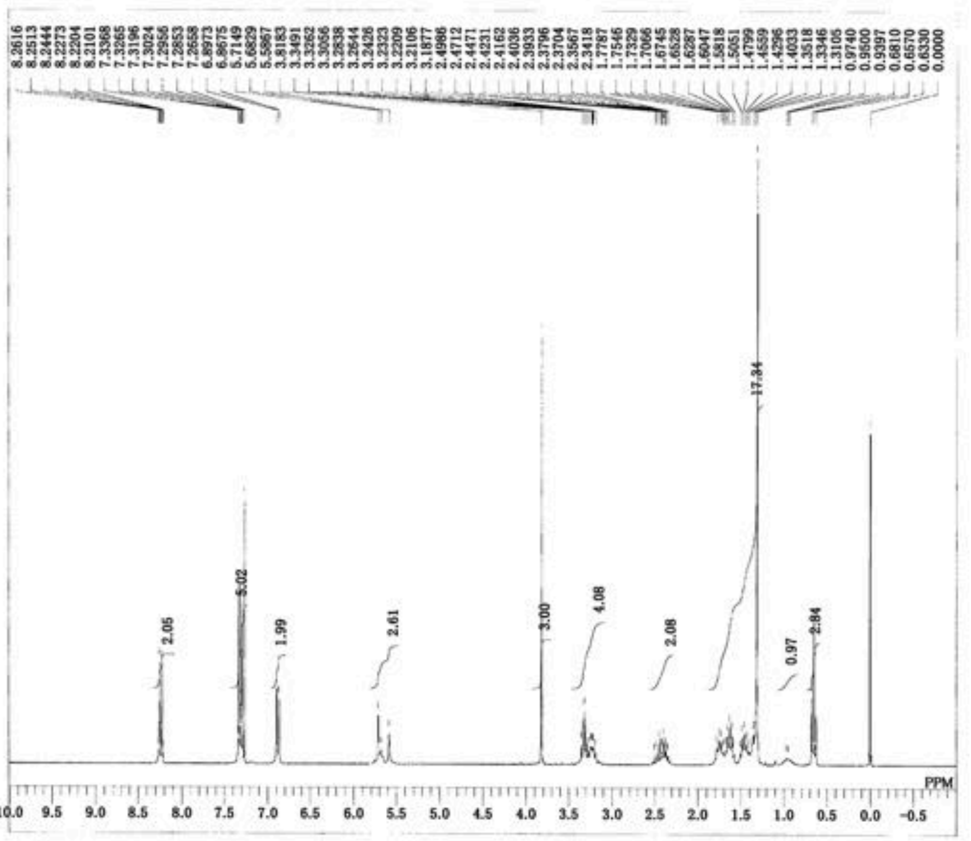

^{13}C NMR ($CDCl_3$) of compound **14**

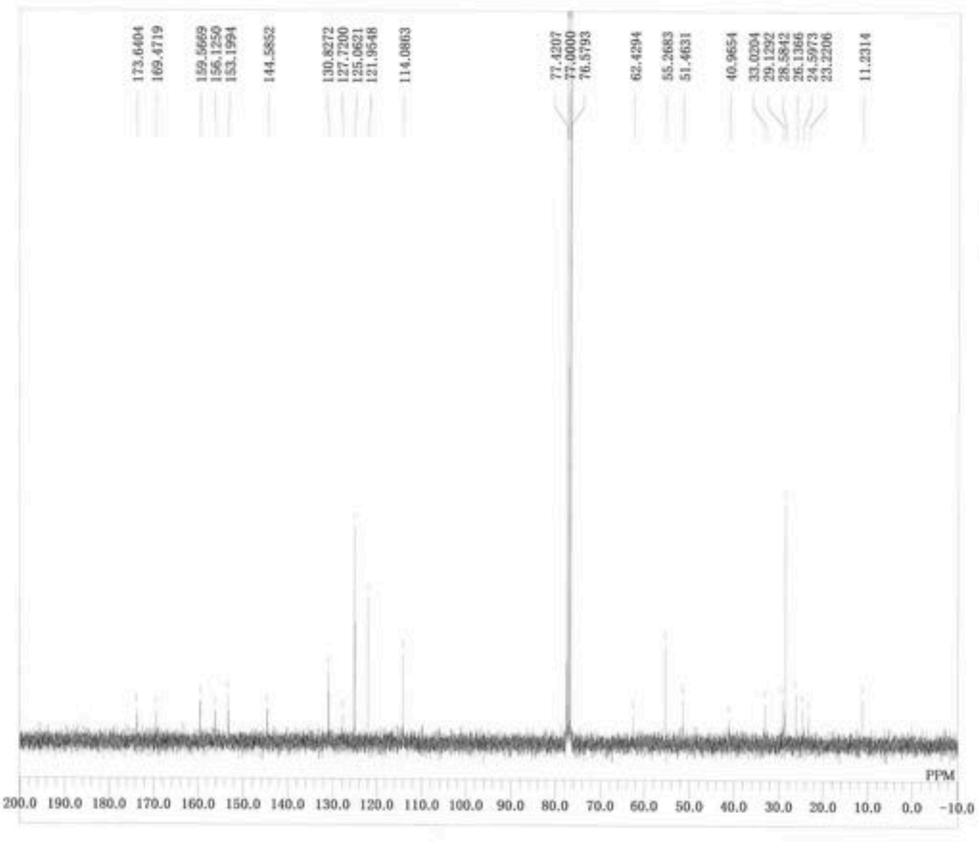

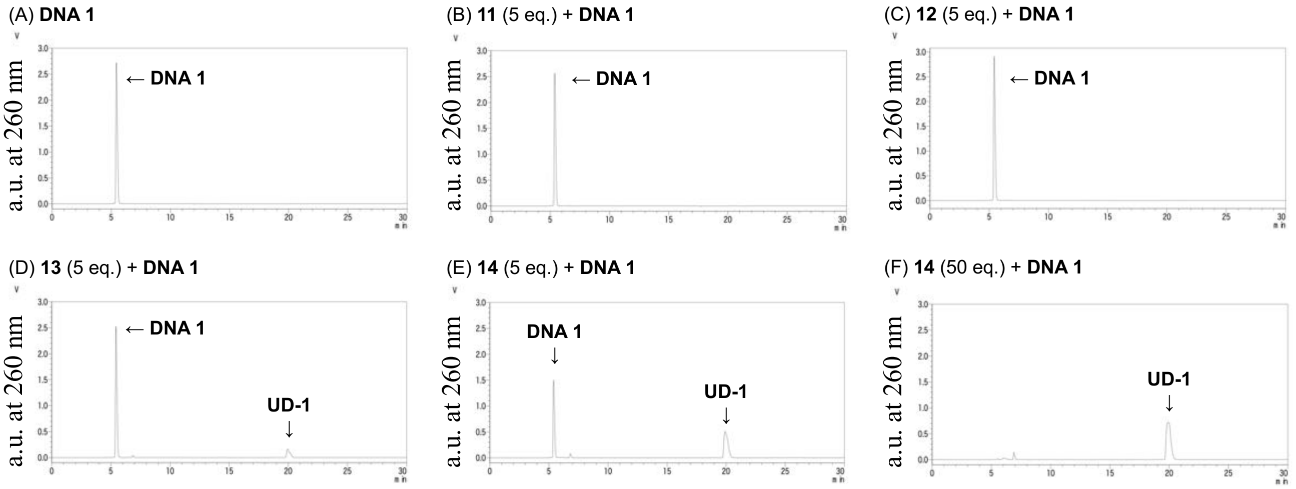

Figure S2. HPLC chromatograms after the conjugation reaction between peptoids **11–14** and **DNA 1**. The reaction mixtures were analyzed by RP-HPLC with a linear gradient of MeCN (from 7 to 40% over 30 min, 50 °C) in 0.1 M TEAA buffer (pH 7.0).

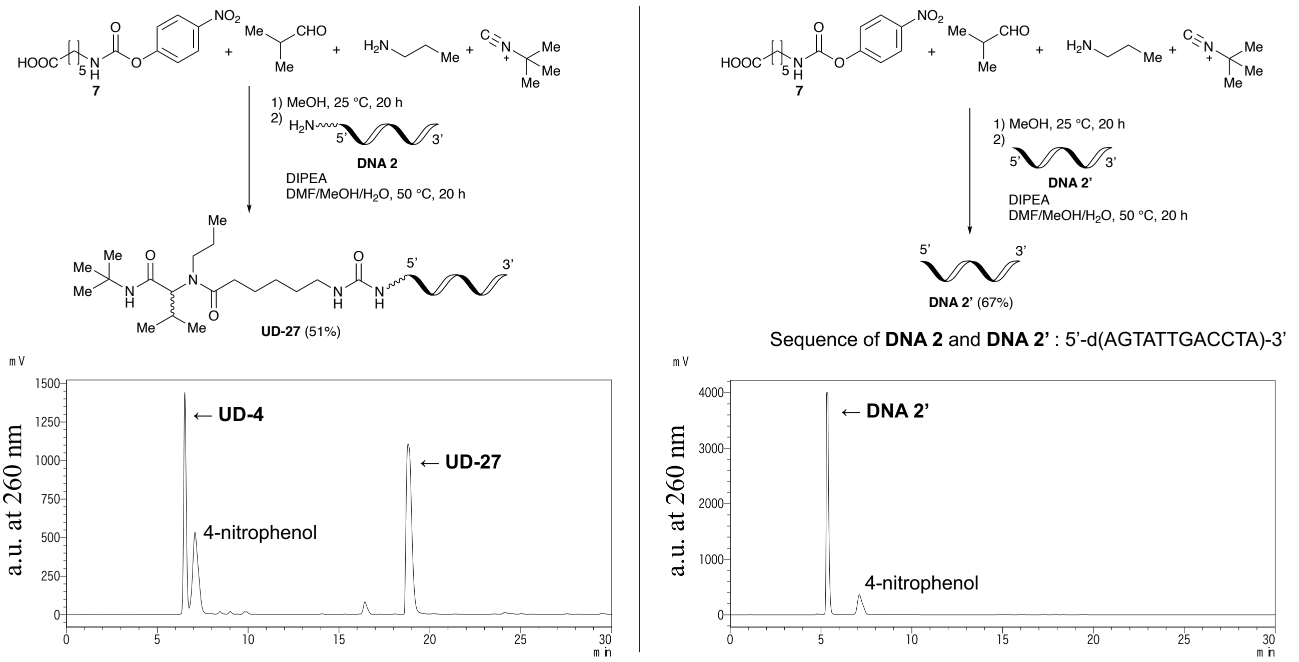

Figure S3. HPLC chromatograms after the conjugation reaction using DNA with or without an aminohexyl linker and the crude peptoid prepared by Ugi reaction. The reaction mixtures were analyzed by RP-HPLC with a linear gradient of MeCN (from 7 to 40% over 30 min, 50 °C) in 0.1 M TEAA buffer (pH 7.0).

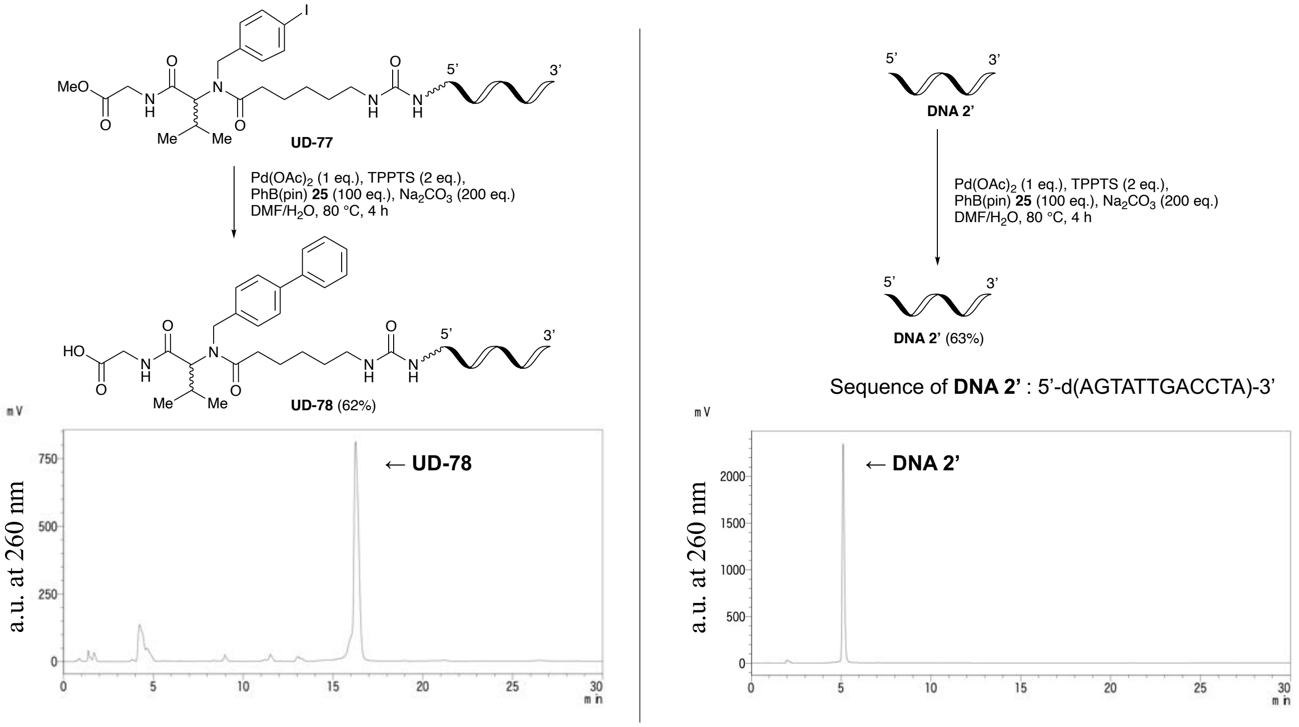

Figure S4. HPLC chromatograms after Suzuki-coupling. The reaction mixtures were analyzed by RP-HPLC with a linear gradient of MeCN (from 7 to 40% over 30 min, 50 °C) in 0.1 M TEAA buffer (pH 7.0).

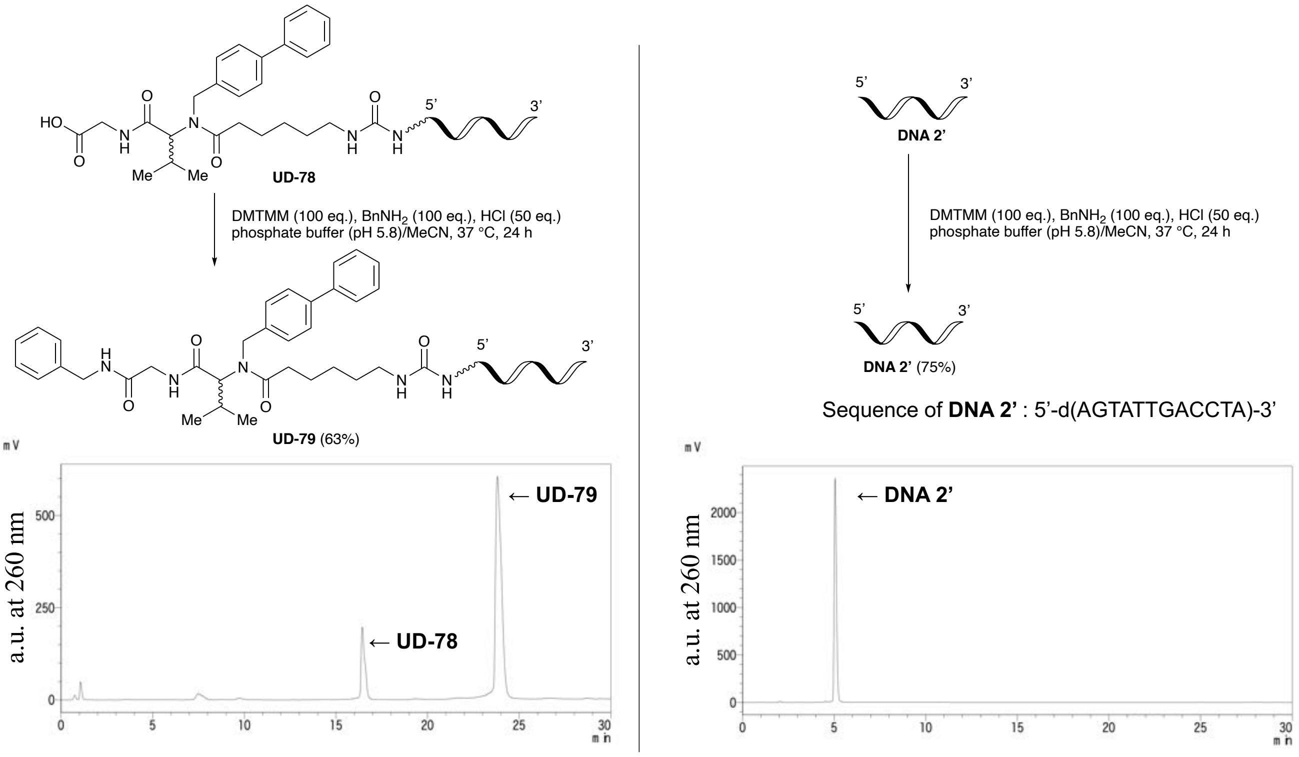

Figure S5. HPLC chromatograms after the amide bond formation reaction. The reaction mixtures were analyzed by RP-HPLC with a linear gradient of MeCN (from 7 to 40% over 30 min, 50 °C) in 0.1 M TEAA buffer (pH 7.0).

Figure S6. HPLC charts and MALDI-TOF mass spectrometry data of each peptoid-conjugated oligonucleotides
HPLC conditions: Detection: UV 260 nm, flow rate: 1.0 mL/min, mobile phase A: 0.1 M TEAA (pH 7.0), mobile phase B: acetonitrile (MeCN), column: Waters XBridge™ Shield RP18 2.5 µm (4.6×50 mm), temperature: 50 °C

UD-1

[B conc. MeCN: 7-40% (30 min)]

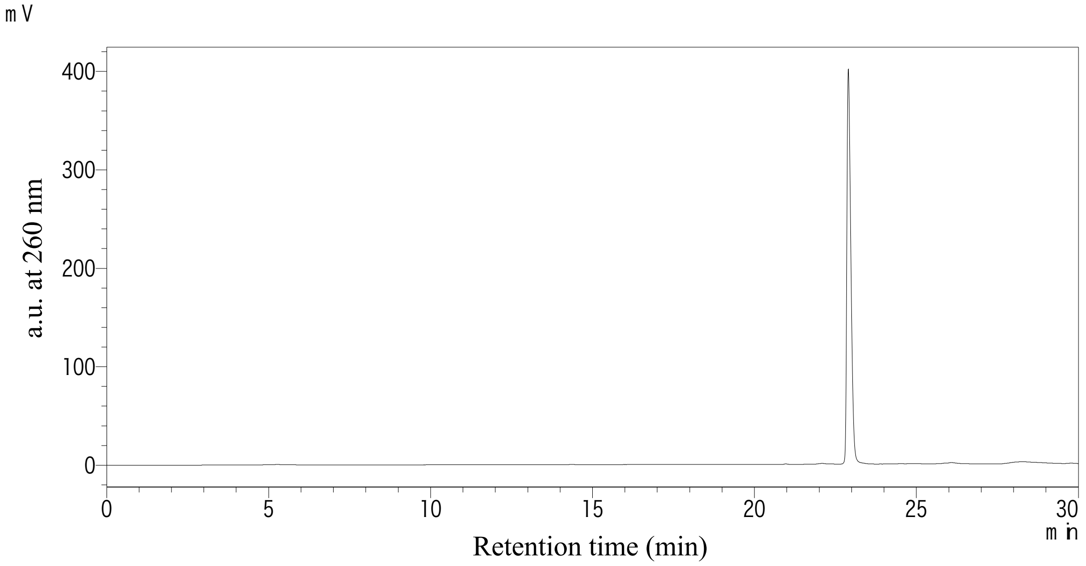

[calcd. $(M\text{-}H)^-$: 3575.7, found: 3578.9]

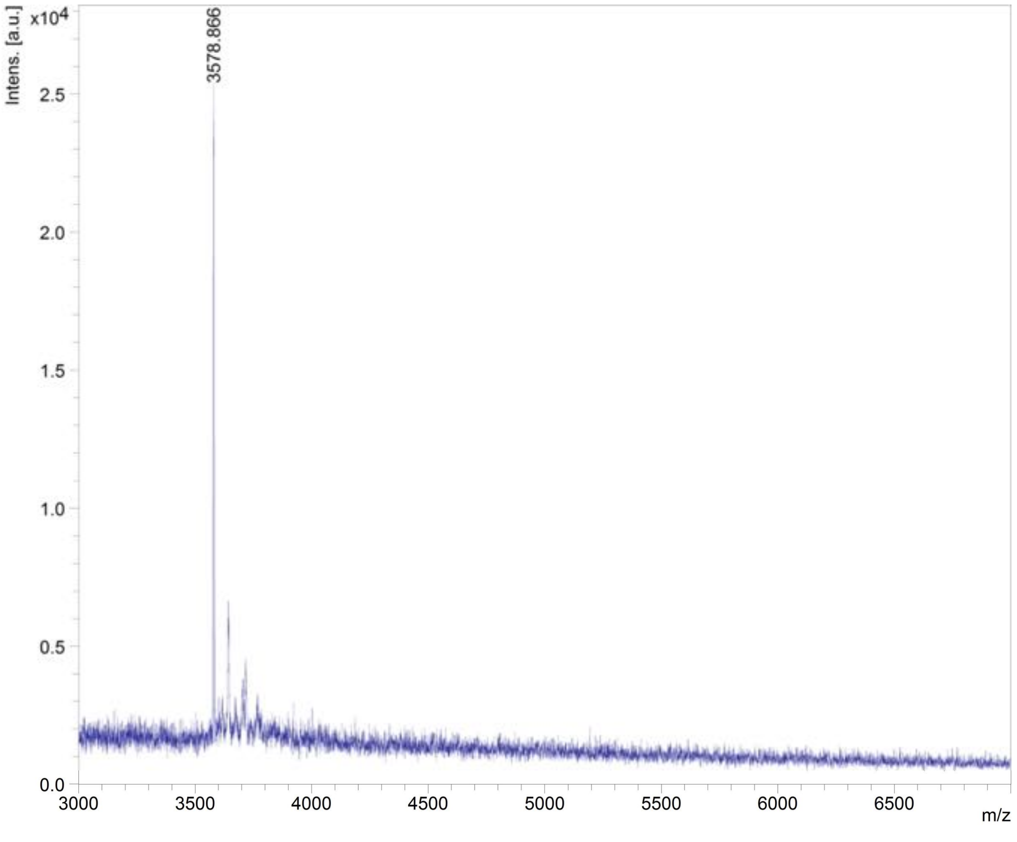

UD-2

[B conc. MeCN: 7-40% (30 min)]

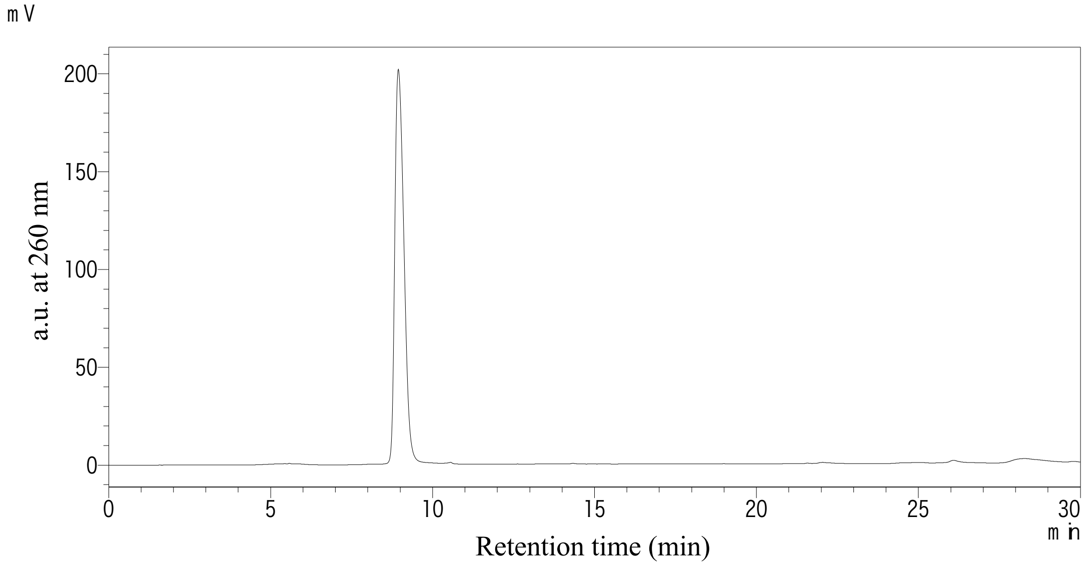

[calcd. (M-H)$^{-}$: 3315.3, found: 3314.4]

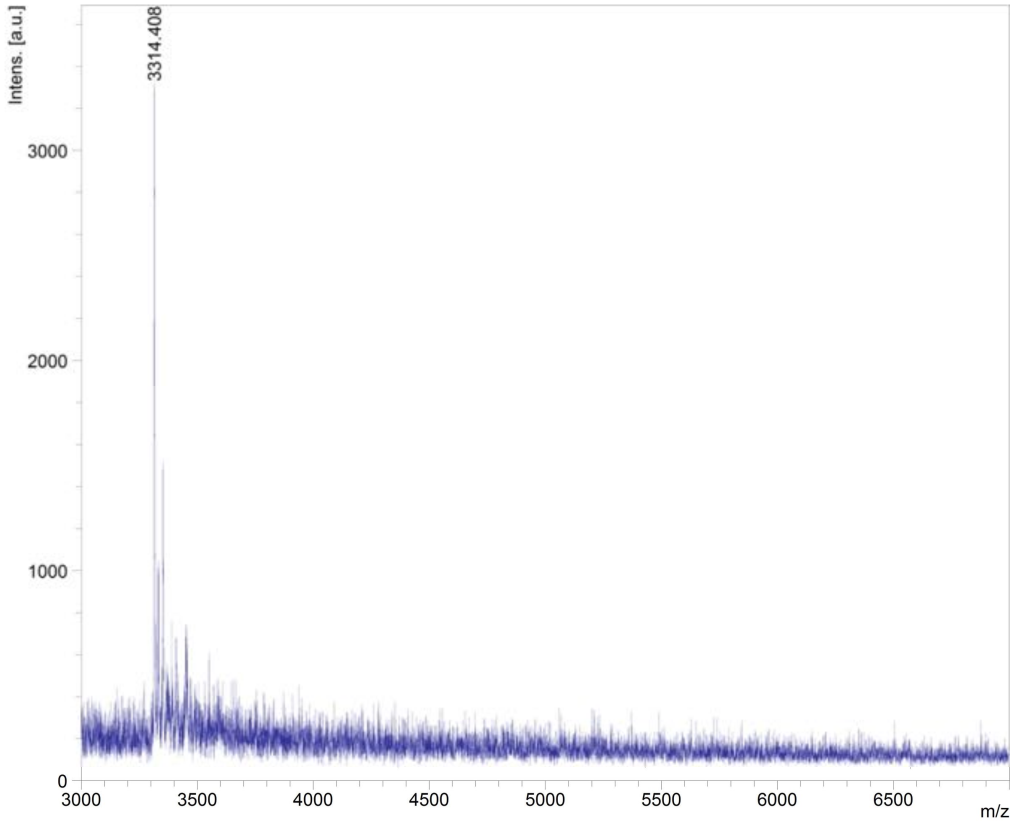

UD-3

[B conc. MeCN: 7-40% (30 min)]

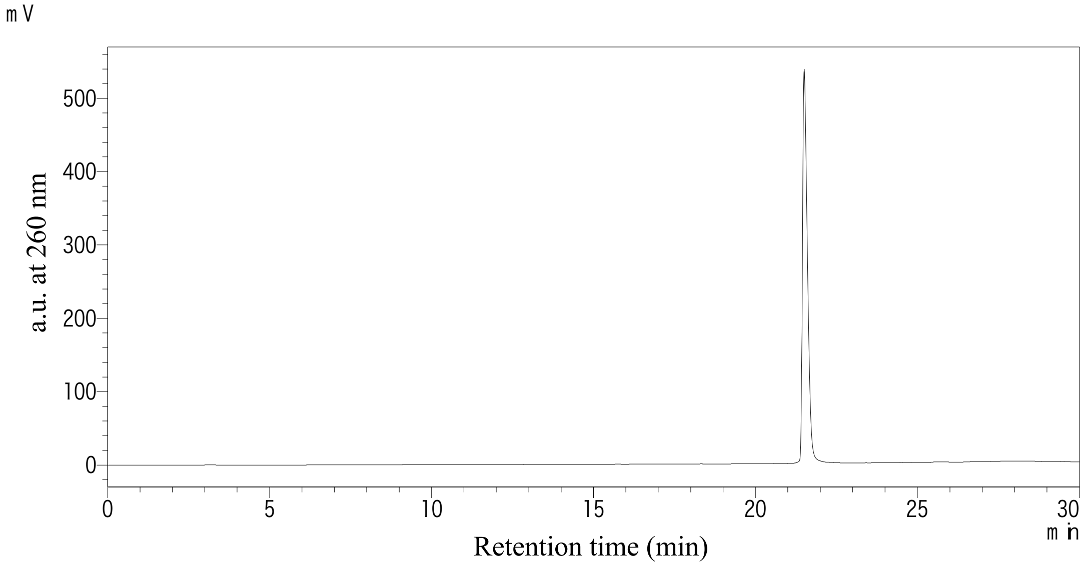

[calcd. (M-H)$^{-}$: 4240.1, found: 4239.8]

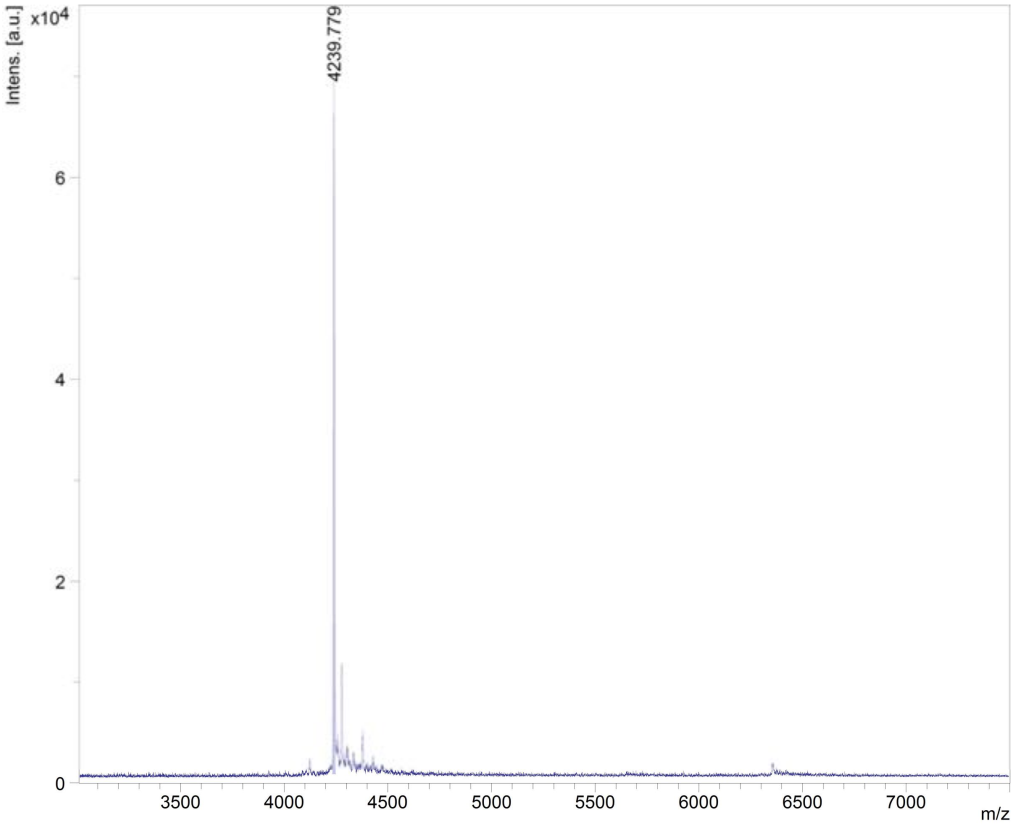

UD-4

[B conc. MeCN: 7-40% (30 min)]

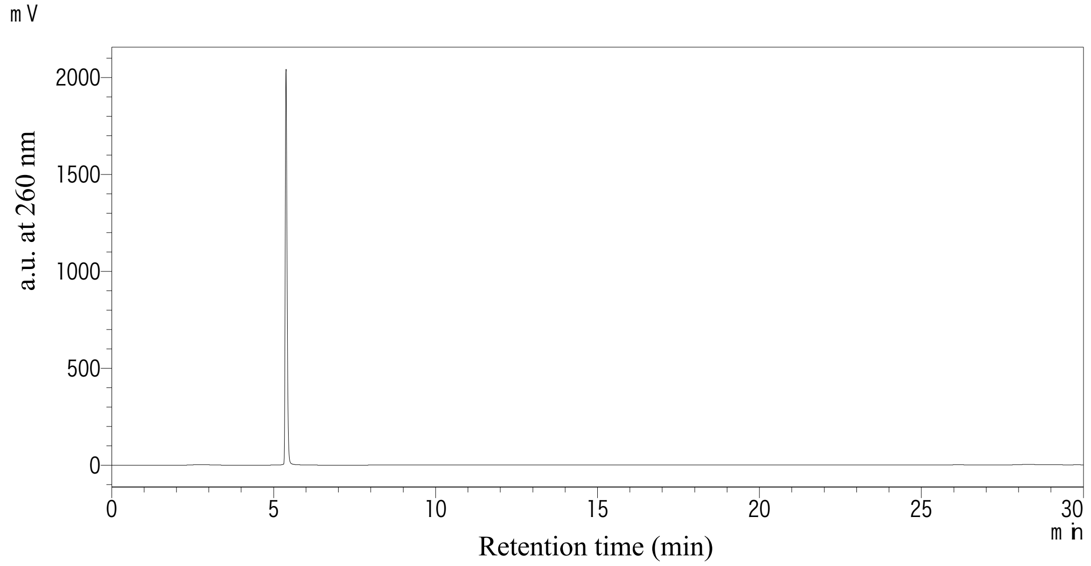

[calcd. (M-H)$^{-}$: 3979.8, found: 3979.5]

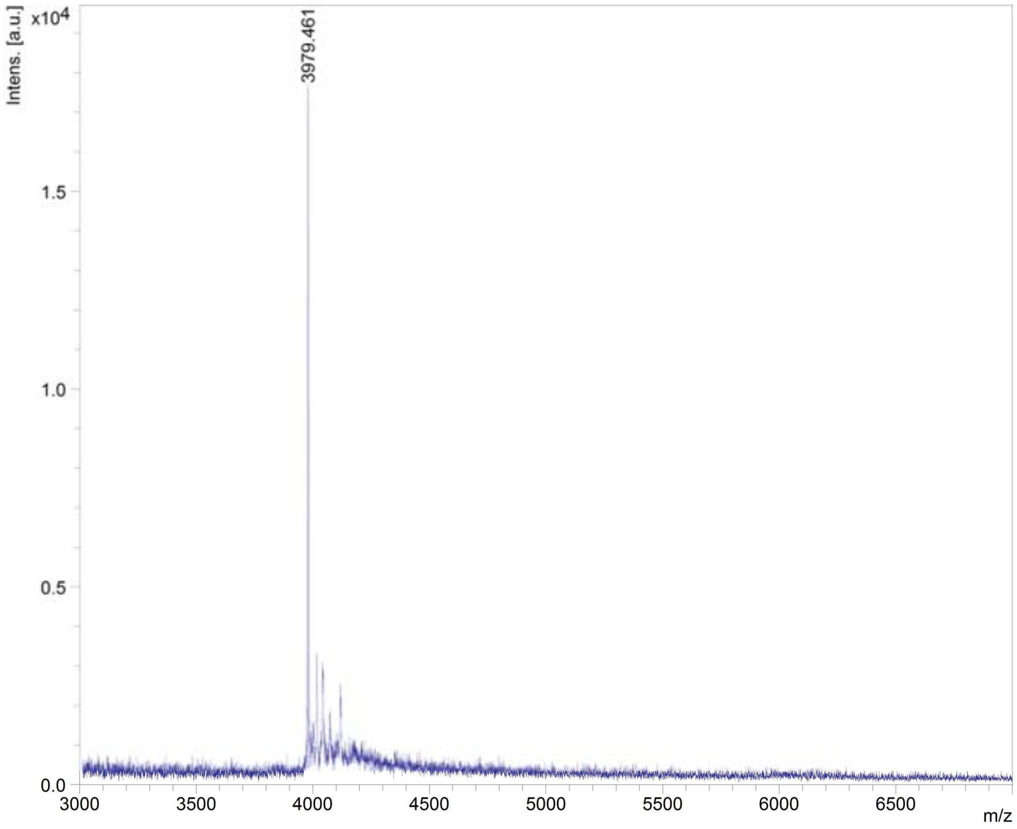

UD-5

[B conc. MeCN: 7-40% (30 min)]

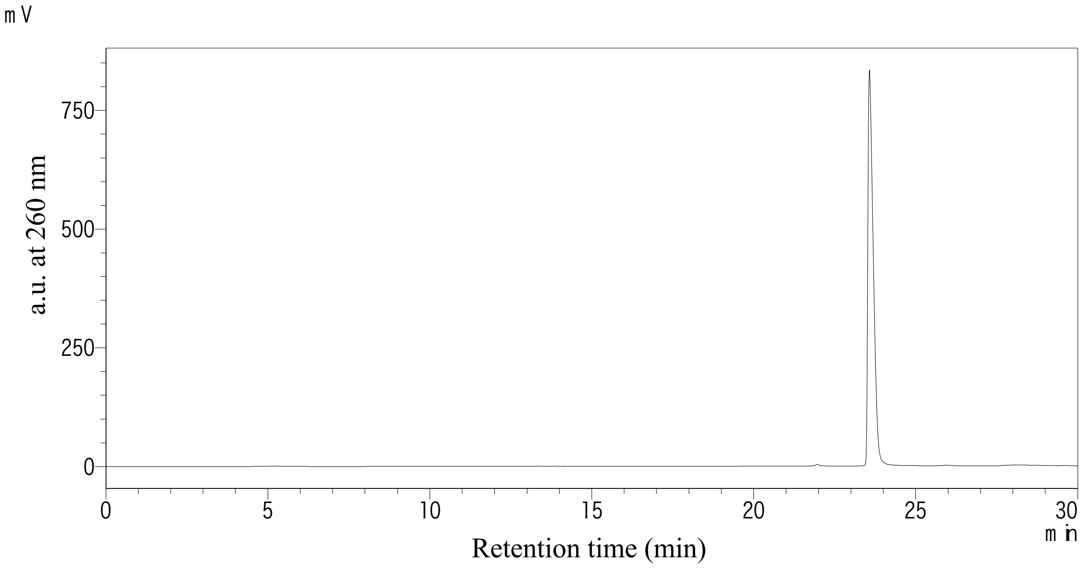

[calcd. $(M-H)^{-}$: 4288.2, found: 4289.1]

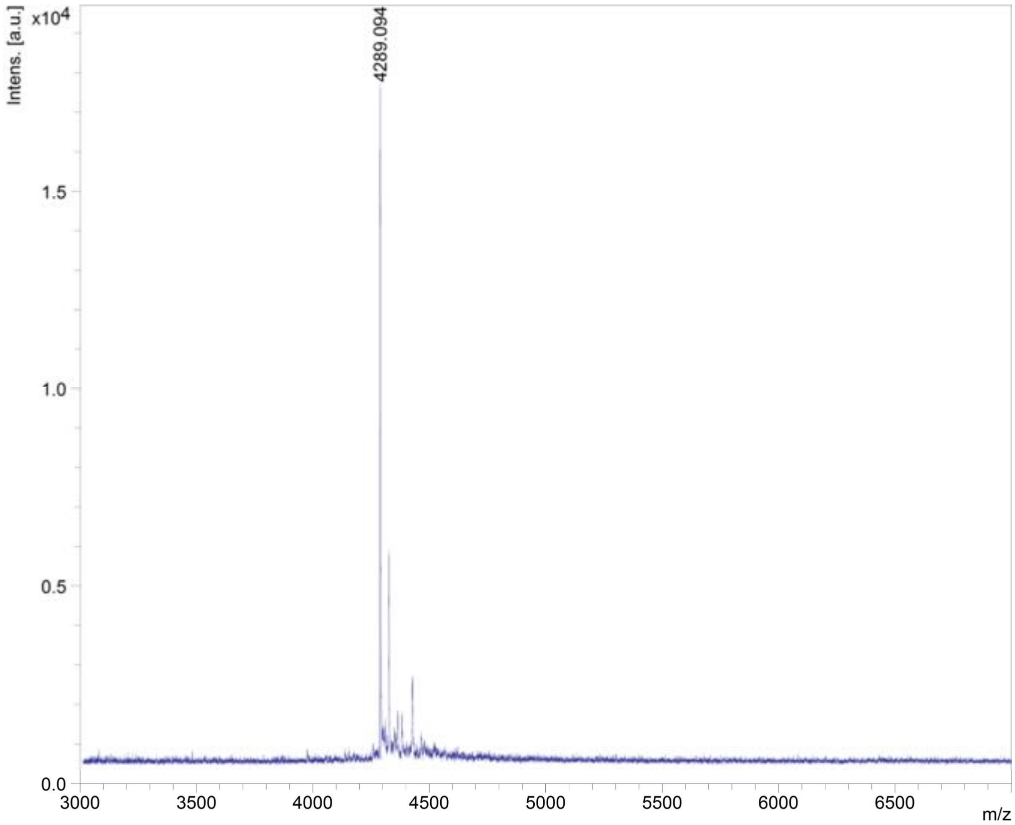

UD-6

[B conc. MeCN: 7-40% (30 min)]

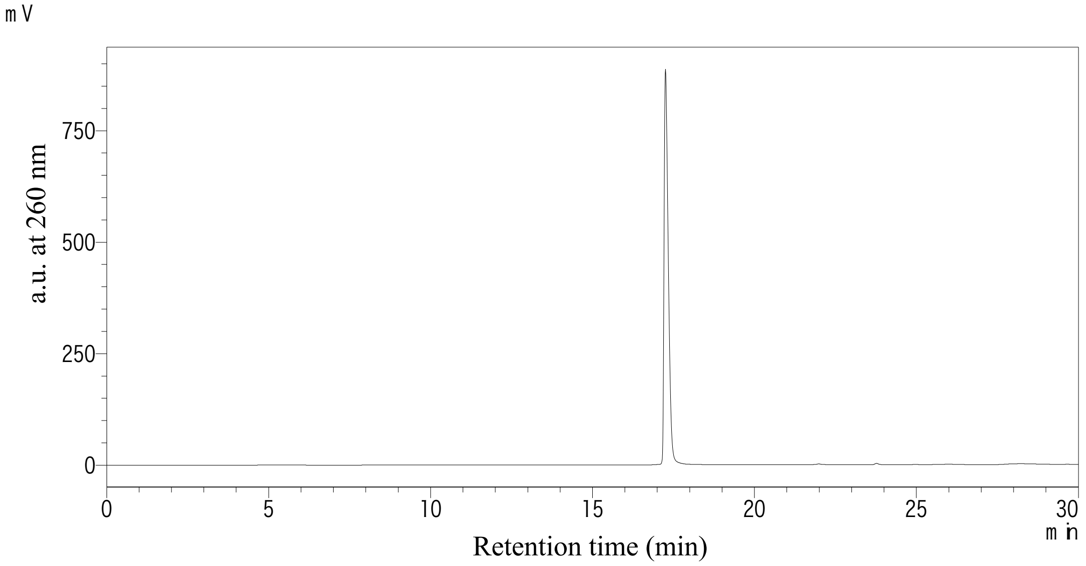

[calcd. (M-H)$^{-}$: 4256.1, found: 4256.6]

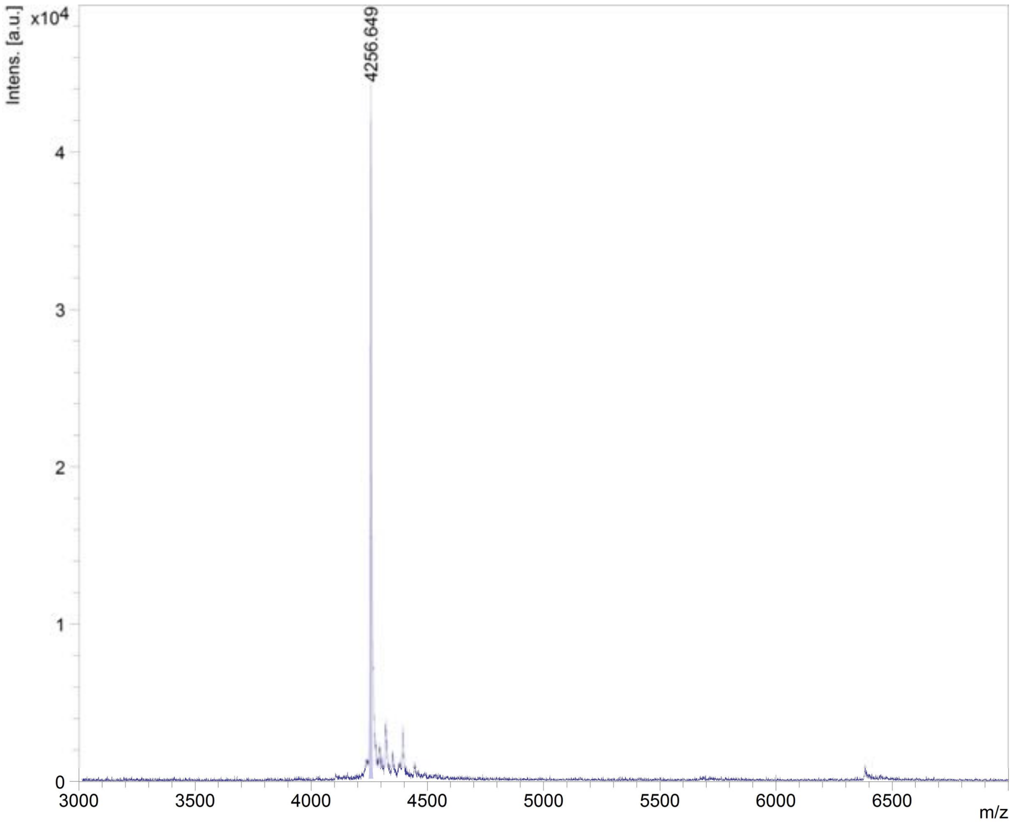

UD-7

[B conc. MeCN: 7-40% (30 min)]

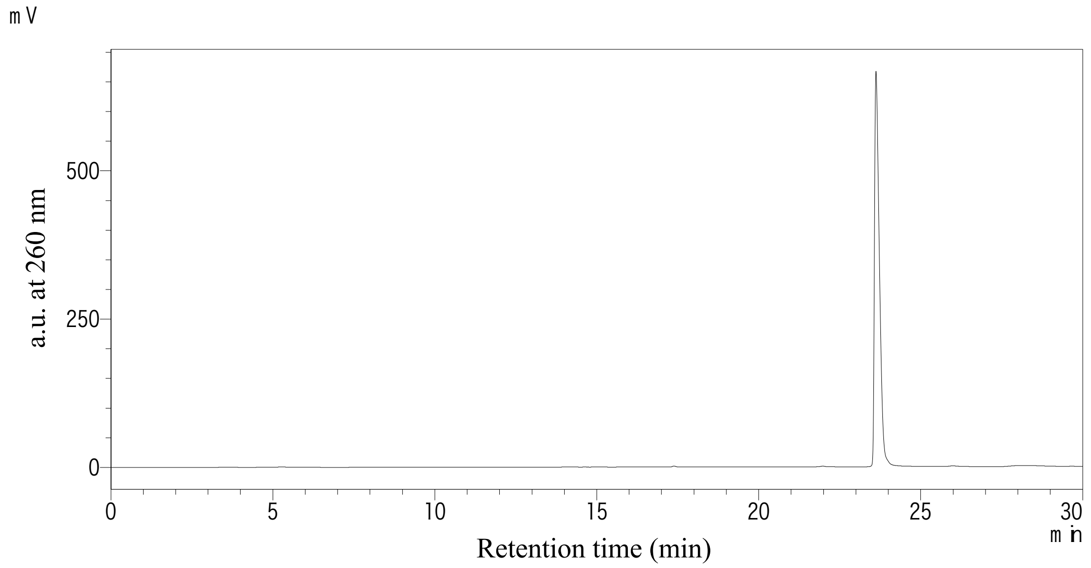

[calcd. (M-H)$^{-}$: 4266.2, found: 4267.5]

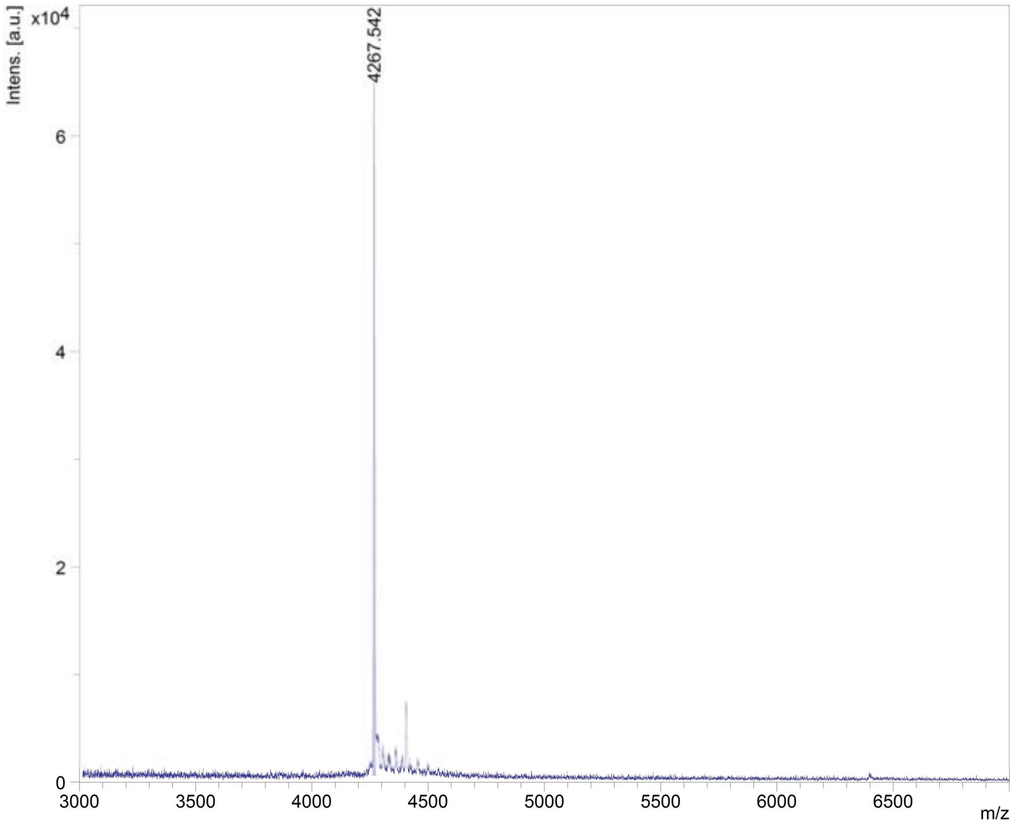

UD-8

[B conc. MeCN: 7-40% (30 min)]

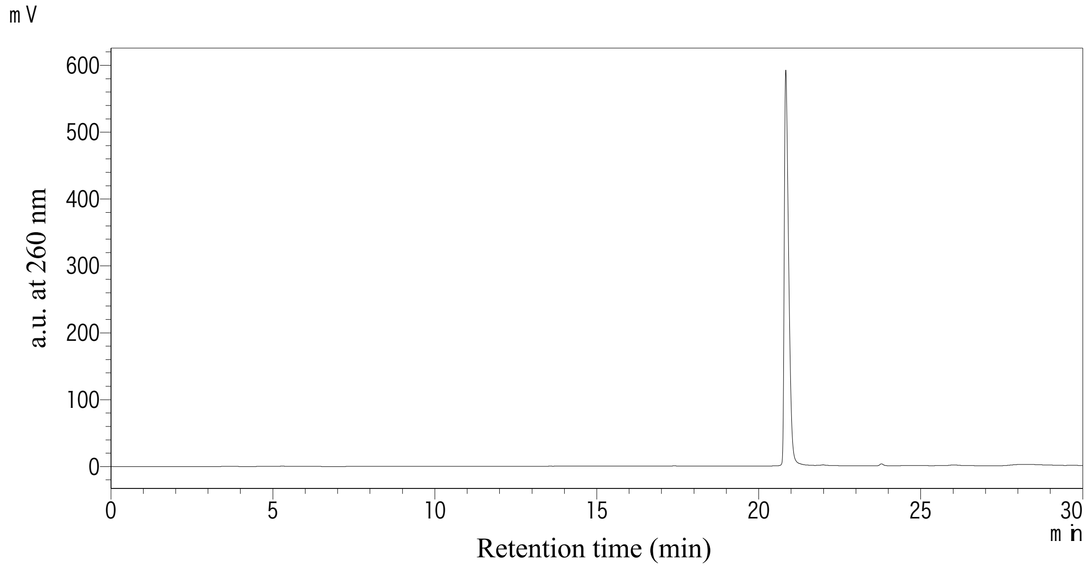

[calcd. (M-H)$^{-}$: 4240.1, found: 4241.4]

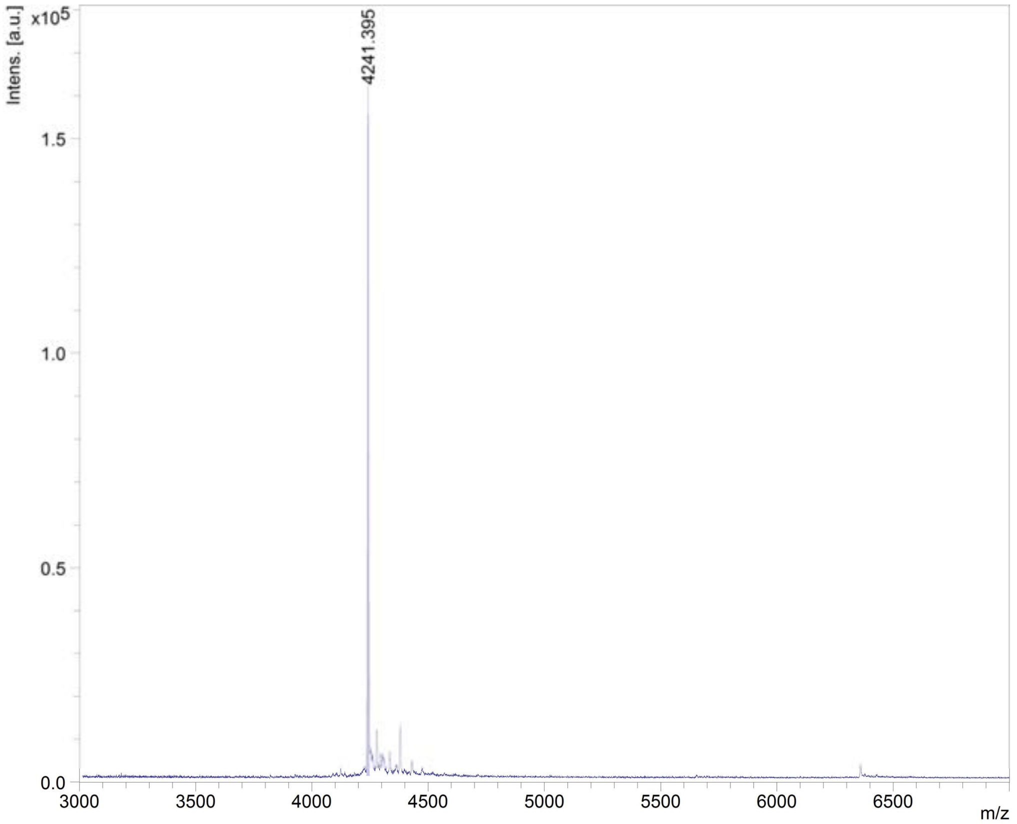

UD-9

[B conc. MeCN: 7-40% (30 min)]

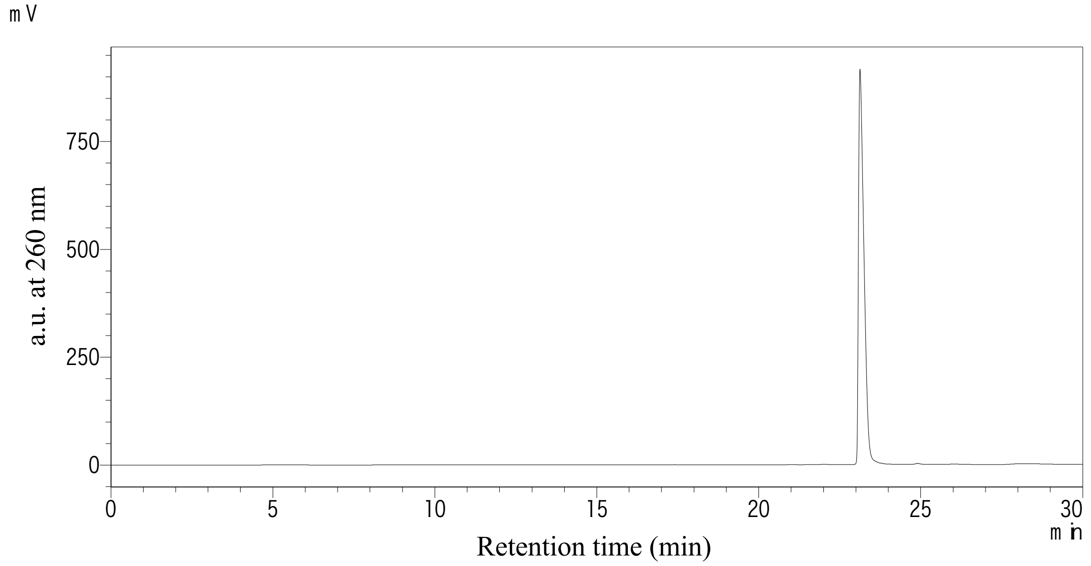

[calcd. (M-H)$^{-}$: 4274.2, found: 4274.8]

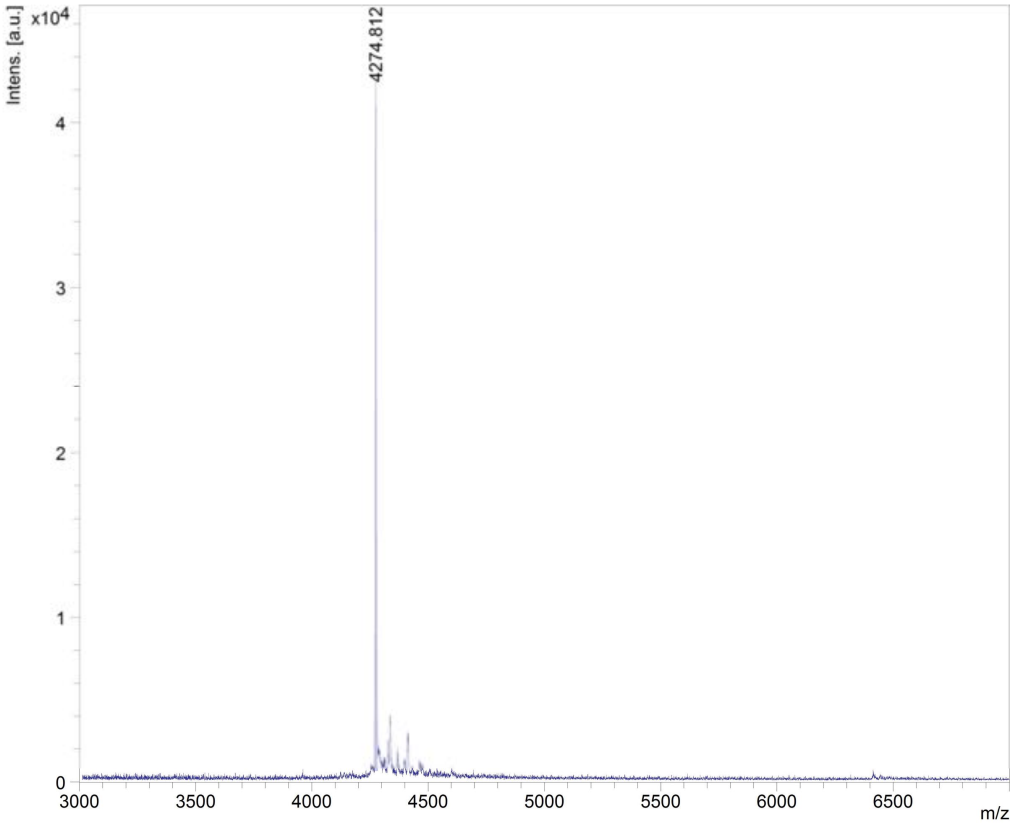

UD-10

[B conc. MeCN: 7-40% (30 min)]

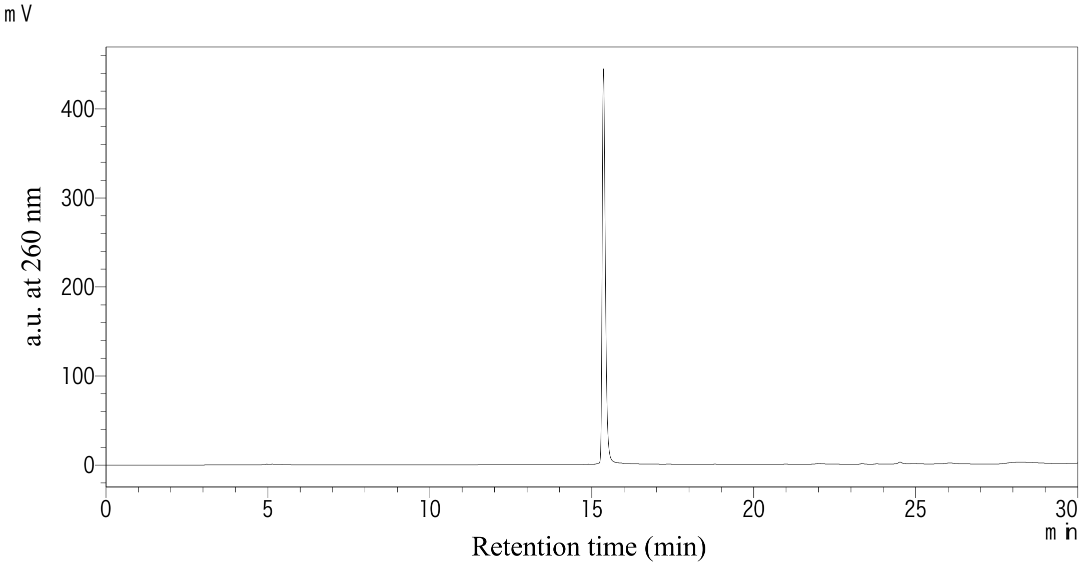

[calcd. (M-H)$^{-}$: 4283.2, found: 4284.4]

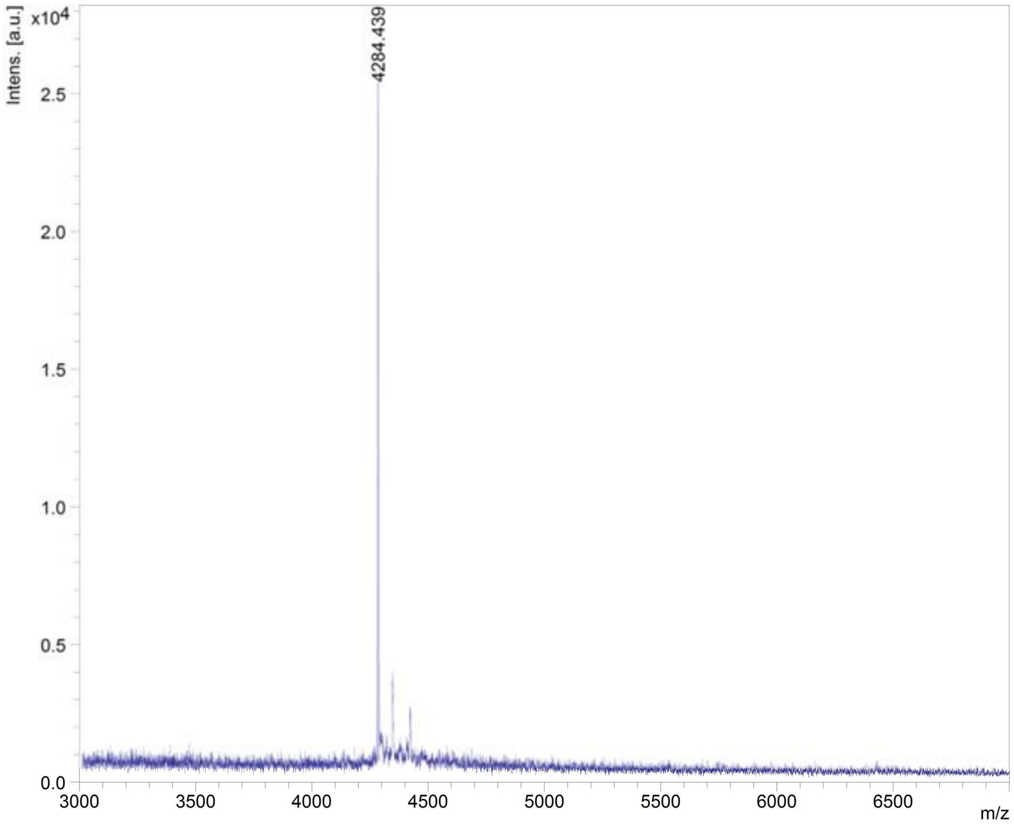

UD-11

[B conc. MeCN: 7-40% (30 min)]

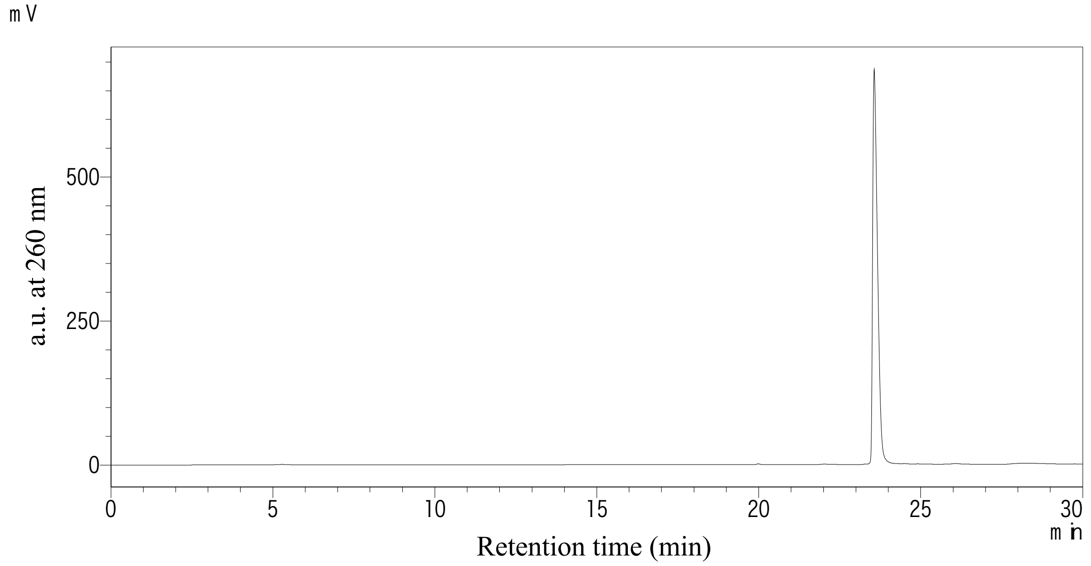

[calcd. (M-H)$^-$: 4266.2, found: 4265.9]

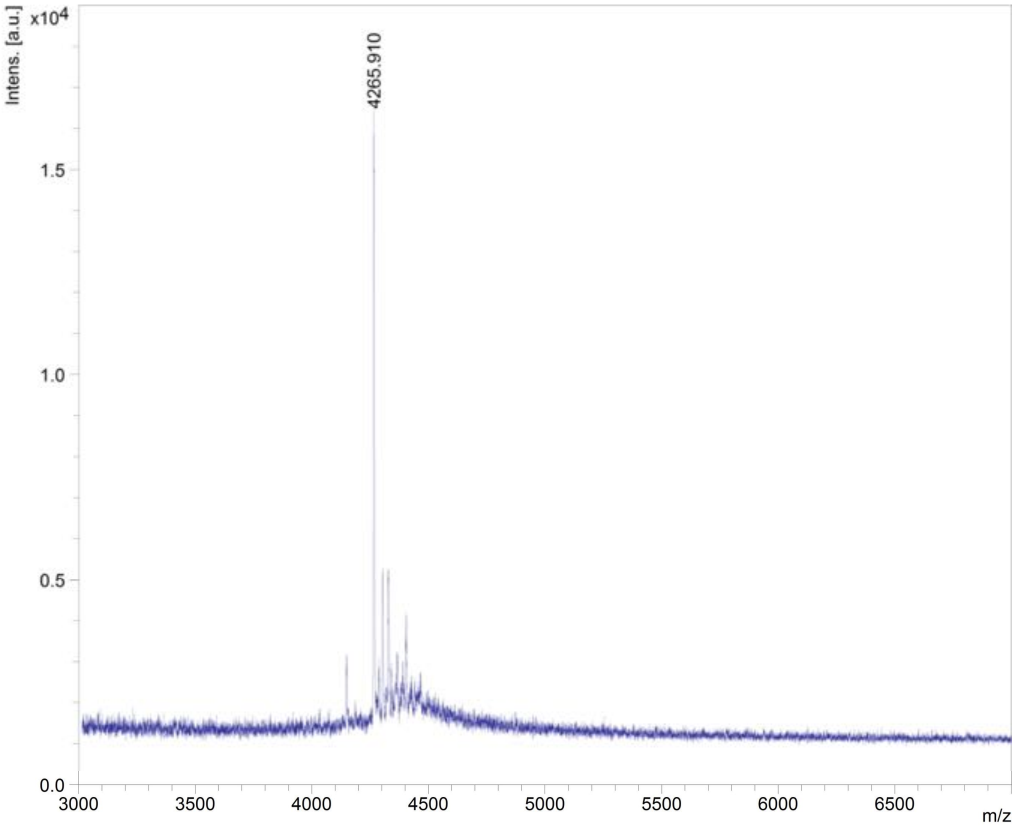

UD-12

[B conc. MeCN: 7-40% (30 min)]

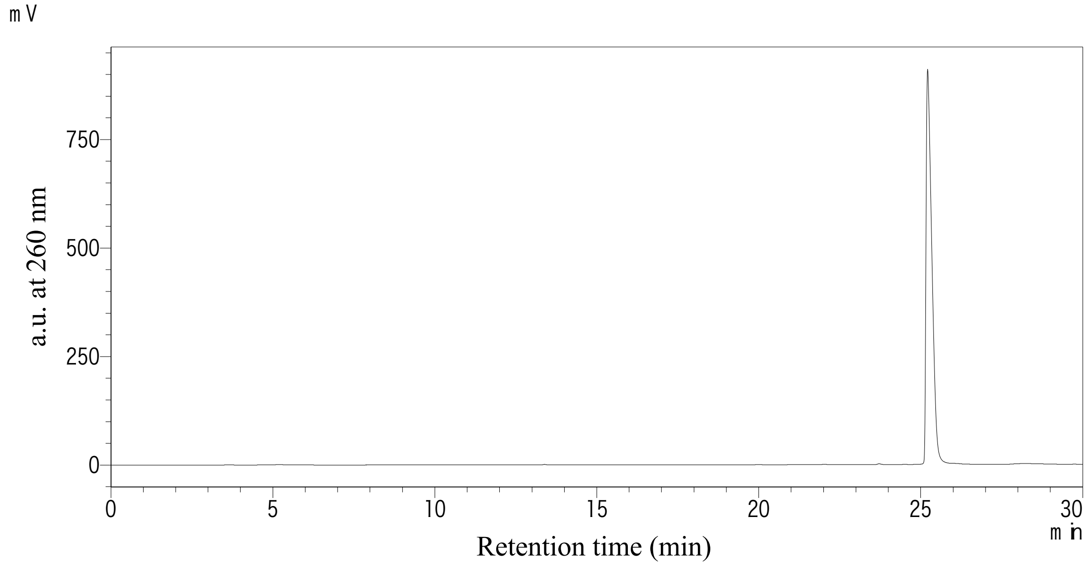

[calcd. (M-H)$^{-}$: 4314.2, found: 4314.2]

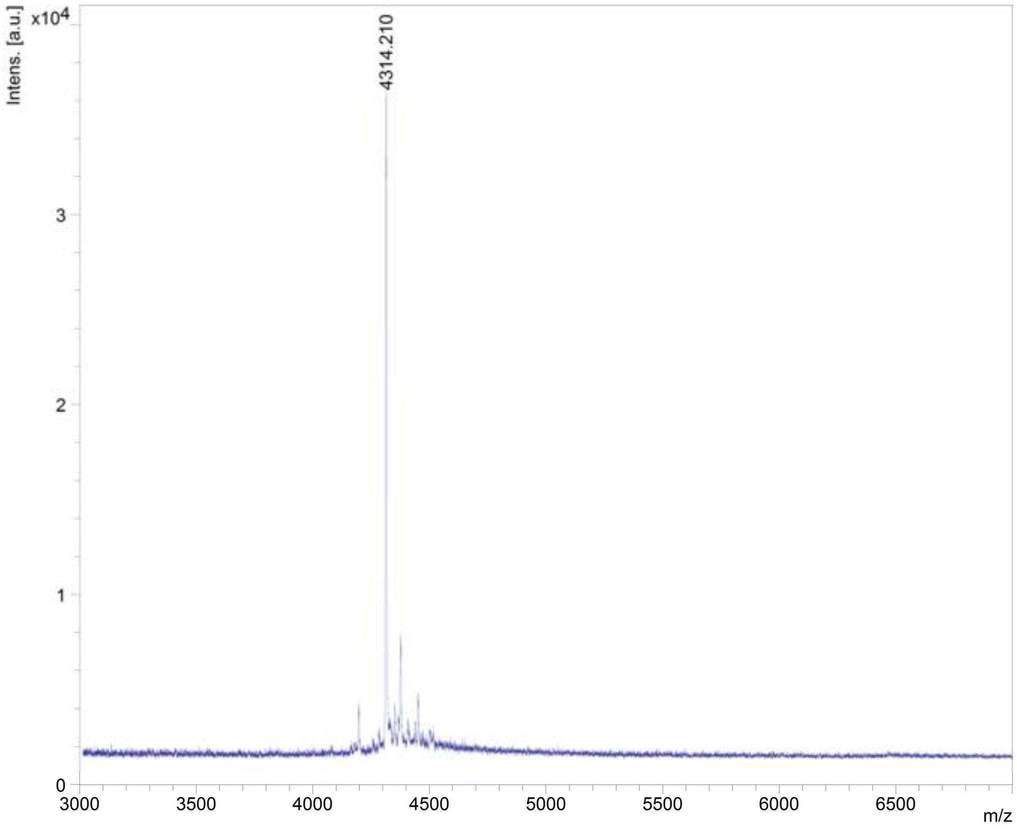

UD-13

[B conc. MeCN: 7-40% (30 min)]

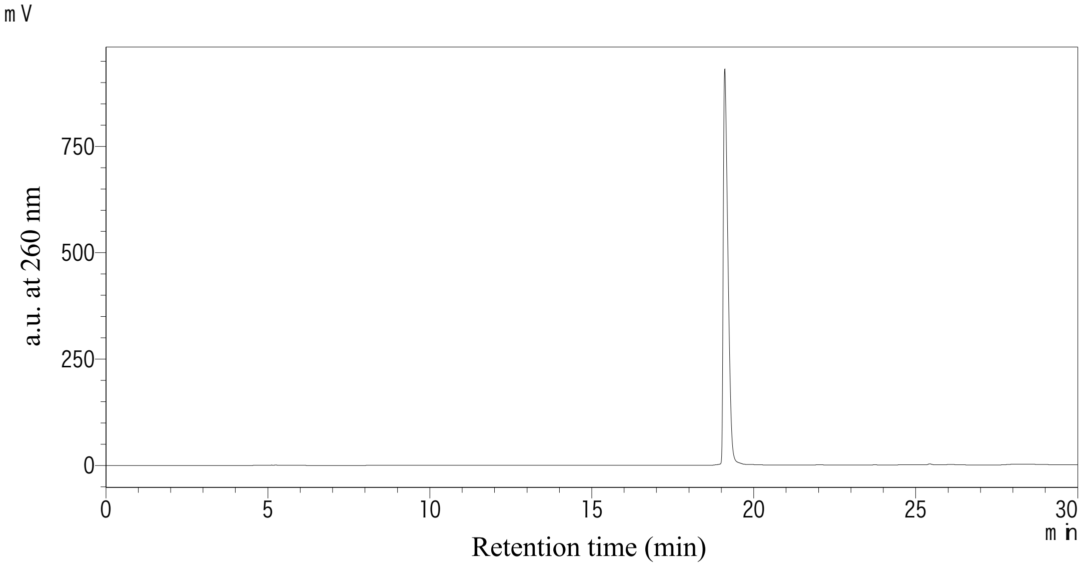

[calcd. (M-H)$^{-}$: 4282.2, found: 4282.7]

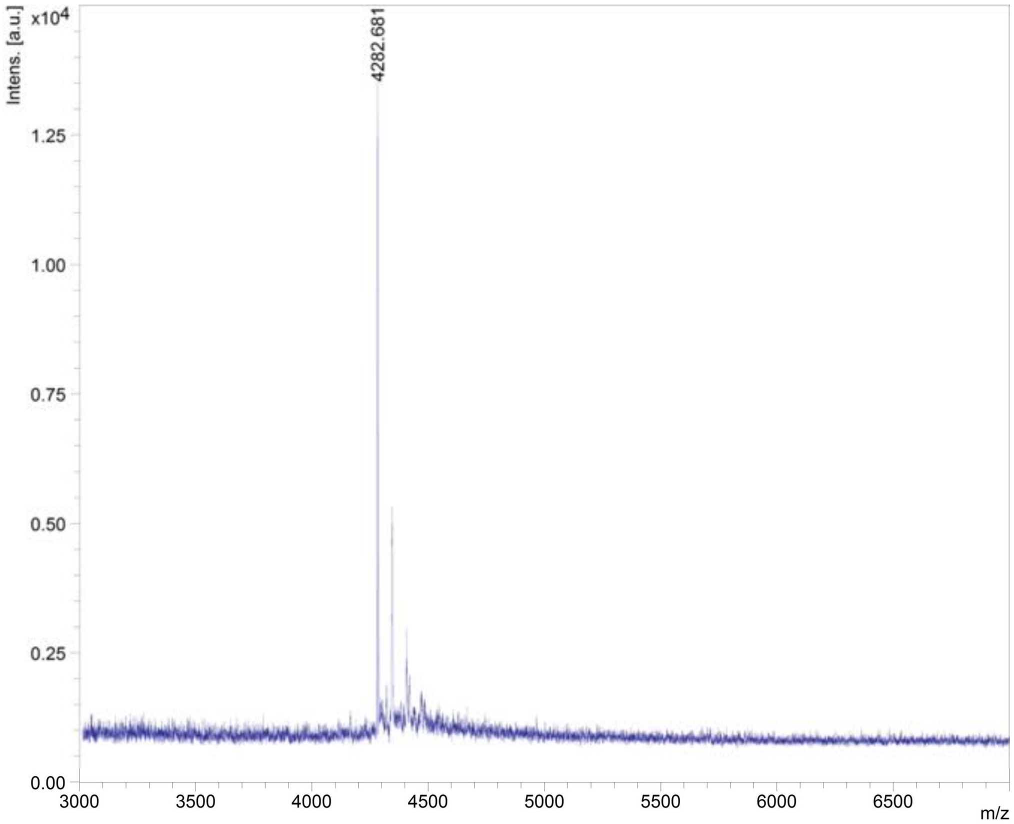

UD-14

[B conc. MeCN: 7-40% (30 min)]

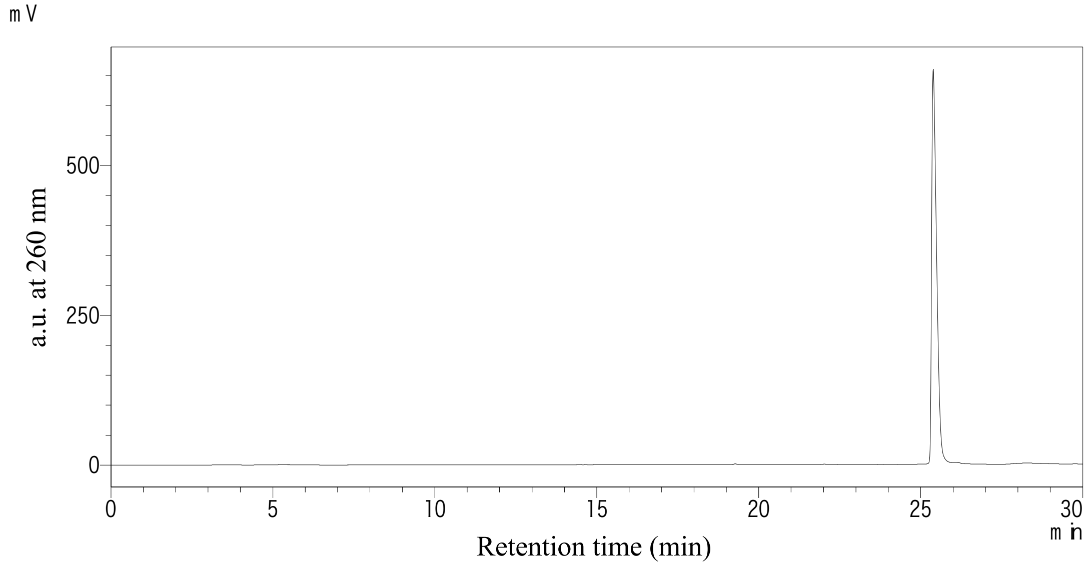

[calcd. (M-H)$^{-}$: 4292.2, found: 4292.8]

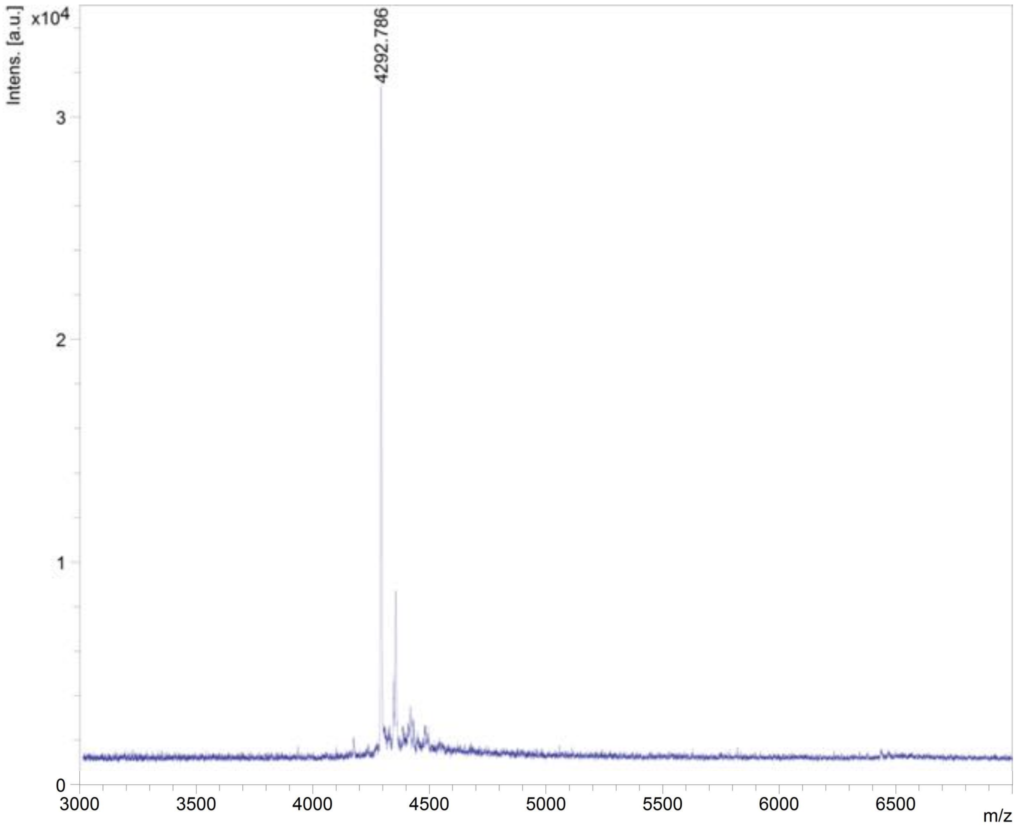

UD-15

[B conc. MeCN: 7-40% (30 min)]

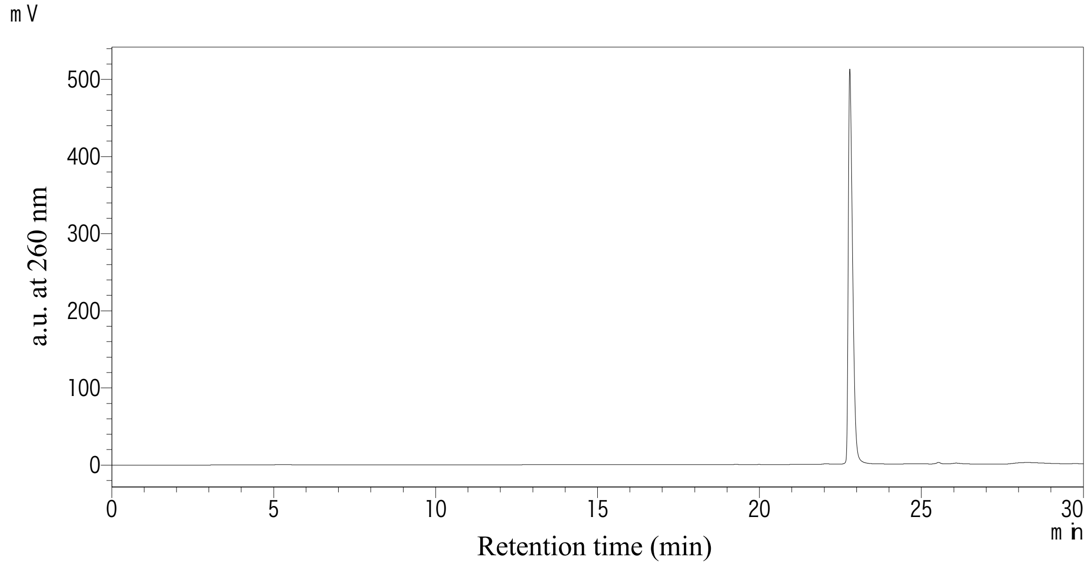

[calcd. (M-H)$^{-}$: 4266.2, found: 4266.1]

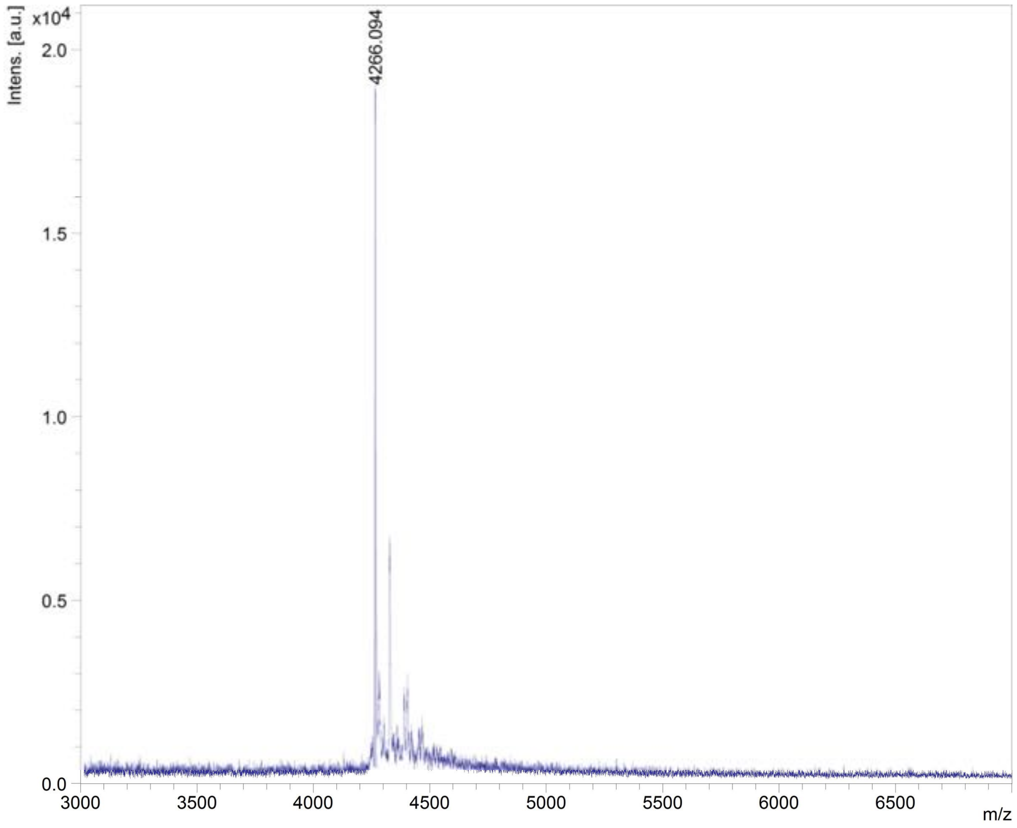

UD-16

[B conc. MeCN: 7-40% (30 min)]

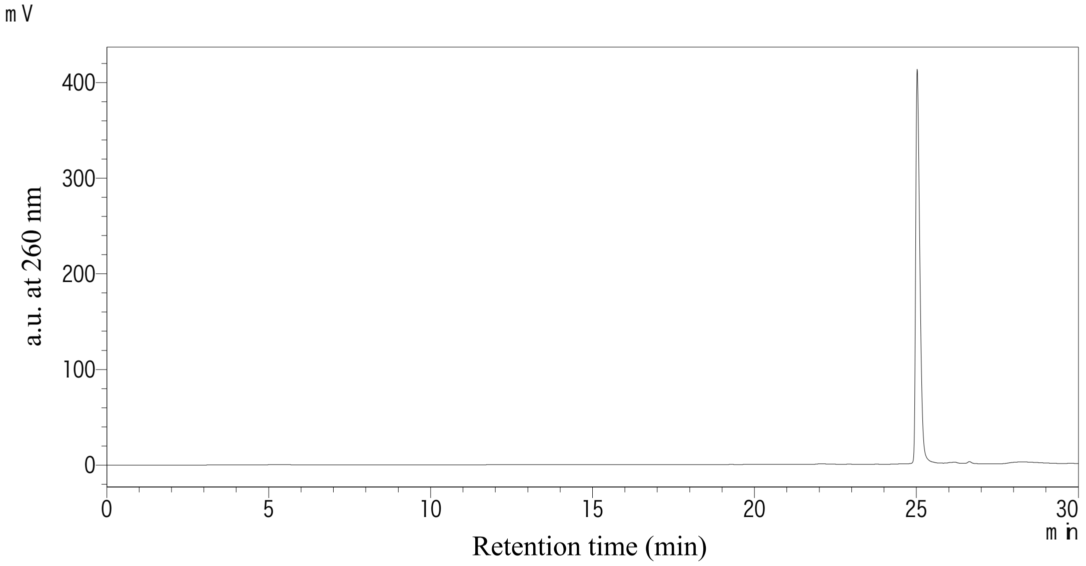

[calcd. $(M-H)^{-}$: 4300.2, found: 4300.8]

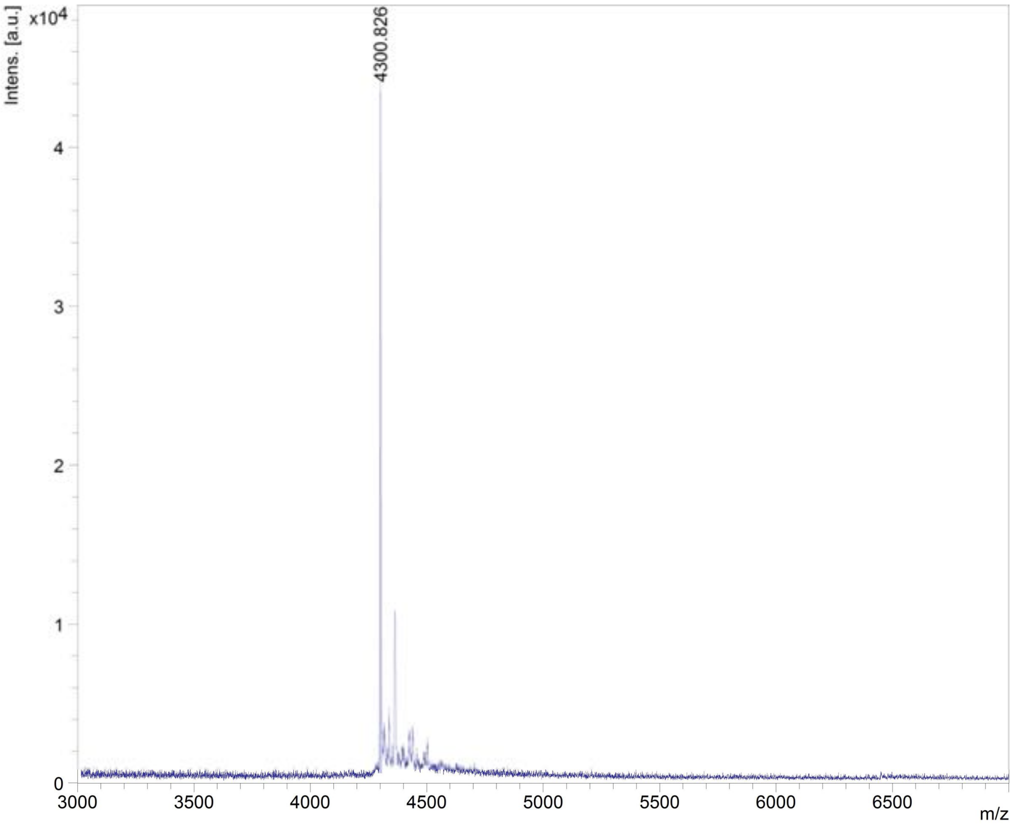

UD-17

[B conc. MeCN: 7-40% (30 min)]

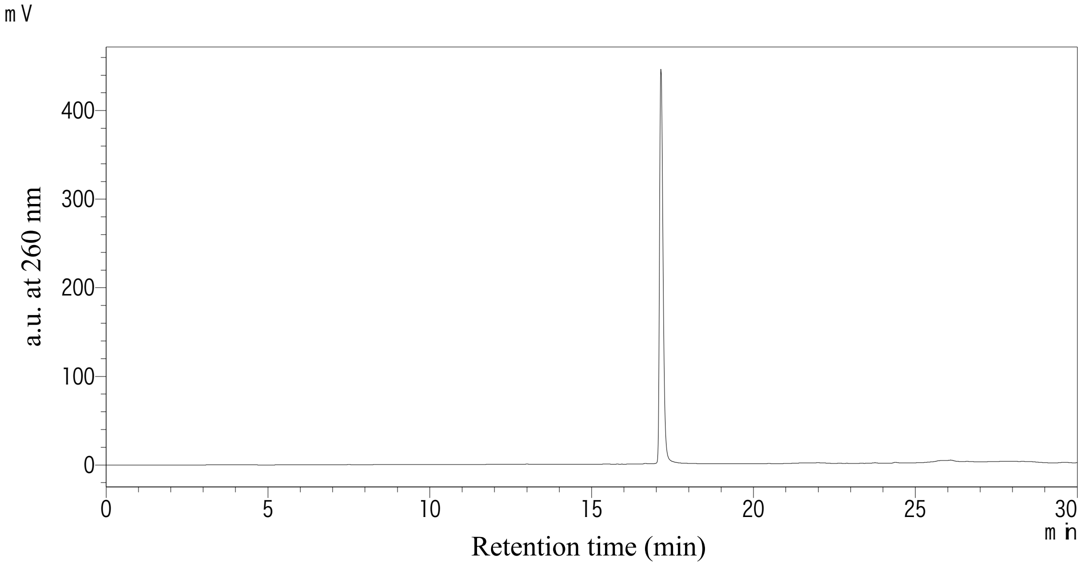

[calcd. (M-H)$^{-}$: 4309.2, found: 4309.9]

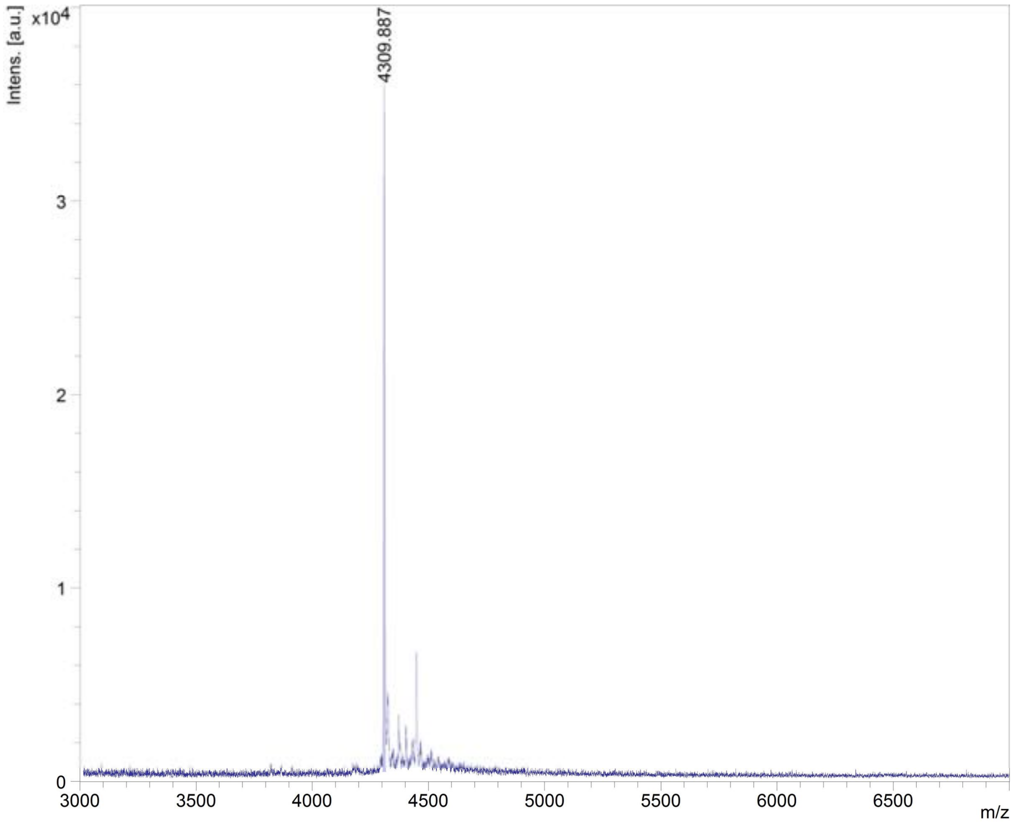

UD-18

[B conc. MeCN: 7-40% (30 min)]

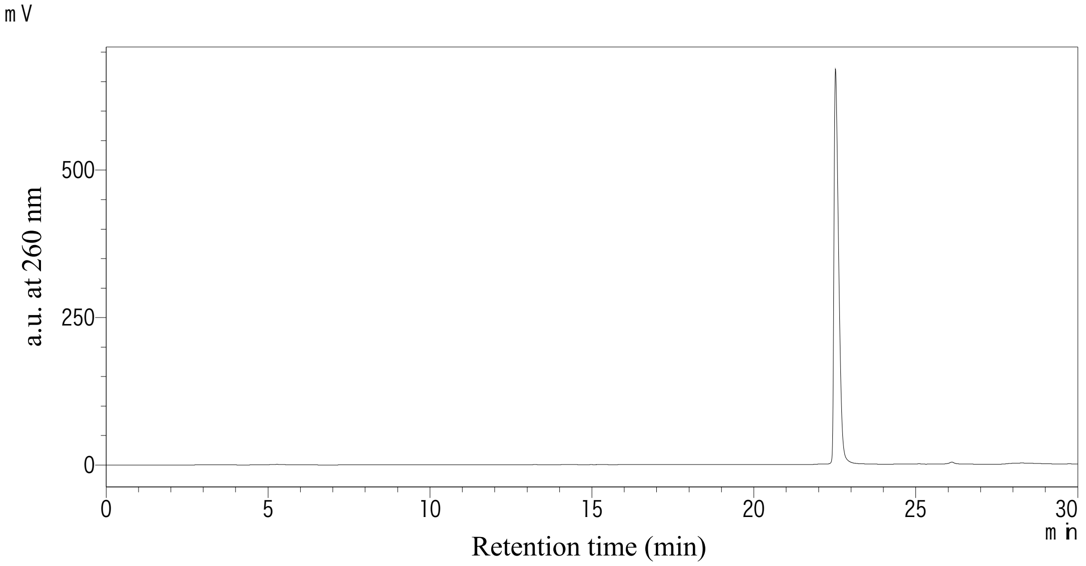

[calcd. (M-H)$^{-}$: 4274.2, found: 4274.2]

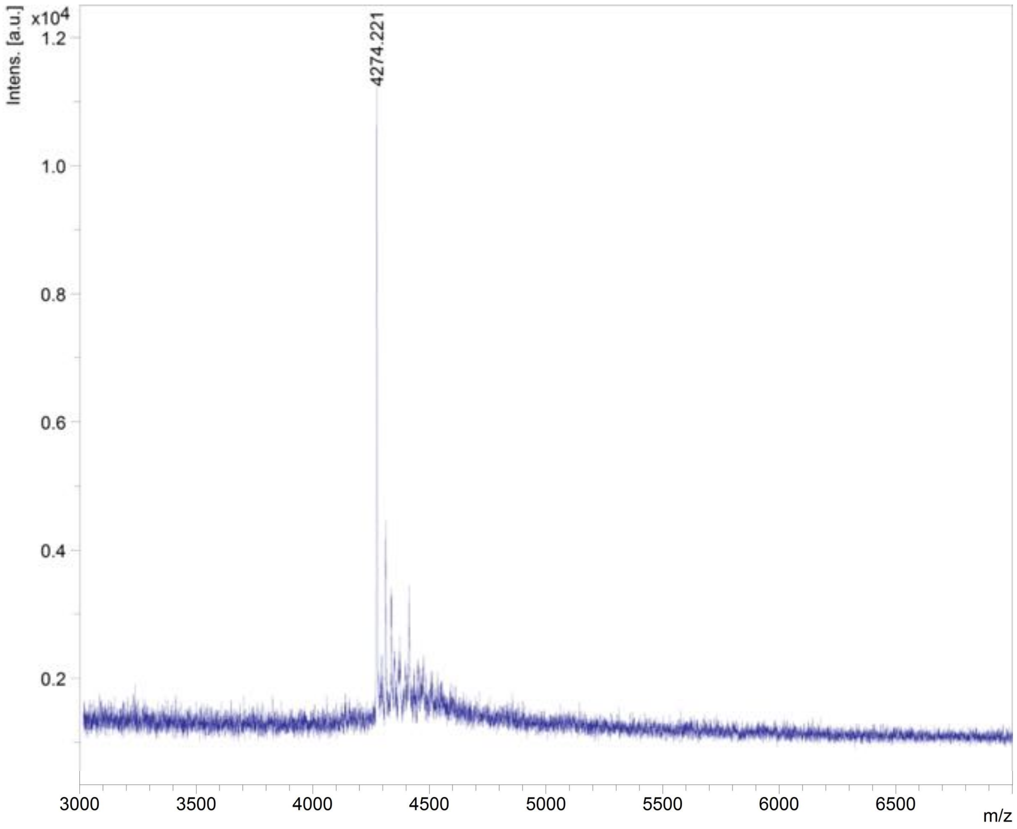

UD-19

[B conc. MeCN: 7-40% (30 min)]

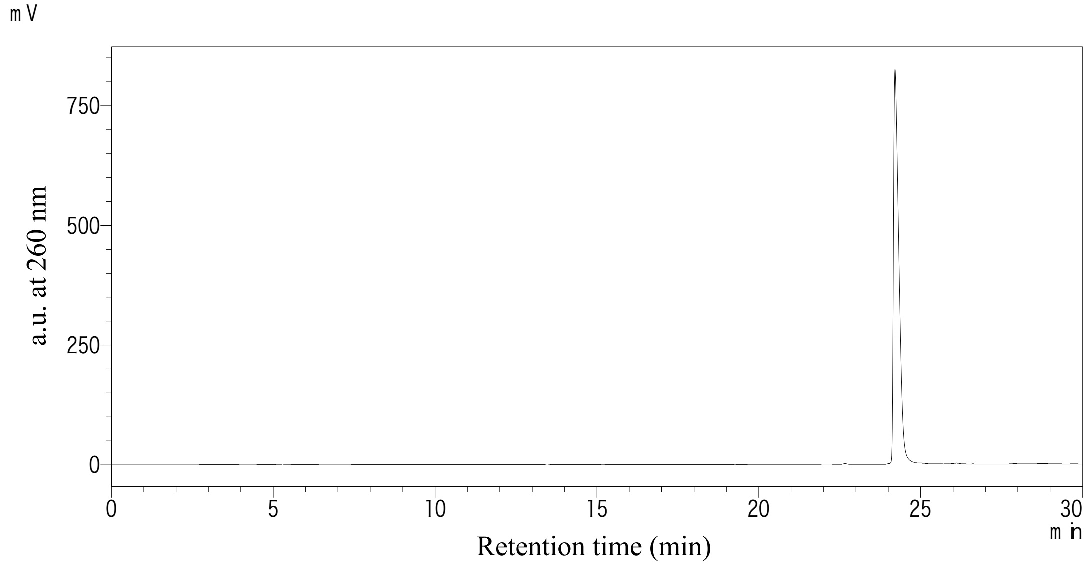

[calcd. (M-H)$^-$: 4322.2, found: 4322.7]

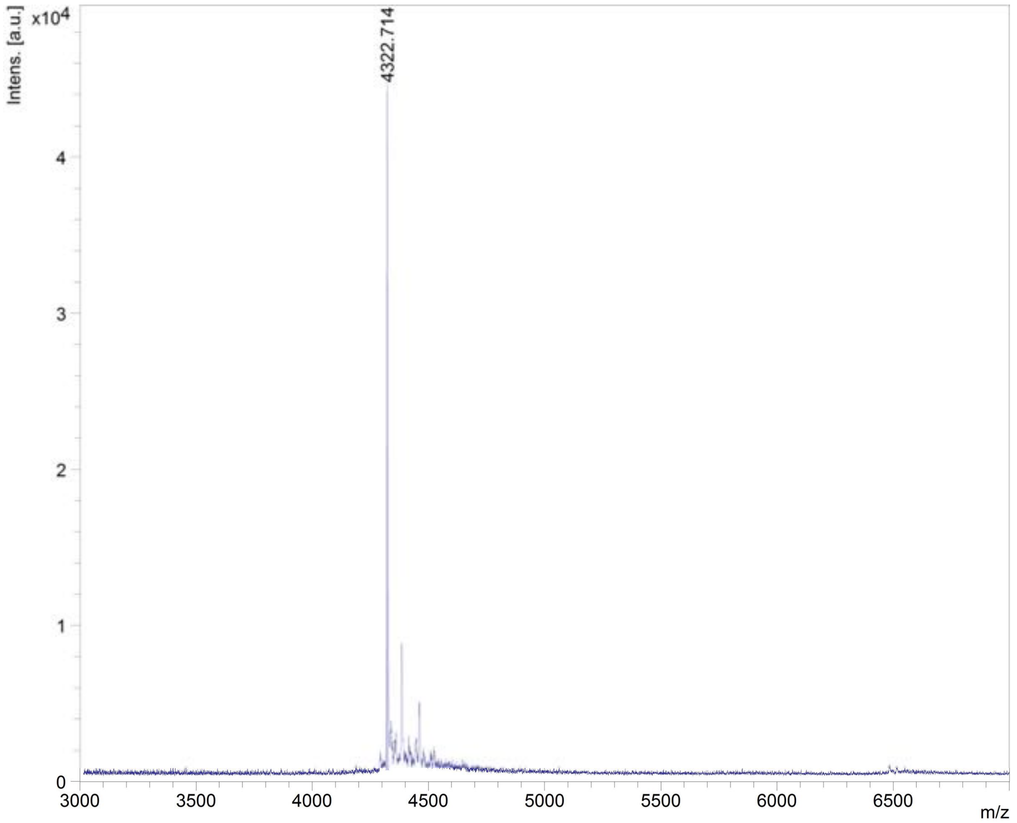

UD-20

[B conc. MeCN: 7-40% (30 min)]

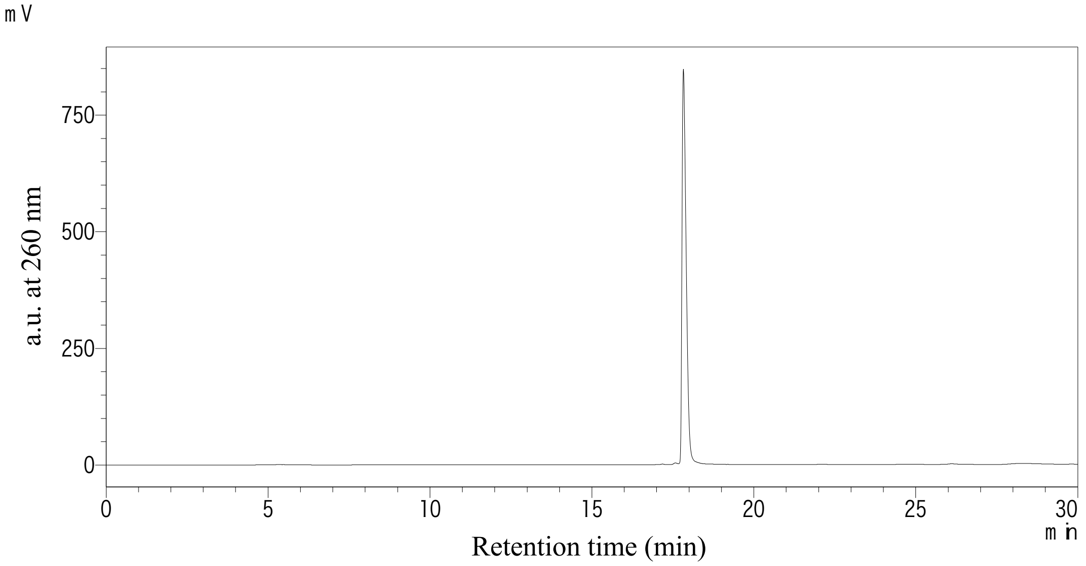

[calcd. $(M-H)^-$: 4290.2, found: 4290.0]

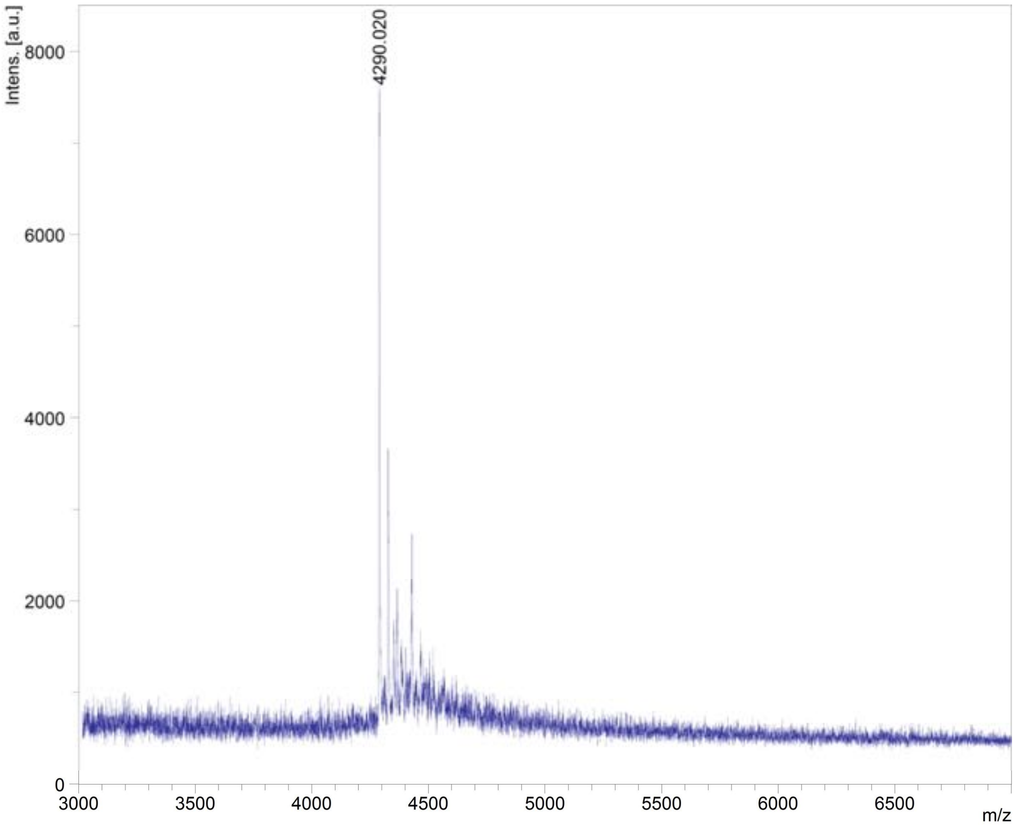

UD-21

[B conc. MeCN: 7-40% (30 min)]

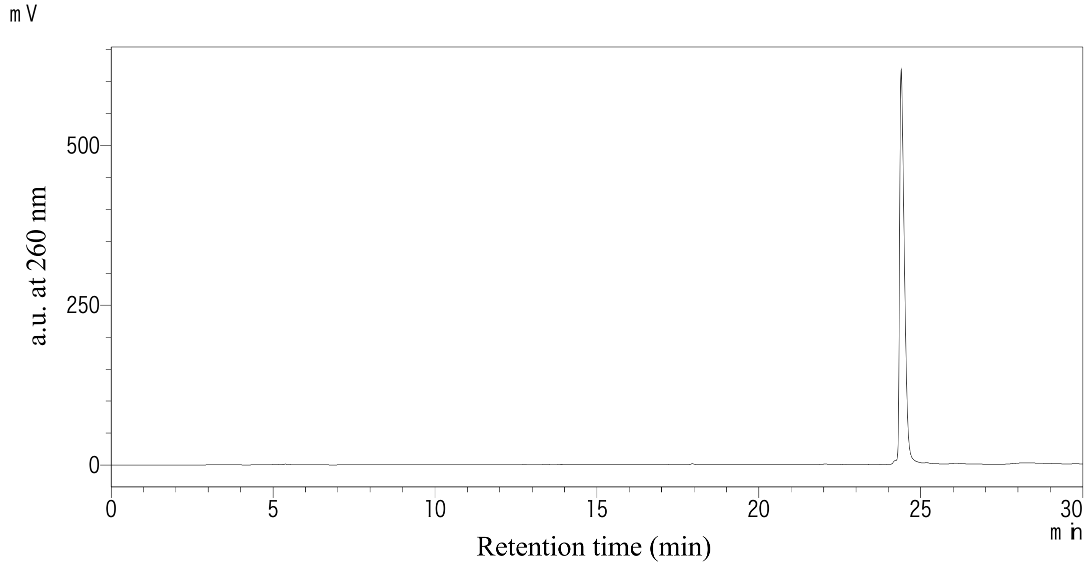

[calcd. $(M-H)^{-}$: 4300.2, found: 4300.1]

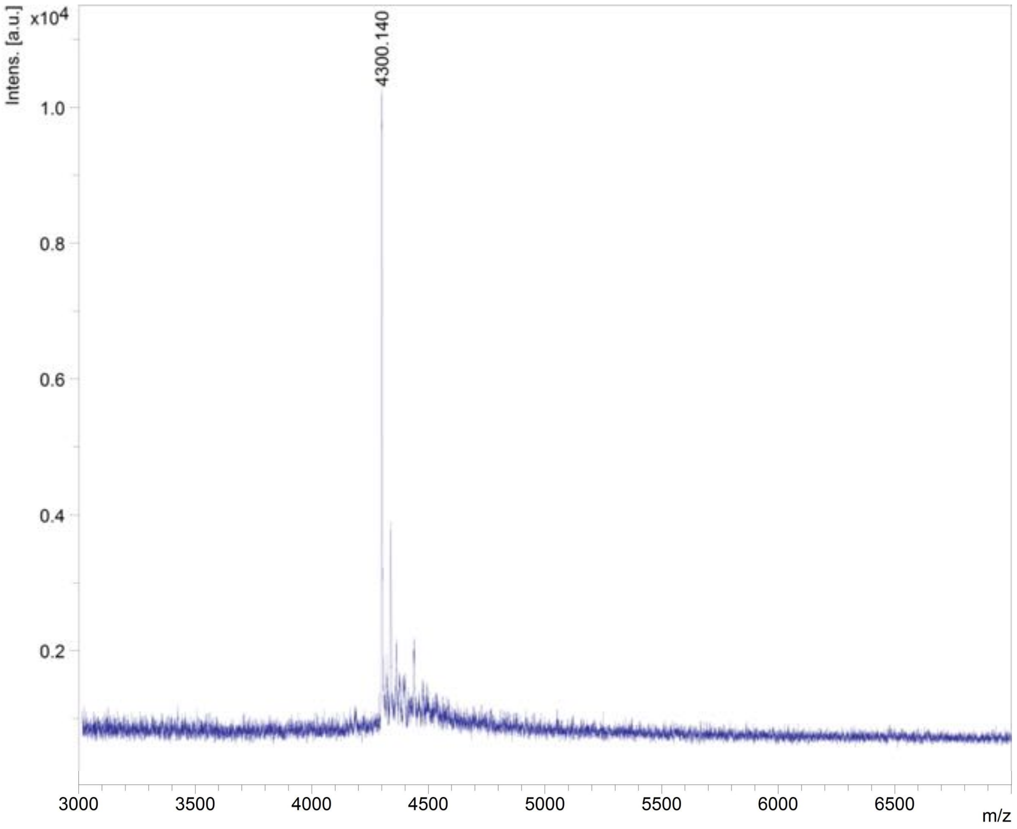

UD-22

[B conc. MeCN: 7-40% (30 min)]

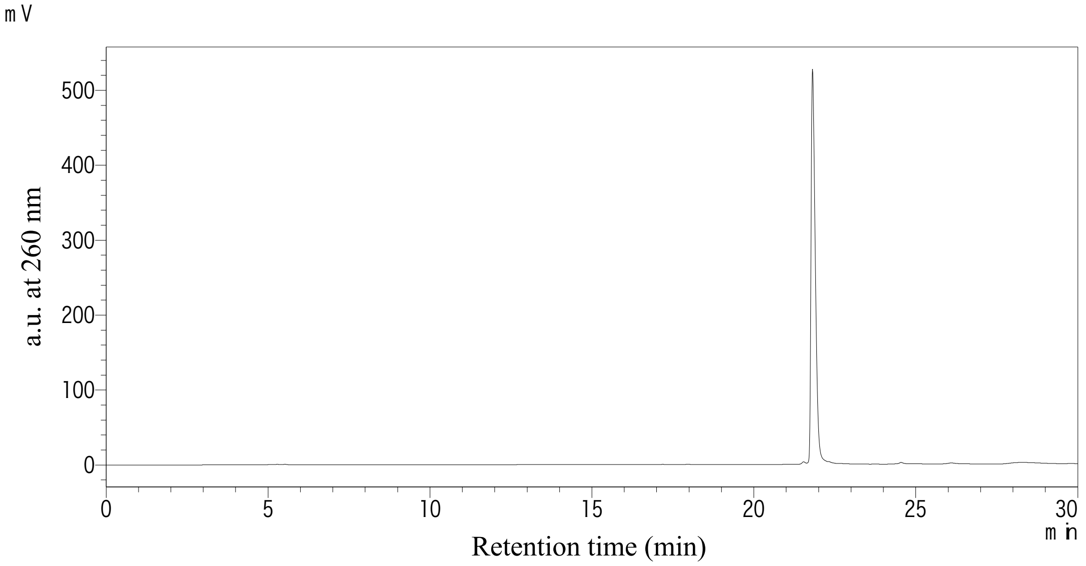

[calcd. (M-H)$^{-}$: 4274.2, found: 4275.1]

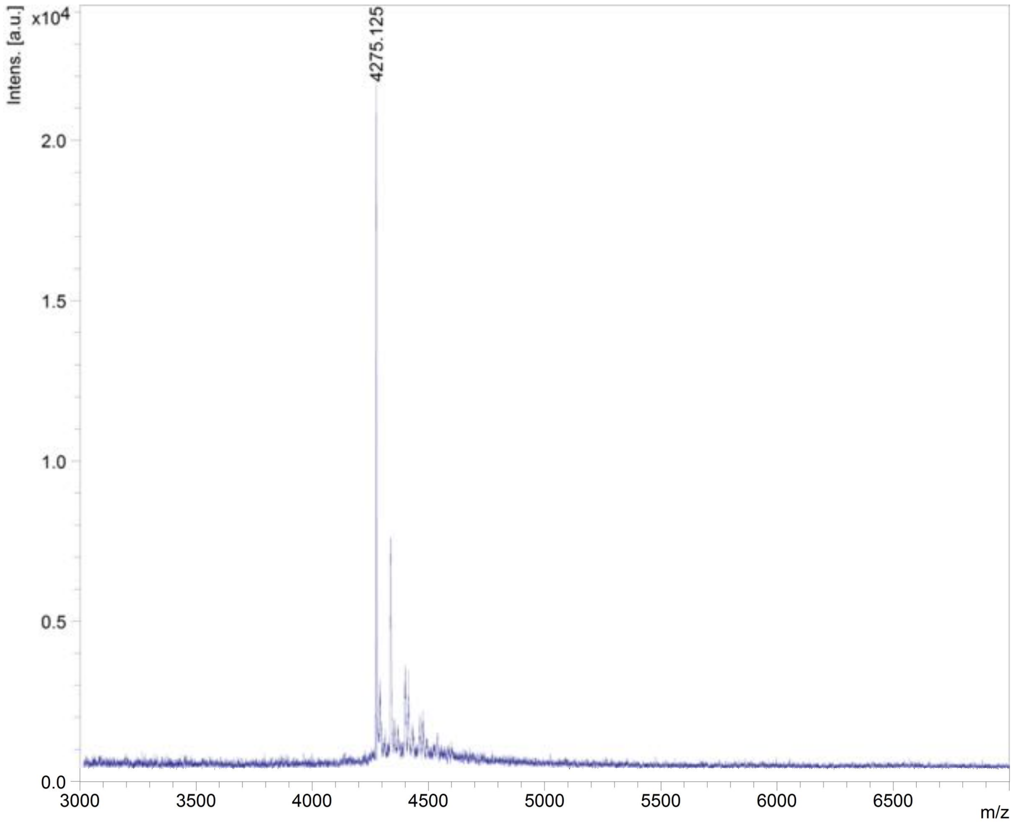

UD-23

[B conc. MeCN: 7-40% (30 min)]

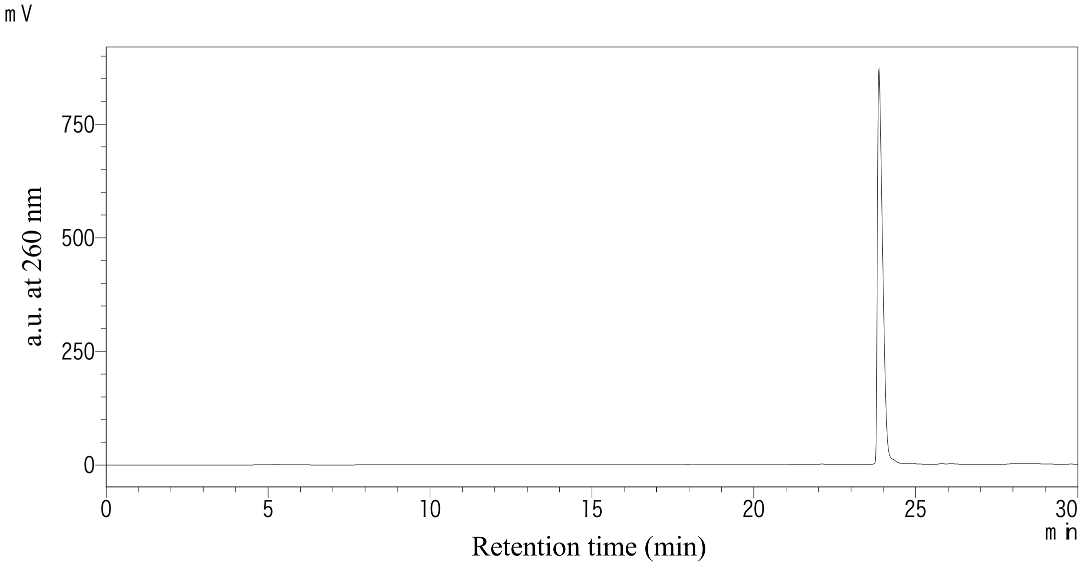

[calcd. (M-H)$^-$: 4308.2, found: 4308.1]

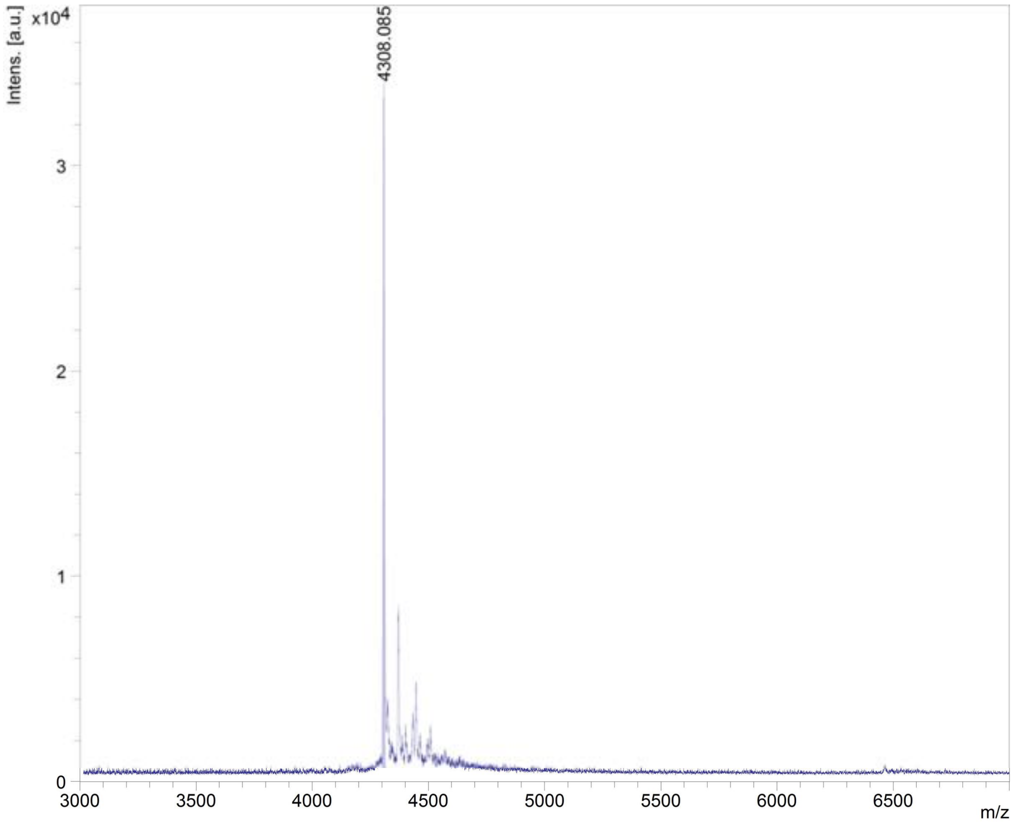

UD-24

[B conc. MeCN: 7-40% (30 min)]

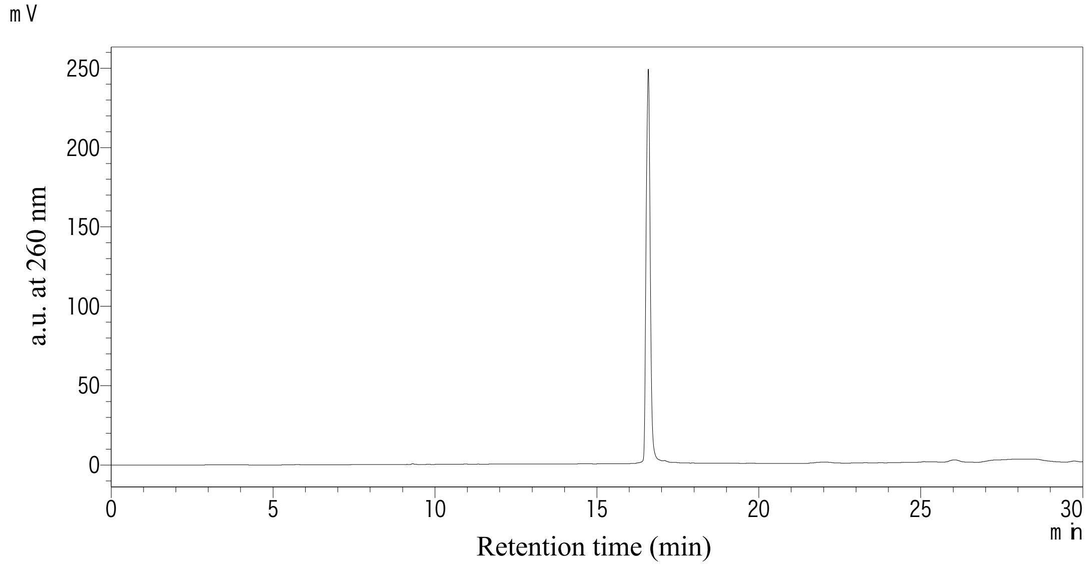

[calcd. (M-H)$^{-}$: 4317.2, found: 4318.4]

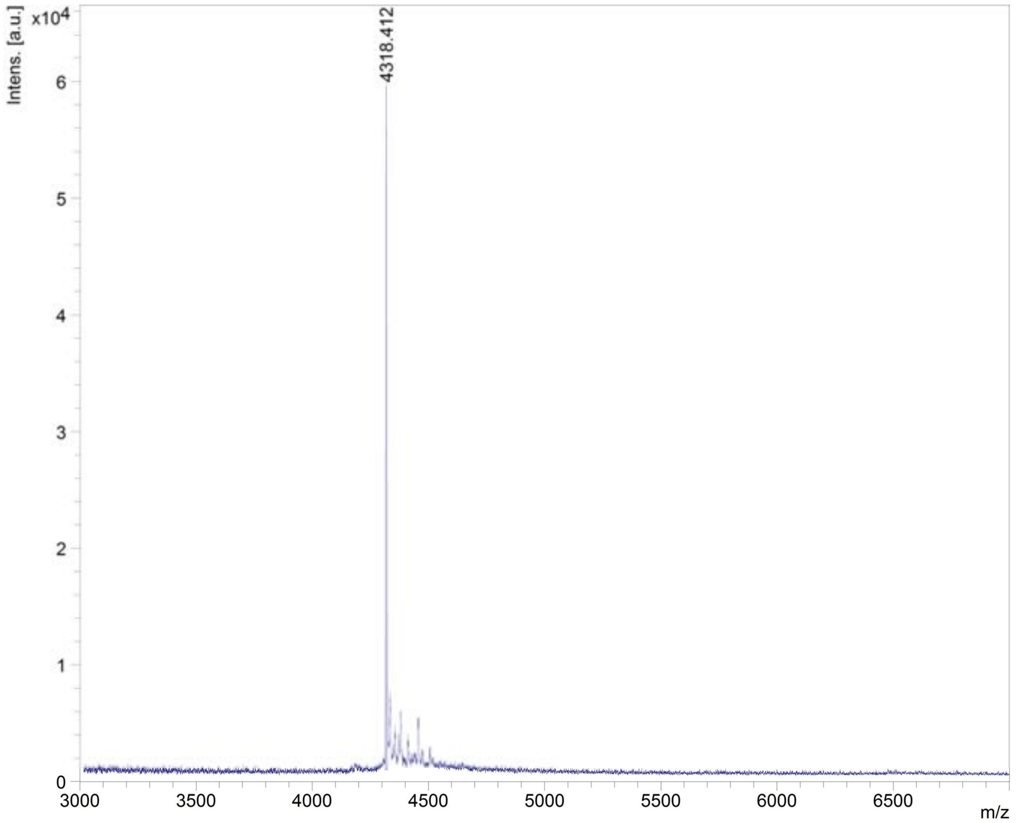

UD-27

[B conc. MeCN: 7-40% (30 min)]

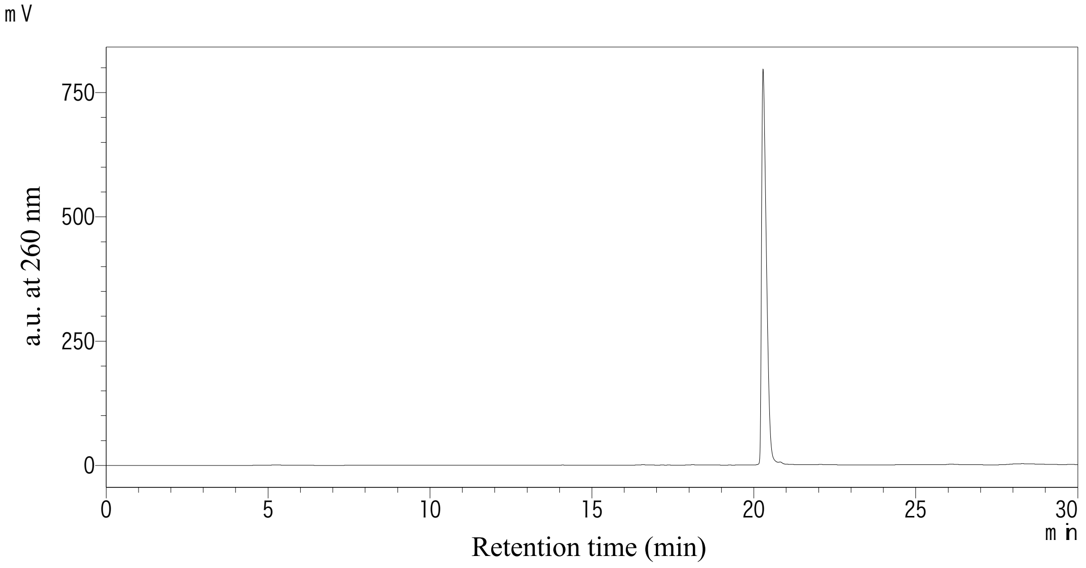

[calcd. (M-H)$^{-}$: 4176.1, found: 4176.7]

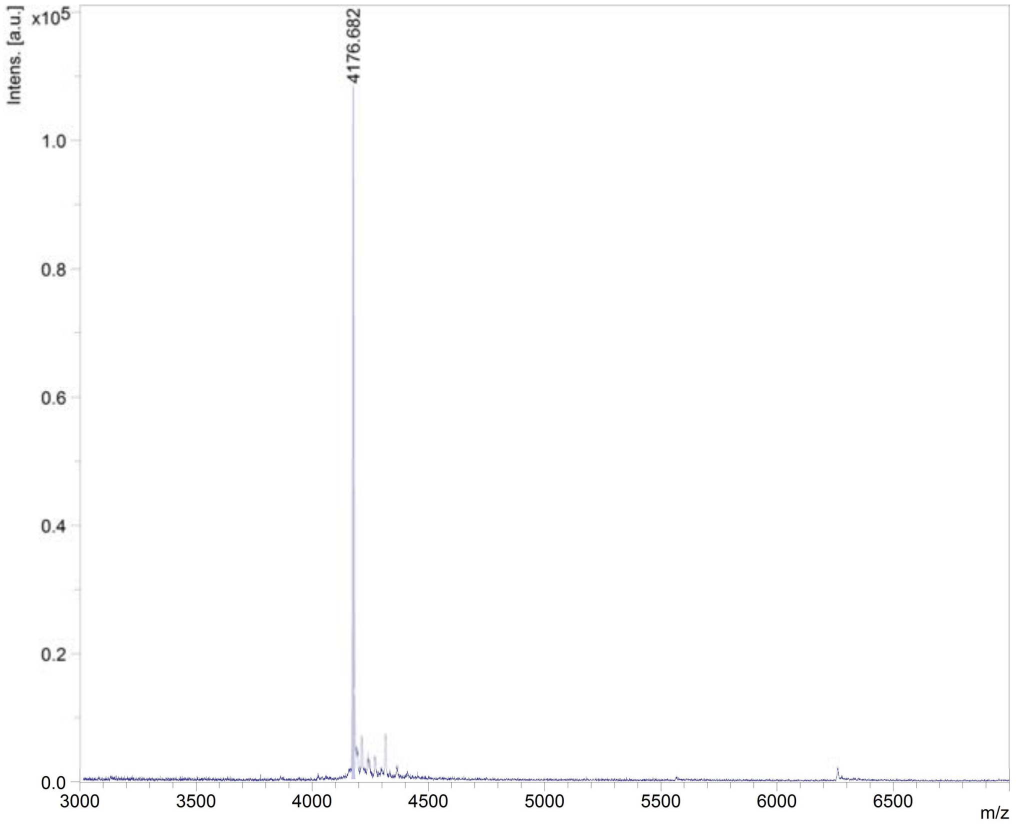

UD-28

[B conc. MeCN: 7-40% (30 min)]

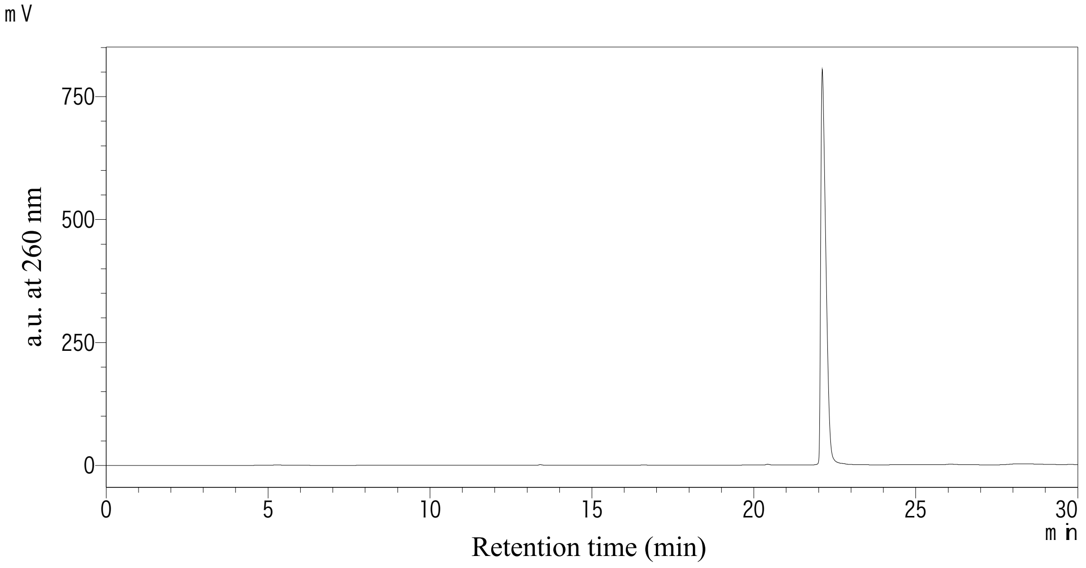

[calcd. (M-H)⁻: 4224.1, found: 4224.6]

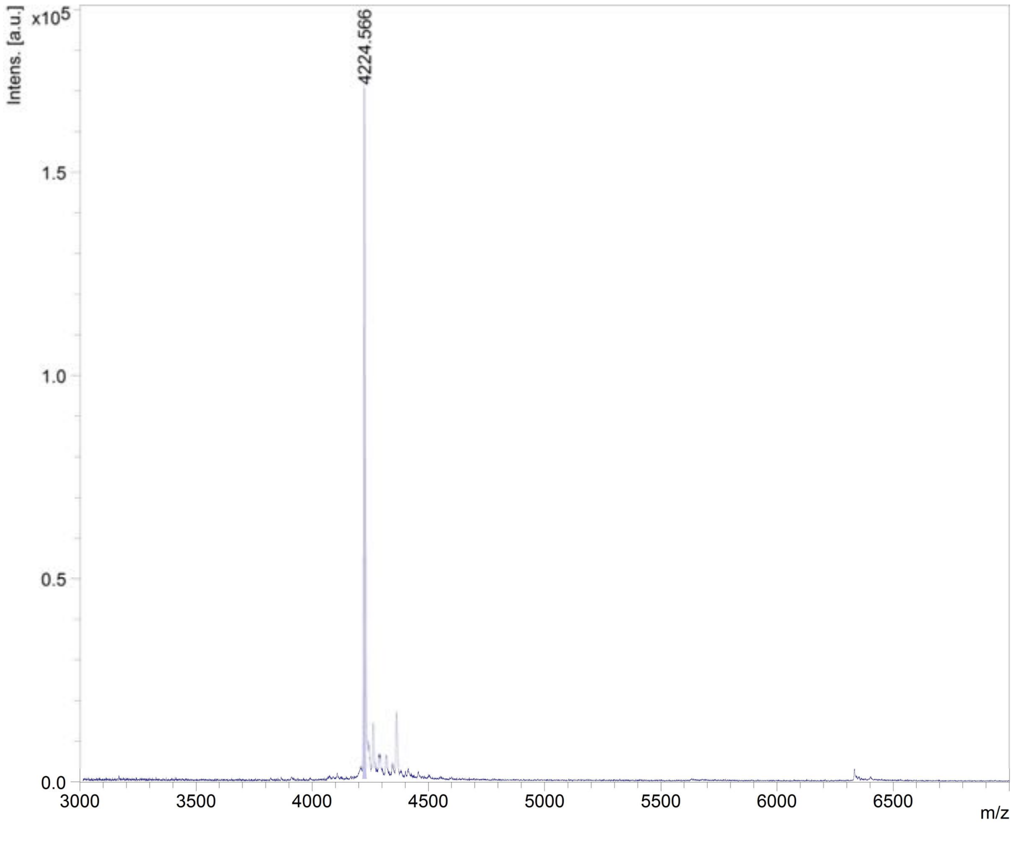

UD-29

[B conc. MeCN: 7-40% (30 min)]

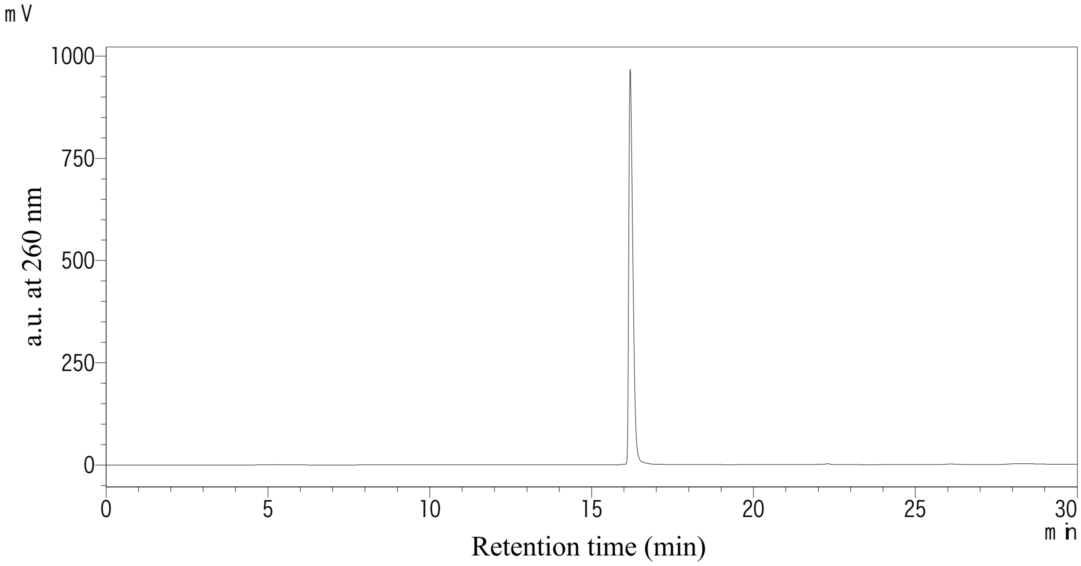

[calcd. (M-H)$^-$: 4192.1, found: 4191.8]

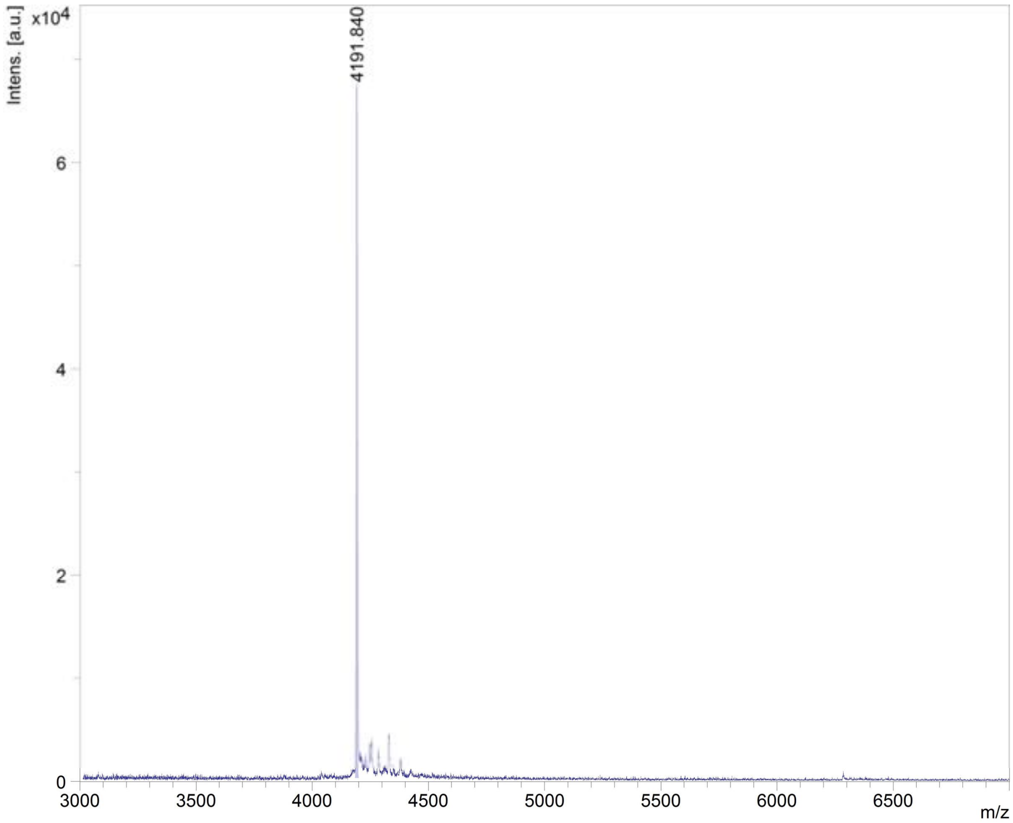

UD-30

[B conc. MeCN: 7-40% (30 min)]

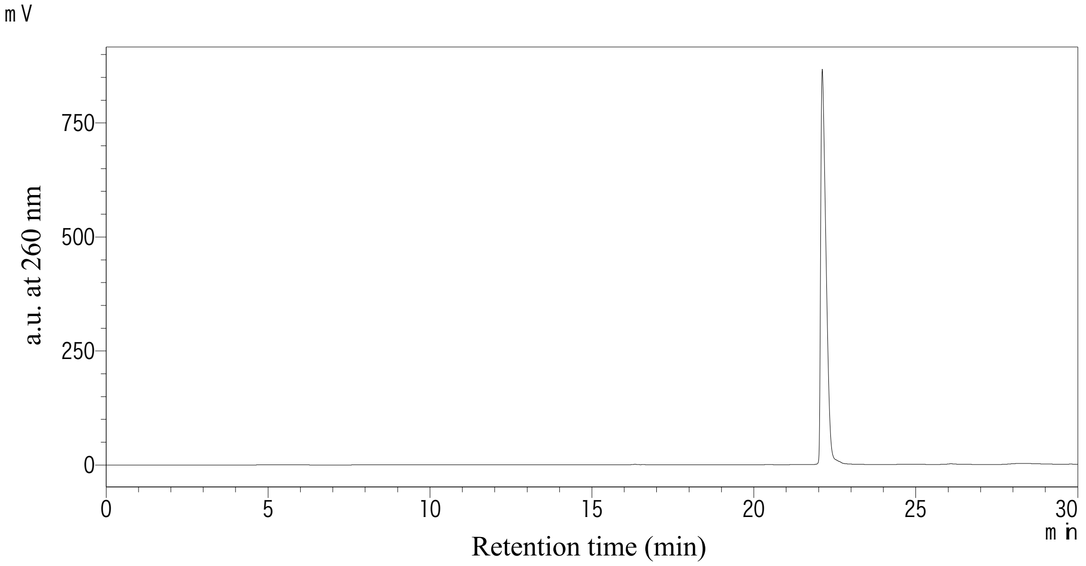

[calcd. (M-H)$^{-}$: 4202.1, found: 4203.8]

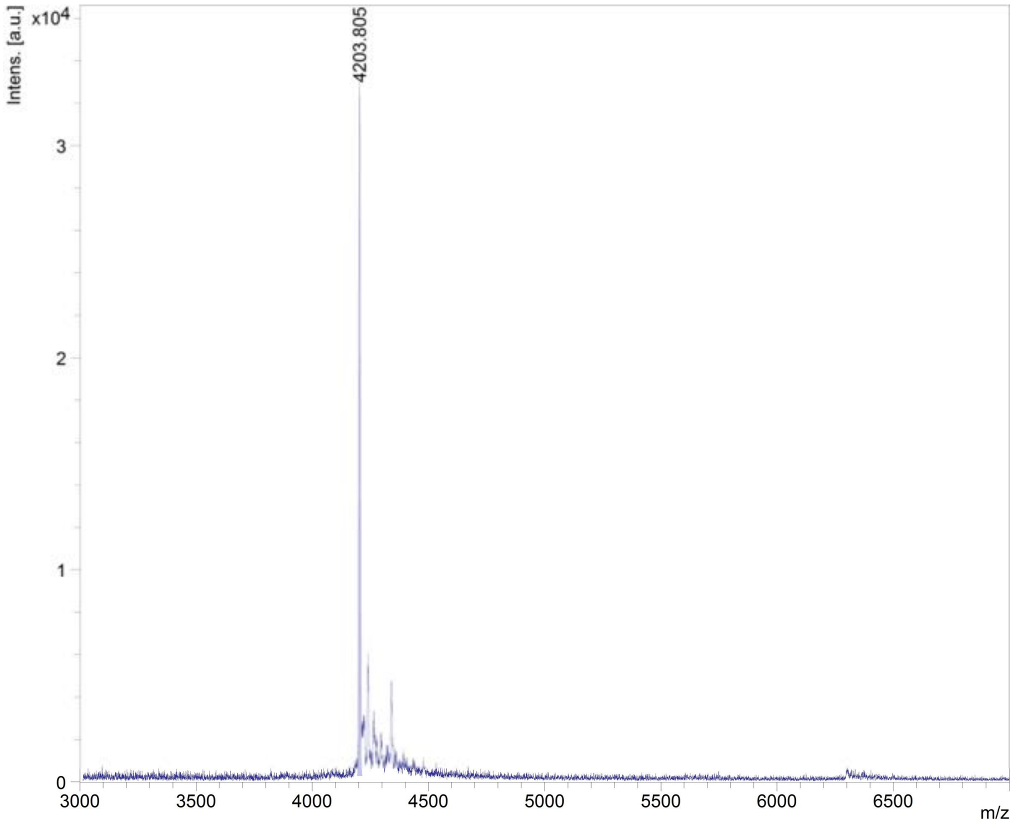

UD-31

[B conc. MeCN: 7-40% (30 min)]

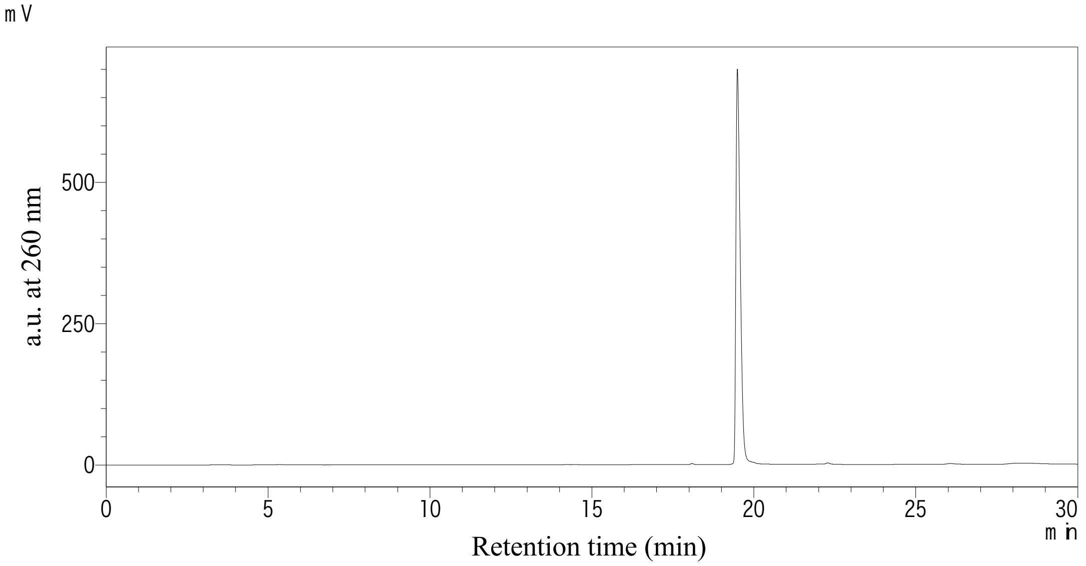

[calcd. (M-H)$^{-}$: 4176.1, found: 4177.8]

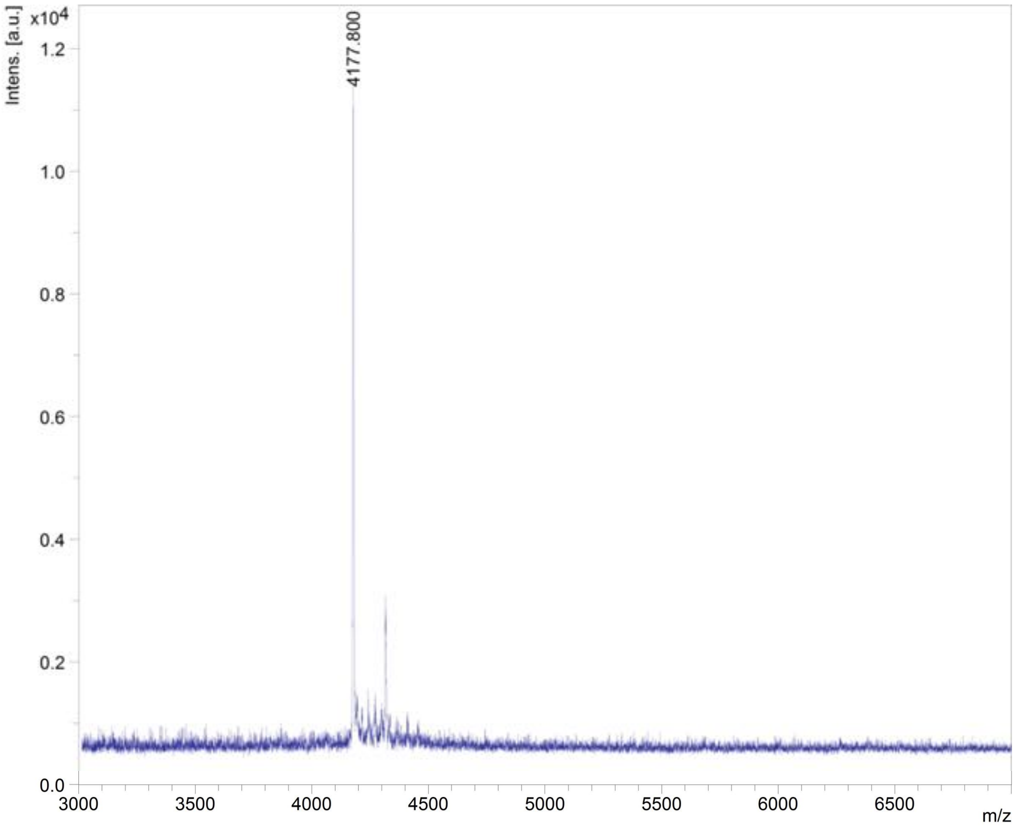

UD-32

[B conc. MeCN: 7-40% (30 min)]

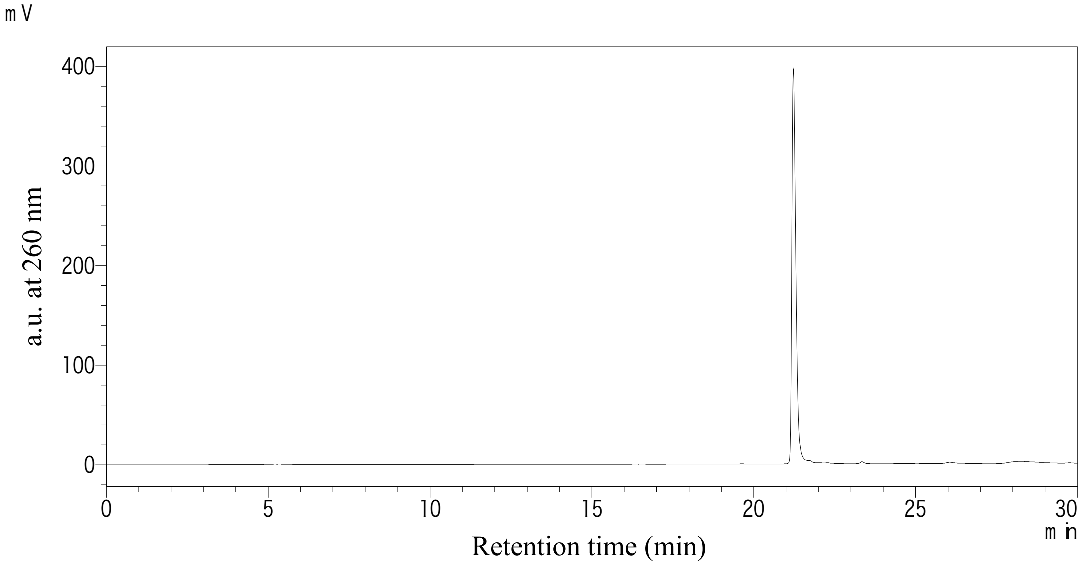

[calcd. $(M\text{-}H)^{-}$: 4210.1, found: 4210.8]

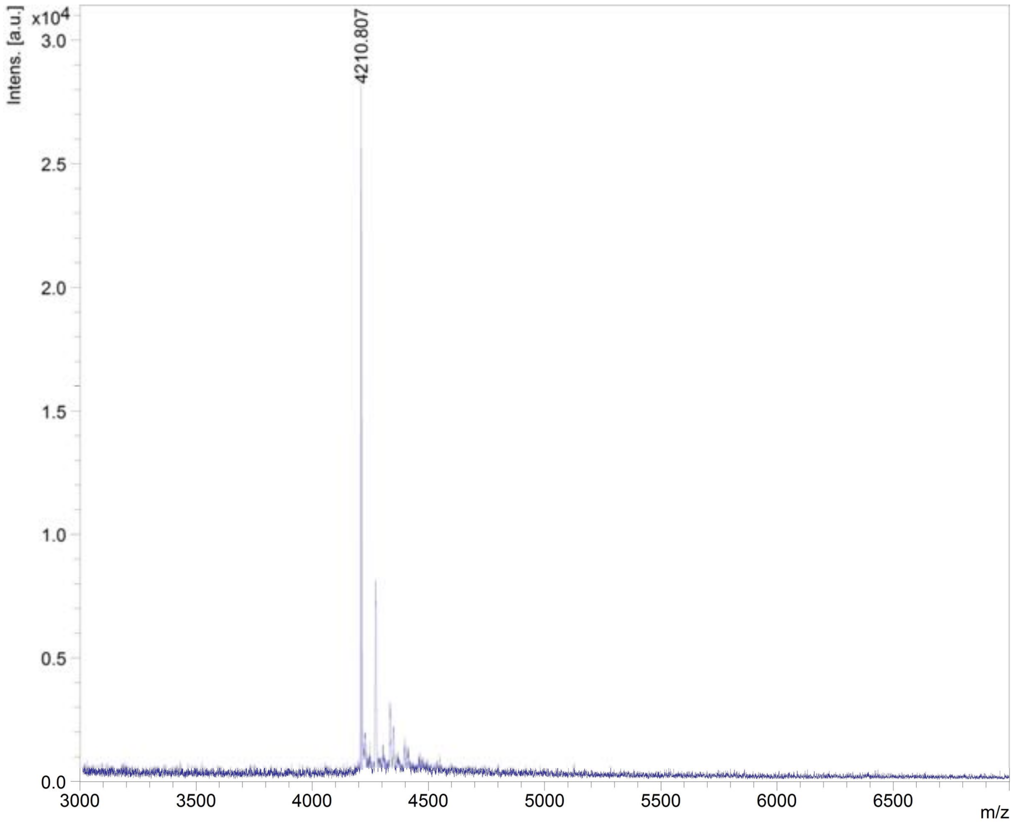

UD-33

[B conc. MeCN: 7-40% (30 min)]

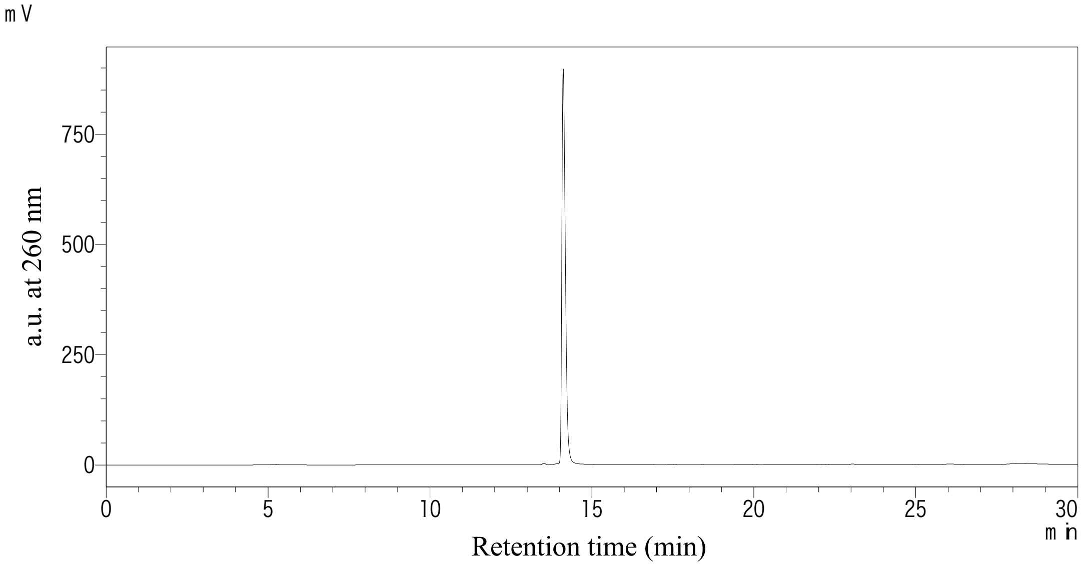

[calcd. $(M-H)^-$: 4219.2, found: 4218.7]

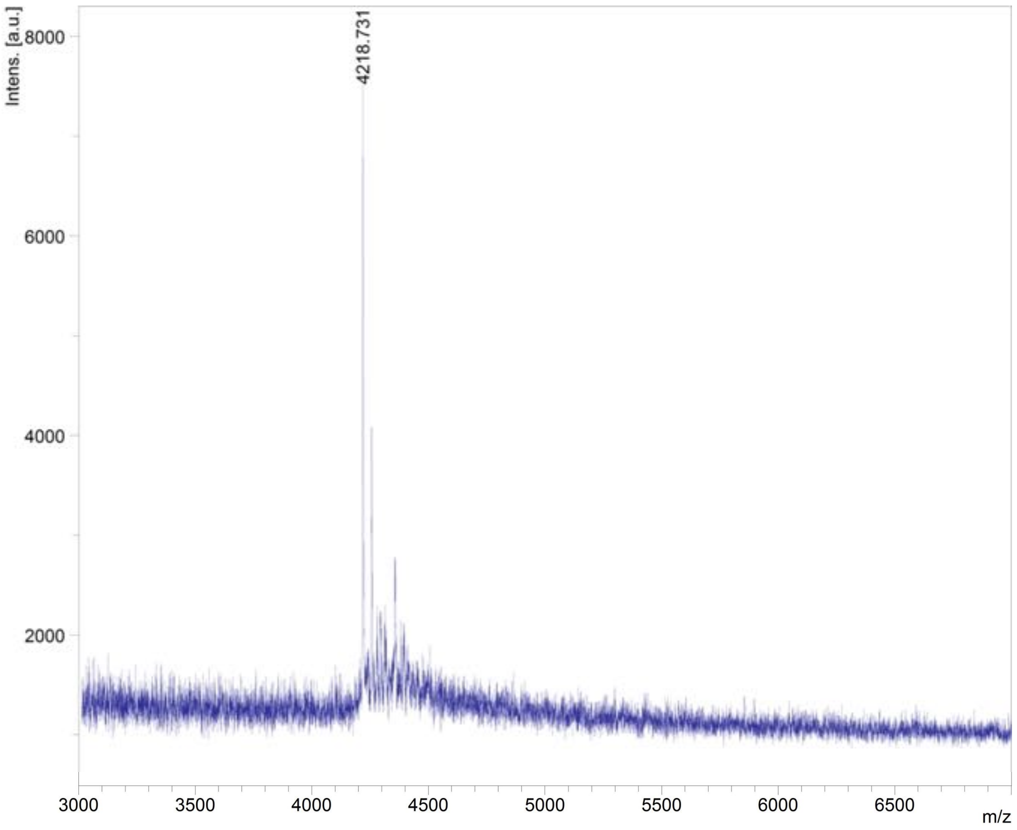

UD-34

[B conc. MeCN: 7-40% (30 min)]

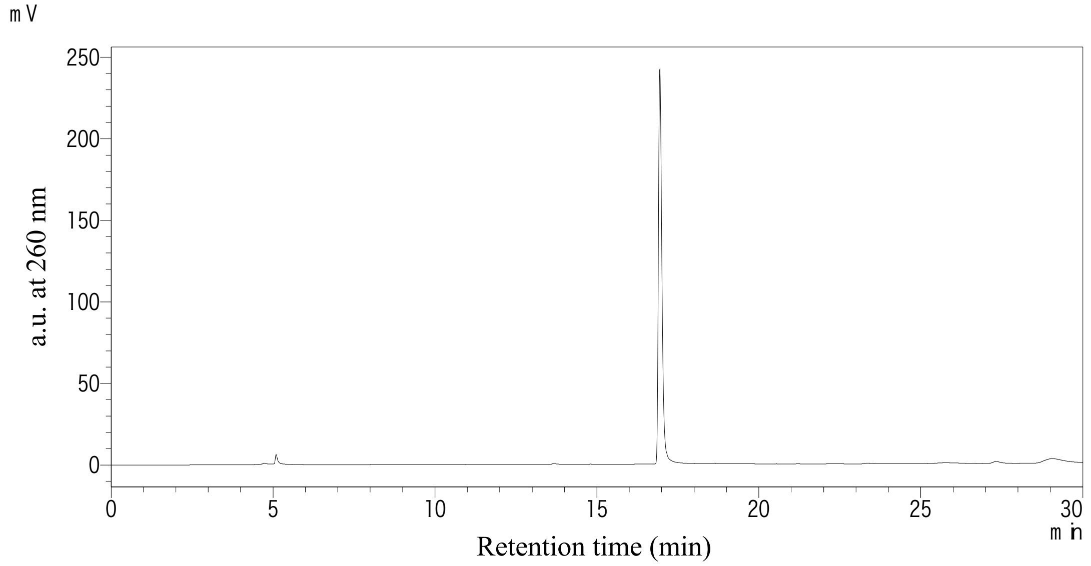

[calcd. (M-H)$^{-}$: 4192.1, found: 4192.7]

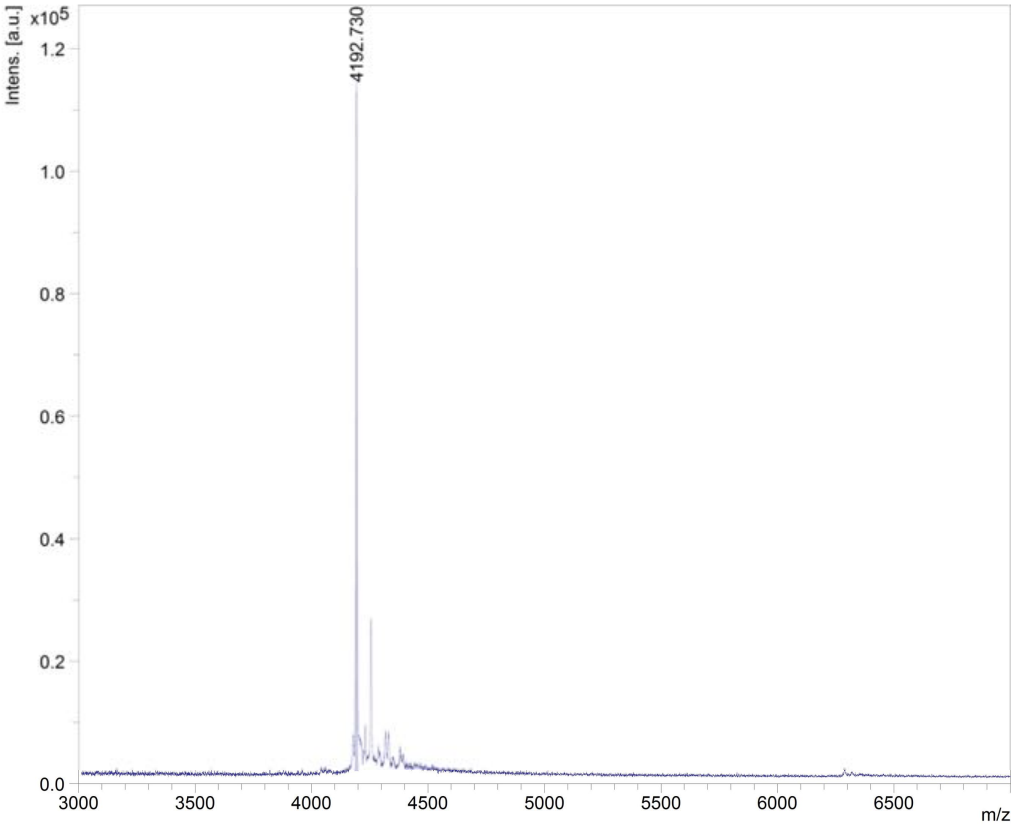

UD-35

[B conc. MeCN: 7-40% (30 min)]

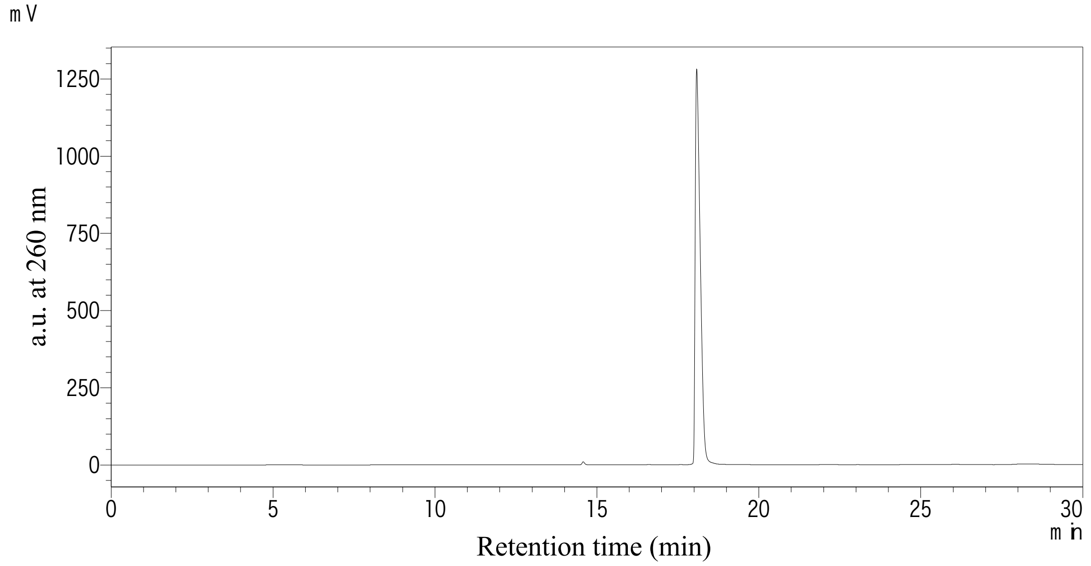

[calcd. (M-H)$^{-}$: 4240.1, found: 4240.6]

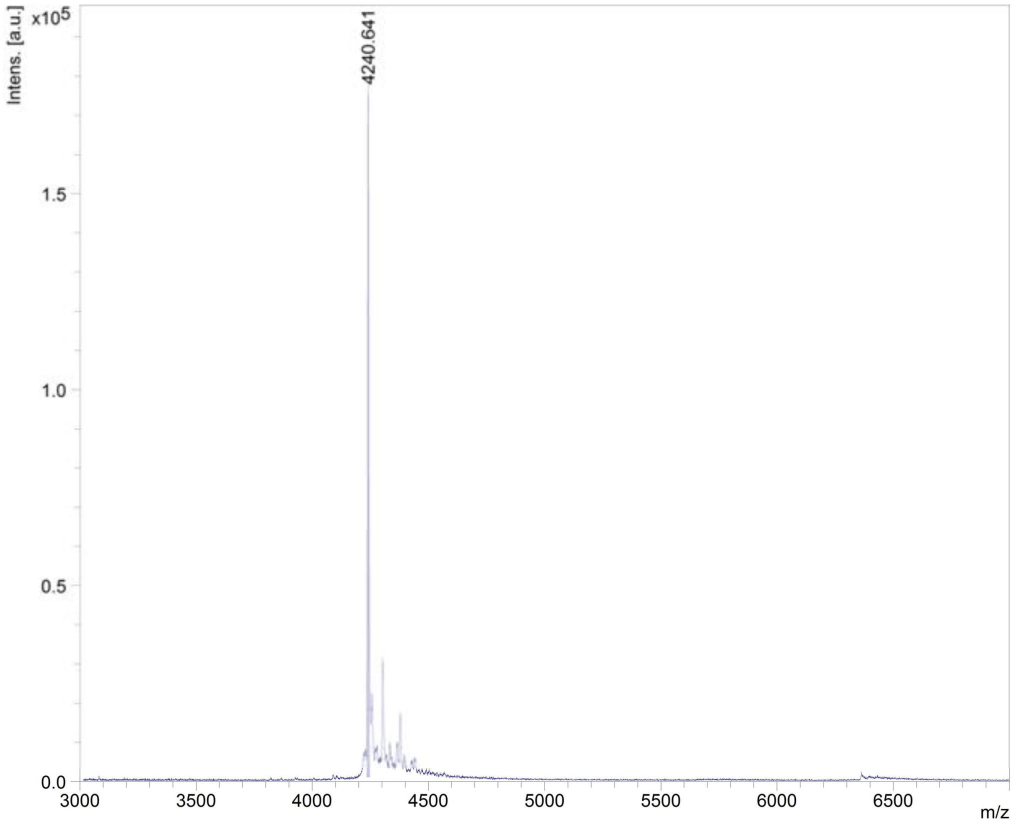

UD-36

[B conc. MeCN: 7-40% (30 min)]

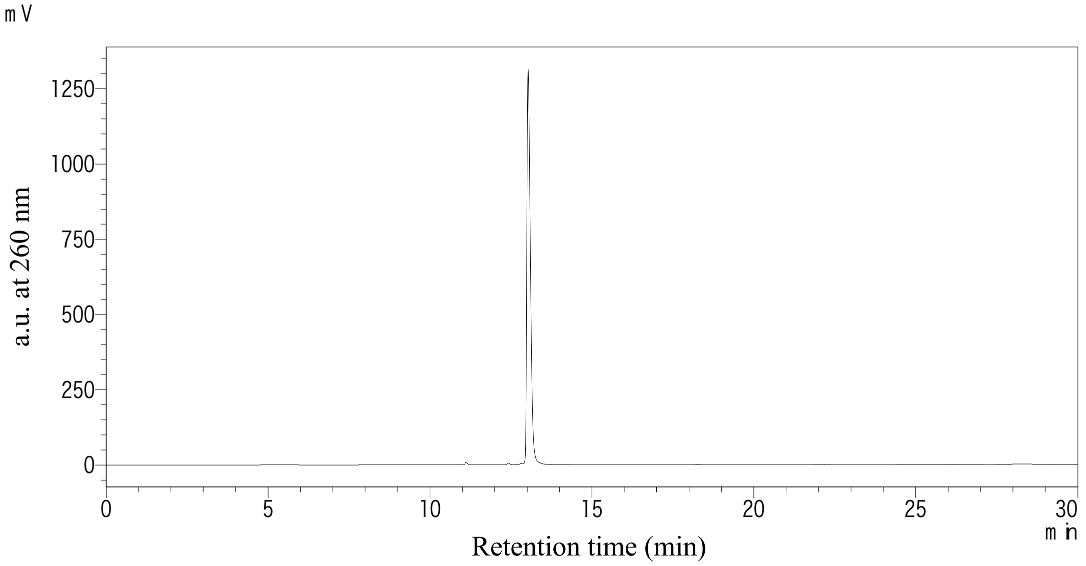

[calcd. (M-H)$^-$: 4208.1, found: 4209.0]

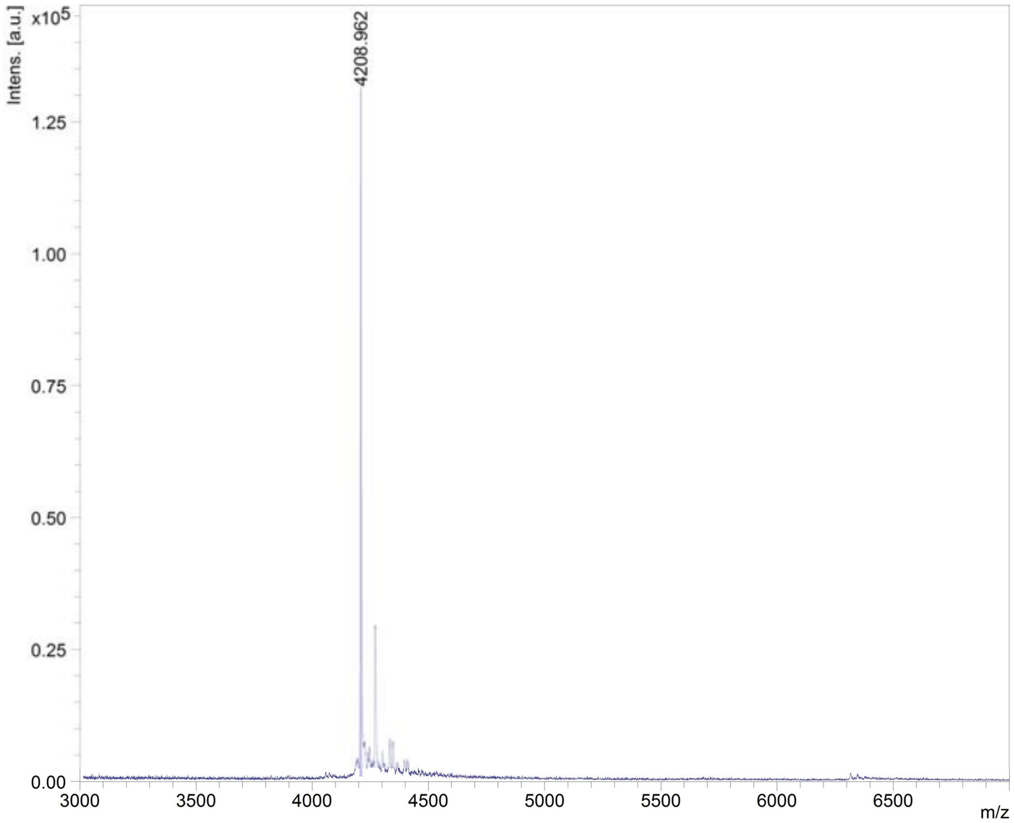

UD-37

[B conc. MeCN: 7-40% (30 min)]

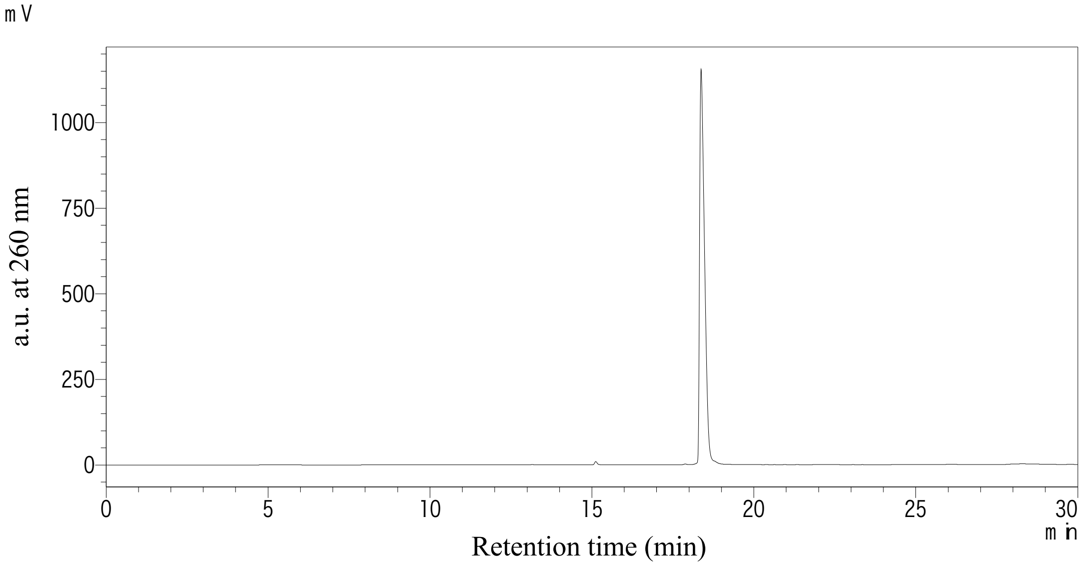

[calcd. (M-H)$^-$: 4218.1, found: 4218.6]

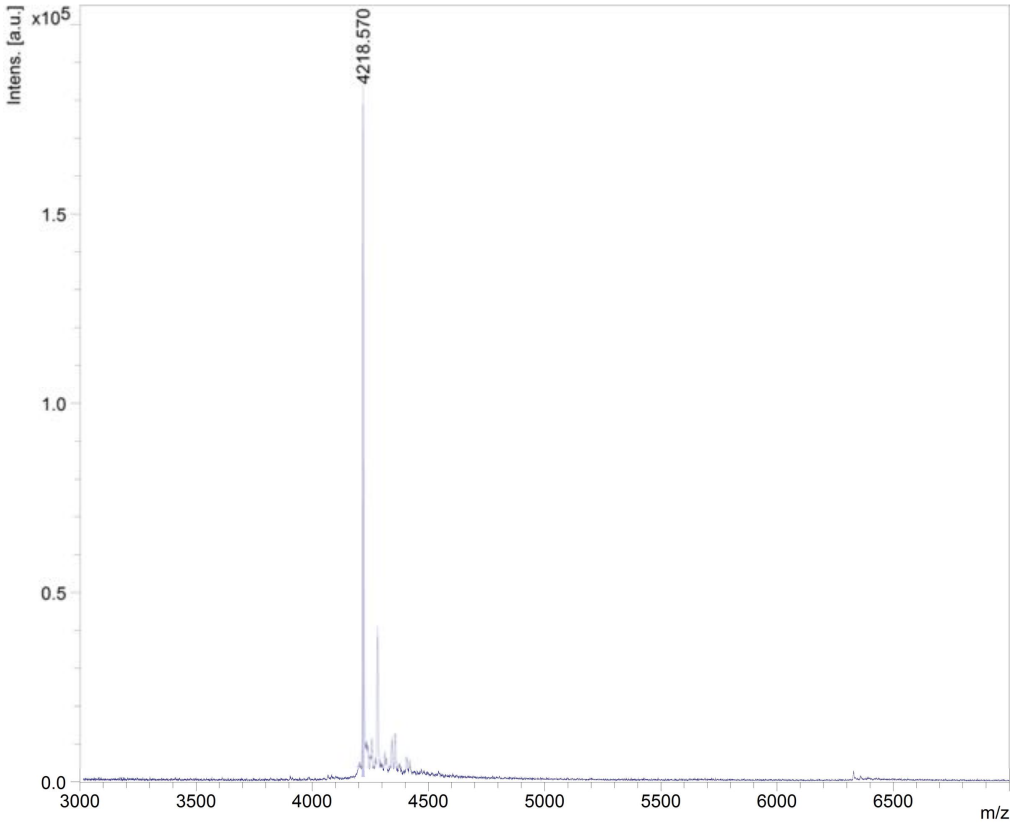

UD-38

[B conc. MeCN: 7-40% (30 min)]

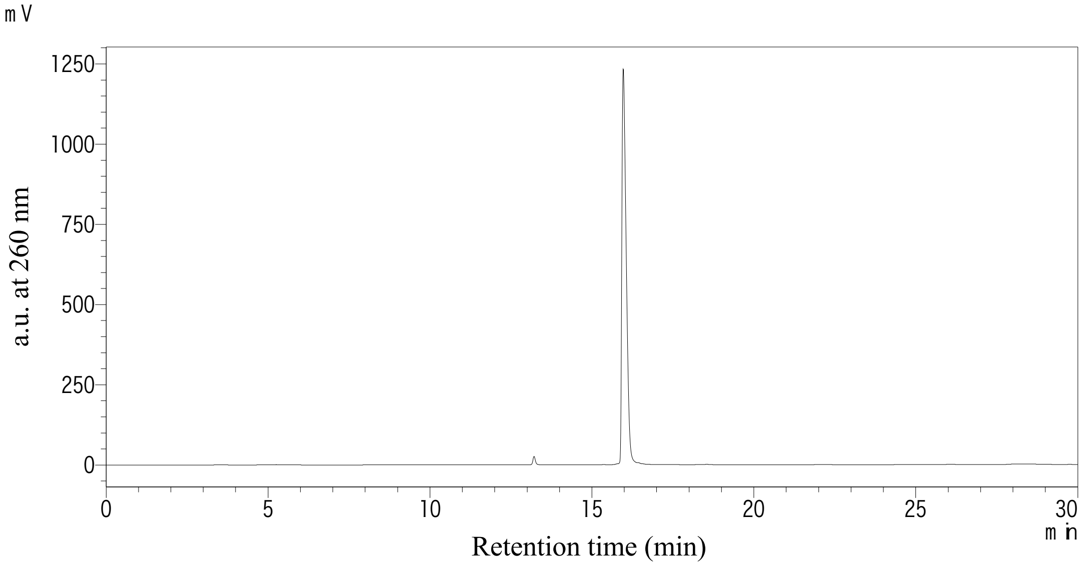

[calcd. (M-H)$^-$: 4192.1, found: 4192.6]

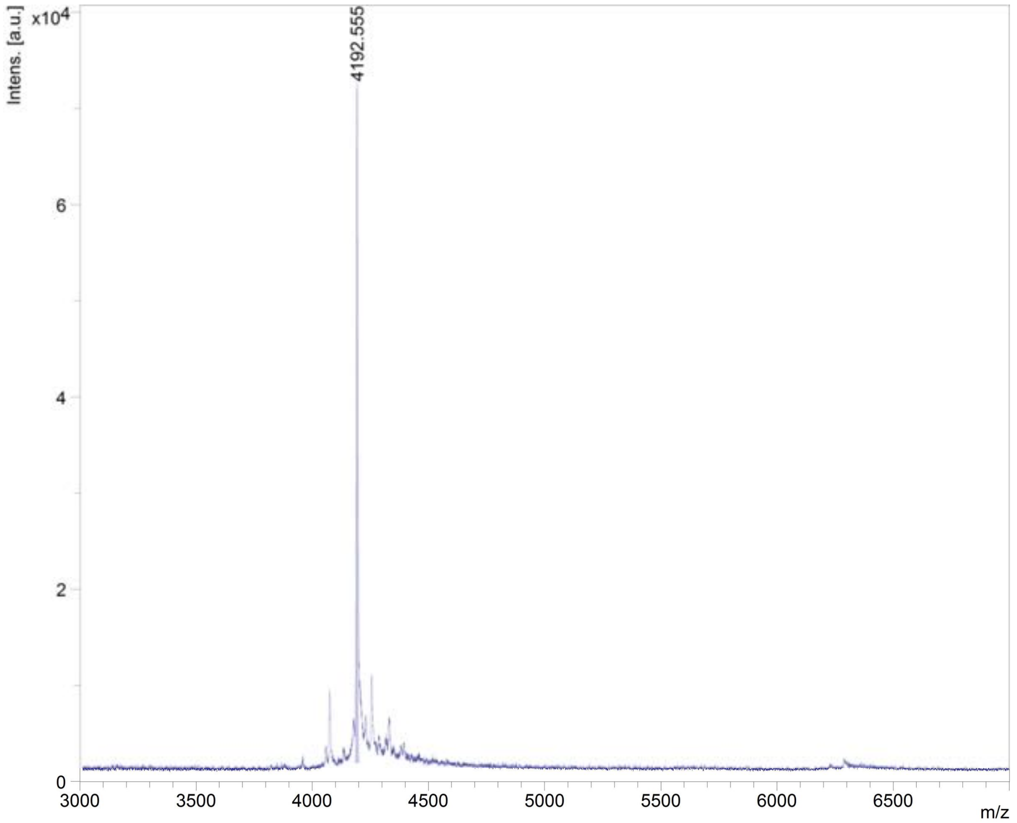

UD-39

[B conc. MeCN: 7-40% (30 min)]

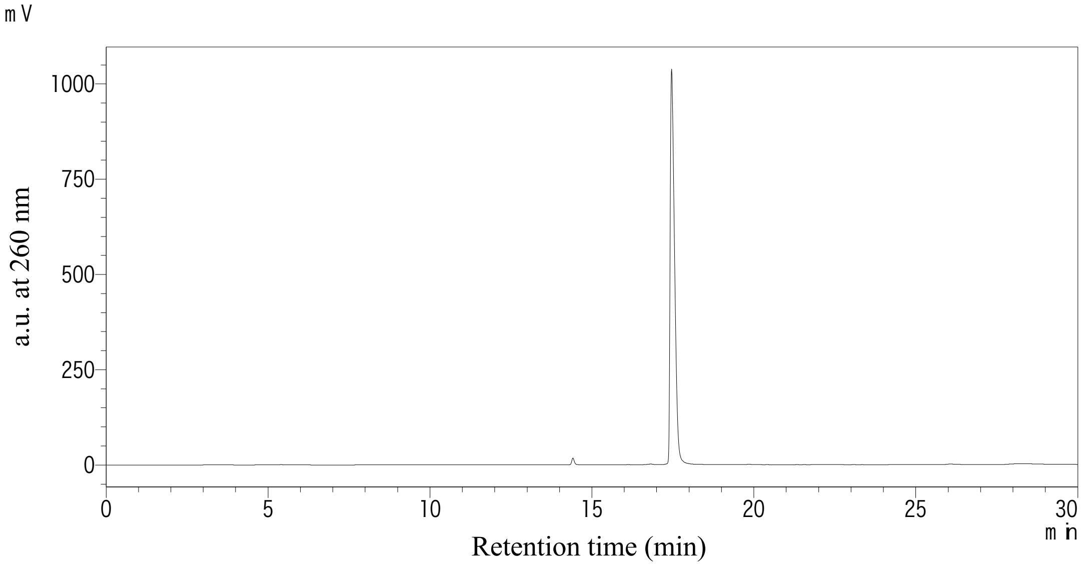

[calcd. (M-H)$^-$: 4226.1, found: 4226.3]

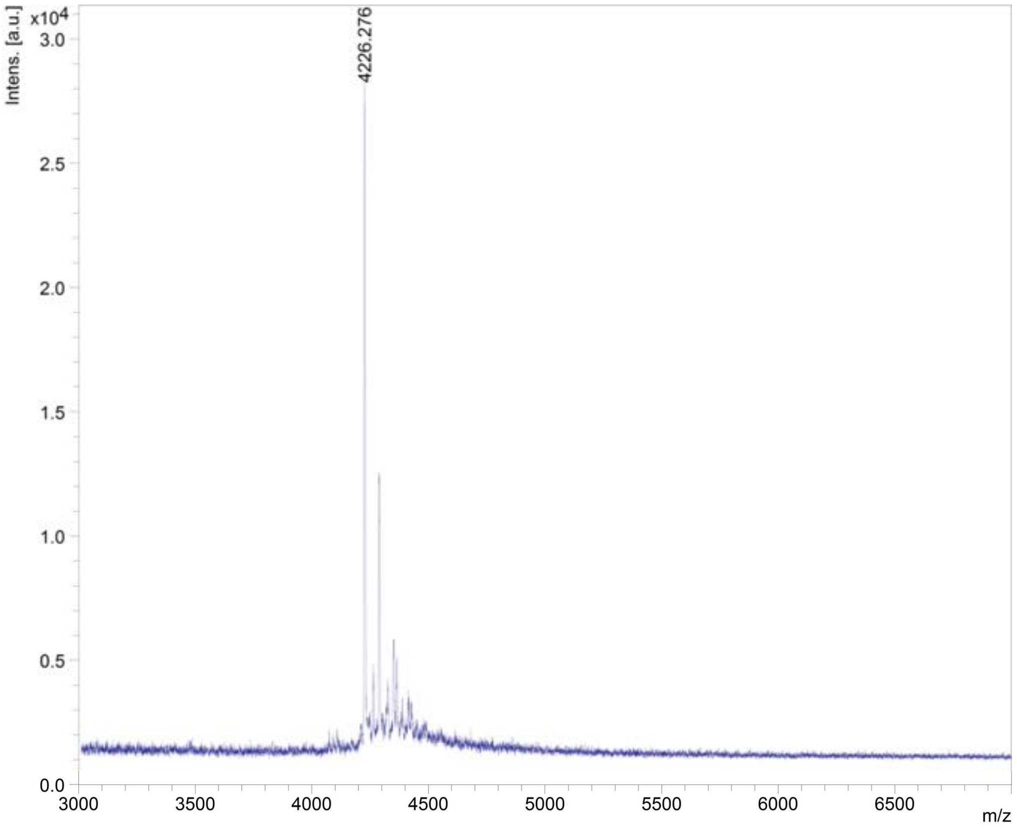

UD-40

[B conc. MeCN: 7-40% (30 min)]

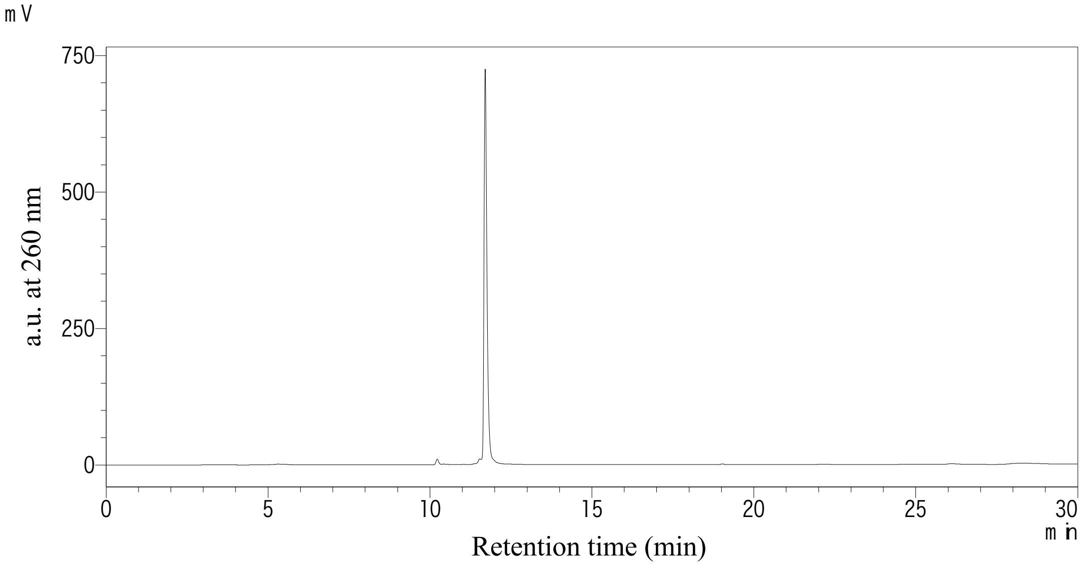

[calcd. (M-H)$^-$: 4235.1, found: 4235.1]

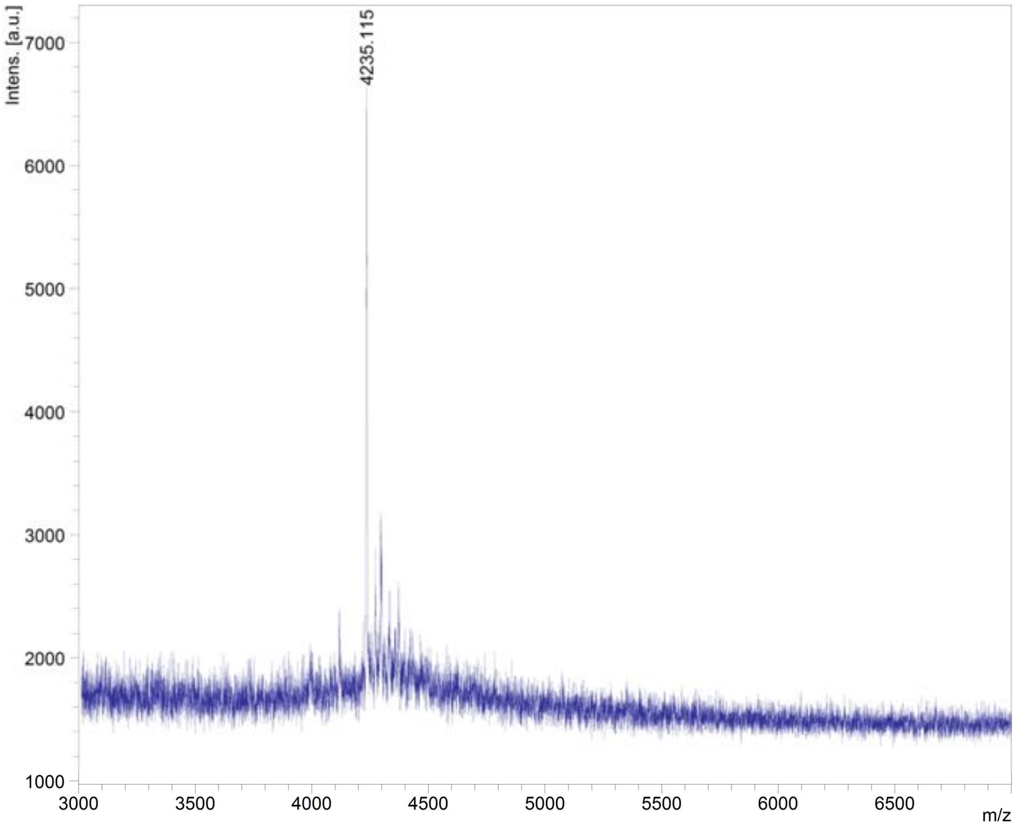

UD-41

[B conc. MeCN: 7-40% (30 min)]

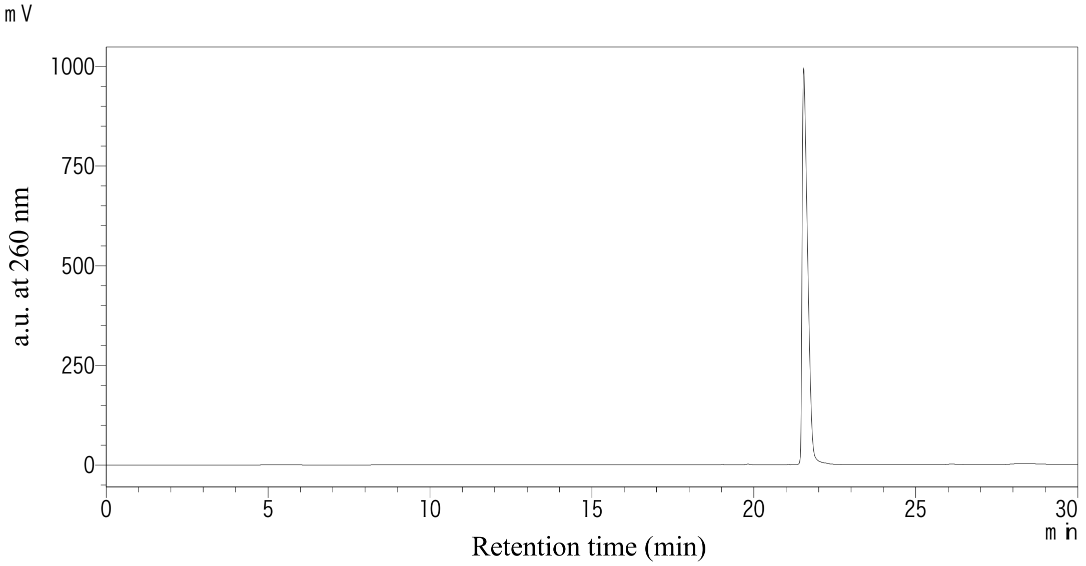

[calcd. (M-H)$^{-}$: 4202.1, found: 4202.1]

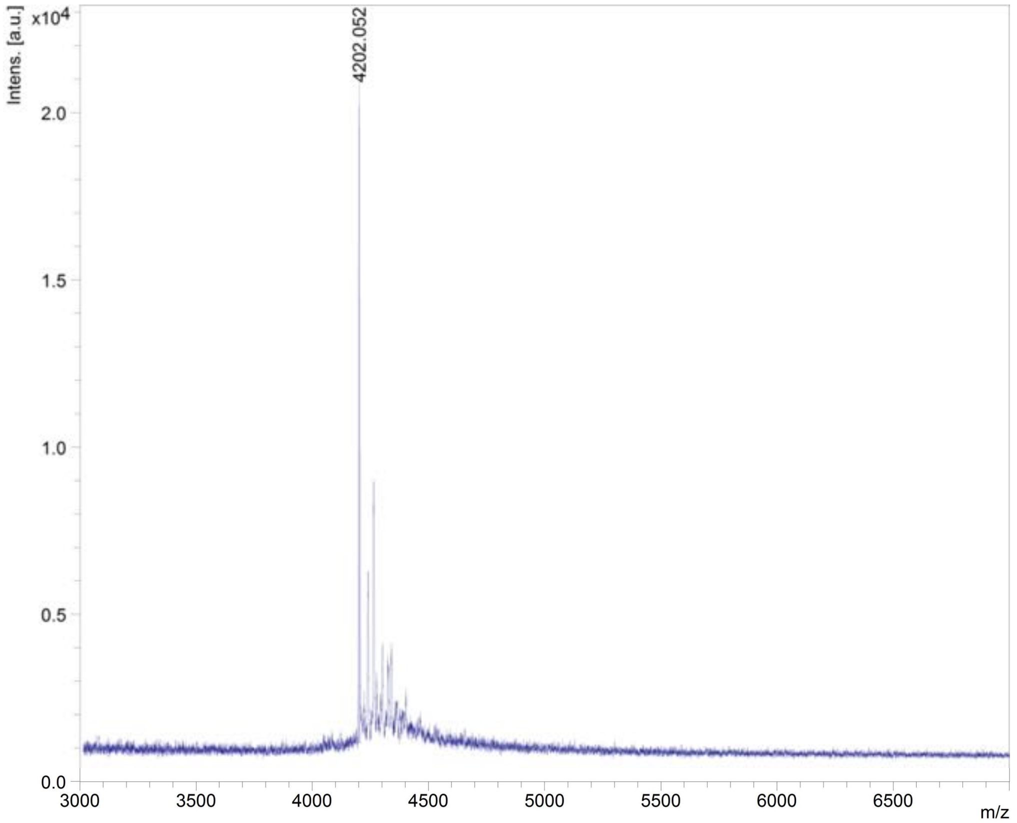

UD-42

[B conc. MeCN: 7-40% (30 min)]

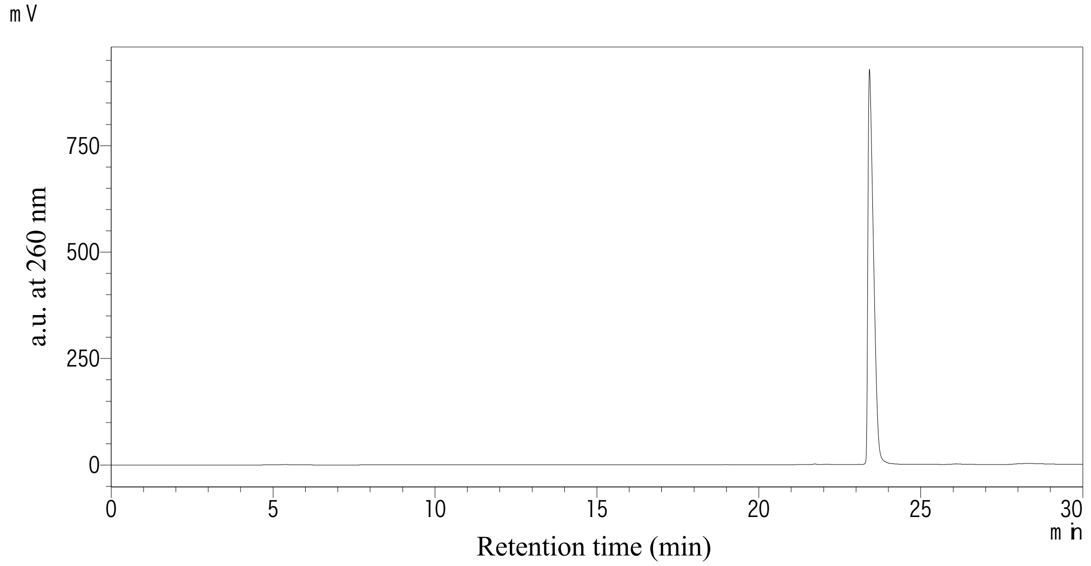

[calcd. (M-H)$^-$: 4250.2, found: 4251.2]

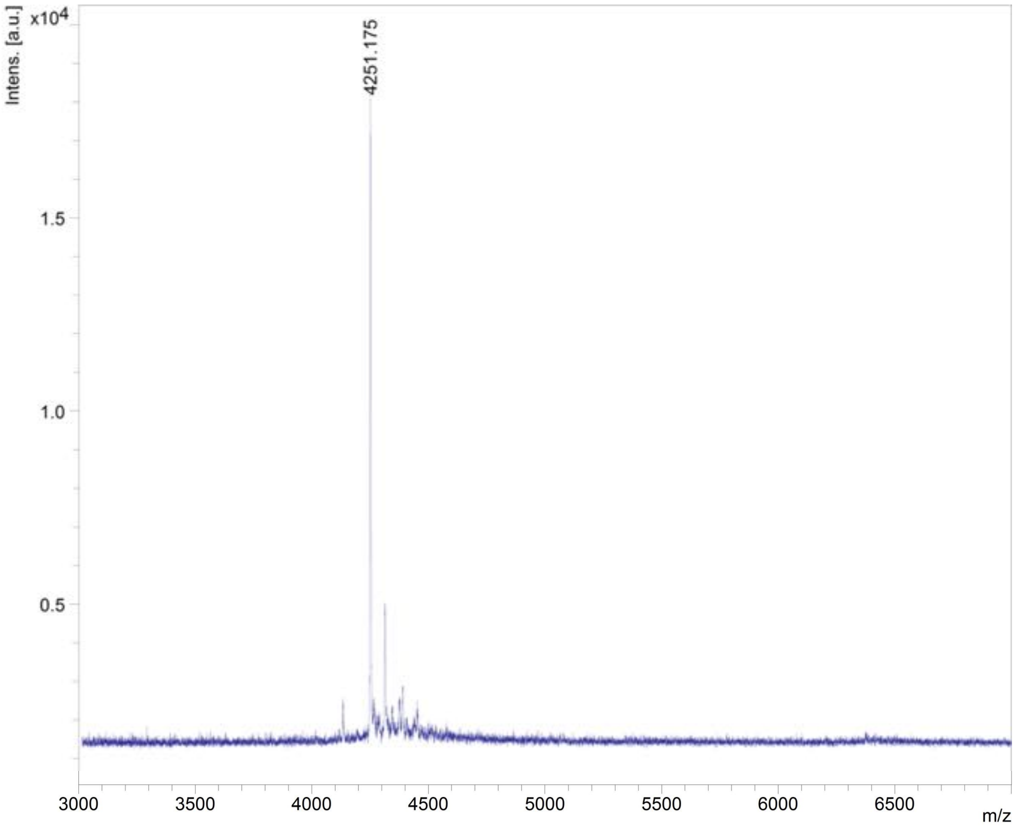

UD-43

[B conc. MeCN: 7-40% (30 min)]

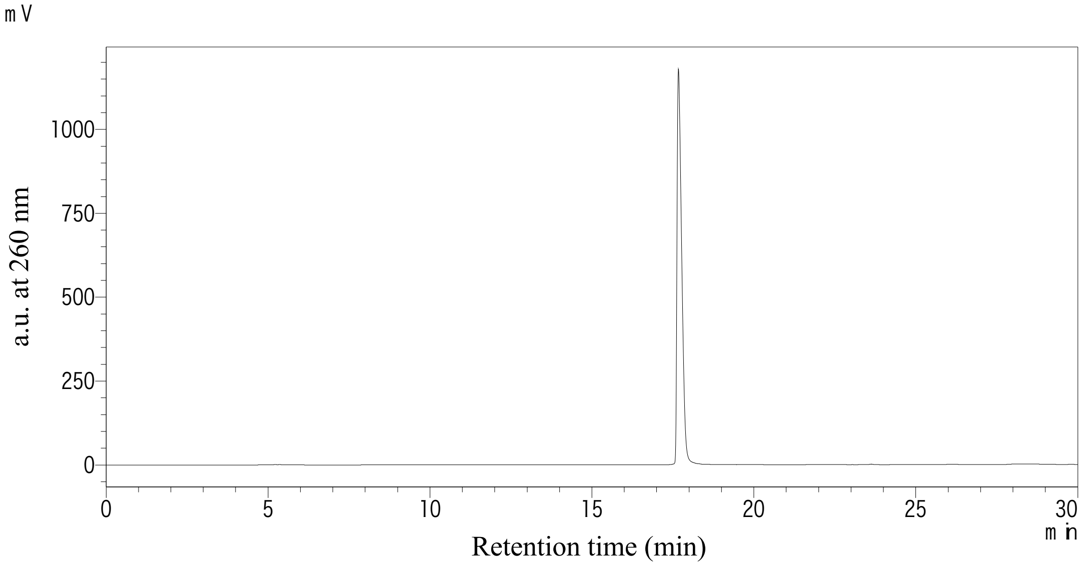

[calcd. (M-H)$^{-}$: 4218.1, found: 4218.8]

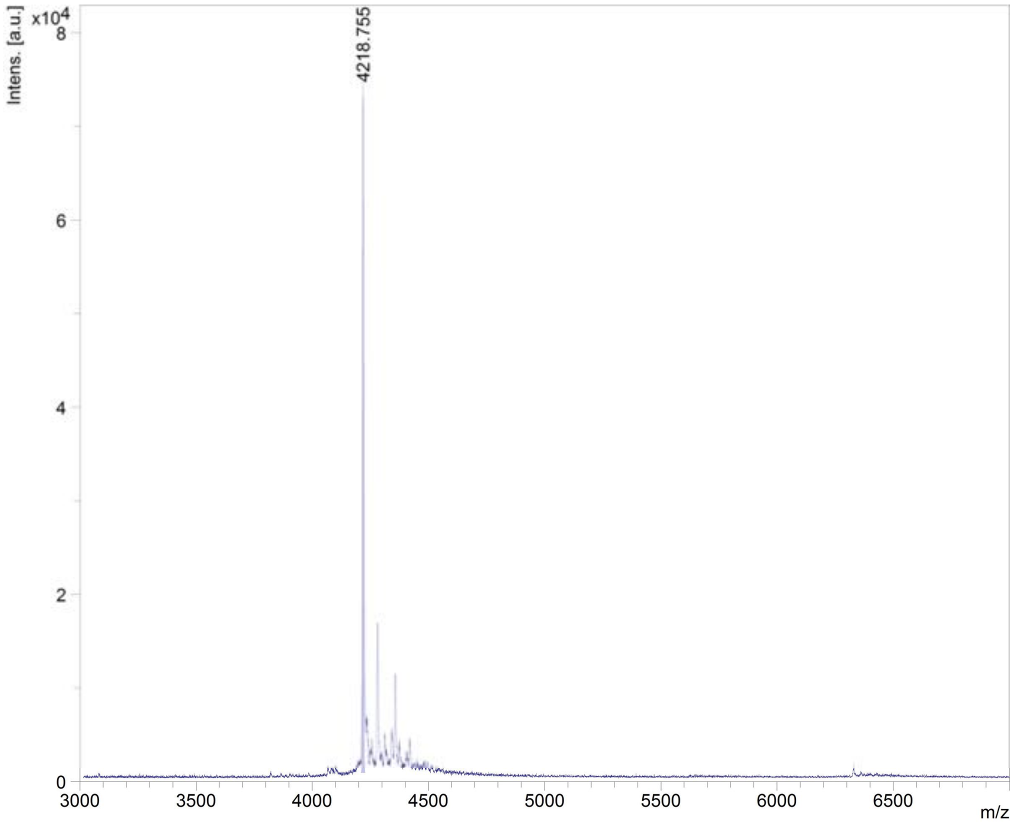

UD-44

[B conc. MeCN: 7-40% (30 min)]

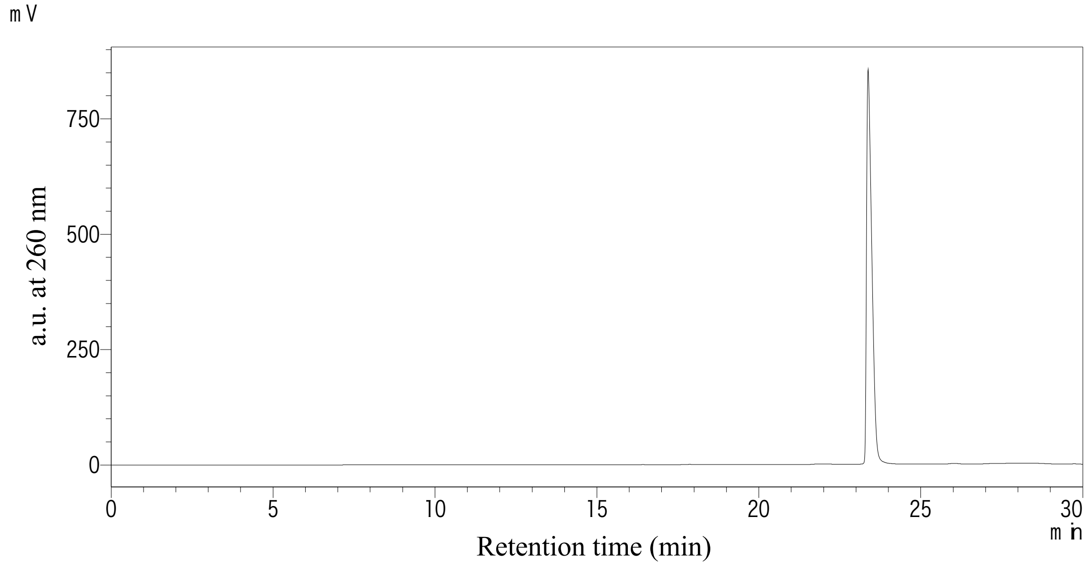

[calcd. (M-H)$^{-}$: 4228.2, found: 4228.2]

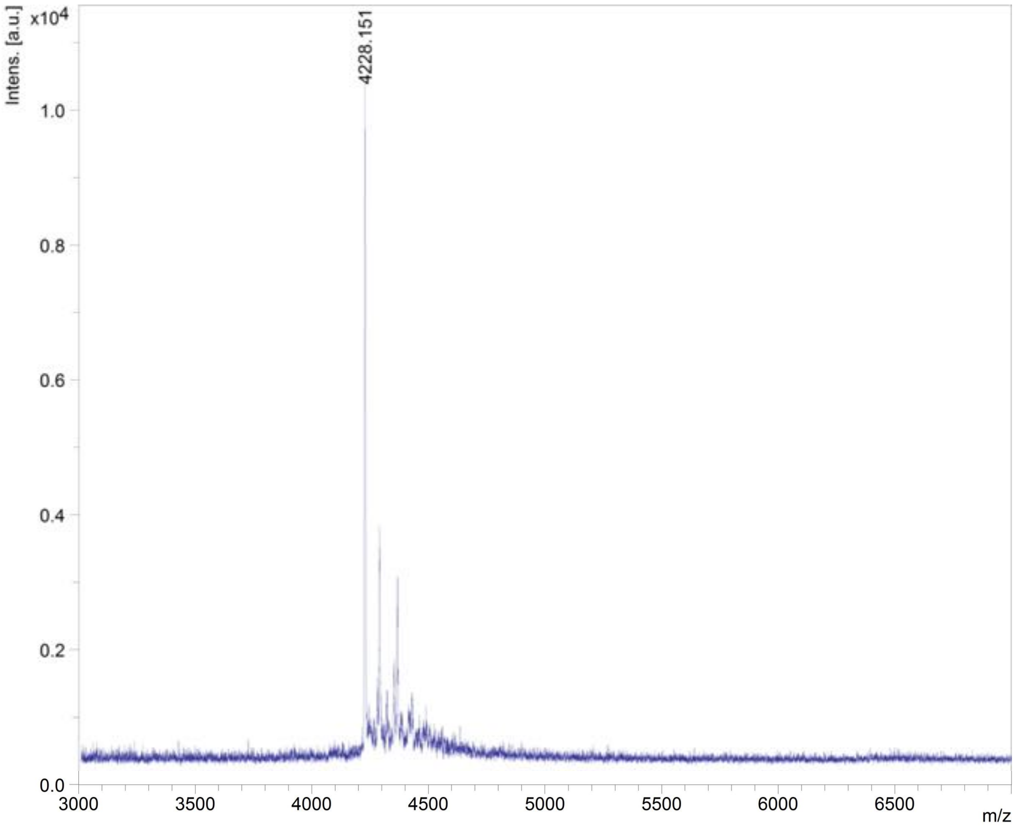

UD-45

[B conc. MeCN: 7-40% (30 min)]

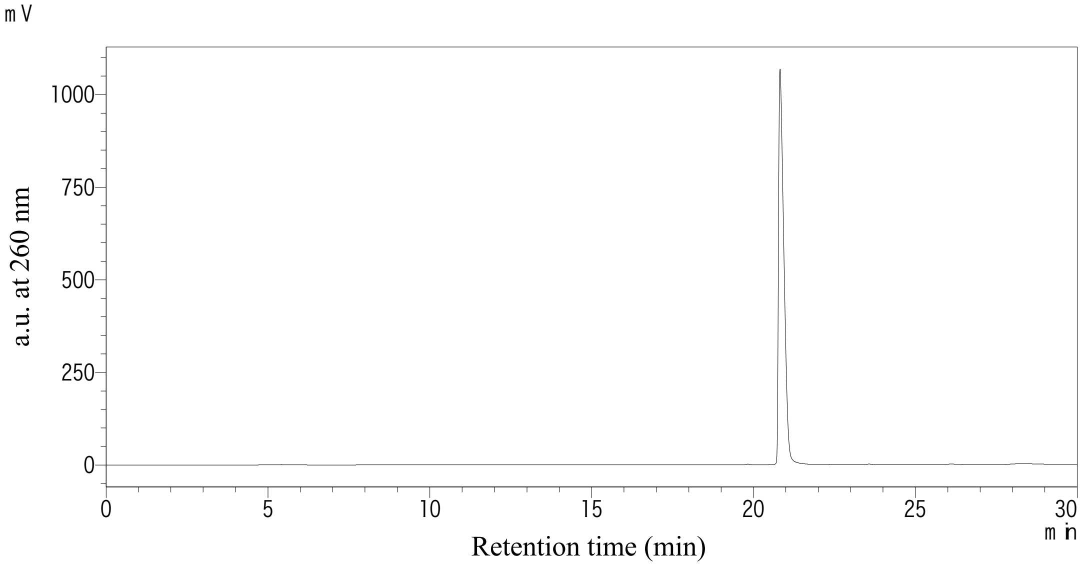

[calcd. (M-H)$^{-}$: 4202.1, found: 4202.6]

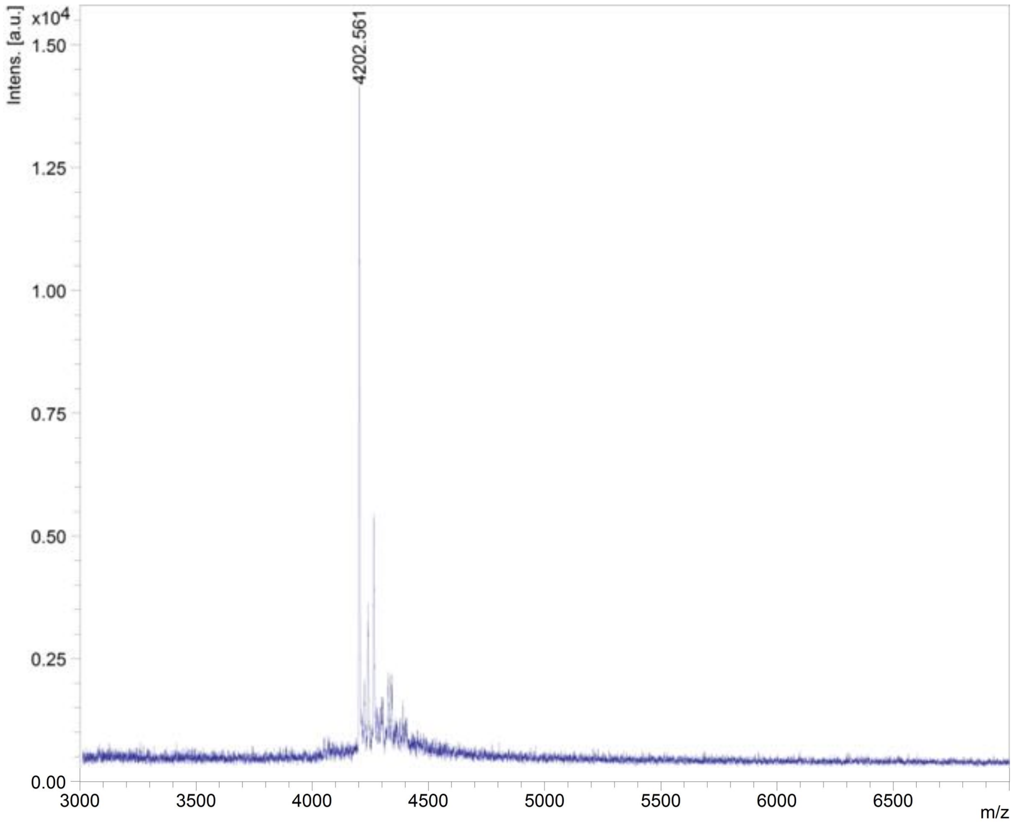

UD-46

[B conc. MeCN: 7-40% (30 min)]

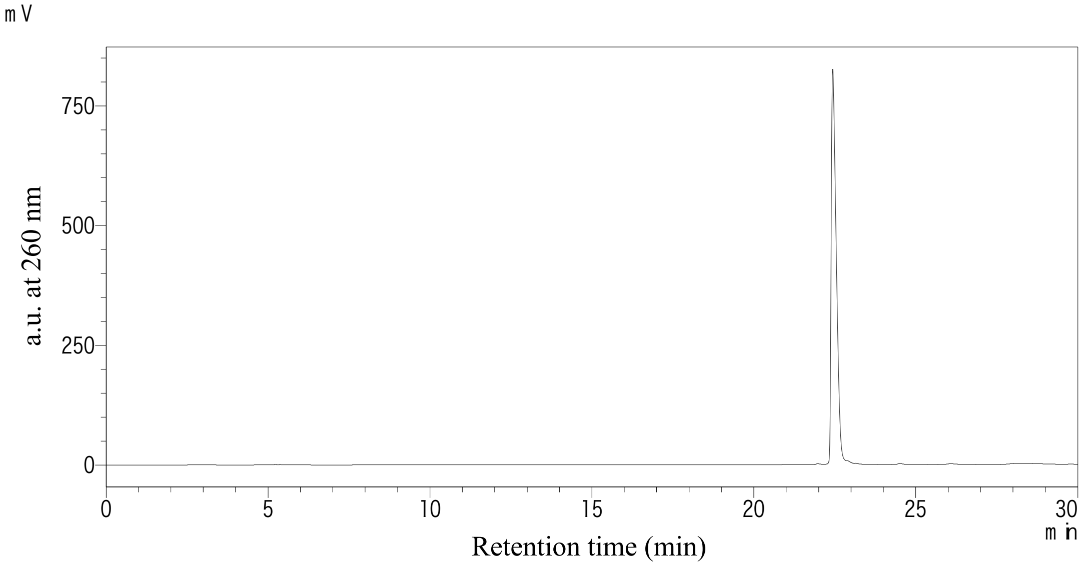

[calcd. $(M\text{-}H)^{-}$: 4236.1, found: 4237.9]

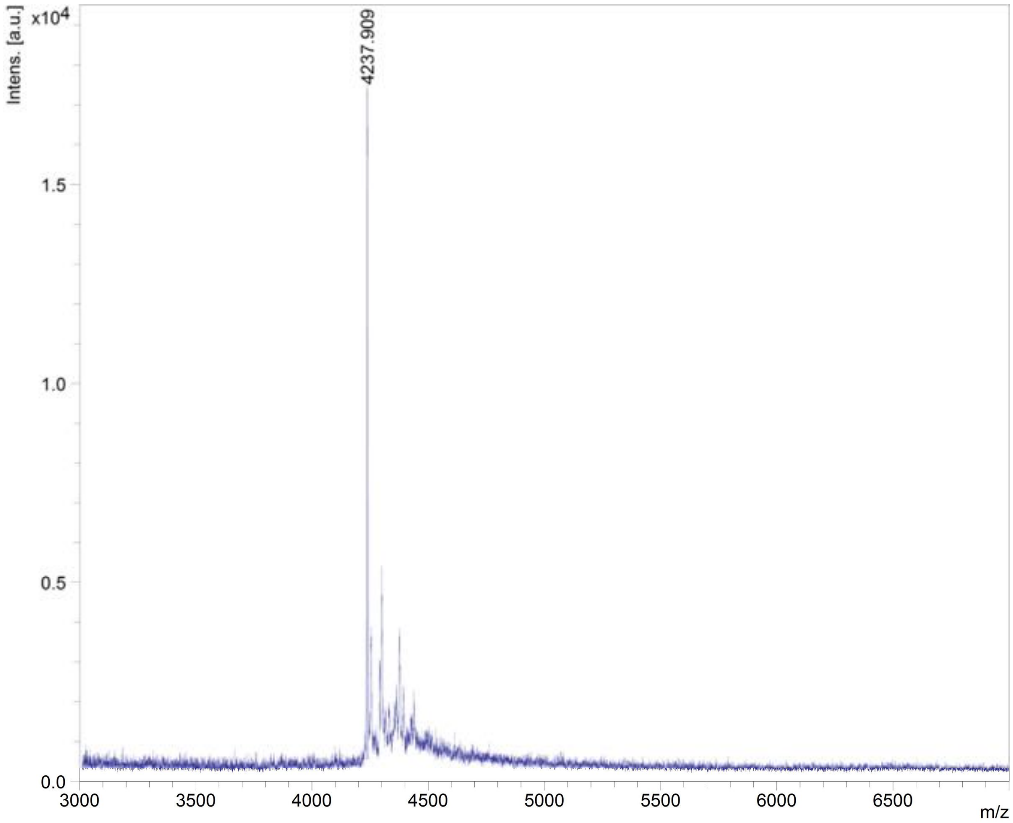

UD-47

[B conc. MeCN: 7-40% (30 min)]

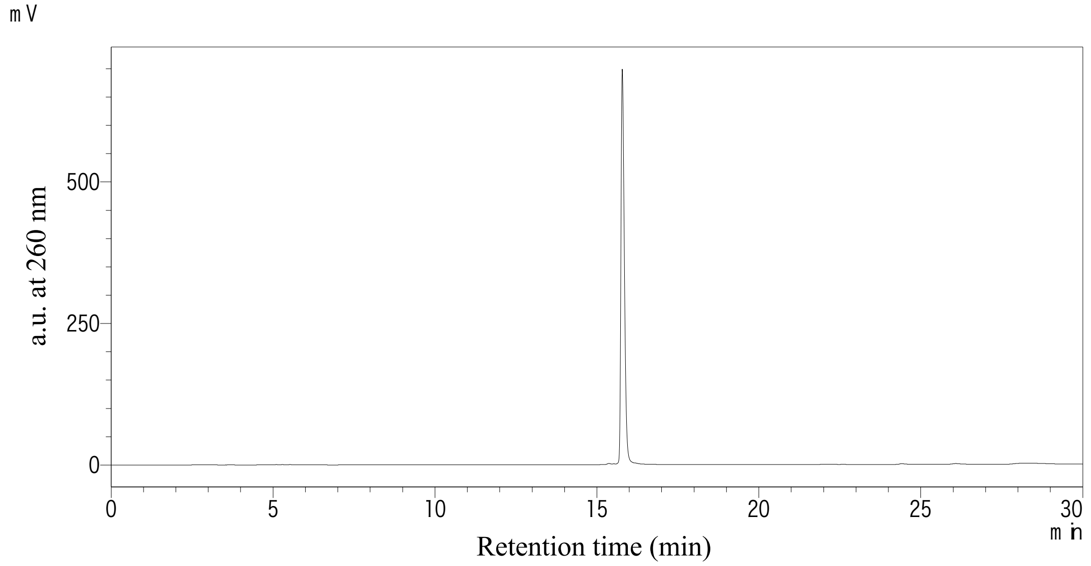

[calcd. (M-H)$^{-}$: 4245.2, found: 4245.6]

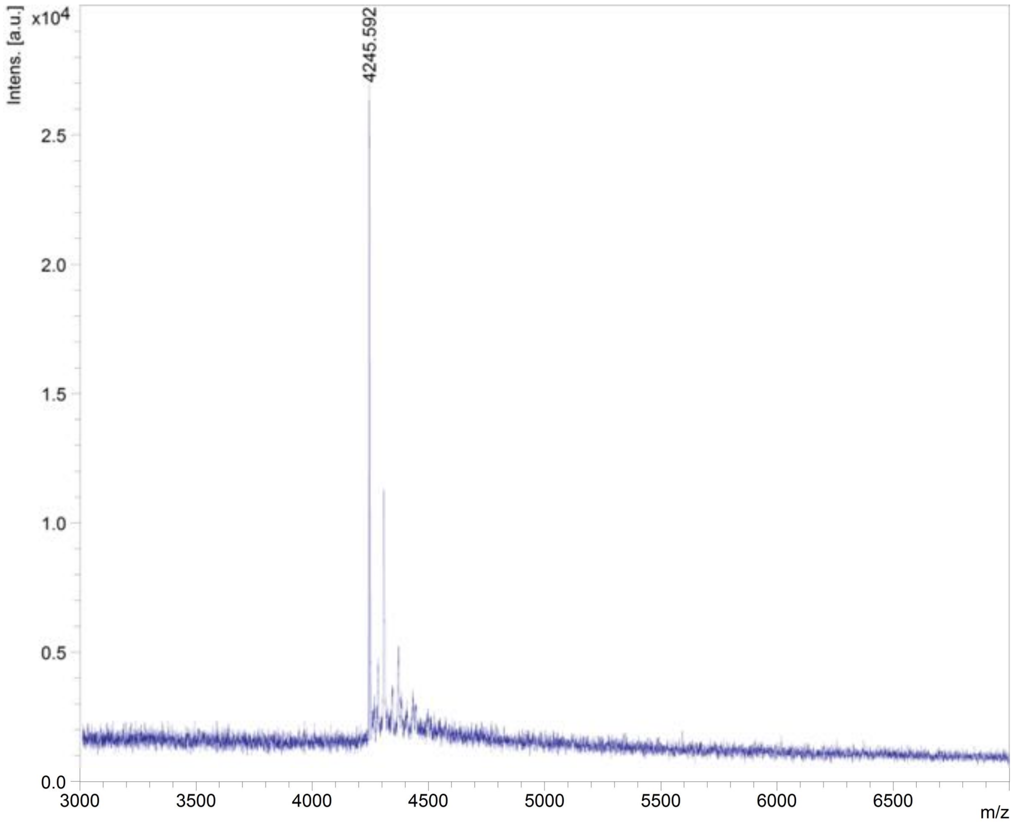

UD-48

[B conc. MeCN: 7-40% (30 min)]

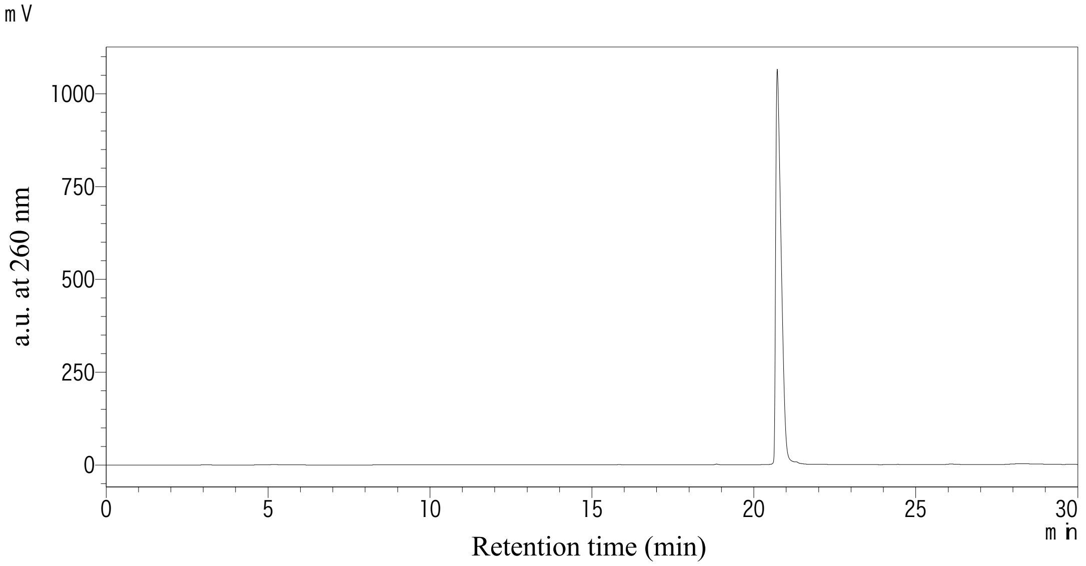

[calcd. (M-H)$^-$: 4210.1, found: 4211.4]

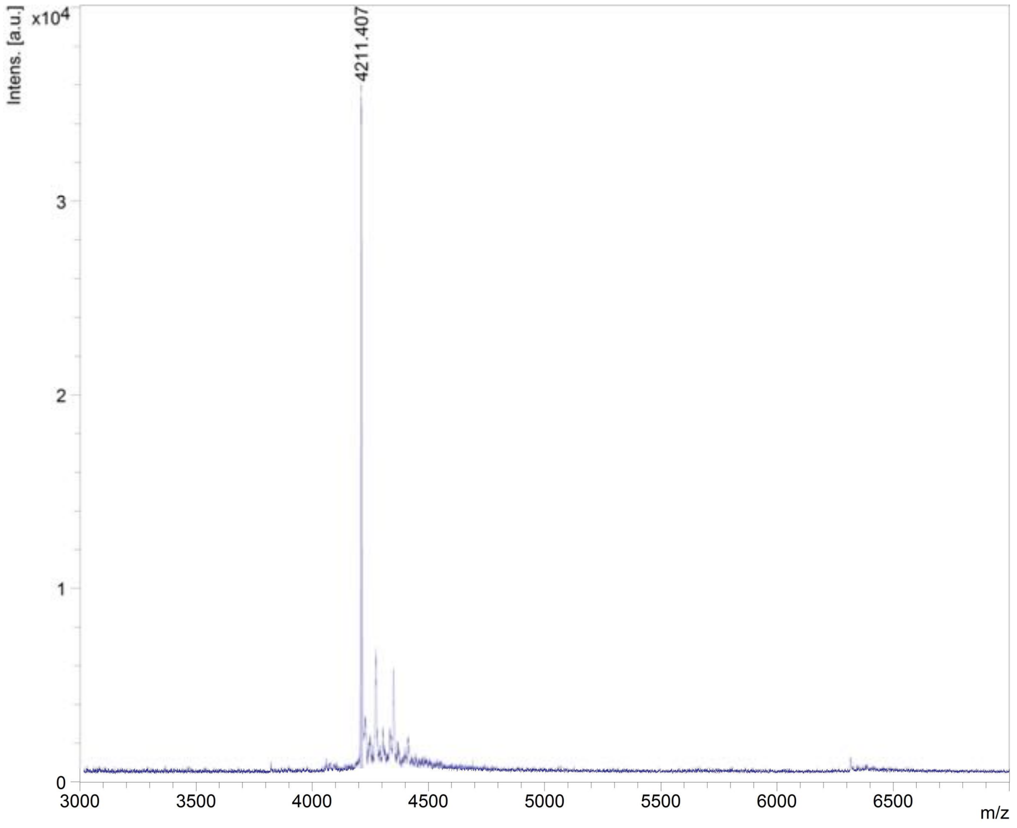

UD-49

[B conc. MeCN: 7-40% (30 min)]

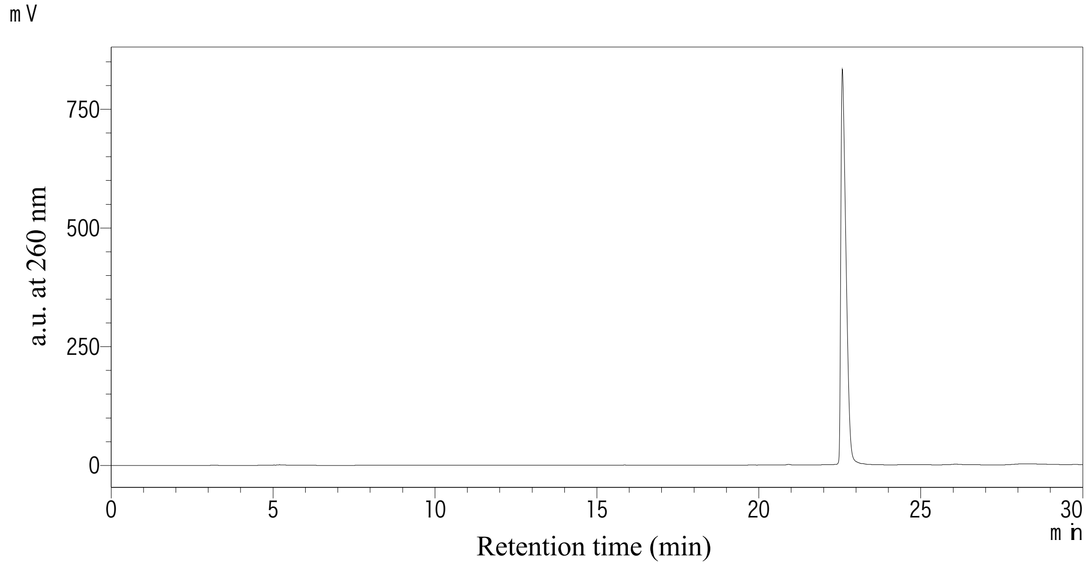

[calcd. (M-H)$^{-}$: 4258.2, found: 4258.4]

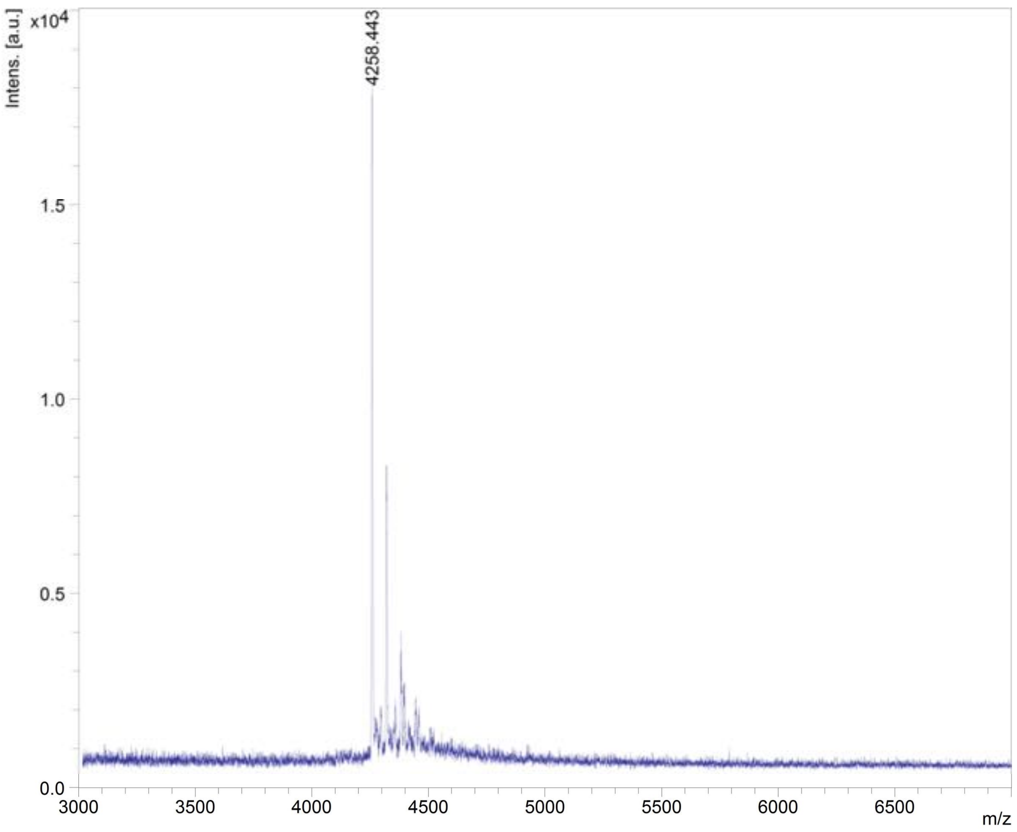

UD-50

[B conc. MeCN: 7-40% (30 min)]

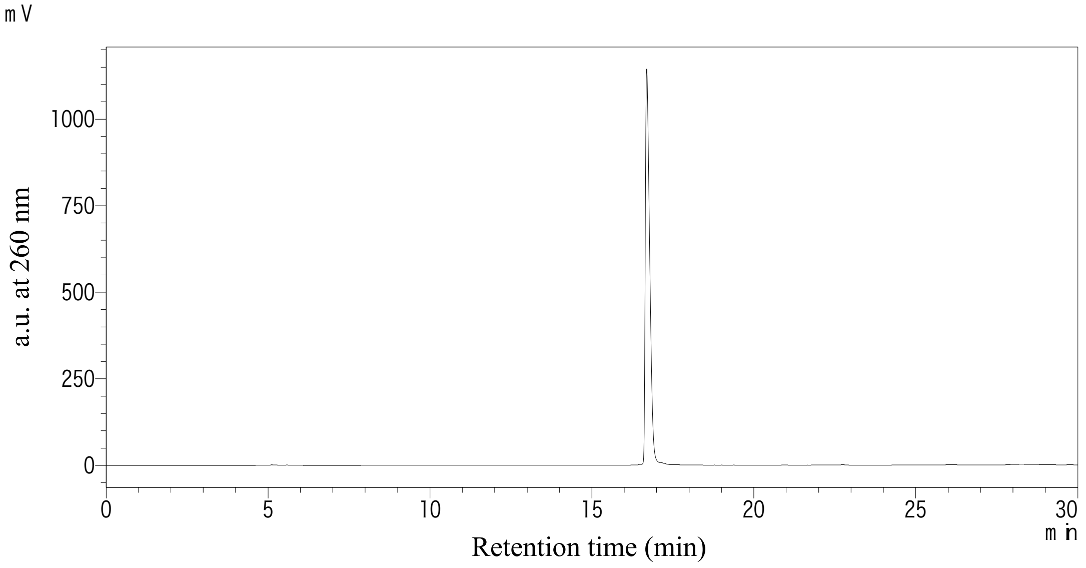

[calcd. (M-H)$^{-}$: 4226.1, found: 4226.8]

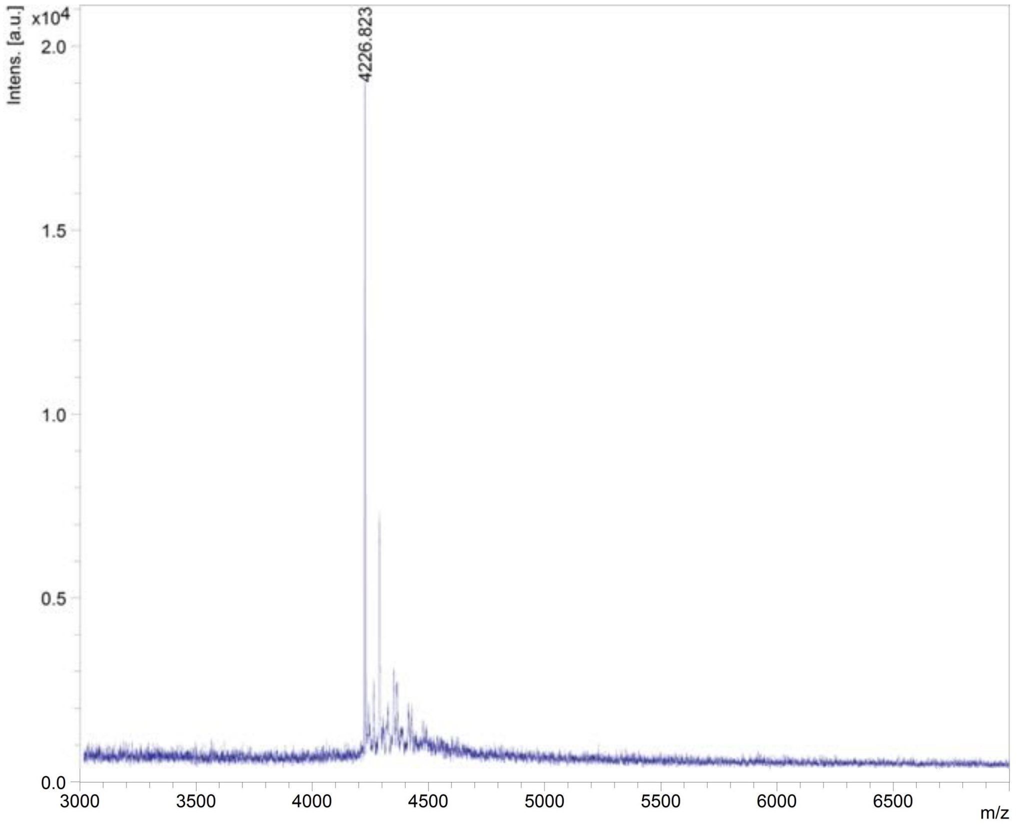

UD-51

[B conc. MeCN: 7-40% (30 min)]

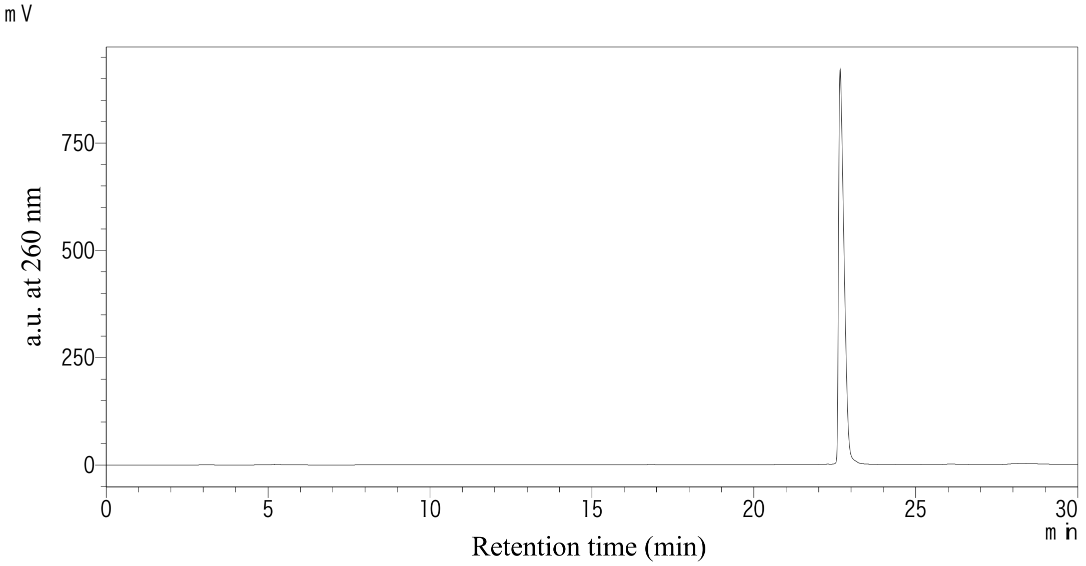

[calcd. (M-H)$^{-}$: 4236.1, found: 4236.5]

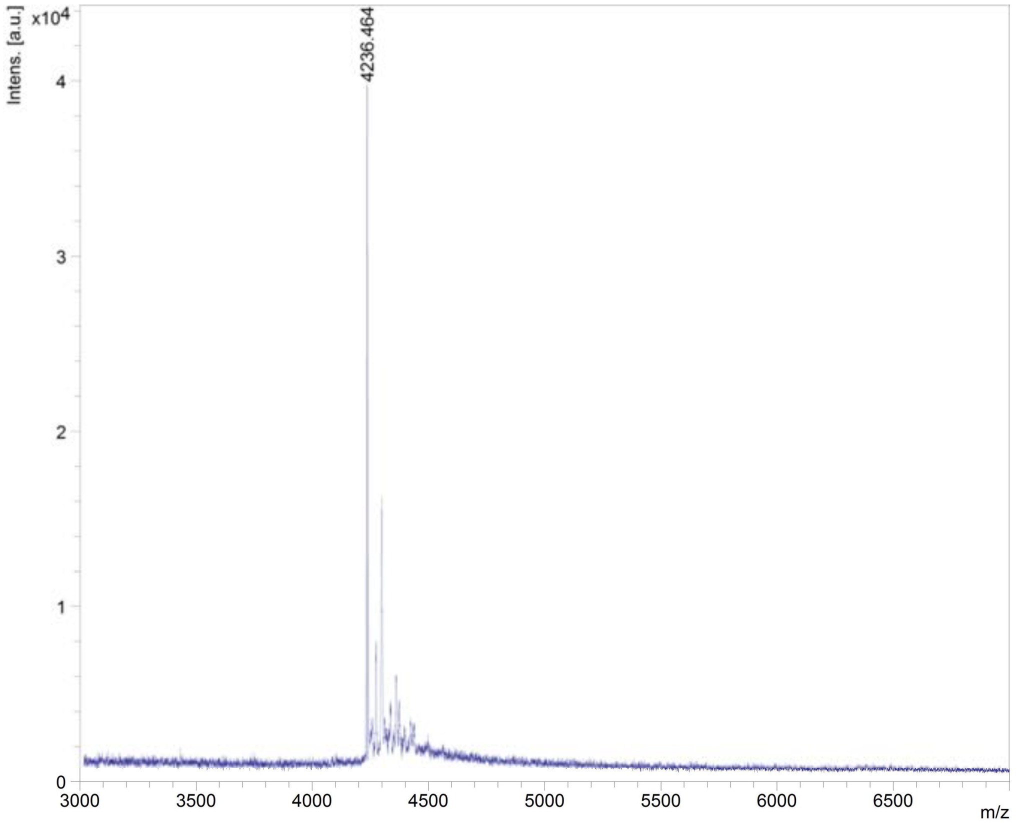

UD-52

[B conc. MeCN: 7-40% (30 min)]

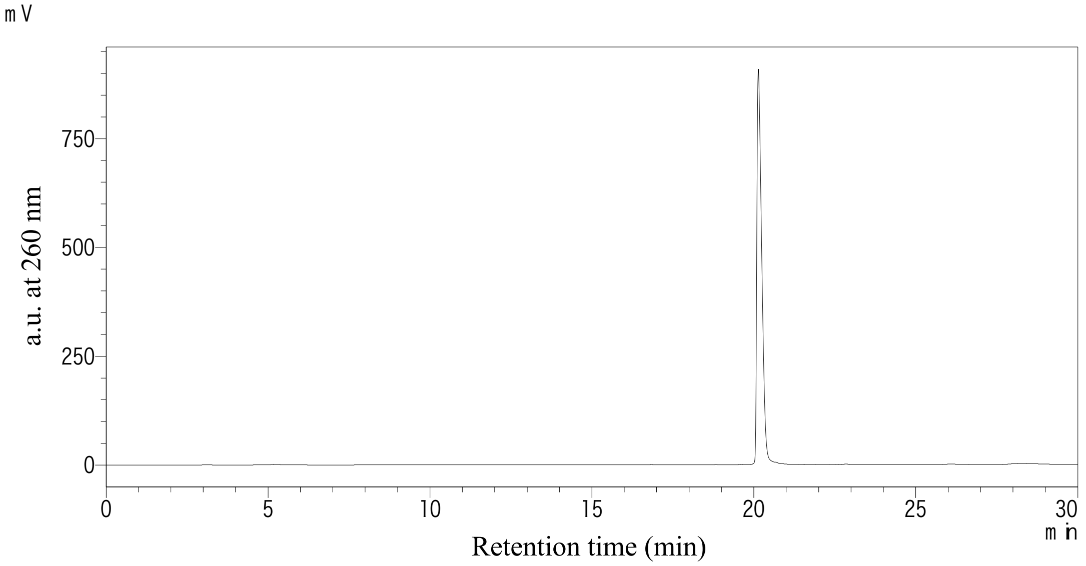

[calcd. $(M\text{-}H)^{-}$: 4210.1, found: 4210.7]

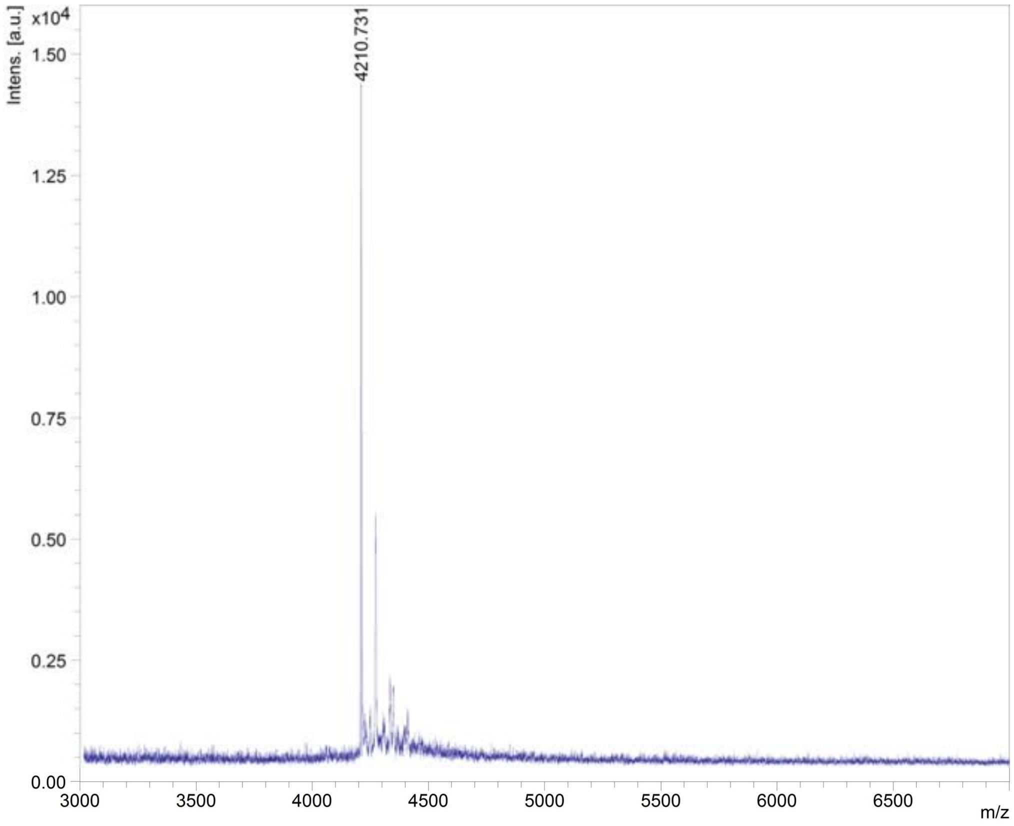

UD-53

[B conc. MeCN: 7-40% (30 min)]

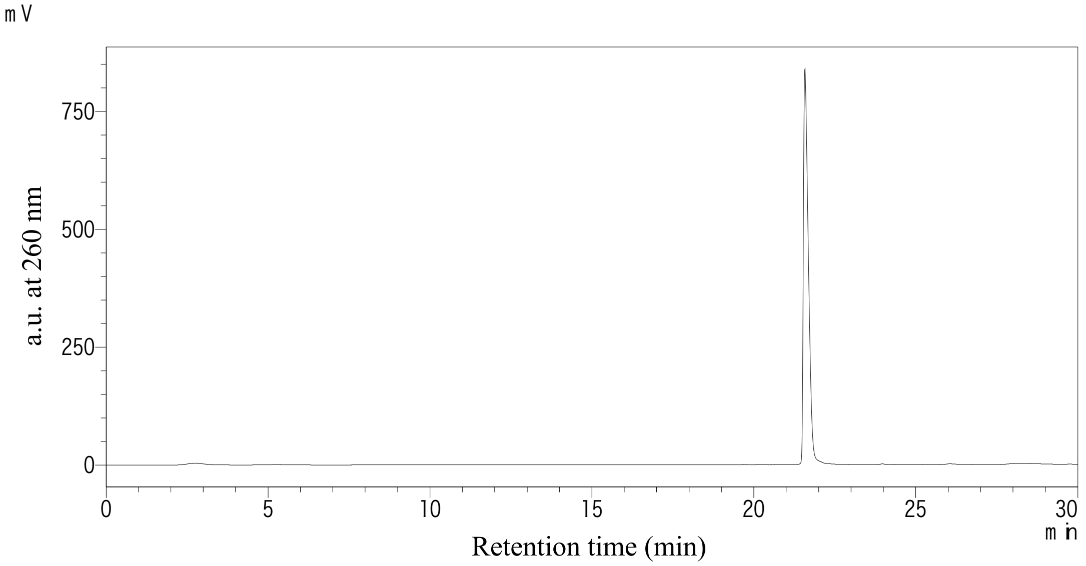

[calcd. (M-H)$^{-}$: 4244.1, found: 4244.6]

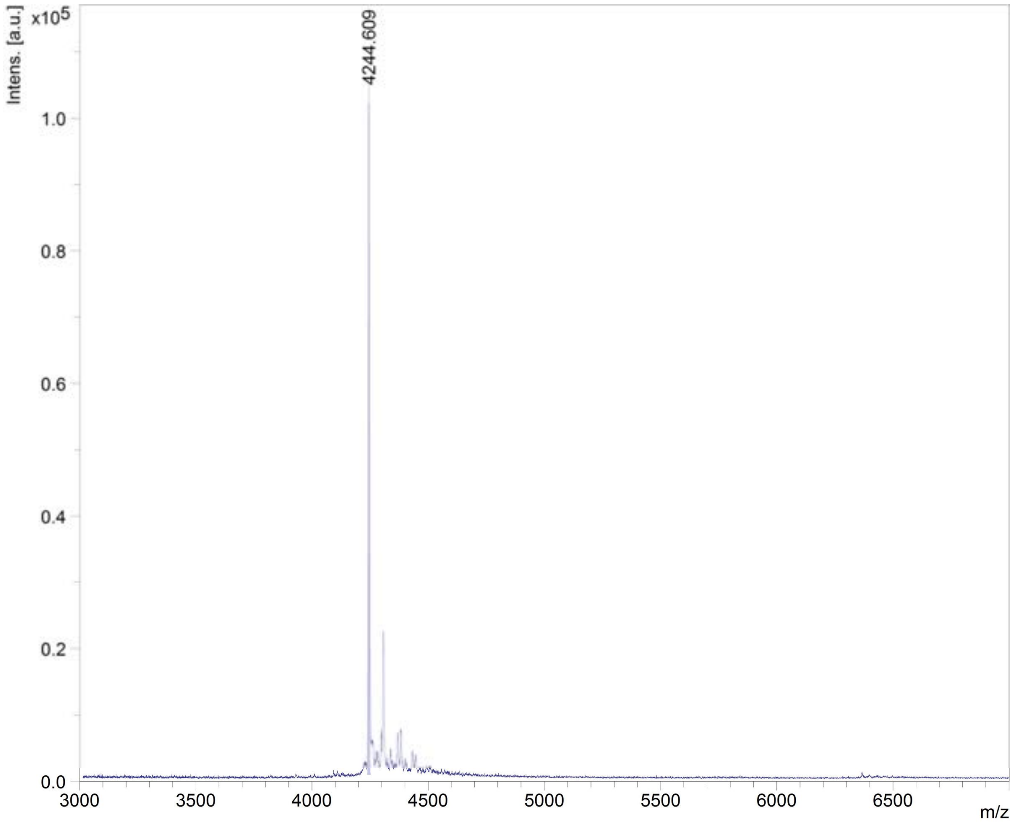

UD-54

[B conc. MeCN: 7-40% (30 min)]

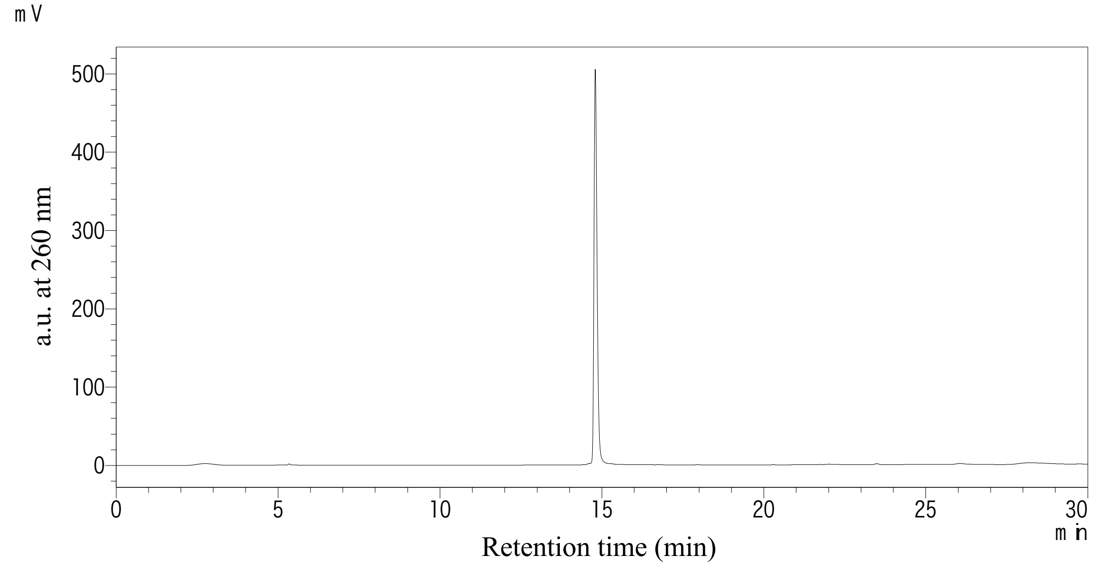

[calcd. (M-H)$^{-}$: 4253.2, found: 4253.3]

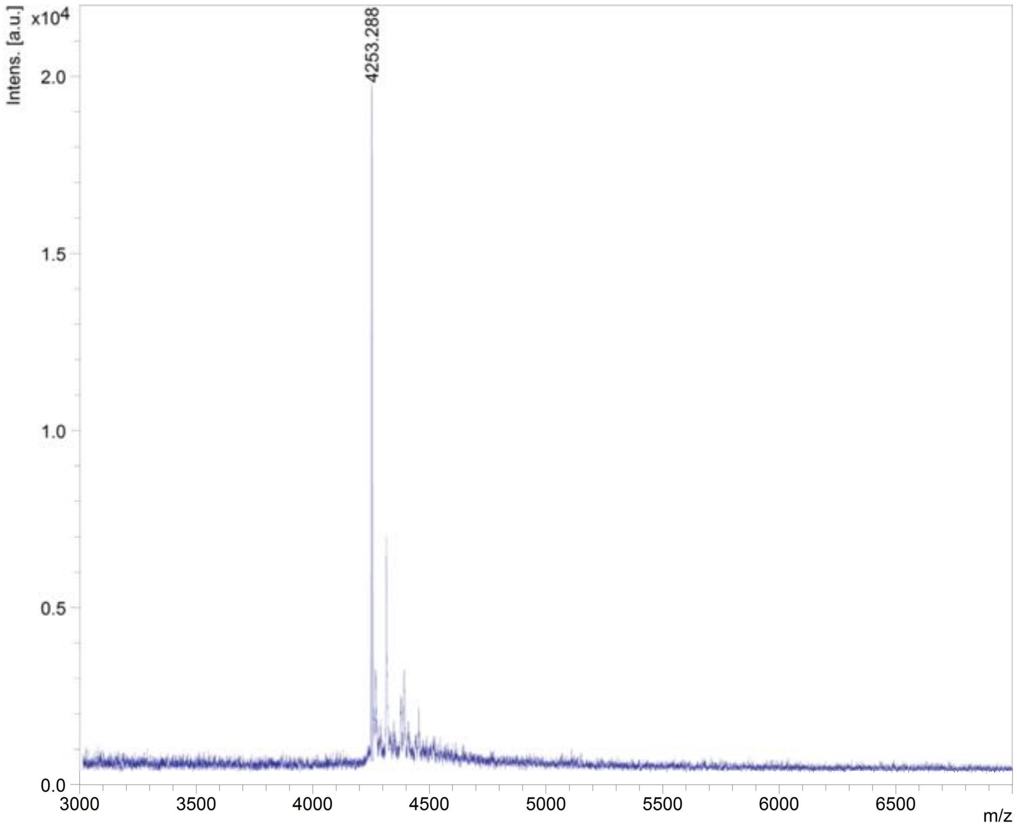

UD-55

[B conc. MeCN: 7-40% (30 min)]

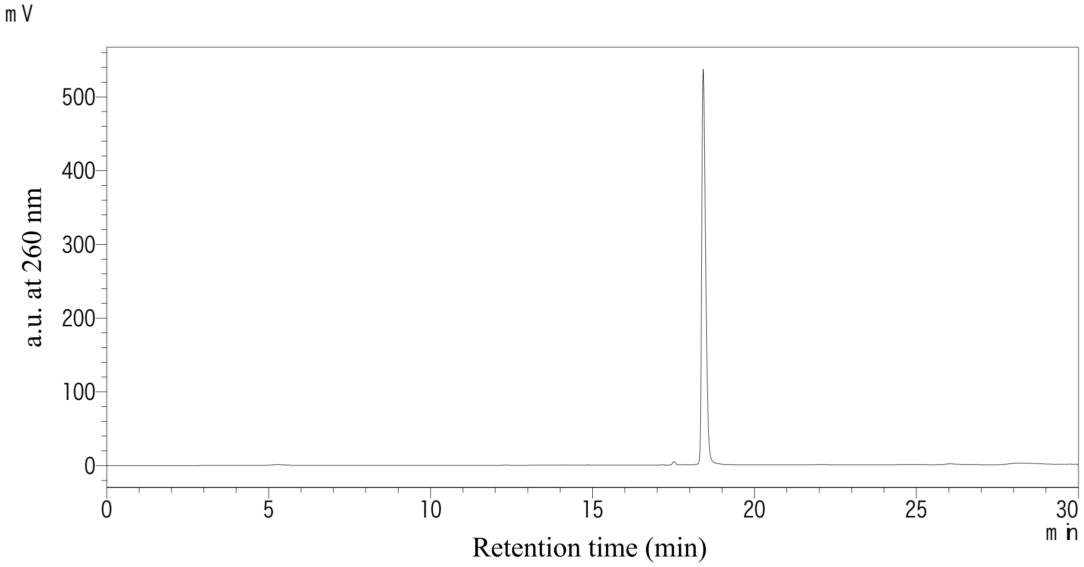

[calcd. (M-H)$^-$: 4146.0, found: 4146.2]

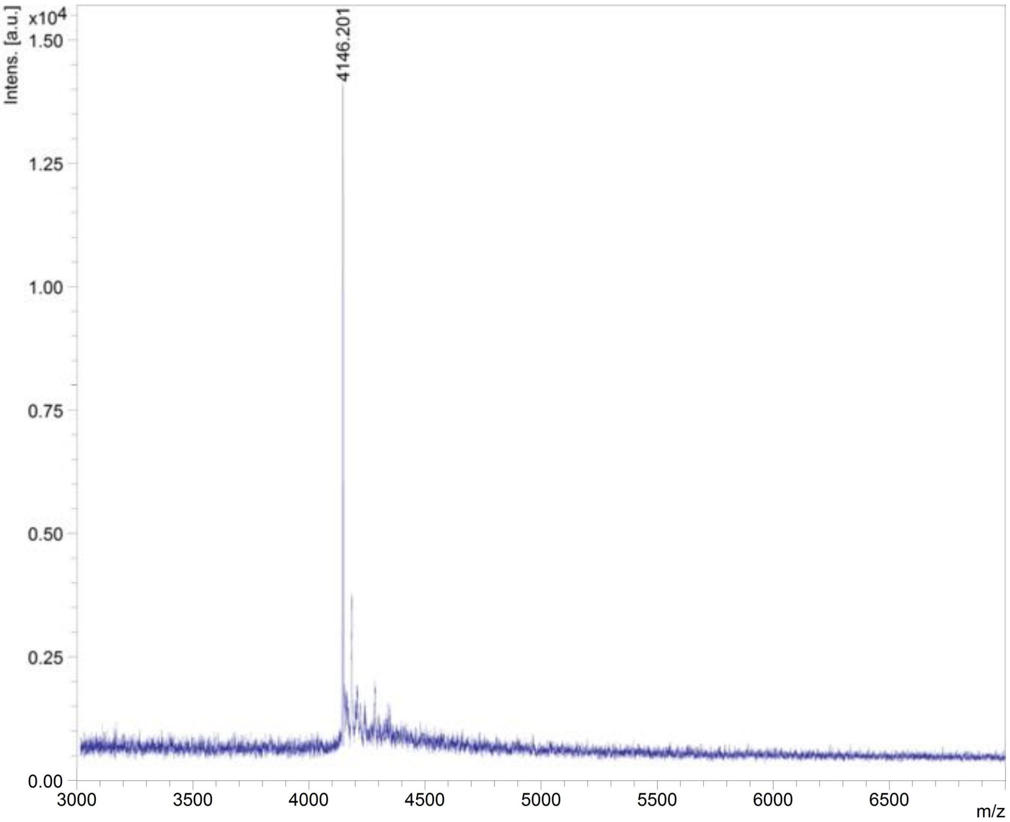

UD-56

[B conc. ACN: 7-40% (30 min)]

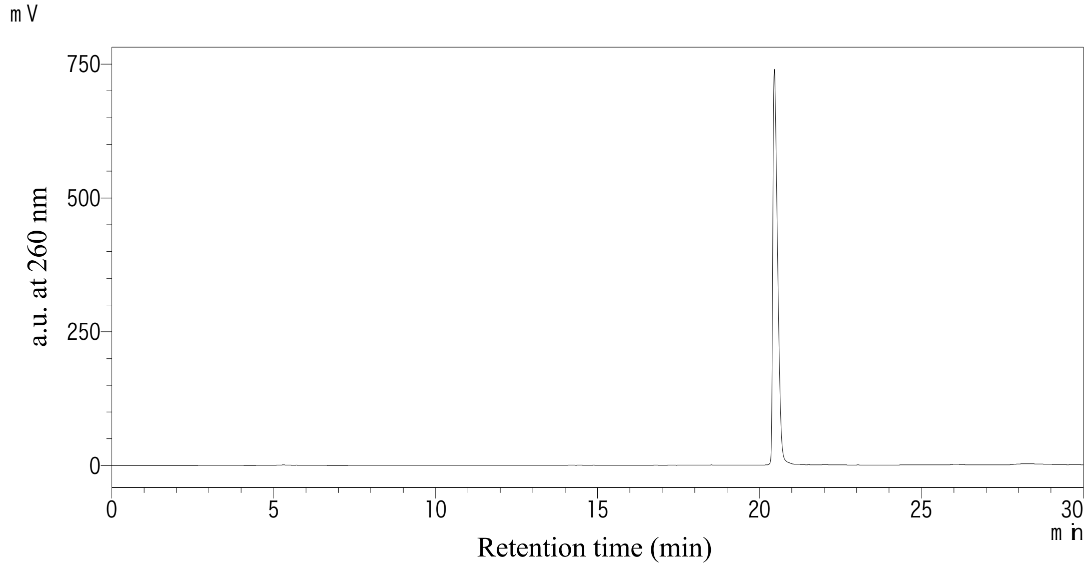

[calcd. (M-H)$^-$: 4194.1, found: 4194.3]

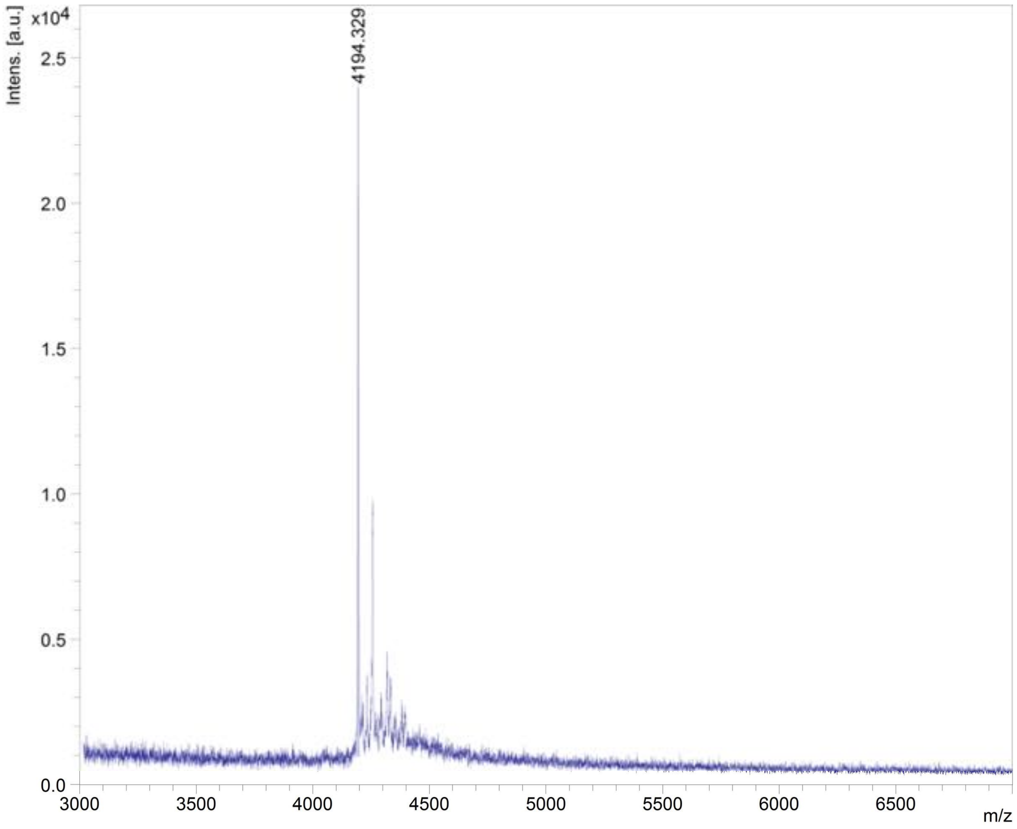

UD-57

[B conc. MeCN: 7-40% (30 min)]

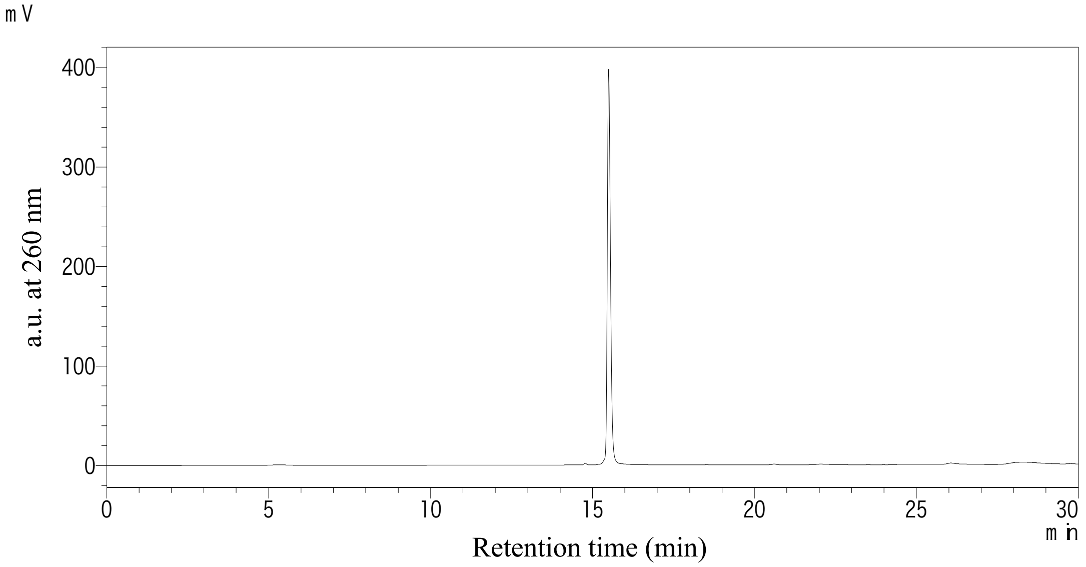

[calcd. (M-H)⁻: 4162.0, found: 4162.5]

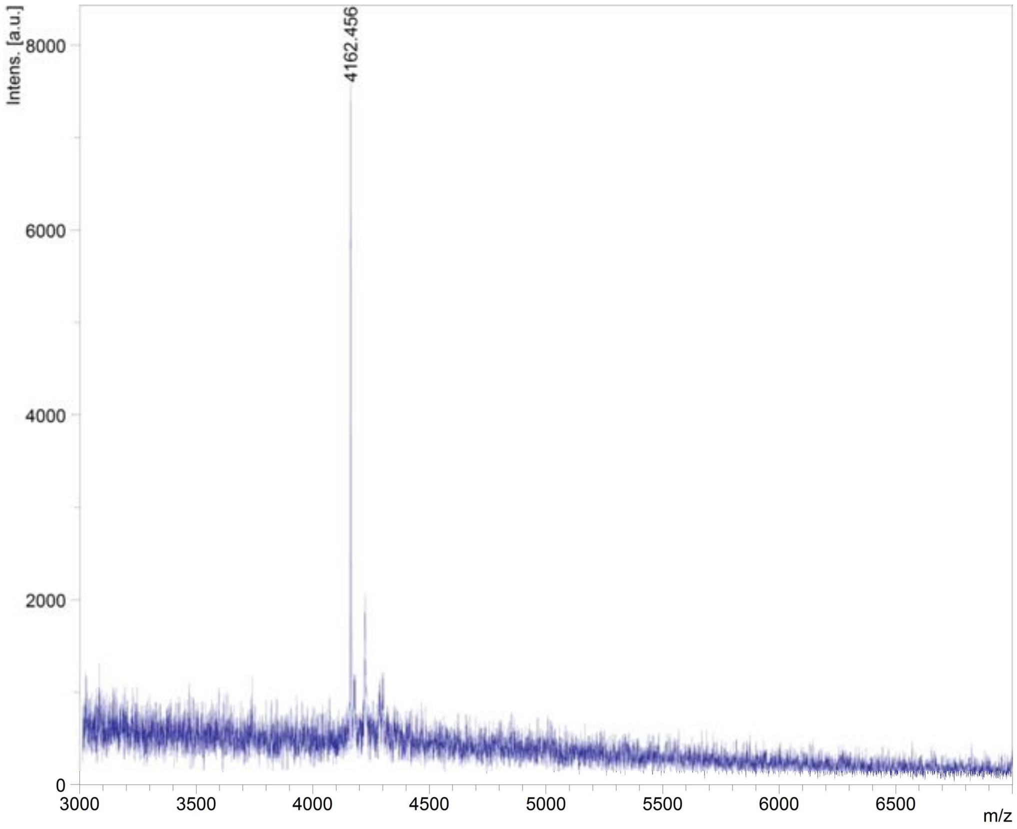

UD-58

[B conc. MeCN: 7-40% (30 min)]

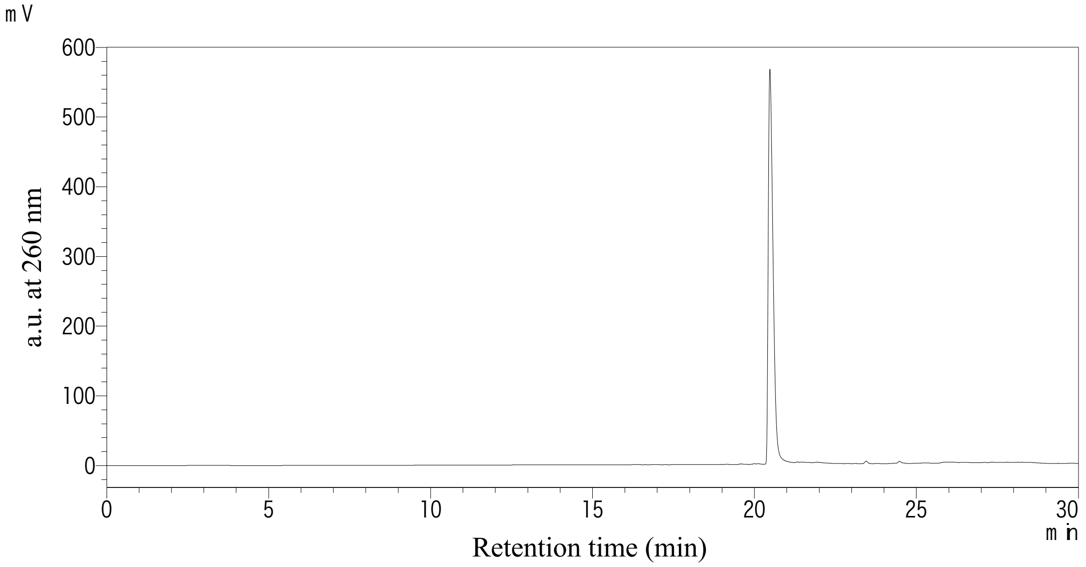

[calcd. (M-H)⁻: 4172.1, found: 4172.3]

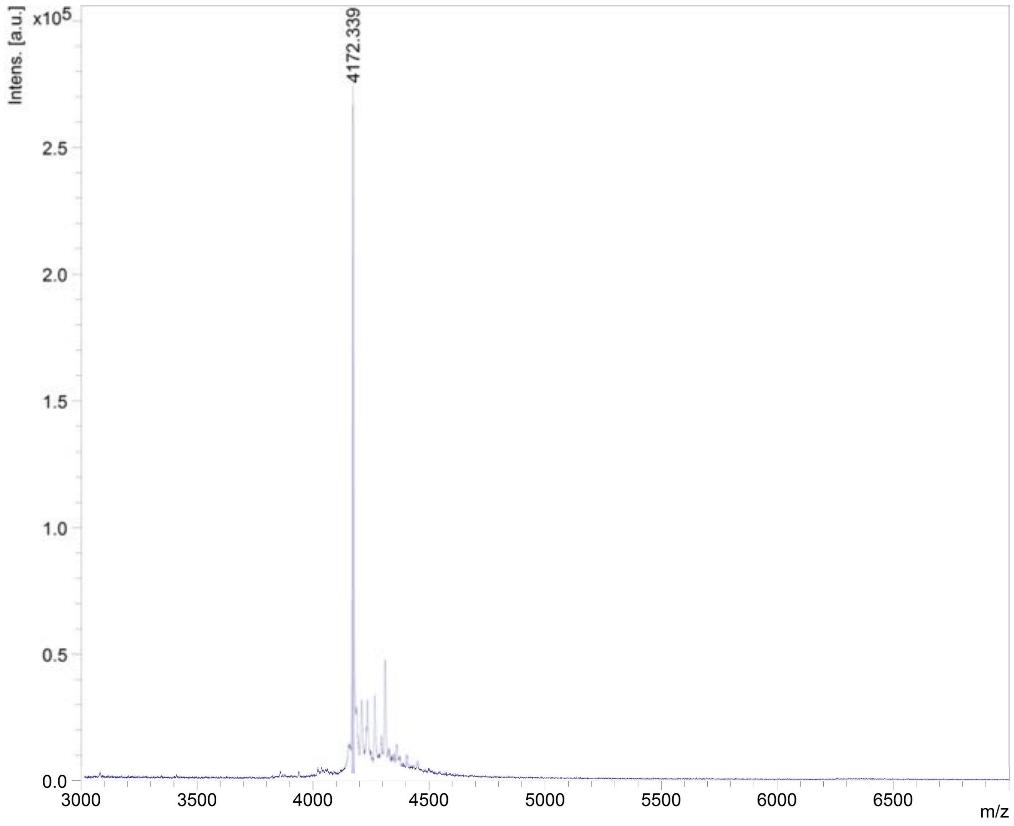

UD-59

[B conc. MeCN: 7-40% (30 min)]

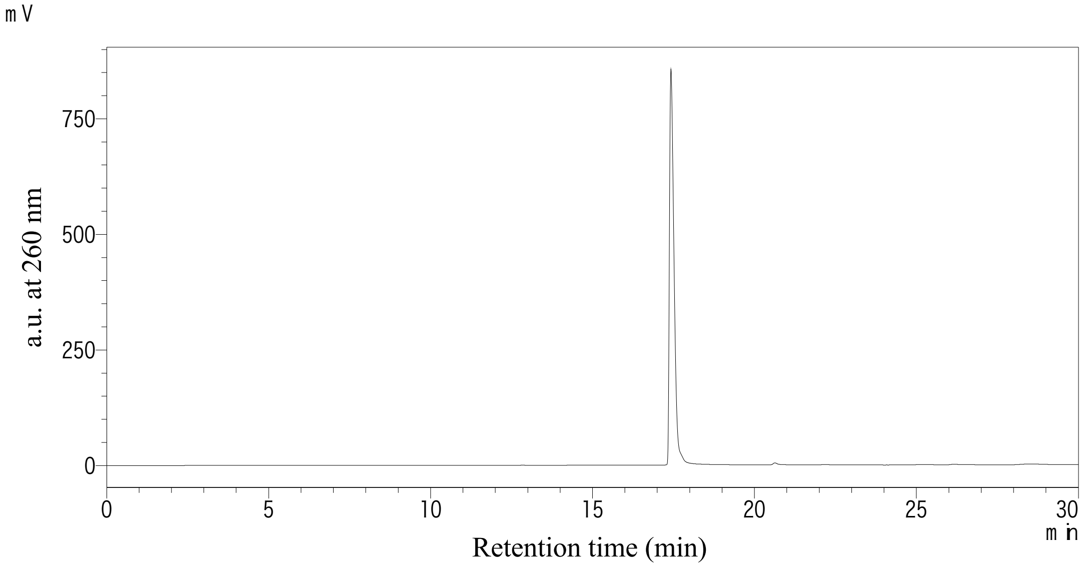

[calcd. (M-H)$^-$: 4146.0, found: 4146.2]

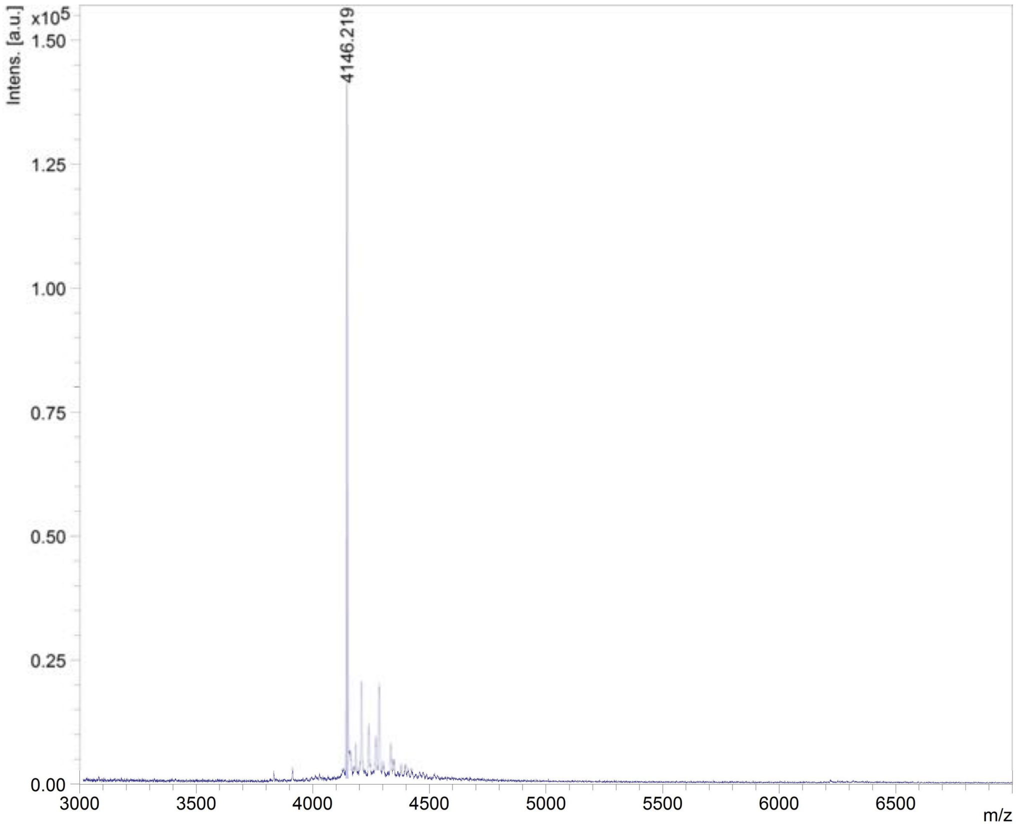

UD-60

[B conc. MeCN: 7-40% (30 min)]

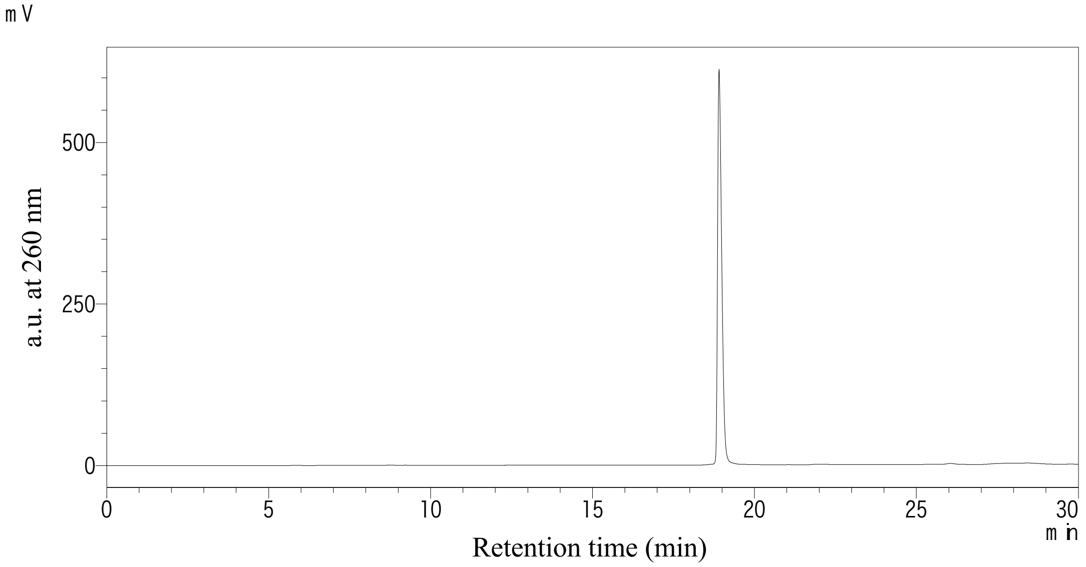

[calcd. (M-H)$^{-}$: 4180.0, found: 4180.6]

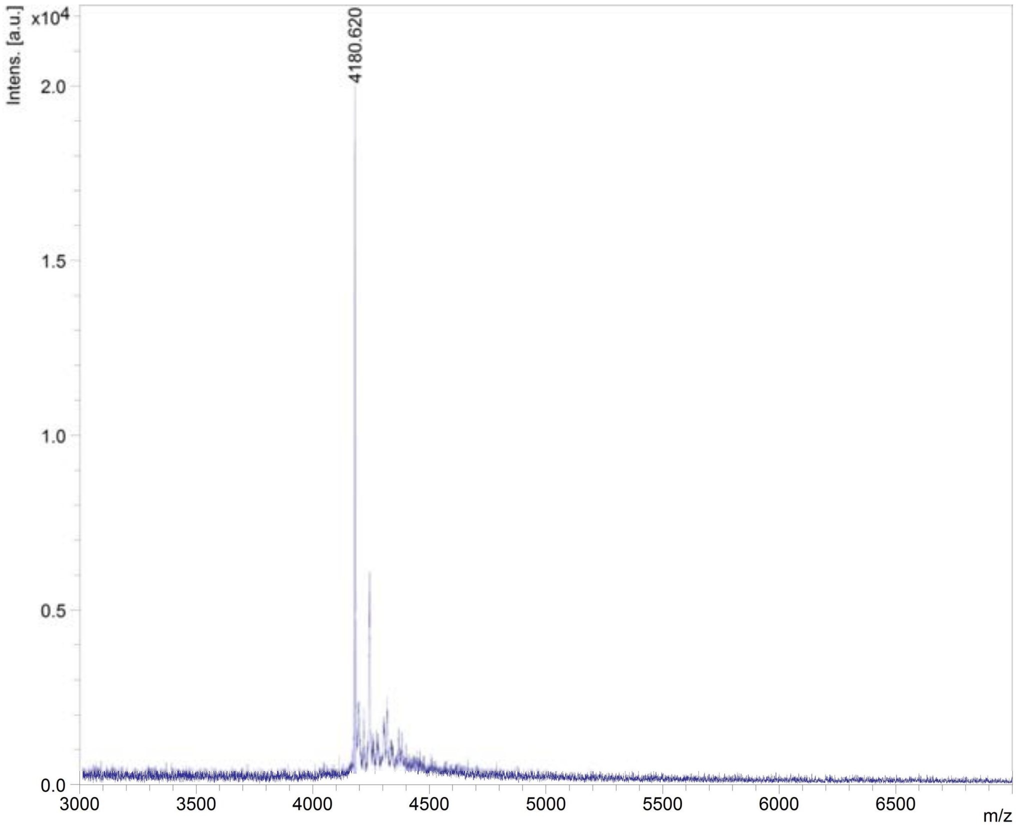

UD-61

[B conc. MeCN: 7-40% (30 min)]

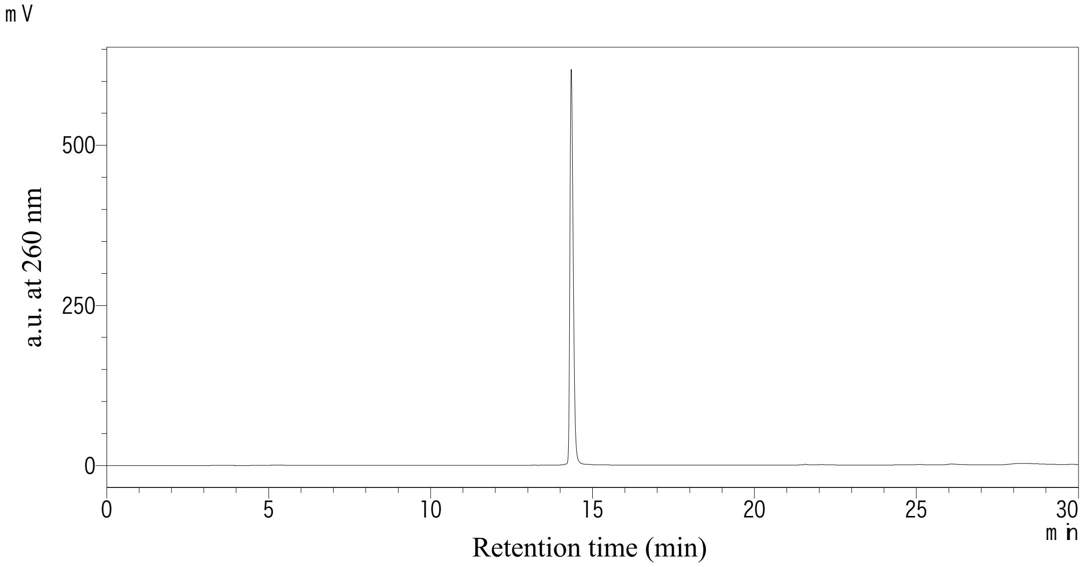

[calcd. (M-H)$^{-}$: 4189.1, found: 4189.0]

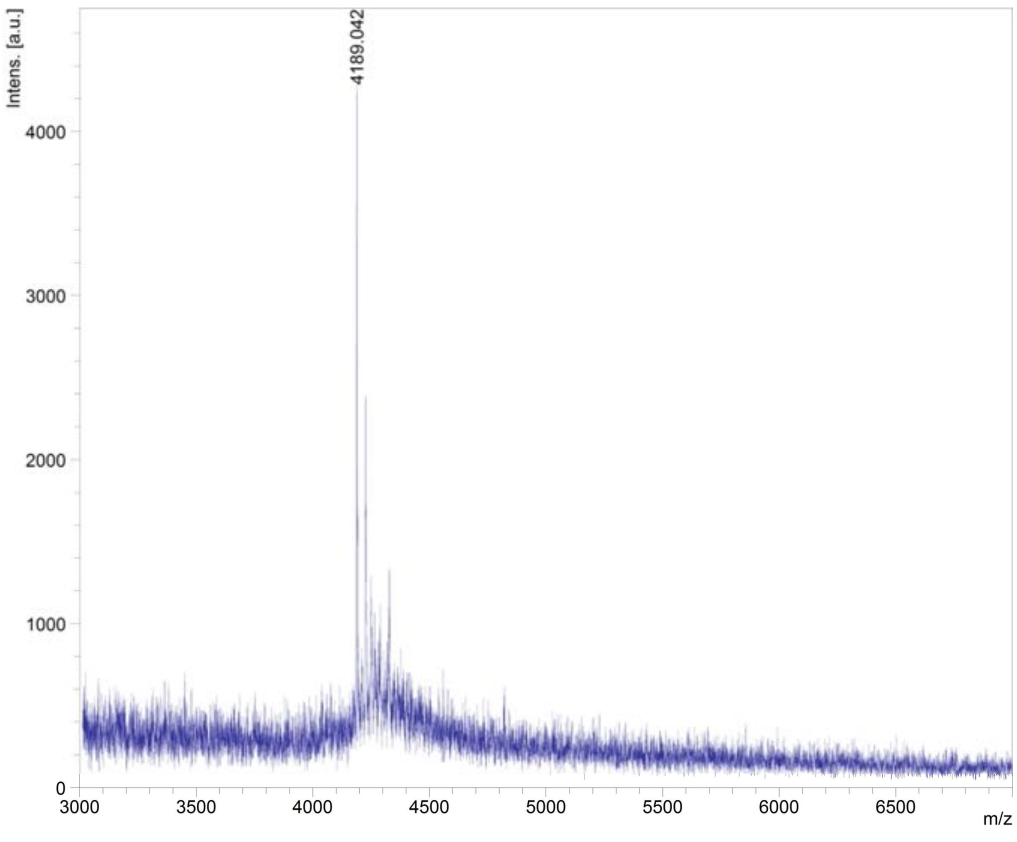

UD-62

[B conc. MeCN: 7-40% (30 min)]

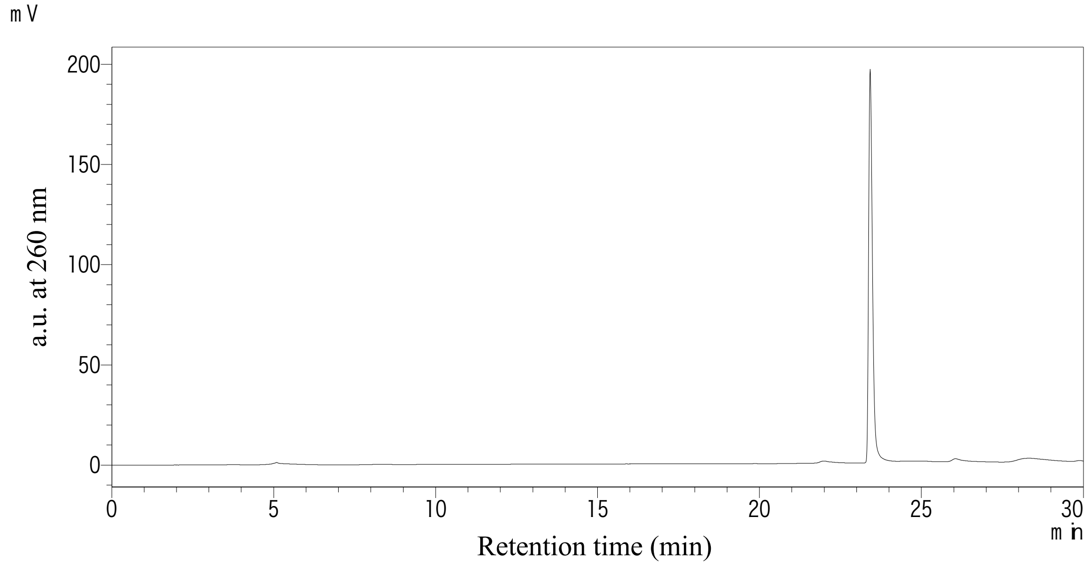

[calcd. (M-H)$^{-}$: 4216.2, found: 4216.2]

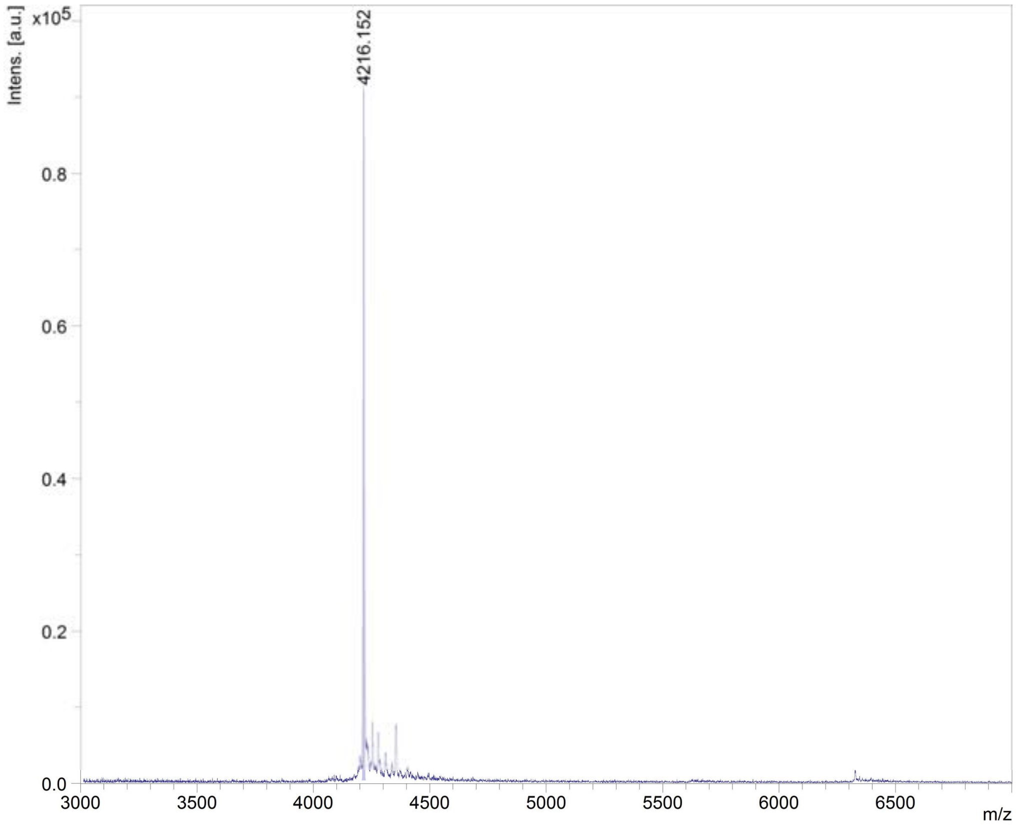

UD-63

[B conc. MeCN: 7-40% (30 min)]

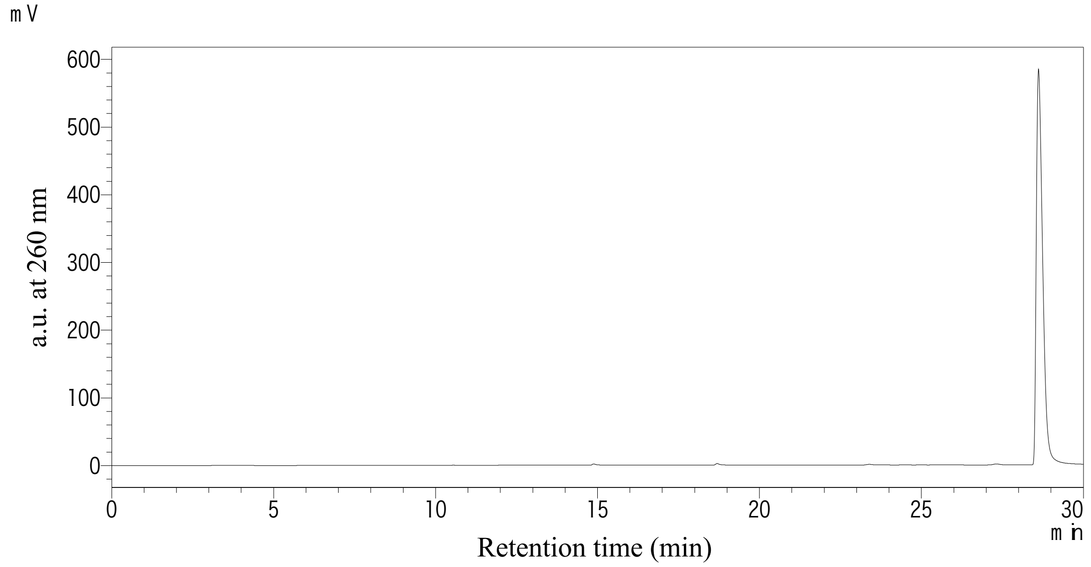

[calcd. (M-H)$^{-}$: 4362.0, found: 4362.7]

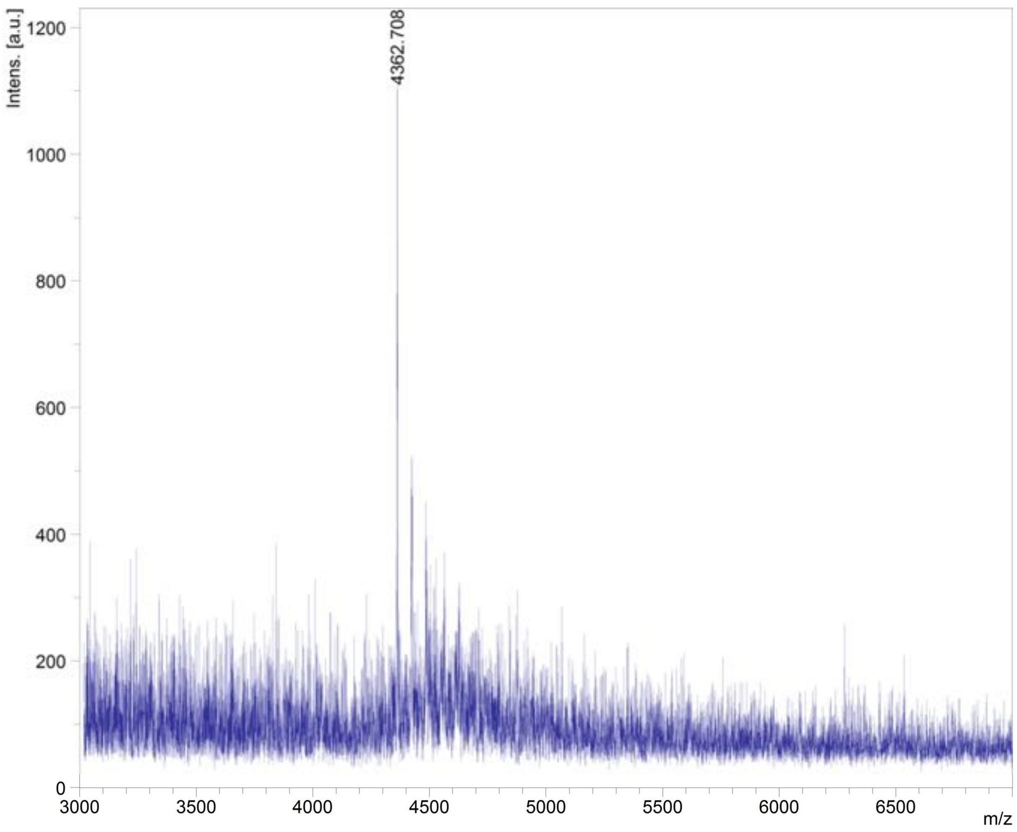

UD-64

[B conc. MeCN: 10-50% (30 min)]

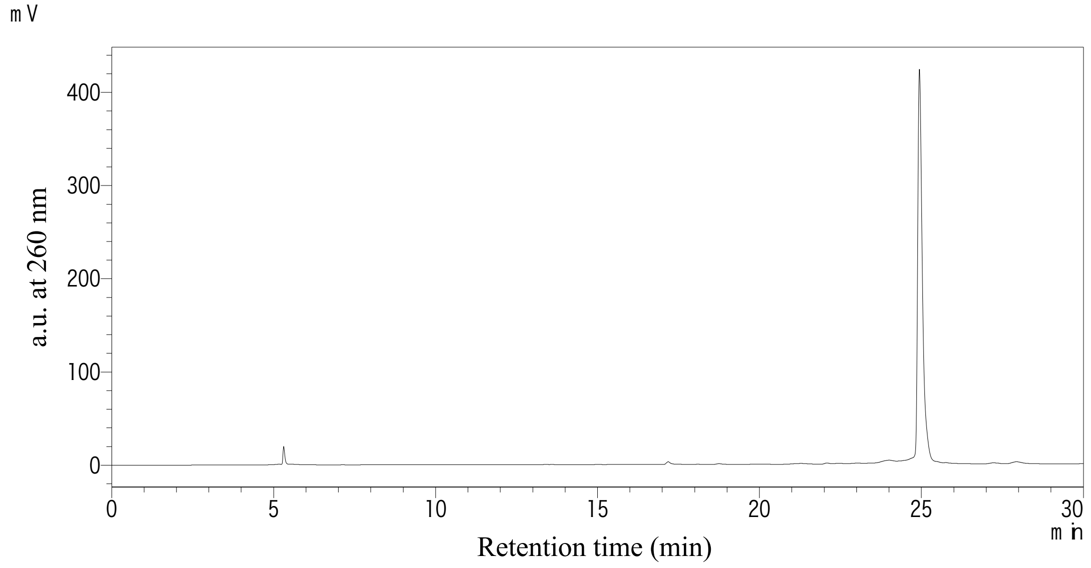

[calcd. (M-H)$^{-}$: 4312.2, found: 4309.9]

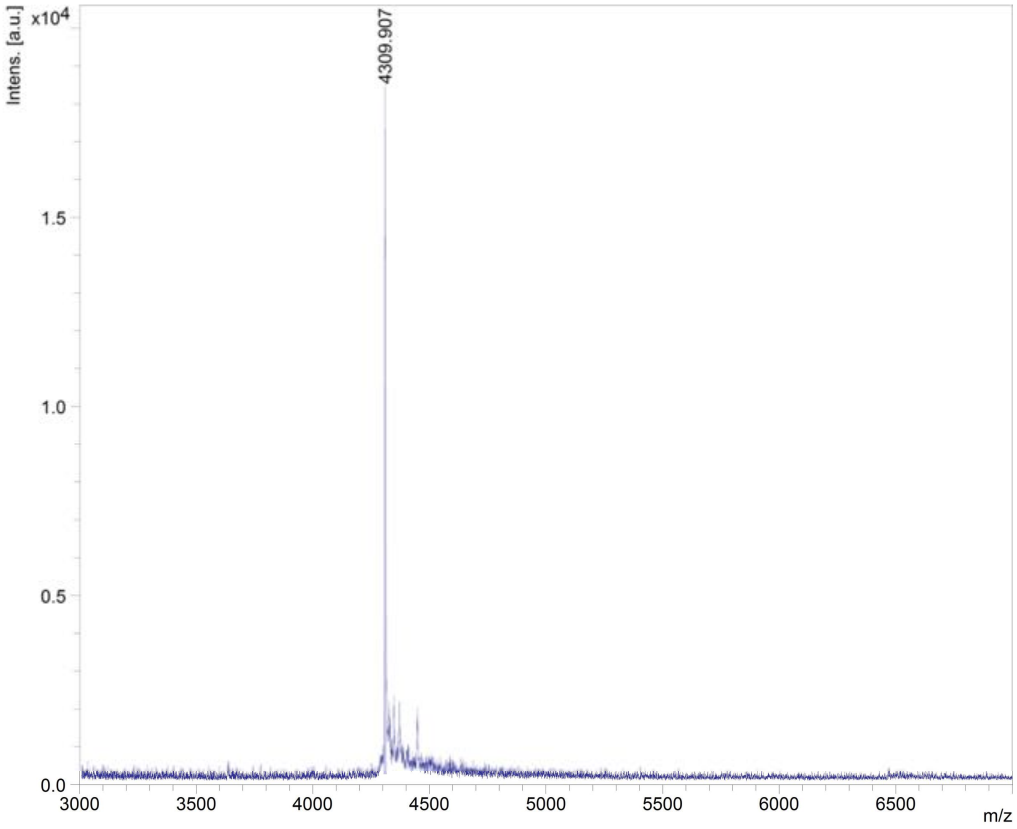

UD-65

[B conc. MeCN: 10-50% (30 min)]

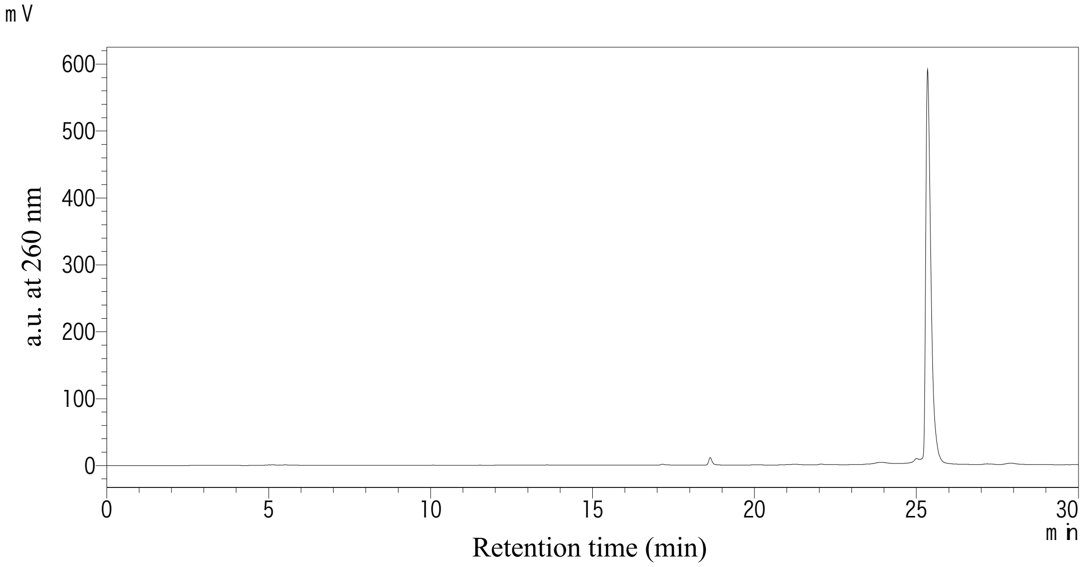

[calcd. (M-H)$^-$: 4330.2, found: 4329.1]

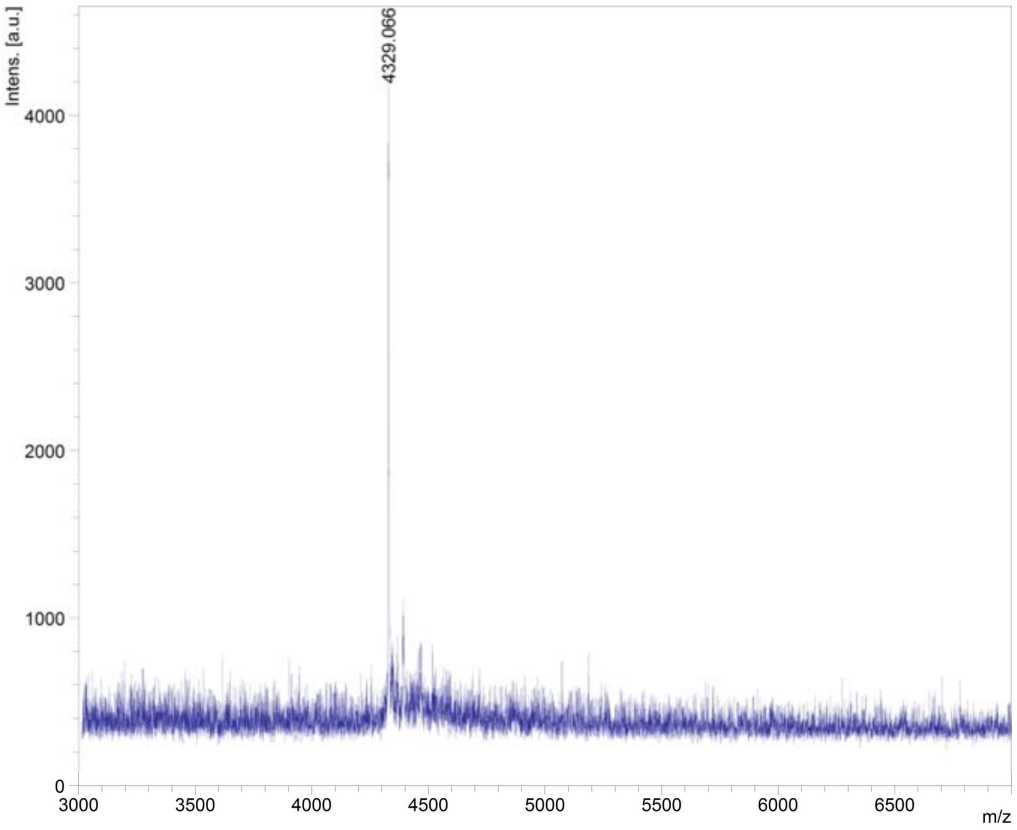

UD-66

[B conc. MeCN: 10-50% (30 min)]

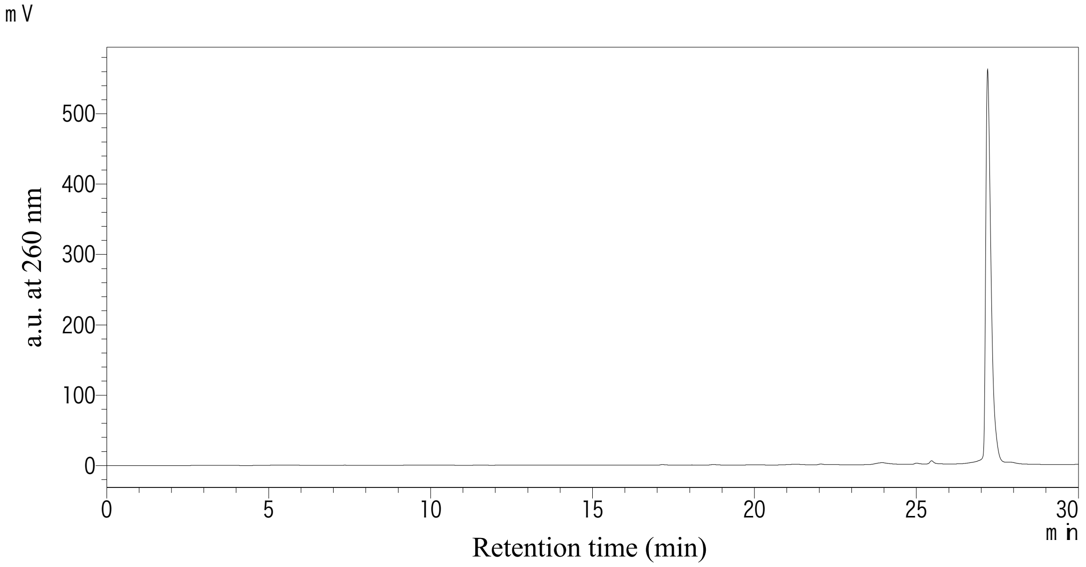

[calcd. $(M-H)^-$: 4346.7, found: 4345.9]

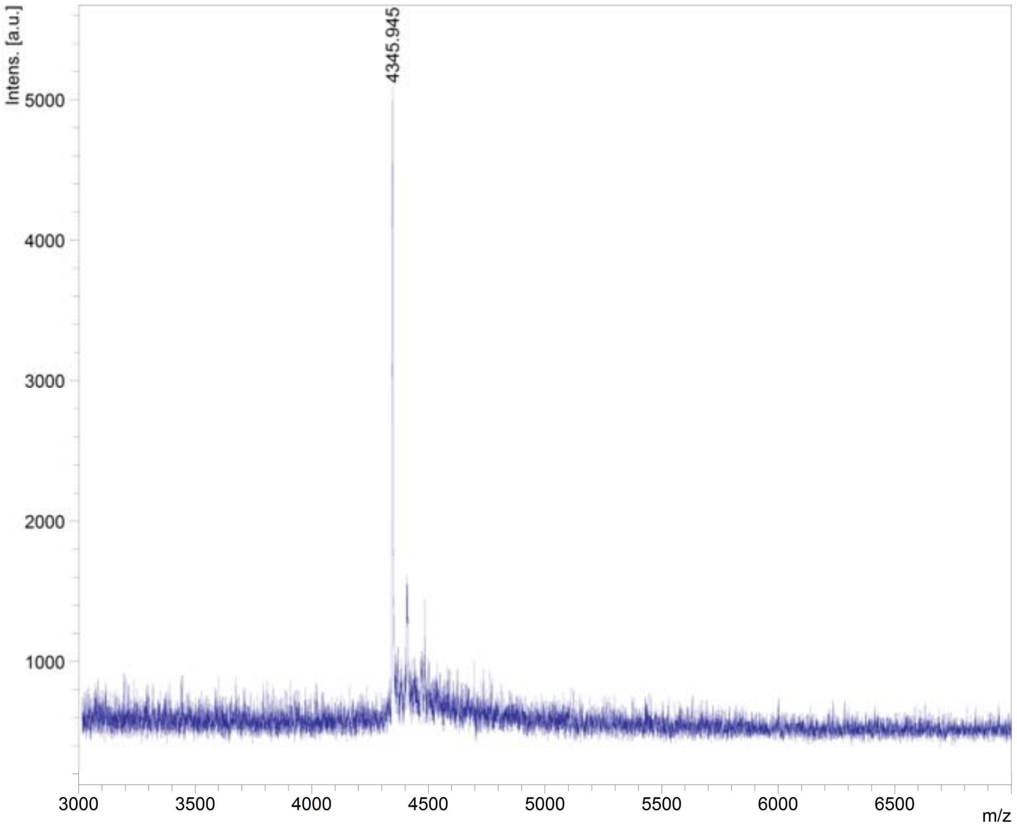

UD-67

[B conc. MeCN: 7-40% (30 min)]

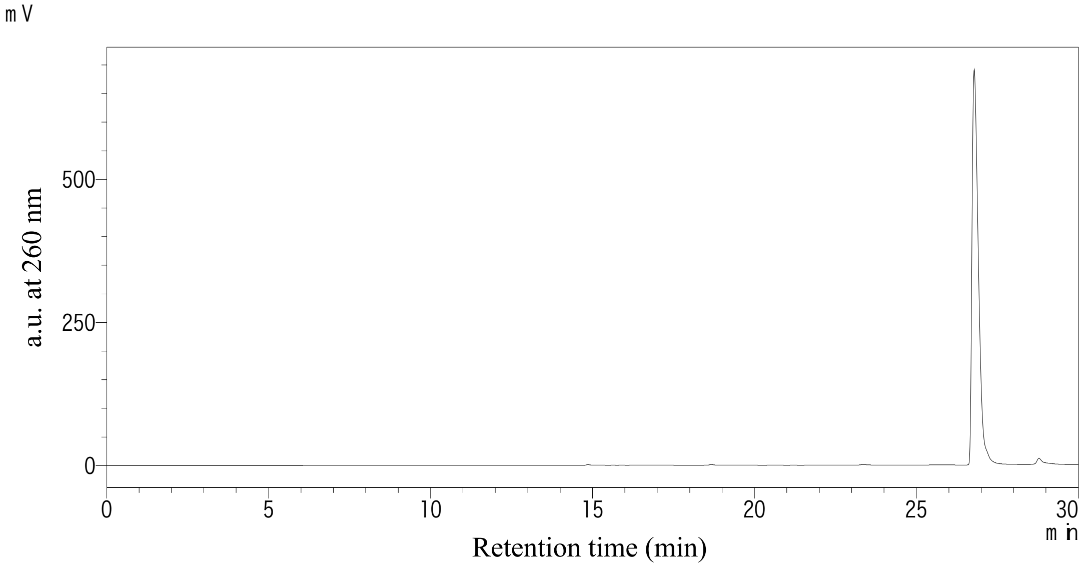

[calcd. (M-H)$^-$: 4350.0, found: 4351.6]

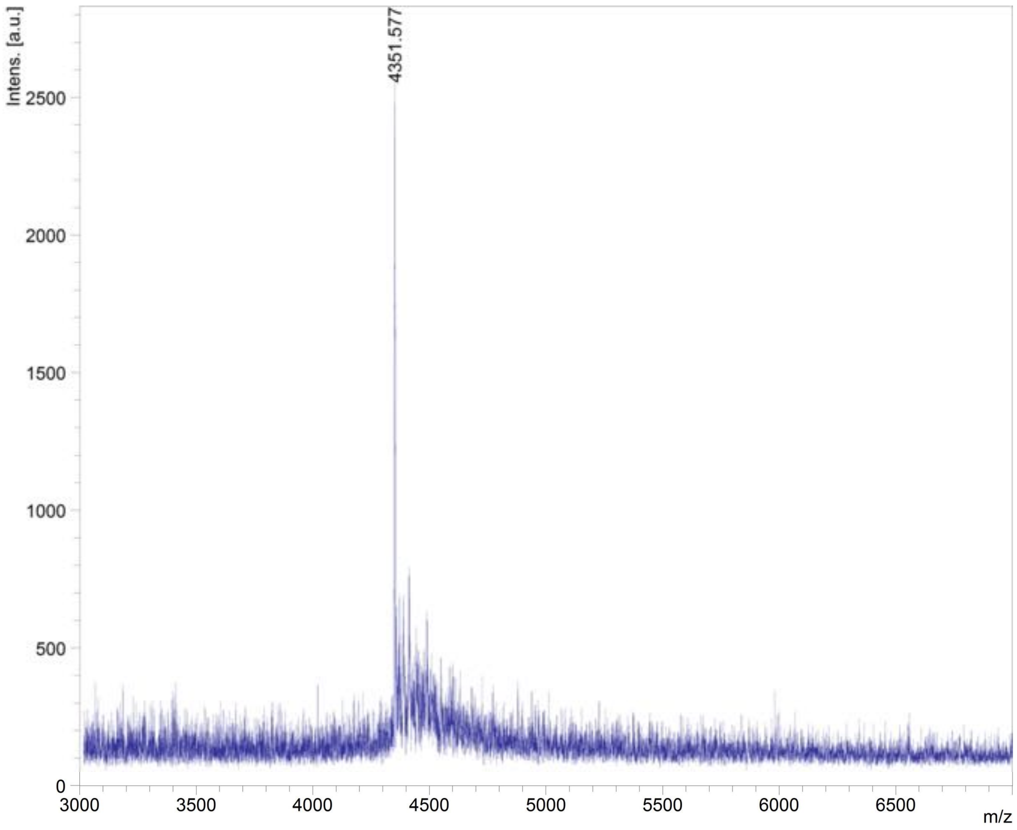

UD-68

[B conc. MeCN: 10-50% (30 min)]

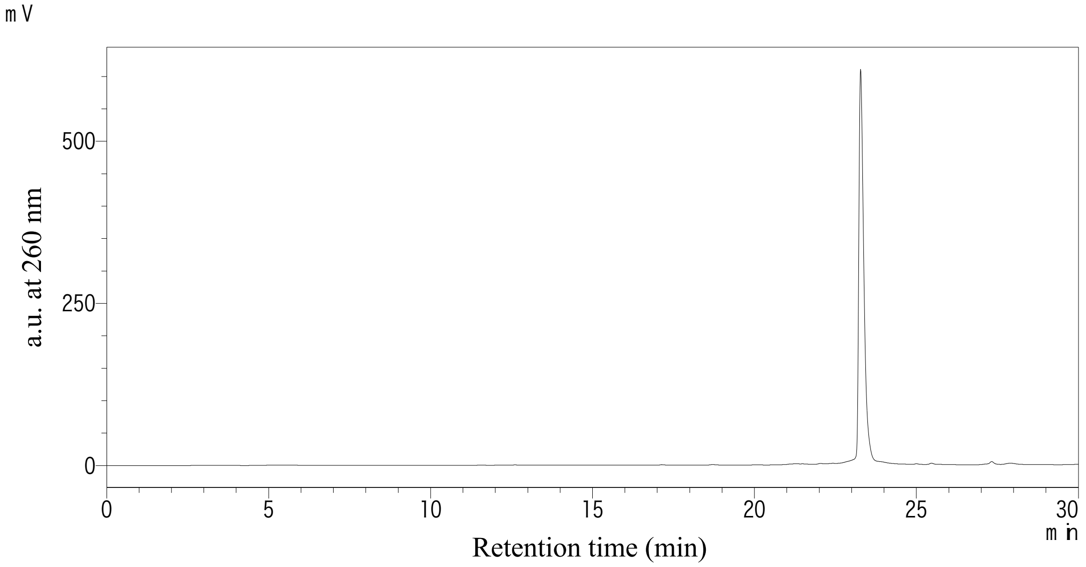

[calcd. (M-H)$^{-}$: 4300.2, found: 4300.0]

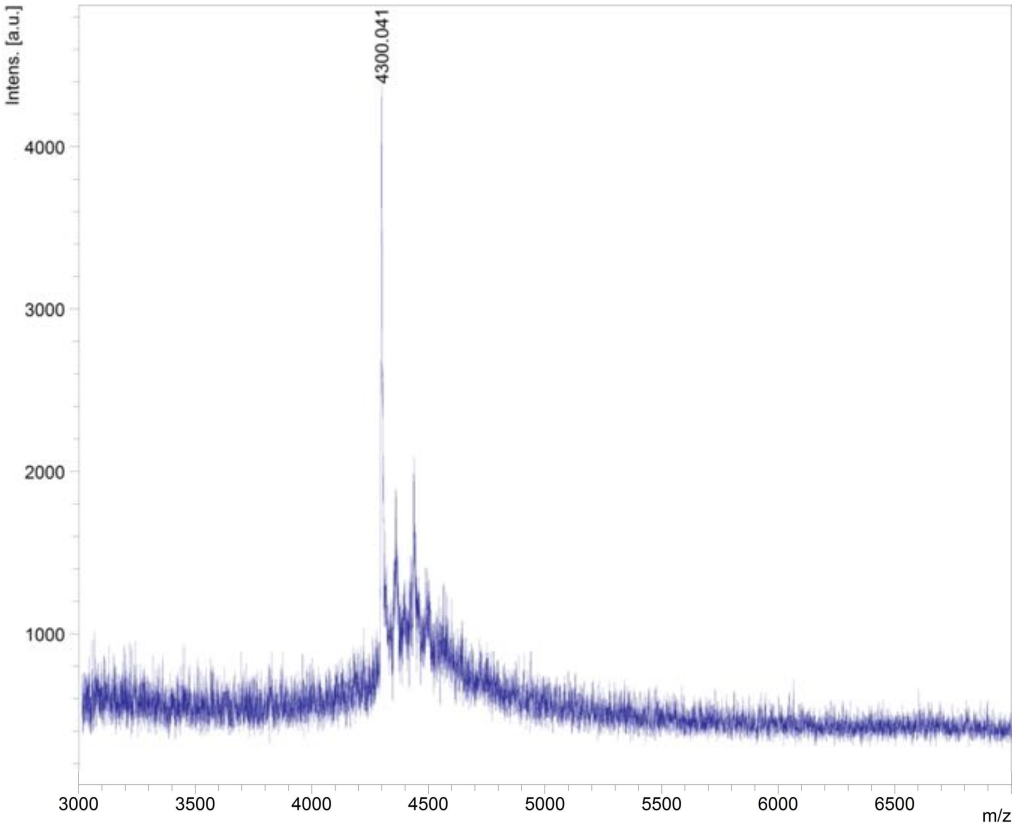

UD-69

[B conc. MeCN: 10-50% (30 min)]

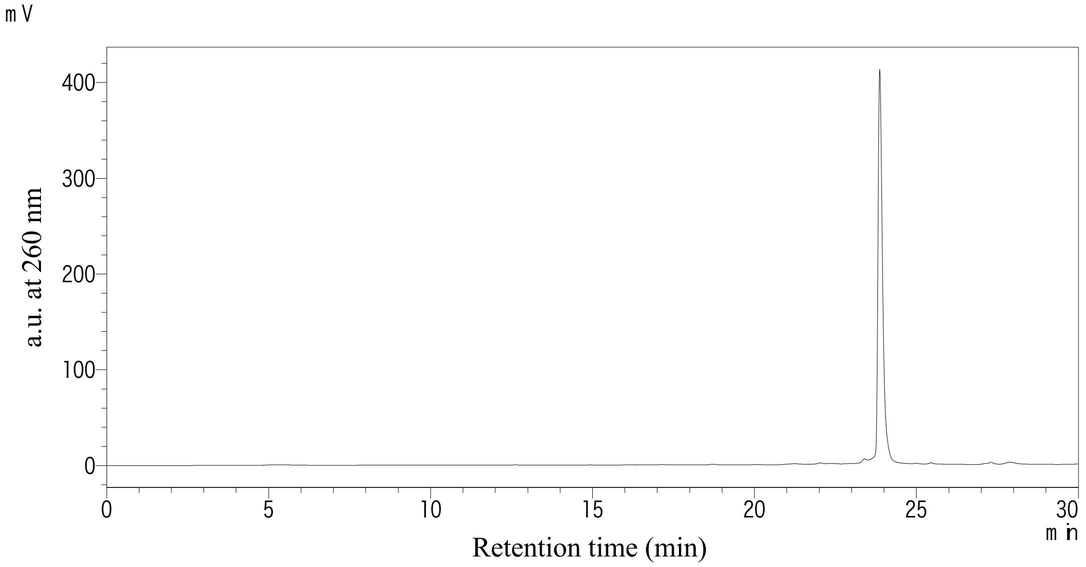

[calcd. (M-H)$^{-}$: 4318.2, found: 4318.6]

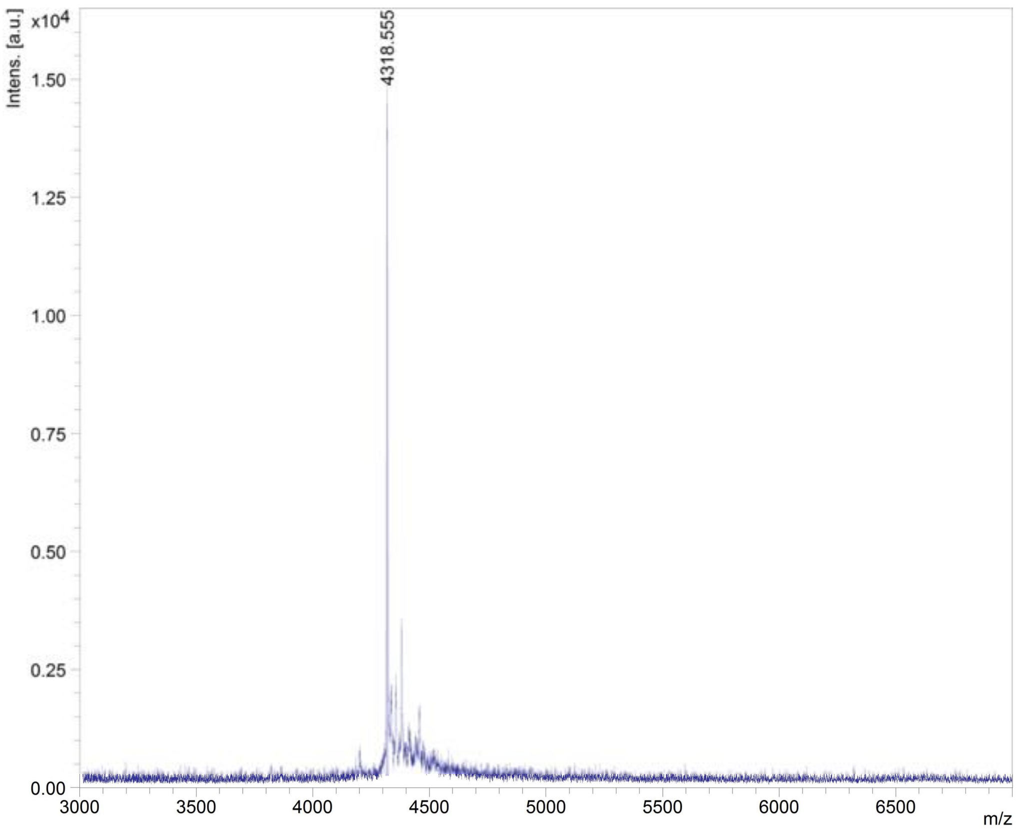

UD-70

[B conc. MeCN: 10-50% (30 min)]

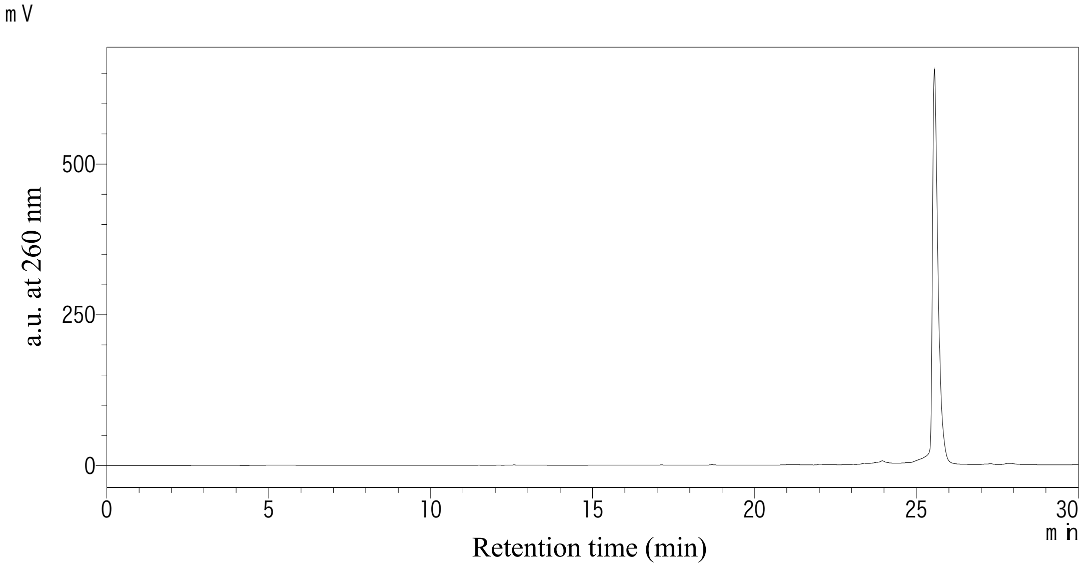

[calcd. (M-H)$^-$: 4334.7, found: 4333.9]

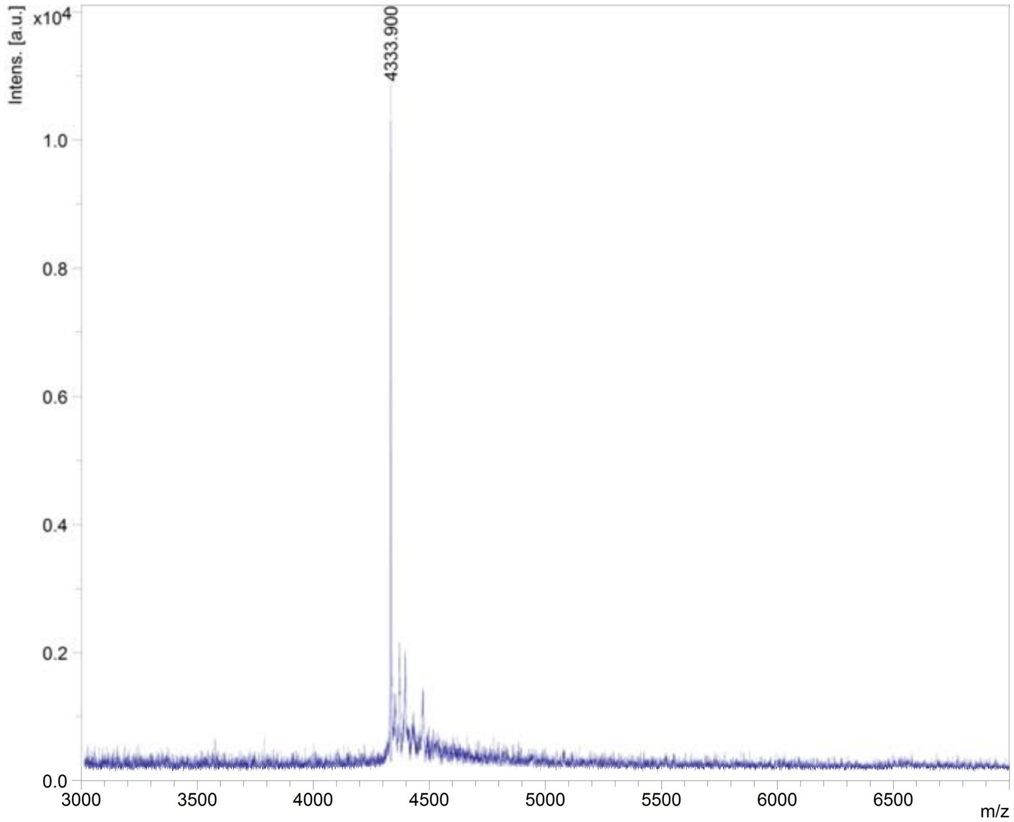

UD-71

[B conc. MeCN: 7-40% (30 min)]

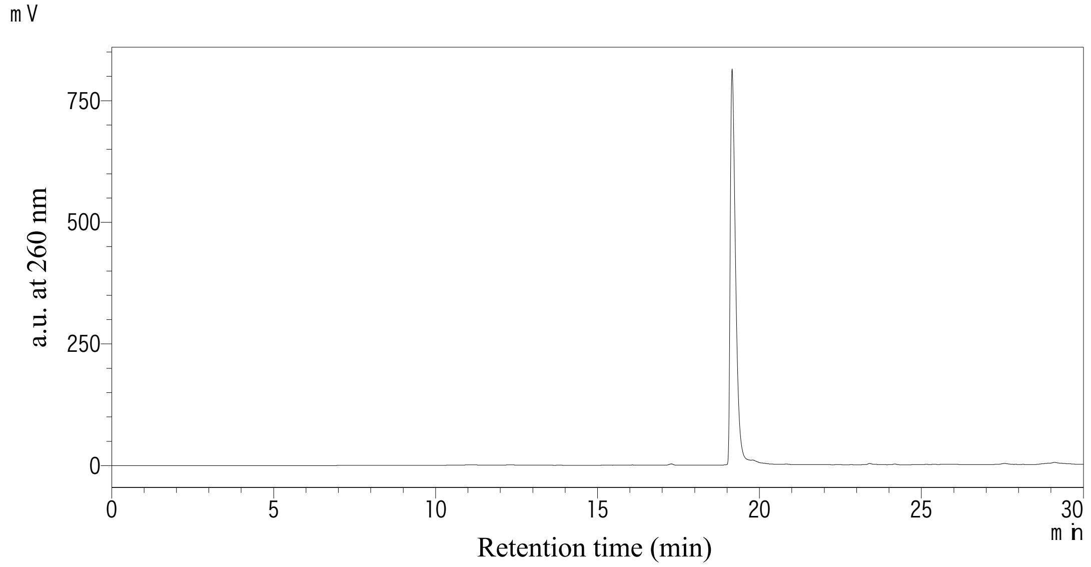

[calcd. (M-H)$^{-}$: 4267.2, found: 4267.5]

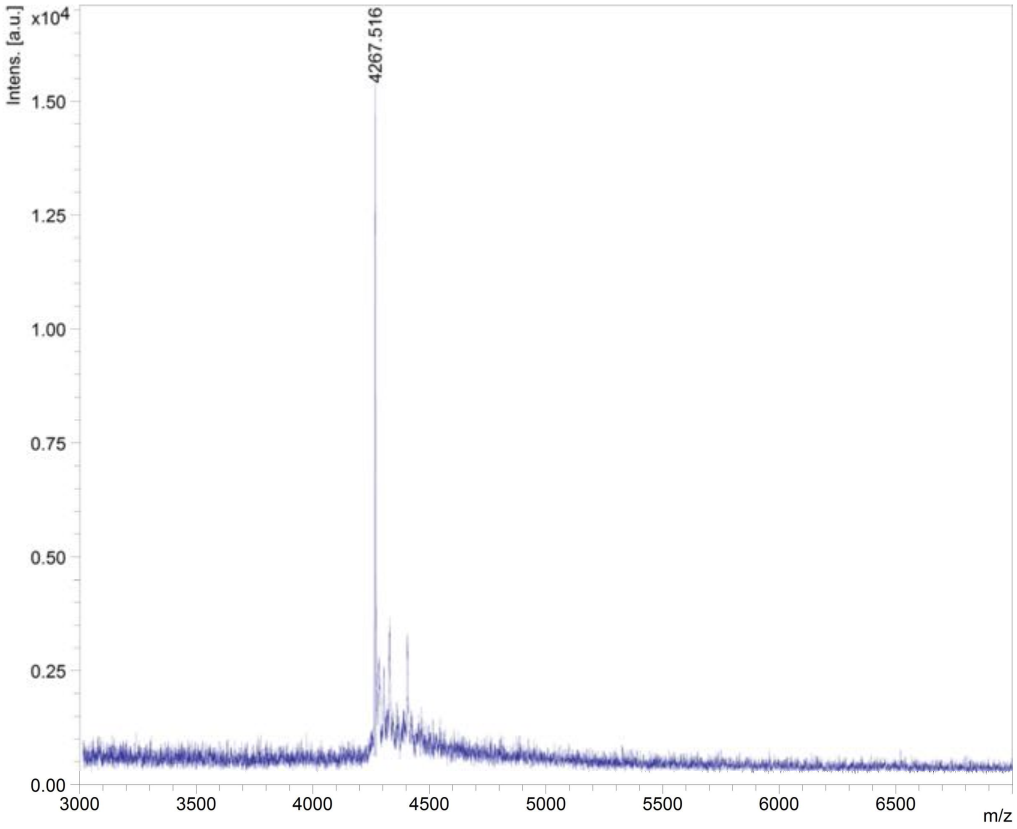

UD-72

[B conc. MeCN: 7-40% (30 min)]

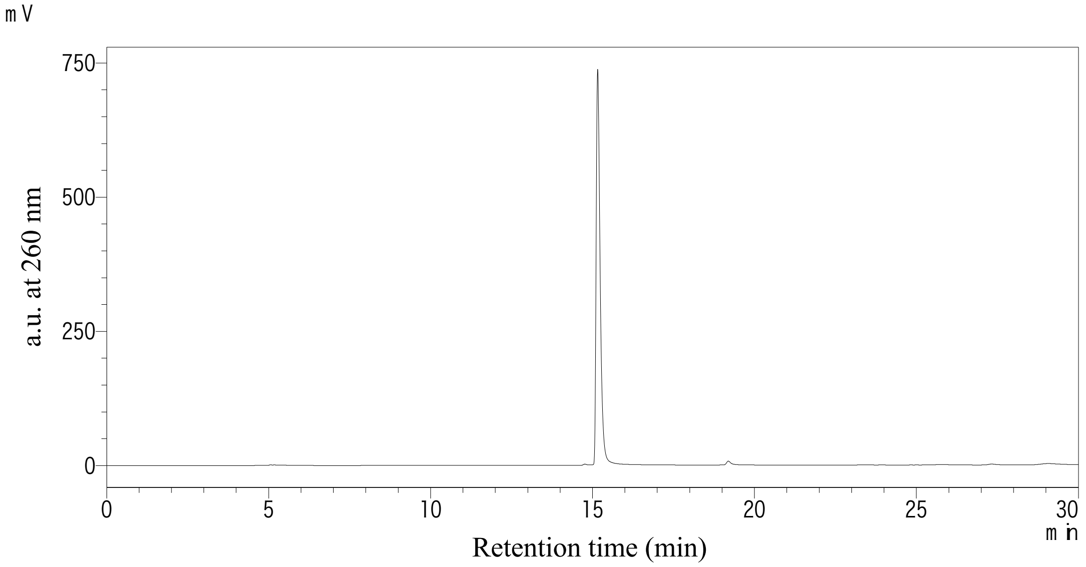

[calcd. (M-H)$^{-}$: 4310.2, found: 4310.3]

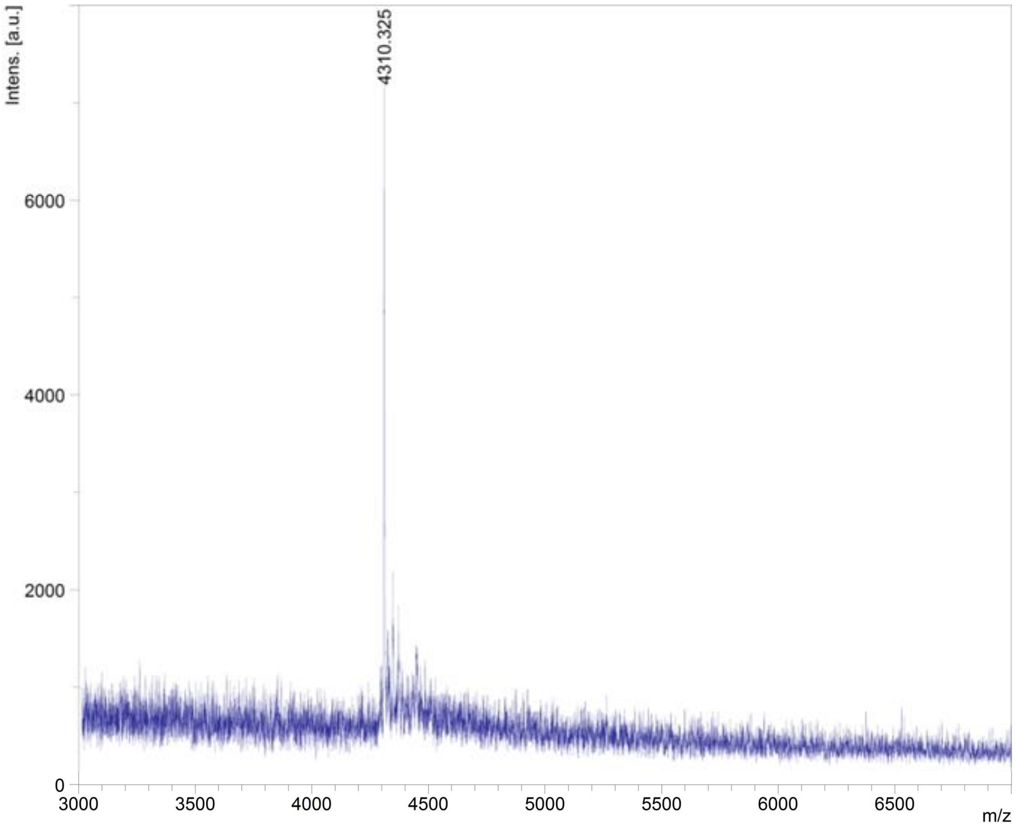

UD-73

[B conc. MeCN: 7-40% (30 min)]

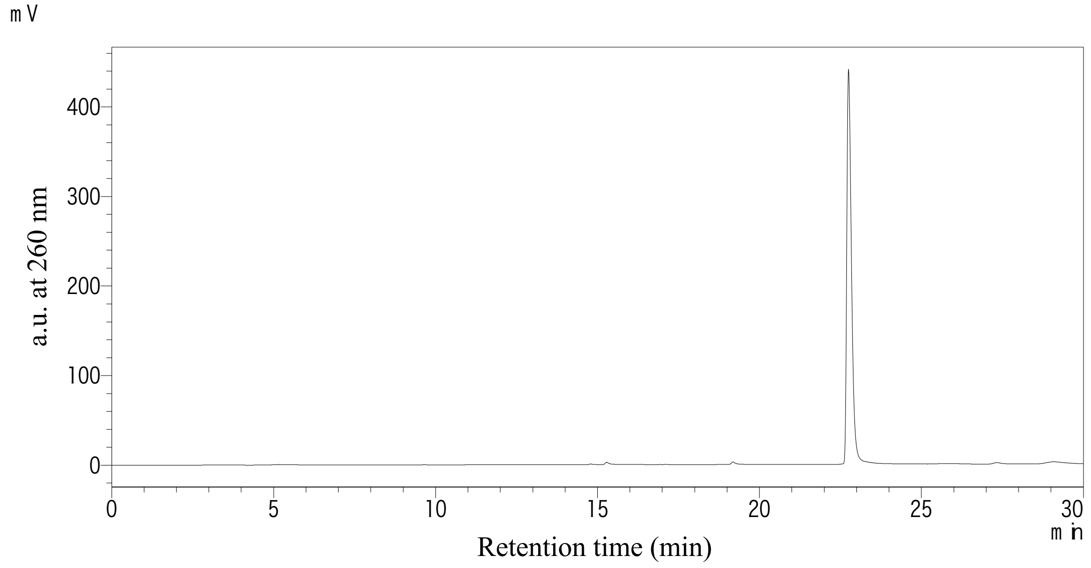

[calcd. (M-H)$^{-}$: 4307.2, found: 4306.4]

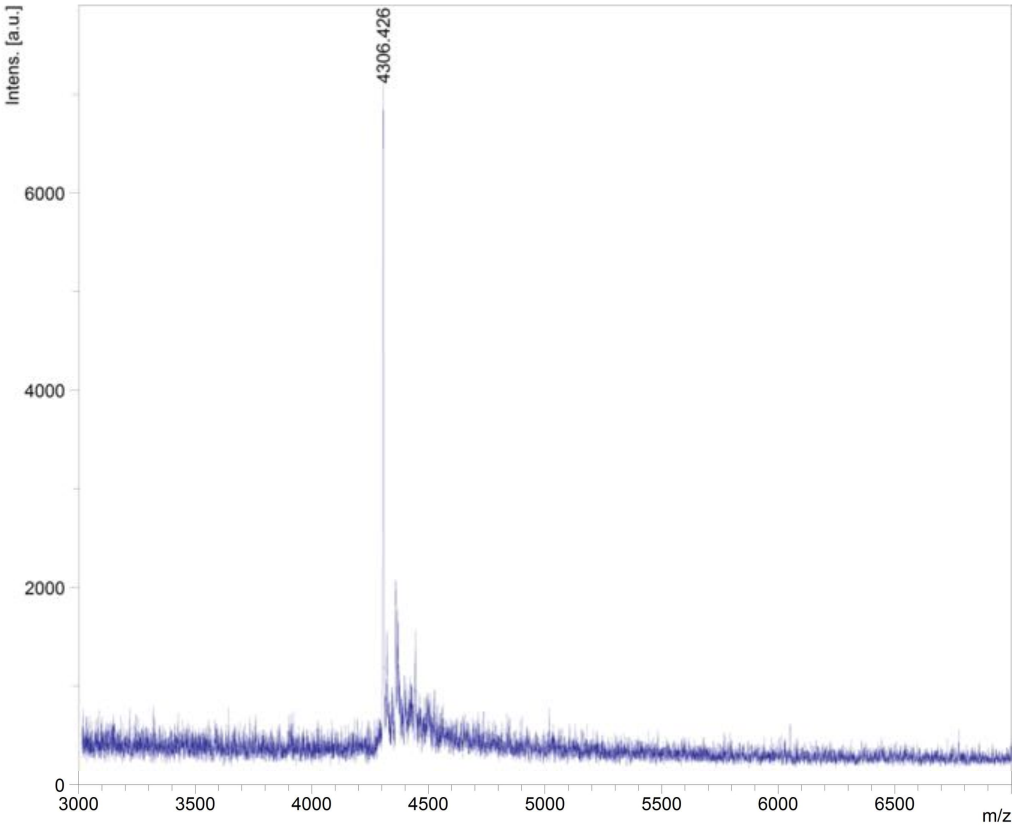

UD-74

[B conc. MeCN: 7-40% (30 min)]

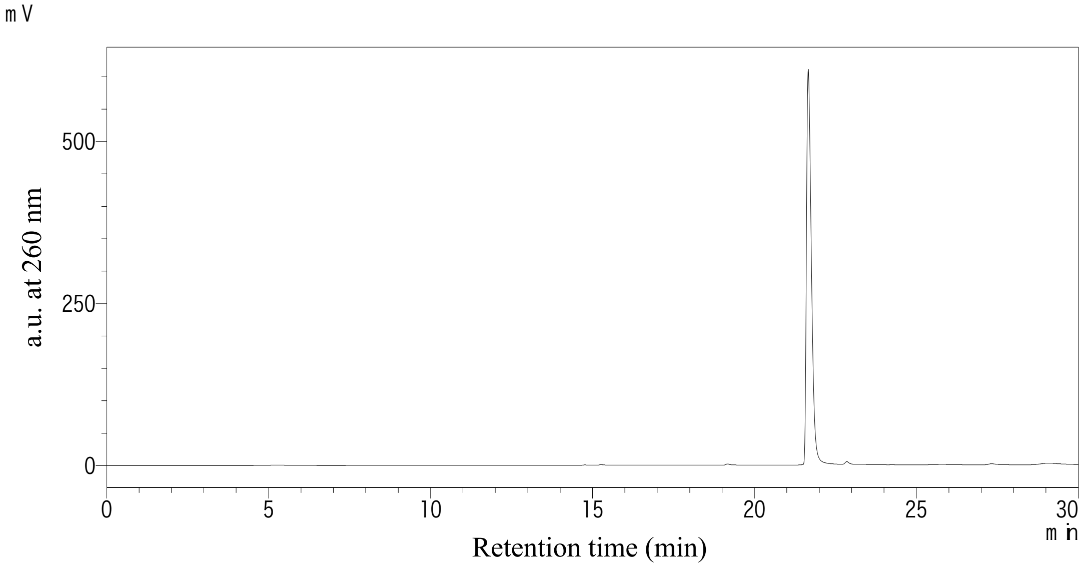

[calcd. (M-H)$^{-}$: 4315.2, found: 4314.6]

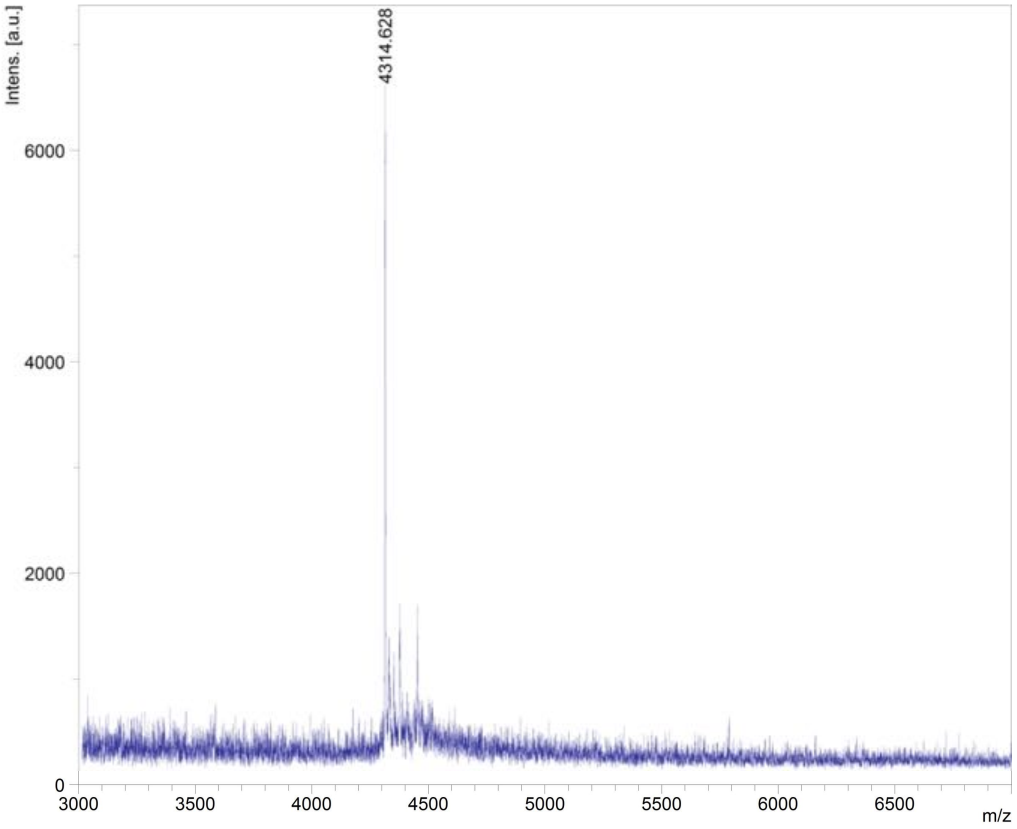

UD-75

[B conc. MeCN: 7-40% (30 min)]

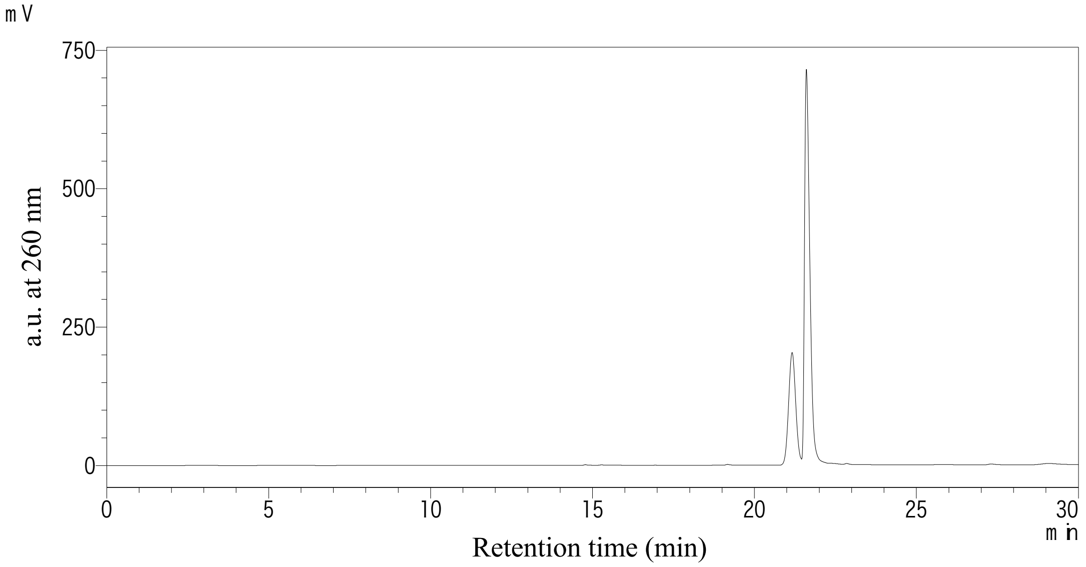

[calcd. (M-H)$^{-}$: 4301.2, found: 4302.7]

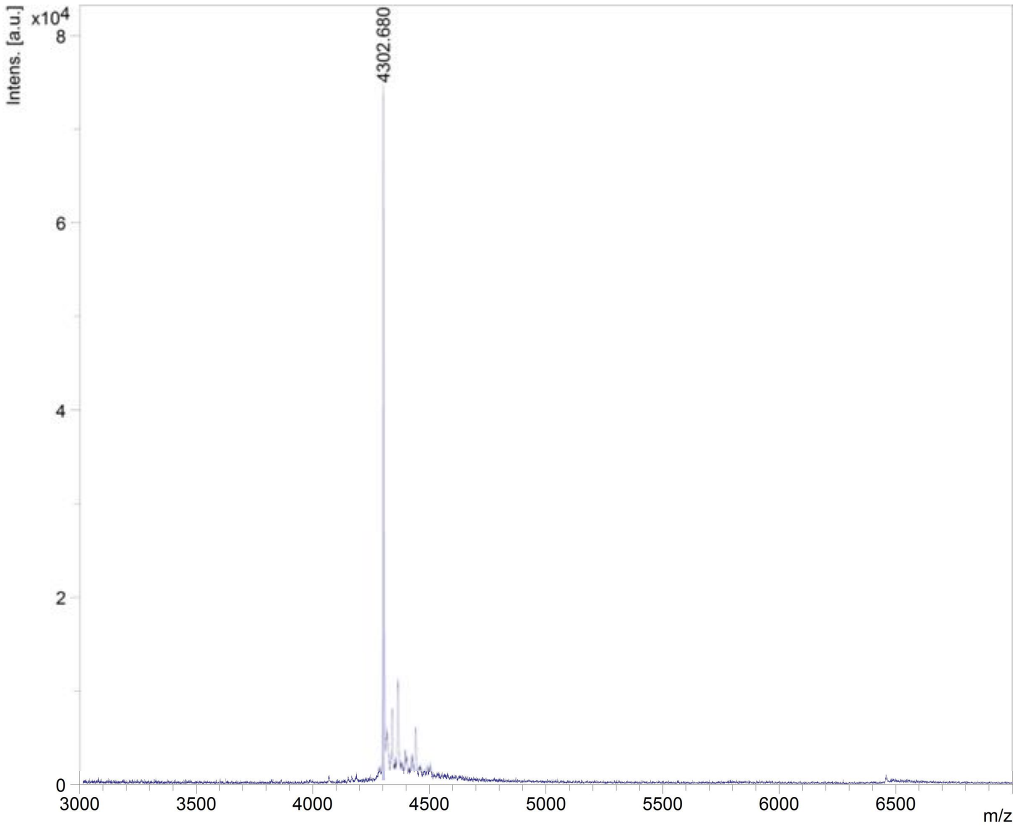

UD-76

[B conc. MeCN: 7-40% (30 min)]

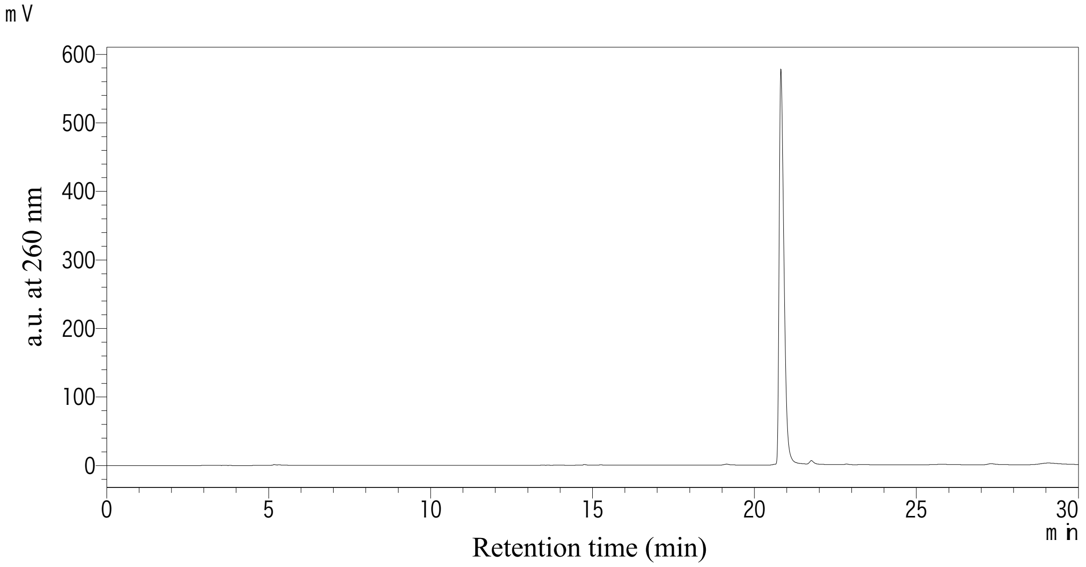

[calcd. (M-H)$^{-}$: 4293.2, found: 4291.9]

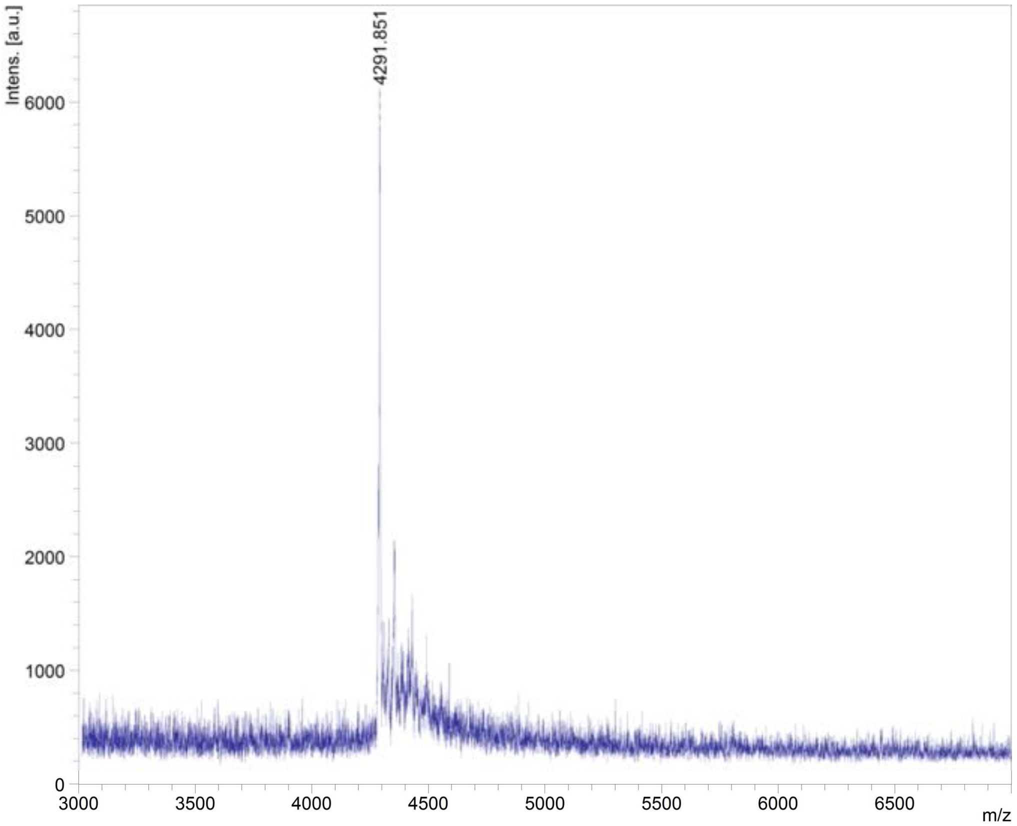

UD-77

[B conc. MeCN: 7-40% (30 min)]

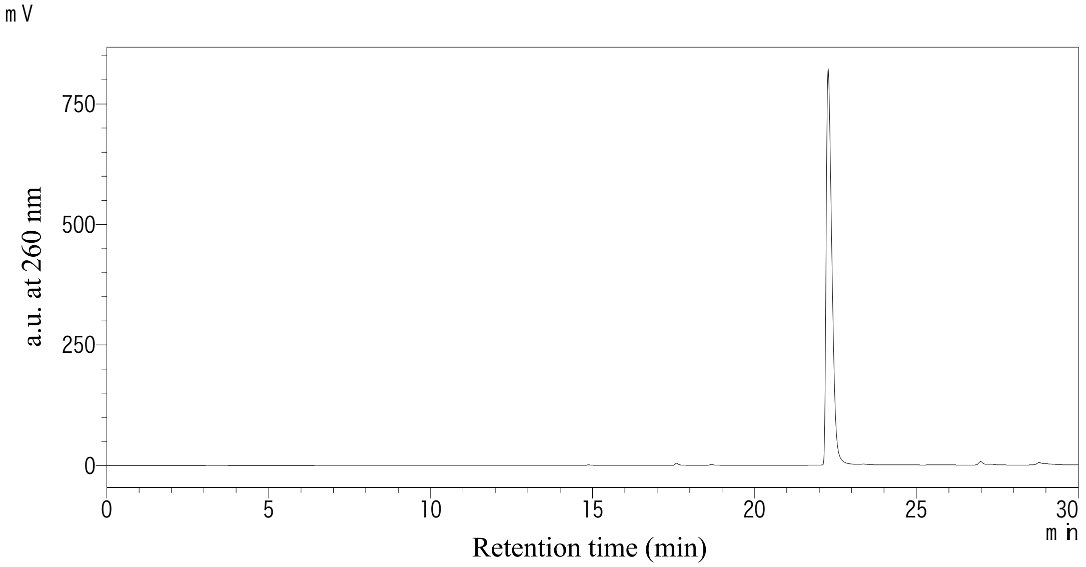

[calcd. (M-H)$^{-}$: 4366.0, found: 4366.3]

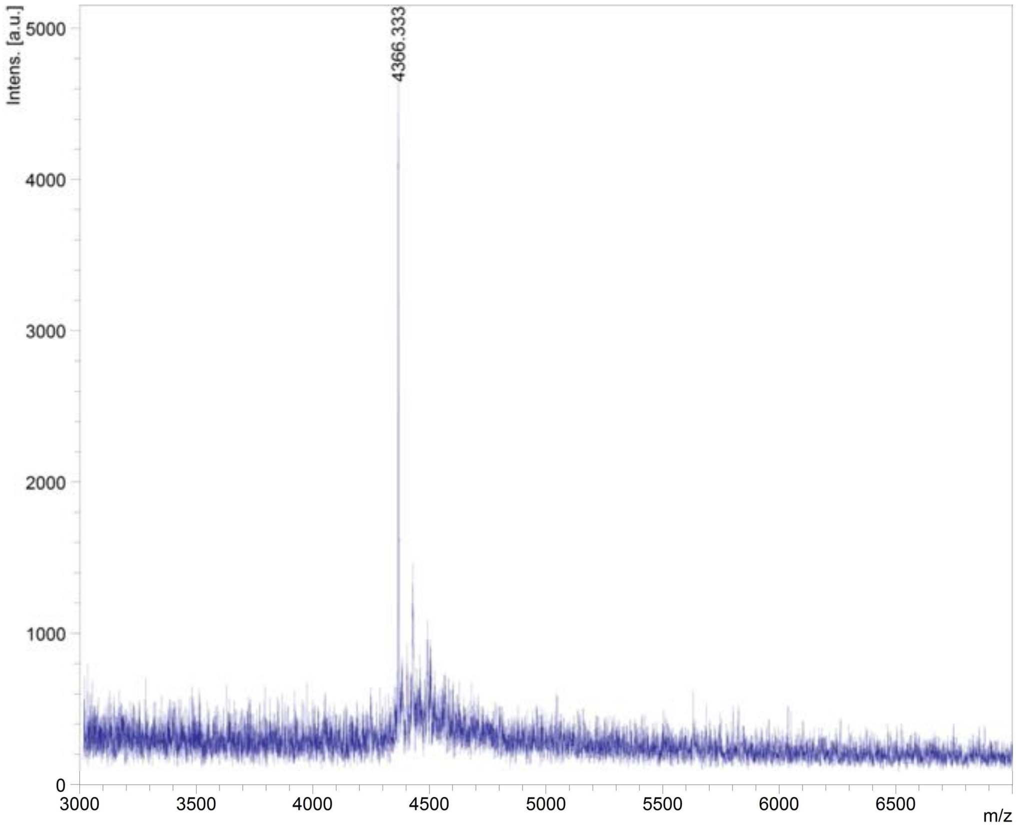

UD-78

[B conc. MeCN: 10-50% (30 min)]

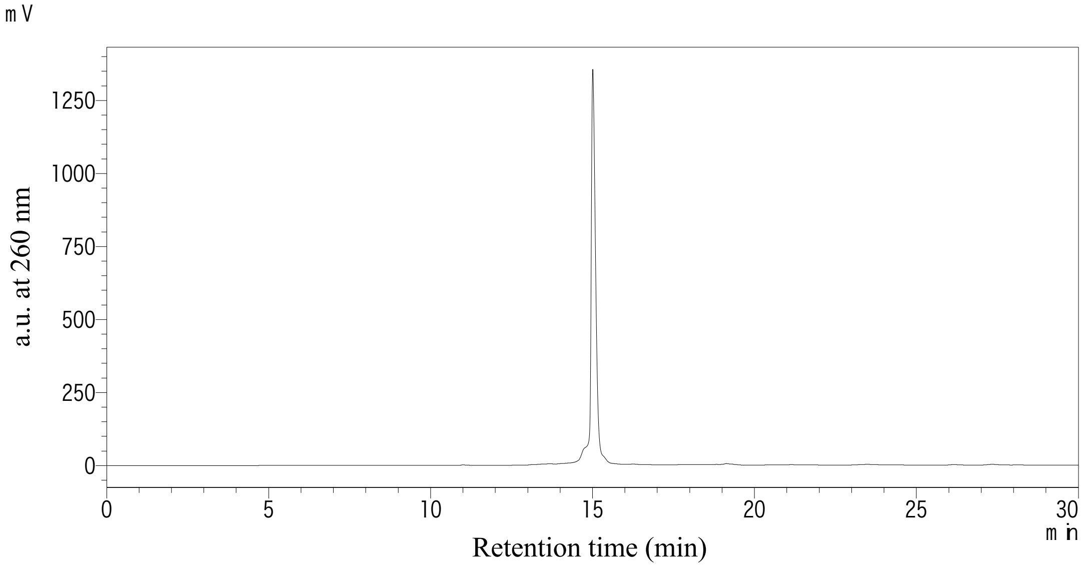

[calcd. (M-H)$^{-}$: 4302.2, found: 4305.6]

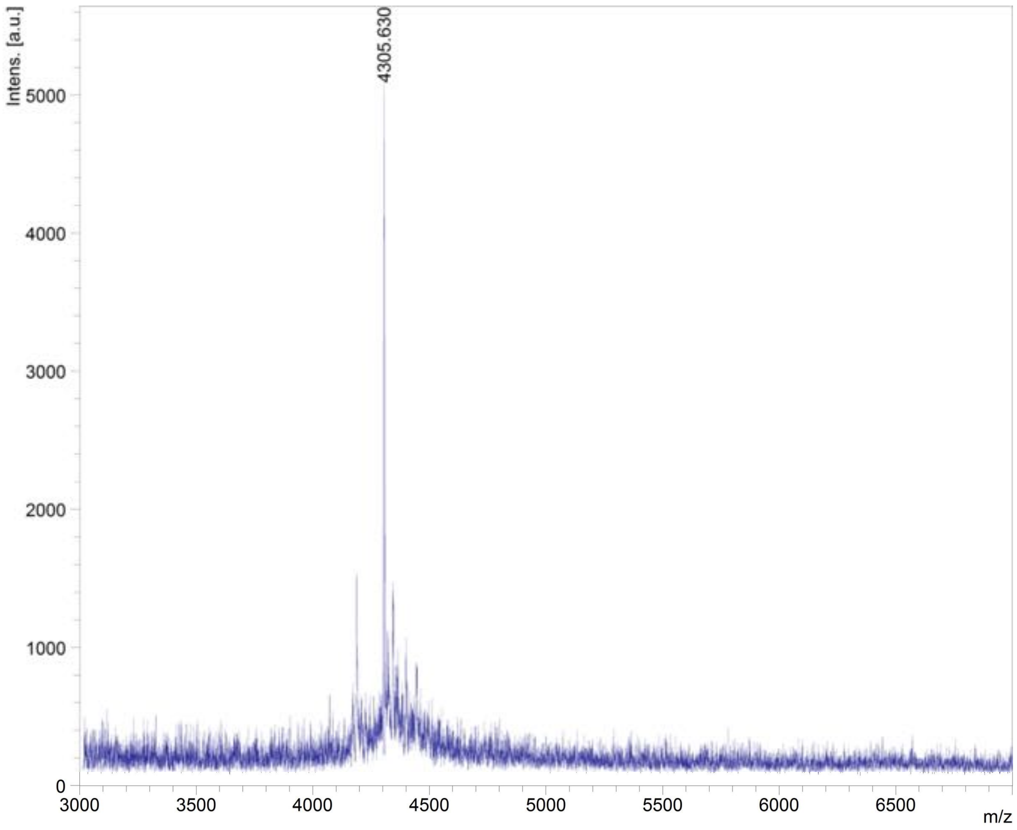

UD-79

[B conc. MeCN: 10-50% (30 min)]

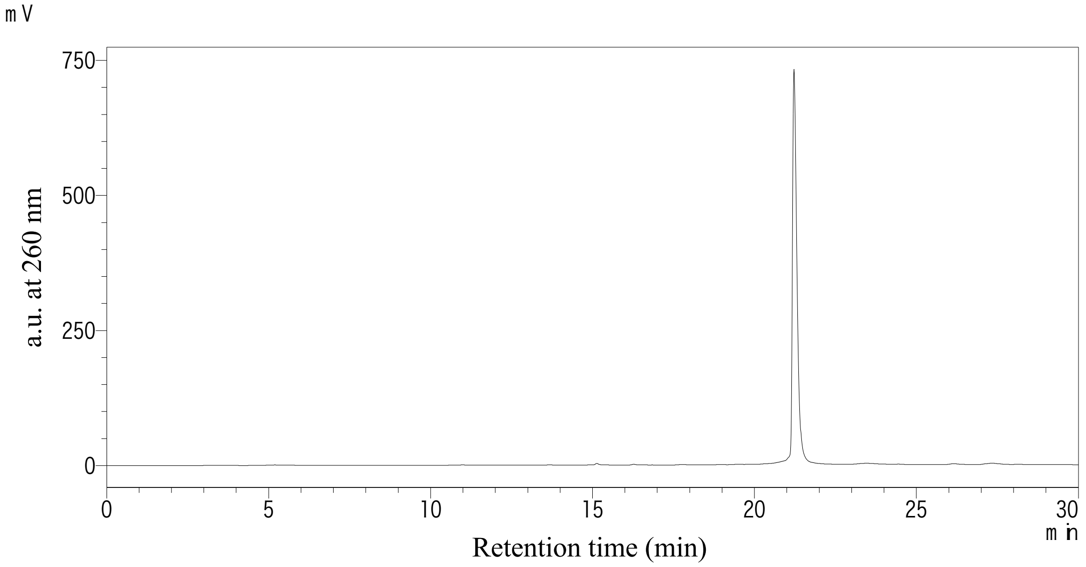

[calcd. (M-H)$^-$: 4391.3, found: 4390.4]

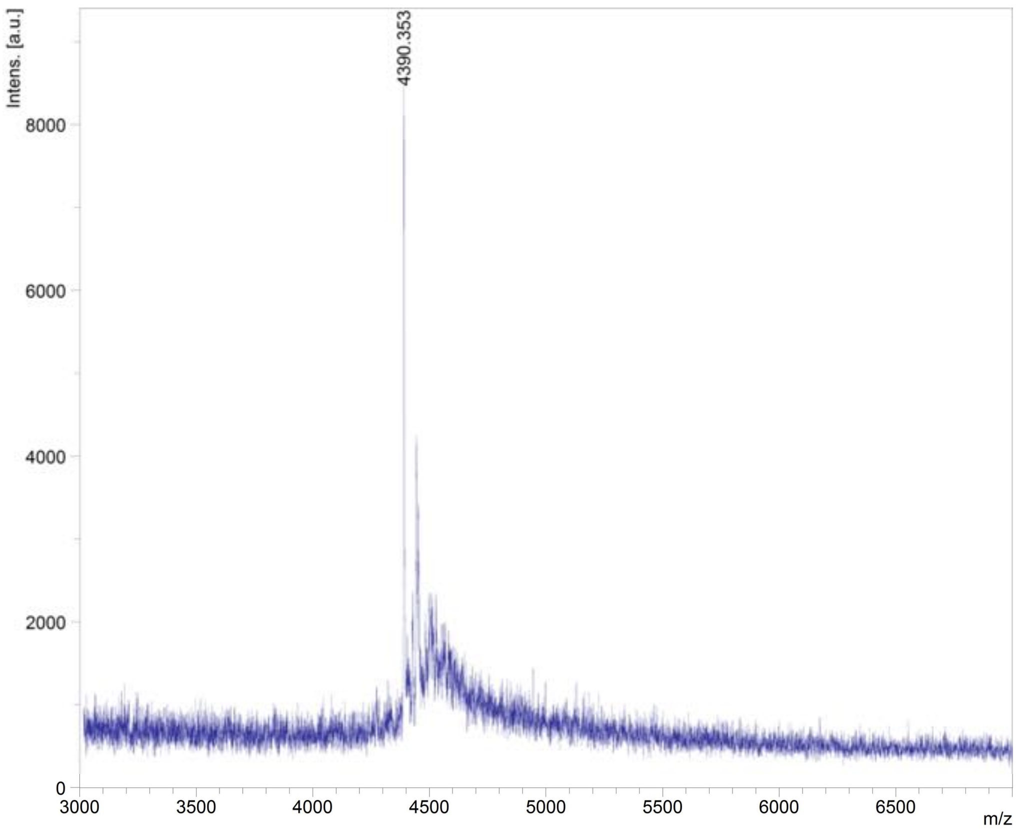

Supplement: CB-003-D1CB00240F-s001 [file CB-003-D1CB00240F-s001.pdf]
